# Illuminating protein space with a programmable generative model

**DOI:** 10.1038/s41586-023-06728-8

**Published:** 2023-11-15

**Authors:** John B. Ingraham, Max Baranov, Zak Costello, Karl W. Barber, Wujie Wang, Ahmed Ismail, Vincent Frappier, Dana M. Lord, Christopher Ng-Thow-Hing, Erik R. Van Vlack, Shan Tie, Vincent Xue, Sarah C. Cowles, Alan Leung, João V. Rodrigues, Claudio L. Morales-Perez, Alex M. Ayoub, Robin Green, Katherine Puentes, Frank Oplinger, Nishant V. Panwar, Fritz Obermeyer, Adam R. Root, Andrew L. Beam, Frank J. Poelwijk, Gevorg Grigoryan

**Affiliations:** Generate Biomedicines, Somerville, MA USA

**Keywords:** Protein design, Machine learning

## Abstract

Three billion years of evolution has produced a tremendous diversity of protein molecules^1^, but the full potential of proteins is likely to be much greater. Accessing this potential has been challenging for both computation and experiments because the space of possible protein molecules is much larger than the space of those likely to have functions. Here we introduce Chroma, a generative model for proteins and protein complexes that can directly sample novel protein structures and sequences, and that can be conditioned to steer the generative process towards desired properties and functions. To enable this, we introduce a diffusion process that respects the conformational statistics of polymer ensembles, an efficient neural architecture for molecular systems that enables long-range reasoning with sub-quadratic scaling, layers for efficiently synthesizing three-dimensional structures of proteins from predicted inter-residue geometries and a general low-temperature sampling algorithm for diffusion models. Chroma achieves protein design as Bayesian inference under external constraints, which can involve symmetries, substructure, shape, semantics and even natural-language prompts. The experimental characterization of 310 proteins shows that sampling from Chroma results in proteins that are highly expressed, fold and have favourable biophysical properties. The crystal structures of two designed proteins exhibit atomistic agreement with Chroma samples (a backbone root-mean-square deviation of around 1.0 Å). With this unified approach to protein design, we hope to accelerate the programming of protein matter to benefit human health, materials science and synthetic biology.

## Abstract (web summary)

Evolution has produced a range of diverse proteins, and now a generative model called Chroma can expand that set by allowing the user to design new proteins and protein complexes with desired properties and functions.

## Main

Protein molecules perform most of the biological functions necessary for life, but creating them is a complicated task that has taken billions of years of evolution. The field of computational protein design aims to shorten this process by automating the design of functional proteins in a programmable manner. Although there has been considerable progress towards this goal over the past three decades^2,3^, including the design of previously unknown topologies, assemblies, binders, catalysts and materials^4–7^, most de novo designs have yet to approach the complexity and variety of macromolecules that are found in nature. Reasons for this include the fact that modelling the relationship between sequence, structure and function is difficult, and most methods of computational design rely on iterative search and sampling processes that, just like evolution, must navigate a rugged fitness landscape incrementally^8^. Although many computational techniques have been developed to accelerate this search^3^ and to improve the prediction of natural protein structures^9^, the space of possible proteins remains combinatorially large and is only partly accessible to conventional computational methods. Determining how to efficiently explore the space of designable protein structures remains an open challenge.

An alternative and potentially appealing approach to protein design is to sample directly from the space of proteins that is compatible with a set of desired functions. Although this approach could address the fundamental limitation of iterative search methods, it would require a way to parameterize the a priori ‘plausible’ protein space, a way to draw samples from this space, and a way to bias this sampling towards desired properties and functions. Deep generative models have proven successful in solving these kinds of high-dimensional modelling and inference problems in other domains, for example in the text-conditioned generation of photorealistic images^10–12^. For this reason, there has been considerable work to develop generative models of protein space, applied to both protein sequences^13–19^ and structures^20–26^.

Despite recent advances in generative models for proteins, we argue that there are three properties that have yet to be realized simultaneously in one system. These are: modelling the joint, all-atom likelihood of sequences and three-dimensional structures of full protein complexes; achieving this with computation that scales sub-quadratically with the size of the protein system; and enabling conditional sampling under diverse design constraints without retraining. The first of these, generating full complexes, is important because proteins function by interacting with other molecules, including other proteins. The second, the sub-quadratic scaling of computation, is important because it has been an essential ingredient for managing complexity in other modelling disciplines, such as computer vision, in which convolutional neural networks scale linearly with the number of pixels in an image, and in computational physics, which uses fast *N*-body methods for the efficient simulation of everything from stellar systems to molecular ones^27^. Finally, the requirement to sample from a model without having to retrain it on new target functions is of considerable interest because protein design projects often involve many complex and composite requirements that may vary over time.

Here we introduce Chroma, a generative model for proteins that achieves all three of these requirements by modelling full complexes with quasi-linear computational scaling and by allowing arbitrary conditional sampling at generation time. It builds on the framework of diffusion models^28,29^, which model high-dimensional distributions by learning to gradually transform them into simple distributions in a reversible manner, and of graph neural networks^30,31^, which can efficiently process geometric information in complex molecular systems. We show that Chroma generates high-quality, diverse and innovative structures that refold both in silico and in crystallographic experiments, and that it enables the programmable generation of proteins conditioned on diverse properties such as symmetry, shape, protein class and even textual input. We anticipate that scalable generative models such as Chroma will enable a widespread and rapid increase in our ability to design and build protein systems that are fit for function.

## A scalable generative model for protein systems

**Fig. 1:**
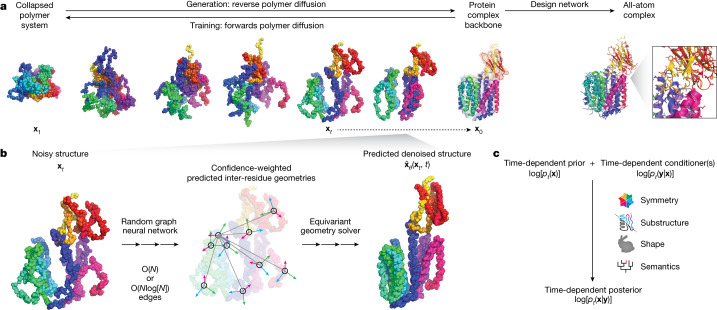
Chroma is a generative model for proteins and protein complexes that combines structured diffusion for protein backbones with scalable molecular neural networks for backbone synthesis and all-atom design. **a**, A correlated diffusion process with chain and radius-of-gyration constraints gradually transforms protein structures into random collapsed polymers (right to left). The reverse process (left to right) can be expressed in terms of a time-dependent optimal denoiser ${\widehat{{\bf{x}}}}_{\theta }\left({{\bf{x}}}_{t},t\right)$ that maps noisy coordinates ${{\bf{x}}}_{t}$ at time *t* to predicted denoised coordinates ${{\bf{x}}}_{0}$. **b**, We parameterize this in terms of a random graph neural network with long-range connectivity inspired by efficient *N*-body algorithms (middle) and a fast method for solving for a global consensus structure given predicted inter-residue geometries (right). Another graph-based design network (**a**, top right) generates protein sequences and side-chain conformations conditionally based on the sampled backbone. **c**, The time-dependent protein prior learnt by the diffusion model can be combined with composable restraints and constraints for the programmable generation of protein systems.

Chroma achieves high-fidelity, efficient generation of proteins by introducing a new diffusion process, neural-network architecture, and sampling algorithm based on principles from contemporary generative modelling and biophysical knowledge. Diffusion models generate data by learning to reverse a ‘noising’ process, which for previous image-modelling applications has typically been uncorrelated Gaussian noise. By contrast, our model learns to reverse a correlated noise process to match the distance statistics of natural proteins, which have scaling laws that are well understood from biophysics (Fig. 1a, Supplementary Appendix D). Previous generative models for protein structure have typically leveraged computation that scales quadratically, *O*(*N*^2^) (refs. ^24,25^), or cubically, *O*(*N*^3^) (refs. ^9,23^), in the number of residues *N*. This has either limited their application to small systems or required large amounts of computation for modestly sized systems. To overcome this problem, Chroma introduces a novel neural-network architecture (Fig. 1b, Supplementary Figs. 4–8, Supplementary Tables 2–3 and Supplementary Appendices E–G) for processing and updating molecular coordinates that uses random long-range graph connections with connectivity statistics inspired by fast *N*-body methods^27^ and that scales sub-quadratically (*O*(*N*) or *O*(*N*log[*N*]); Supplementary Fig. 4 and Supplementary Appendix E). We found that these modelling components improved performance, as measured by likelihood and in silico refolding across an ablation study of seven different model configurations (Supplementary Fig. 22 and Supplementary Appendix L). Finally, we introduce methods for low-temperature sampling with a modified diffusion process that allows us to trade an increased quality of sampled backbones (increasing likelihood) for reduced conformational diversity (reducing entropy; Supplementary Figs. 1–2, Supplementary Table 4 and Supplementary Appendix C). Given backbones from this diffusion process, the Chroma design network then generates sequence and side-chain conformations that are conditioned on the sampled backbone to yield a joint generative model for the sequences and structure of a protein complex. The design network is based on a similar graph neural-network architecture (Supplementary Figs. 7, 8 and 15), but with conditional sequence and side-chain decoding layers that build on previous studies^15,16^ that have seen further refinement and experimental validation^32–34^.

An important aspect of our diffusion-based framework is that it enables programmability of proteins through conditional sampling under combinations of user-specified constraints. This is made possible by a key property of diffusion models: they learn a process that transforms a simple distribution into a complex data distribution through a sequence of many infinitesimal steps. These ‘microscopic’ steps, therefore, can be biased or constrained by different user-specified requirements to produce a new conditional diffusion process at design time. We built on this with a diffusion-conditioner framework that allows us to automatically sample from arbitrary mixtures of hard constraints and soft penalties implemented as composable primitives (Fig. 1c and Supplementary Appendix M). We explored several conditioner primitives including geometrical constraints that can outfill proteins from fixed substructures (Supplementary Appendix N), enforce particular distances between atoms (Supplementary Appendix O), graft motifs into larger structures (Supplementary Appendix P), symmetrize complexes under arbitrary symmetry groups (Supplementary Appendix Q) and enforce shape adherence to arbitrary point clouds (Supplementary Appendix R). We also explored the possibilities of semantic prompting by training neural guidance networks that predict multi-scale protein classifications (Supplementary Appendix S) and natural language annotations (Supplementary Appendix T) from protein structures. We can invert these predictive models by sampling proteins that optimize classifier predictions. Any subset of conditioners may then be composed for bespoke, on-demand protein generation subject to problem-specific requirements.

## Analysis of unconditional samples

**Fig. 2:**
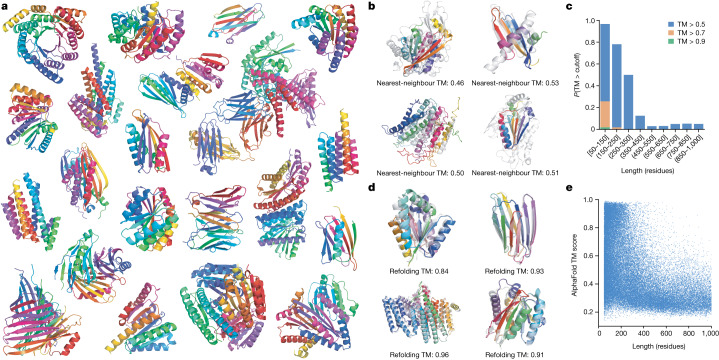
Analysis of unconditional samples reveals diverse geometries that exhibit new higher-order structures and refold in silico. **a**, A representative set of Chroma-sampled proteins and protein complexes exhibits complex and diverse topologies with high secondary-structure content, including familiar TIM (triose-phosphate isomerase) barrel-like folds (top left), antibody–antigen-like complexes (centre right) and new arrangements of helical bundles and β-sheets. **b**,**c**, Despite these qualitative similarities, samples frequently have low nearest-neighbour similarity to structures in the PDB, as measured by nearest-neighbour TM score ^41^ (**b**; Supplementary Appendix J.4), with structures demonstrating frequent novelty across length ranges (**c**). **d**,**e**, When we attempted to refold samples in silico using only a single sequence sample per structure, we observed widespread refolding with a high degree of superposition (**d**), including occasionally in the very high length range of more than 800 residues (**e**).

We sought to characterize the space of possible proteins parameterized by Chroma by generating a large number of unconditional samples of proteins and protein complexes (100,000 single-chain proteins and 20,000 complexes across two versions of the models, v.0 and v.1; Supplementary Appendix G and Supplementary Table 2). As can be seen in Fig. 2a, unconditional samples display many properties shared by natural proteins, such as complex layering of bundled α-helices and β-sheets in cooperative, unknotted folds. In some cases, we observed recognizable protein-complex configurations, including what seems to be an antibody–antigen complex in Fig. 2a (centre-right); note that the closest Protein Data Bank (PDB) structural matches to the two ‘antigen’ chains of this complex are at template-modelling (TM) scores^41^ of 0.46 and 0.43, indicating that this sample is not a result of memorization. We provide grids of random samples in Supplementary Figs. 9 and 10 for single-chain and complex structures, respectively. To quantitatively characterize the agreement of Chroma samples with natural proteins, we computed distributions of several key structural properties, including secondary-structure utilization, contact order^35^, length-dependent radius of gyration^36^, length-dependent long-range contact frequency and density of inter-residue contacts (Supplementary Table 5 and Supplementary Appendix J). We observe a general agreement of these statistics with corresponding distributions from the PDB (Supplementary Fig. 11), although we do see an overrepresentation of α-helices in the later version of Chroma (v.1) that seems to be a consequence of low-temperature sampling, which accentuates the already increased frequency of helices relative to strands in natural proteins (Supplementary Fig. 11b). Because these protein properties focus on low-order structural statistics, we also sought to characterize the extent to which they reproduce higher-order atomic geometries of natural protein structures. Natural protein structures exhibit considerable degeneracy in their use of local tertiary backbone geometries, such that completely unrelated proteins tend to use very similar tertiary motifs^37,38^. Chroma-generated structures exhibit the same type of degeneracy, utilizing natural tertiary motifs in a way that closely resembles native proteins, including complex tertiary geometries with four or five disjoint backbone fragments (Supplementary Fig. 11c and Supplementary Appendix J).

Although reproducing native-like properties of backbone geometries is important in design, our top priority is the extent to which the proteins can be realized as sequences that fold and function as intended. The definitive answer to this question involves experimental characterization (see below), but in silico evidence can be gathered more systematically. We sought to evaluate the fidelity of sequence–structure pairs generated by Chroma by measuring their agreement with three state-of-the-art methods for structure prediction^9,39,40^. We sampled one sequence for each backbone with Chroma’s design network and assessed whether each structure-prediction method would predict these sequences to fold into the corresponding generated structures (Supplementary Fig. 14 and Supplementary Appendix J). We observed widespread refolding of Chroma samples, whether stratified by protein length (Fig. 2e) or helical content and novelty (Supplementary Fig. 14). It is not surprising that successful refolding is less frequent for longer proteins, but it is remarkable that high TM scores^41^ are routinely achieved even for proteins more than 800 residues in length. Interestingly, helix content does not seem to be as strong of a predictor of refolding as the distance to the nearest neighbour in the PDB (Supplementary Fig. 15, middle and bottom rows, respectively). We note that this sequence–structure consistency test is not perfect because it rests on the assumption that structure-prediction models will generalize to new folds and topologies. However, the test does provide partial supporting evidence for the generation of realizable protein models in instances in which the predicted and generated structures have strong agreement.

Quantification of the structural homology between Chroma-generated samples and proteins in the PDB indicates that the model generates previously unseen structures at a frequency that increases sharply with length (Fig. 2c and Supplementary Fig. 12a). However, this analysis suffers from the problem that coverage of longer structures is expected to be lower in any finite database. To get a better understanding of the novelty of Chroma samples at different lengths, we defined a novelty score as the number of CATH^42^ domains required to greedily cover 80% of the residues in a protein at a TM score above 0.5, normalized by protein length (Supplementary Appendix J). Note that most valid proteins will be covered by at least some finite number of CATH domains because we retain even very small domains (such as single secondary-structural elements) in the coverage test. As shown in Supplementary Fig. 12c,d, there is a clear gap between native and Chroma-generated proteins by this metric, with most native backbones requiring approximately 2–5 times fewer CATH domains to be covered per length than generated backbones.

We also find that samples from Chroma are diverse and cover natural protein space. In Supplementary Fig. 13, we present samples from Chroma and a set of native structures with global topology descriptors derived from knot theory^43,44^ and embed them into two dimensions with UMAP^45^. The resulting embedding seems to be semantically meaningful because subsets of structures belonging to different categories by size and secondary structures cluster in this projection (sub-panels on the left in Supplementary Fig. 13a). False colour of the points in the embedding shows that novelty is spread broadly and is not biased to only certain types of structure space. This is especially clear when looking at a representative selection of samples shown in Supplementary Fig. 13b.

## Programmability

An important aspect of Chroma is its programmability, which means it is straightforward to specify high-level desired protein properties (such as symmetry groups) that are compiled into a set of sampling conditioners that bias the diffusion process towards these properties (Fig. 1c, Supplementary Fig. 23 and Supplementary Appendix M). To demonstrate the range of protein properties that can be programmed with conditional generation, we explored several composable conditioning primitives (Supplementary Table 6, Supplementary Figs. 23–33 and Supplementary Appendices N–T). Although we believe that each of these represents only a preliminary demonstration of possible conditioning modes, they provide a glimpse of the potential for programmable protein design.

**Fig. 3:**
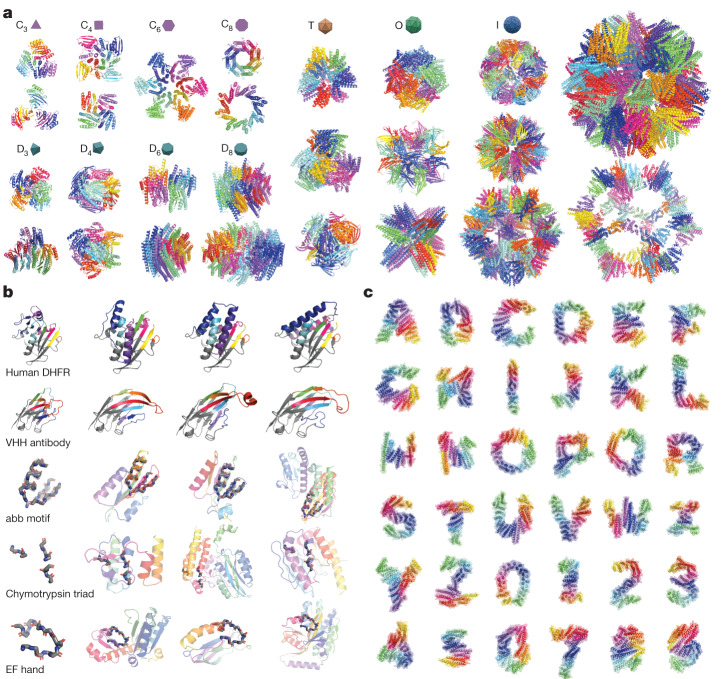
Symmetry, substructure and shape conditioning enable geometric molecular programming. **a**, Sampling oligomeric structures with arbitrary chain symmetries is possible by using a conditioner that tessellates an asymmetric subunit in the energy function. Cyclic (C_*n*_), dihedral (D_*n*_), tetrahedral (T), octahedral (O) and icosahedral (I) symmetry groups can produce a wide variety of possible homomeric complexes. The right-most protein complex contains 60 subunits and 60,000 total residues, which is enabled by leveraging symmetries and using our subquadratically scaling architecture. **b**, Conditioning on partial substructure (monochrome) enables protein infilling or outfilling. The top two rows illustrate regeneration (colour) of half a protein (the enzyme DHFR, first row) or complementarity-determining region loops of a VHH antibody (second row). The next three rows show conditioning on a predefined motif. The order and matching location of motif segments is not prespecified here. **c**, Conditioning on arbitrary volumetric shapes is exemplified by the complex geometries of the Latin alphabet and Arabic numerals. All structures were selected from protocols with high rates of in silico refolding (Supplementary Appendix K).

We began by considering analytic conditioners that can control protein backbone geometry. We found that conditioning on the symmetry of protein complexes can readily generate samples under arbitrary symmetry groups (Fig. 3a, Supplementary Figs. 17, 27–29 and Supplementary Appendix Q). Figure 3a illustrates symmetry-conditioned generation across many groups, from simple four-subunit cyclic symmetries up to a capsid-sized icosahedral complex with 60,000 total residues and more than 240,000 atoms. This also demonstrates why favourable computational scaling properties, such as quasilinear computation time (Supplementary Appendix E), are important, as efficient computation enables scaling to larger systems. Symmetric assemblies are common in nature and there have been some successes with de novo symmetric designs^46,47^, but it has generally been difficult to simultaneously optimize for both the desired overall symmetry and the molecular interaction details between protomers. Symmetry conditioning within the generation process in Chroma should make it simpler to sample structures that simultaneously meet both requirements.

We next explored substructure conditioning (Fig. 3b, Supplementary Figs. 16, 24–26, Supplementary Appendices N–P), which is a central problem for protein design because it can enable the preservation of one part of the structure of a protein (such as an active site) while modifying another part of the structure (and potentially function). In the top row, we cut the structure of human dihydrofolate reductase (DHFR; PDB code 1DRF) into two halves with a plane, remove one of the halves and regenerate the missing half. The cut plane introduces multiple discontinuities in the chain simultaneously, and the generative process must sample a solution that simultaneously satisfies these boundary conditions while being biophysically plausible. Nevertheless, the samples achieve both goals and, interestingly, do so in a manner very different from each other and from natural DHFR. In the second row of Fig. 3b, we cut out the complementarity-determining regions of a VHH antibody and rebuilt them conditioned on the remaining framework structure. Finally, the bottom three rows of Fig. 3b condition on sub-structure in an unregistered manner, meaning that the exact alignment of the substructure (motif) within the chain is not specified a priori, as it was in the previous examples. We outfilled the protein structure around several structural and functional motifs, including an αββ packing motif, backbone fragments encoding the catalytic triad active site of chymotrypsin and the EF-hand Ca-binding motif. Again, these motifs are accommodated in a realistic manner using diverse and structured solutions.

In Fig. 3c we provide an early demonstration of a more exotic kind of conditioning in which we attempted to solve for backbone configurations subjected to arbitrary volumetric shape specifications. We accomplished this by adding heuristic classifier gradients based on optimal transport distances^48^ between atoms in the structures and user-provided point clouds (Supplementary Appendix R). As a stress test of this capability, we conditioned the generation of single protein chains on the shapes of the Latin alphabet and Arabic numerals (Supplementary Fig. 18 and Supplementary Appendix K.3). We see the model routinely implementing several core phenomena of protein backbones, such as high secondary-structure content, close packing with room for designed side chains, and volume-spanning α-helical bundle and β-sheet elements. Although these shapes represent purely a challenging set of test geometries, more generally shape is intimately related to function in biology, for example, with membrane transporters, receptors and structured assemblies that organize molecular events in space. Being able to control shape would be a useful subroutine for generalized programmable protein engineering.

**Fig. 4:**
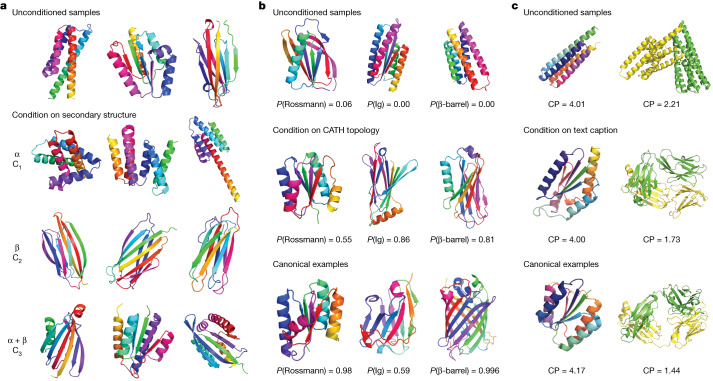
Protein structure classifiers and caption models can bias the sampling process towards user-specified properties. **a**, Neural networks trained to predict protein properties can bias unconditional samples (top) towards states that optimize predicted properties, such as secondary-structure composition (bottom) indicated by CATH class level codes (C1, Mainly Alpha; C2, Mainly Beta; C3, Alpha Beta). **b**, A neural network trained to predict CATH topology annotations can routinely drive generation towards samples with high predicted probabilities of the intended class label, which sometimes aligns with our intended fold topology for highly abundant labels. Left, highly abundant Rossmann fold (CATH topology 3.40.50, 14.0% of training set); middle, highly abundant Ig fold (CATH topology 2.60.40, 9.8% of training set); right, a rare specific β-barrel fold (CATH topology 2.40.155, 0.07% of training set). **c**, Fine-tuning a multi-label predictor to bias a pretrained large language model into a structure caption predictor can enable natural language conditioning. We begin to see examples of semantic alignment between prompts and output structures for highly abundant classes of structures, although we do not always see this reflected in the time-zero caption perplexity (CP, lower is better). Left, ‘crystal structure of a Rossmann fold’; right, ‘crystal structure of a Fab antibody fragment’.

Finally, we demonstrate in Fig. 4 that it is possible to condition on protein semantics, such as secondary structure, fold class (Fig. 4a, Supplementary Figs. 19, 30 and Supplementary Appendix S) and natural language (Fig. 4b, Supplementary Figs. 20, 31–33, and Supplementary Appendix T). Unlike geometric conditioning, in which the classifier is correct by construction (for example, the presence of a motif with less than a certain root-mean-square deviation is unambiguous), here the classifiers are neural networks trained on structure data, so there can be a discrepancy between the label assigned by the classifier and the ground truth class. Thus, for the fold-conditioned generation (Fig. 4a), we see that conditional samples always improve classifier probabilities over unconditioned samples taken from the same random seed, but the classification is not always perfect. For example, for the ‘Rossman fold’ class, the generated samples reproduce the canonical mixed topology. However, in the ‘Ig fold’ and ‘β-barrel fold’ examples, the structures exhibit some of the features characteristic of the classes (for example, β-sheets packed against each other) but do not contain all such features (for example, the Ig topology does not appear canonical and the barrel does not form a closed cycle). In Fig. 4b we demonstrate two examples of semantic conditioning on natural language captions, where we again occasionally observe alignment between samples and intended prompts, especially for highly-represented protein classes. It is exciting to imagine the potential of such a capability, that is being able to request desired protein features and properties directly through natural language prompts. Generative models such as Chroma can reduce the challenge of function-conditioned generation to the problem of building accurate classifiers for functions given structures. Although there is clearly much more work to be done to make this useful in practice, high-throughput experiments and evolutionary data are likely to enable this in the near term.

Supplementary Appendix K demonstrates extensive in silico refolding studies of samples generated with the conditioners described above. As shown in Supplementary Figs. 16–20, all of these conditional-generation processes can produce samples that refold accurately to their generated backbones. The rates at which this happens vary according to the specific condition and protein length (and are subject to the caveats of this test mentioned above), but even in the challenging cases of shape-, complex symmetry-, class- and language-conditioned designs, we observe widespread refolding across specific conditions and structure prediction methods.

## Experimental validation

To experimentally validate Chroma, we built a simple design protocol (based on Chroma v.0) that was intended to generate high-likelihood samples drawn from the model. Specifically, the protocol involved three steps: generate backbones by drawing independent samples from Chroma at low temperature; design sequences for each backbone using the Chroma design network; and automatically select a subset for experimental characterization to match the desired experimental scale, driven primarily by sequence and/or structure likelihood (as shown in Supplementary Table 7 and Supplementary Appendix U.1). Notably, we deliberately did not filter designs for refolding by a structure-prediction method or using any structure–energetic calculations. However, such filtering could potentially be used to improve the success rate of design.

**Fig. 5:**
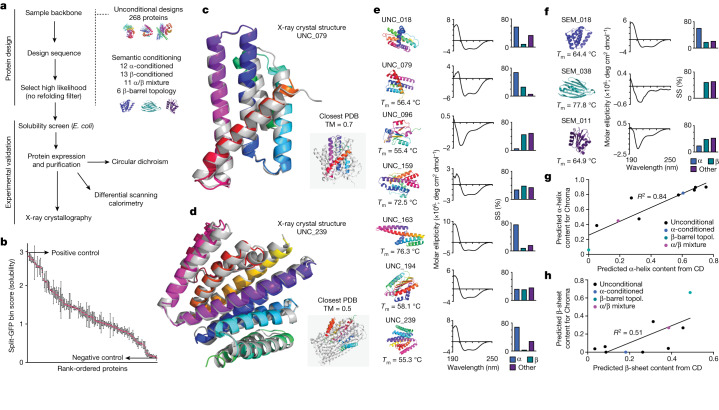
Experimental validation of Chroma-designed proteins. **a**, Protocol for protein design and experimental validation. Unconditional designs: 268 proteins. Semantic conditioning: 12 α-conditioned, 13 β-conditioned, 11 α/β mixtures and 6 with β-barrel topology. See text for details. **b**, Rank-ordered unconditional Chroma protein solubility scores by the split-GFP assay for 172 tested proteins. Red dots and error bars denote means and standard deviations, respectively, from three biological replicates. **c**,**d**, X-ray crystal structures (rainbow) of UNC_079 (**c**, 1.1 Å resolution, PDB 8TNM, root-mean-square deviation (RMSD) = 1.1 Å) and UNC_239 (**d**, 2.4 Å resolution, PDB 8TNO, RMSD = 1.0 Å) overlaid with Chroma-generated models (grey). Insets compare each crystal structure (rainbow) with its nearest PDB match (4NH2 and 6AFV, respectively; grey). **e**, CD data for seven purified Chroma proteins. The fraction of α-helical and β-strand content was determined using BeStSel^50^. *T*_m_ is the melting temperature determined by differential scanning calorimetry and SS designates secondary structure. **f**, CD data for three purified Chroma conditional designs: SEM_018 (α-conditioned), SEM_038 (β-barrel topology) and SEM_011 (α/β mixture). **g**,**h,** Correlation between predicted secondary-structure content in Chroma designs compared with the prediction from CD, for α-helical (**g**) and β-strand (**h**) content.

We generated 310 proteins (unconditional or semantically conditioned on CATH class or topology) for attempted expression and structural characterization (Fig. 5a). We first addressed an initial set of 172 unconditional proteins, ranging between 100 and 450 amino acids in length (Supplementary Fig. 36). We used a pooled protein solubility assay that was based on the split-GFP reporter system^49^ to prioritize tractable proteins for subsequent characterization (Supplementary Fig. 38a). After FACS and Nanopore sequencing (Supplementary Fig. 38b), enrichment scores were assigned to categorize the soluble expression levels of each protein (Supplementary Fig. 38c). All 172 tested proteins were assigned higher enrichment scores than the negative control (human β_3_ adrenergic receptor, Supplementary Table 8), indicating that a wealth of Chroma-designed unconditional proteins can be solubly expressed in *Escherichia* *coli* (Fig. 5b). We confirmed stable fluorescence in sorted cell populations (Supplementary Fig. 38d) and corroborated our split-GFP screen results using western blotting, observing soluble expression of 19 of the 20 top-scoring proteins and 0 of the 20 lowest-scoring proteins (Supplementary Fig. 39). We created an additional set of 96 unconditional Chroma proteins encompassing a wider range of lengths (from 100 to 950 amino acids; Supplementary Fig. 40a), which performed similarly to the first unconditional protein set using the split-GFP reporter assay (Supplementary Fig. 40b,c). In this additional set, soluble expression of nine of the ten top-scoring proteins was confirmed by western blotting (Supplementary Fig. 40d).

Of the proteins identified in the top 10% of the split-GFP solubility screen, we purified seven for interrogation using circular dichroism (CD; Fig. 5e) and differential scanning calorimetry (Supplementary Fig. 41 and Extended Data Table 1). The results indicate that most of the isolated proteins were stably folded with appreciable secondary structure. From these proteins, we were able to obtain X-ray crystal structures (Extended Data Table 2) for UNC_079 (PDB 8TNM; Fig. 5c) and UNC_239 (PDB 8TNO; Fig. 5d). The observed structures matched the anticipated designs to a high degree (root-mean-square deviation = 1.1 Å and 1.0 Å, respectively), indicating that Chroma-generated structures are realizable. Importantly, these structures are unique with respect to the PDB, with the top PDB hit to UNC_079 (PDB entry 4NH2, chain E) having query and target TM scores of 0.7 and 0.3, respectively, and the top hit to UNC_239 (PDB entry 6AFV, chain A) having query and target TM scores of 0.5 and 0.23, respectively (Fig. 5c,d).

The results of the split-GFP assay show that it is more difficult to succeed with longer designs, because there is an inverse correlation between length and split-GFP score (Supplementary Fig. 34). Interestingly, although we might expect the extent of refolding by structure prediction to also correlate with experimental success, we saw no correlation when length is corrected for (Supplementary Fig. 34). Similarly, we saw no correlation between soluble expression and structural novelty. We did find model likelihoods to be weakly predictive of experimental success for the first conditional set, but this did not hold true for the second set, in which lengths were extended up to 950 amino acids (Supplementary Fig. 35).

To test the ability of Chroma to propose well-behaved proteins in a conditioned setting, we next evaluated a set of 42 proteins conditioned by ProClass on CATH class (36 designs split among the classes mainly α, mainly β and mixed α/β) and on CATH topology (six designs conditioned on the β-barrel topology 2.40.155; Supplementary Fig. 37a). In the split-GFP solubility assay, 40 of these proteins (95%) scored above the negative control, indicating a high success rate of soluble protein expression (Supplementary Fig. 37b). We purified one representative protein from each secondary-structure category (two designs conditioned on mainly-*α* and mixed α/β classes, and one design conditioned on the β-barrel topology). Differential scanning calorimetry data for these proteins were consistent with relatively stable folding, with melting temperatures ranging from 64 °C to 78 °C (Supplementary Fig. 37c). On the basis of secondary-structure predictions from CD spectra^50^, we observed higher α-helical content in the mainly-α design, higher β-sheets in the β-barrel design, and mixed secondary structure in the mixed-content protein (Fig. 5f). Indeed, across both conditional and unconditional designs, the inferred secondary-structure content from CD was closely correlated with the secondary-structure content calculated from Chroma-generated models, for both the fraction of α-helices (*R*^2^ = 0.84; Fig. 5g) and β-sheets (*R*^2^ = 0.51; Supplementary Fig. 5h), indicating that proteins with various structural compositions can be designed by Chroma.

## Discussion

In this work we present Chroma, a generative model that can generate new and diverse proteins across a broad array of structures and properties. Chroma is programmable in the sense that it can sample proteins with a wide array of user-specified properties, including inter-residue distance and contact, domain, sub-structure and semantic specification from classifiers. Chroma is able to generate proteins that have arbitrary and complex shapes, and it has even begun to demonstrate the ability to accept descriptions of desired properties as free text. Its efficient design, with an innovative diffusion process, quasilinear scaling neural architecture and low-temperature sampling method, means that Chroma can generate extremely large proteins and protein complexes (with more than 3,000 residues) on a commodity graphics processing unit (such as an NVIDIA V100) in a few minutes.

We reasoned that the best way to determine the plausibility of the protein space parameterized by Chroma was to draw independent samples from the model and test them experimentally. Note that this is a departure from the prototypical protein-design protocol, in which initial proposal designs are down-selected using a custom set of filters intended to avoid known or hypothesized model deficiencies and help focus on designs that are more likely to work experimentally. Although the latter practice, which is broadly adopted in the field, can be effective at increasing design success rates, it does require a custom set of filters for each design project and makes fully automated design difficult to achieve. Furthermore, such an approach would detract from our intention of characterizing the distribution learned by Chroma.

Our experimental validation shows that Chroma has learnt a sufficiently accurate distribution such that sampling from it results in proteins that express, fold, have favourable biophysical properties and conform to intended structures at non-trivial rates. Even under the highly conservative view that only the proteins we purified and characterized individually in solution constitute successful designs (as opposed to others that performed comparably by split-GFP, for example), Chroma would still have a 3% success rate. Moreover, the two designs with experimentally determined crystal structures demonstrate that a non-trivial fraction of this distribution should be expected to be atomistically accurate. Given the breadth and novelty of the structure space learned by Chroma (Fig. 2 and Supplementary Figs. 9, 10 and 13), even these conservative estimates of success rate would translate into immense swaths of unexplored actionable protein space that can now be accessible through commodity computing hardware.

The task of exploring protein structure space in a way that can produce physically reasonable and designable conformations has been a long-standing challenge in protein design. In a few protein systems, it has been possible to parameterize the backbone conformation space mathematically—most notably the α-helical coiled coil^51^ and a few other cases that have high symmetry^52^—and in these cases, design efforts have benefited tremendously, creating possibilities that are not available in other systems^52,53^. For all other structure types, however, a great amount of computational time has been spent on the search for reasonable backbones, often leaving the focus on actual functional specifications out of reach. Chroma has the potential to address this problem, enabling a shift from focusing on generating feasible structures towards a focus on the specific task at hand—namely, what the protein is intended to do. By leveraging proteins sampled over more than 3 billion years of evolution, and by finding new ways to assemble stable protein matter, generative models such as Chroma are well poised to drive another expansion of biomolecular diversity with benefits for human health and bioengineering.

### Reporting summary

Further information on research design is available in the Nature Portfolio Reporting Summary linked to this article.

## Online content

Any methods, additional references, Nature Portfolio reporting summaries, source data, extended data, supplementary information, acknowledgements, peer review information; details of author contributions and competing interests; and statements of data and code availability are available at 10.1038/s41586-023-06728-8.

### Supplementary information

Supplementary Information Extended descriptions of the Chroma model, sampling algorithms, conditioners, in silico evaluations, an ablation study, training pipelines and the experimental validation. It includes appendices, Supplementary figures and Supplementary tables.

Reporting Summary

Peer Review File

## Figures and Tables

**Extended Data Table 1:**
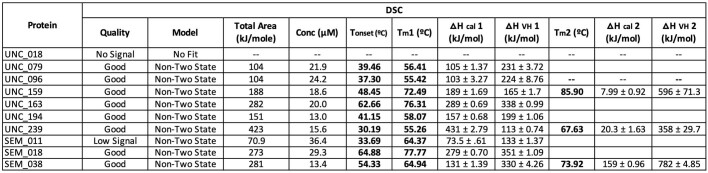
Differential scanning calorimetry data

Differential scanning calorimetry data for Chroma proteins evaluated experimentally.

**Extended Data Table 2:**
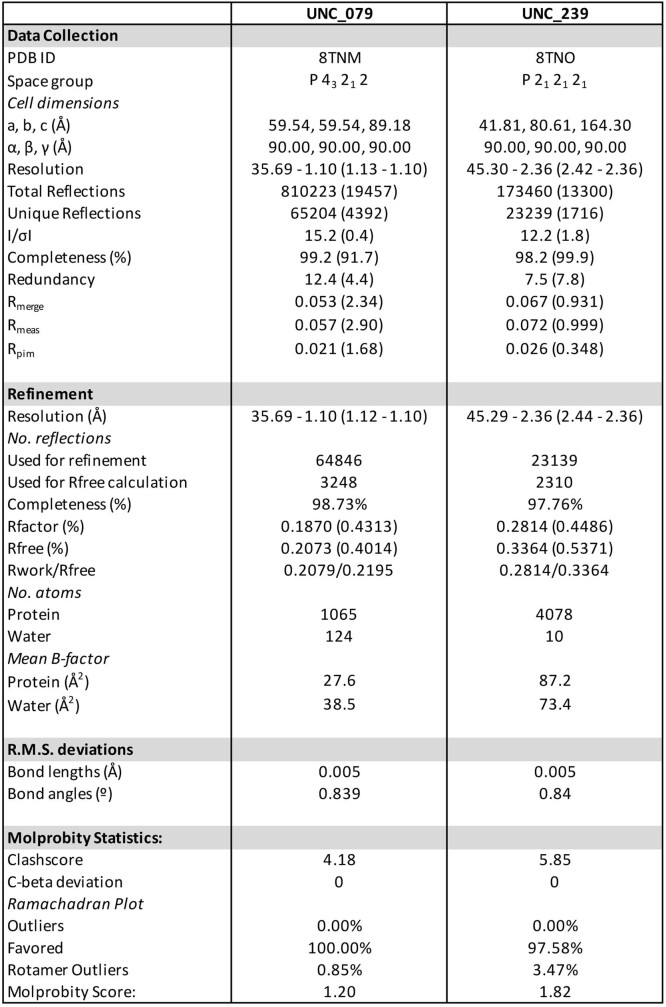
X-ray crystallography data collection and refinement statistics

Statistics related to protein X-ray crystal structures solved in this article. Values in parentheses are for the highest-resolution shell.

## Data Availability

All experimental and computational results are available in the Supplementary Information and Extended Data Tables 1 and 2. Experimental structures solved as part of this study were deposited under PDB accession codes 8TNM and 8TNO. Training datasets were constructed based on the PDB (https://www.rcsb.org/), as queried on 20 March 2022, UniProt 2022_01 (https://www.uniprot.org) and PFAM 35 (http://pfam.xfam.org/). PDB IDs comprising Chroma training, test and validation sets are available in the Zenodo dataset at 10.5281/zenodo.8285077.

## Code availability

Chroma code is available at https://github.com/generatebio/chroma under the Apache 2.0 open-source licence.

## References

[1] The UniProt Consortium. (2021). UniProt: the universal protein knowledgebase in 2021. Nucleic Acids Res..

[2] Kuhlman B, Bradley P (2019). Advances in protein structure prediction and design. Nat. Rev. Mol. Cell Biol..

[3] Huang P-S, Boyken SE, Baker D (2016). The coming of age of de novo protein design. Nature.

[4] Koga N (2012). Principles for designing ideal protein structures. Nature.

[5] Cao L (2022). Design of protein-binding proteins from the target structure alone. Nature.

[6] Kries H, Blomberg R, Hilvert D (2013). De novo enzymes by computational design. Curr. Opin. Chem. Biol..

[7] Joh NH (2014). De novo design of a transmembrane Zn^2+^-transporting four-helix bundle. Science.

[8] Smith JM (1970). Natural selection and the concept of a protein space. Nature.

[9] Jumper J (2021). Highly accurate protein structure prediction with AlphaFold. Nature.

[10] Ramesh, A. et al. Zero-shot text-to-image generation. In *Proc. 38th International Conference on Machine Learning* (eds Meila, M. et al.) 8821–8831 (PMLR, 2021).

[11] Ramesh, A., Dhariwal, P., Nichol, A., Chu, C. & Chen, M. Hierarchical text-conditional image generation with CLIP latents. Preprint at https://arxiv.org/abs/2204.06125 (2022).

[12] Saharia, C. et al. Photorealistic text-to-image diffusion models with deep language understanding. In *Proc.**Advances in Neural Information Processing Systems**35* (eds Koyejo, S. et al.) 36479–36494 (NeurIPS, 2022).

[13] Riesselman AJ, Ingraham JB, Marks DS (2018). Deep generative models of genetic variation capture the effects of mutations. Nat. Methods.

[14] Greener JG, Moffat L, Jones DT (2018). Design of metalloproteins and novel protein folds using variational autoencoders. Sci. Rep..

[15] Ingraham, J., Garg, V., Barzilay, R. & Jaakkola, T. Generative models for graph-based protein design. In *Proc.**Advances in Neural Information Processing Systems**32* (eds Wallach, H. et al.) (NeurIPS, 2019).

[16] Anand N (2022). Protein sequence design with a learned potential. Nat. Commun..

[17] Madani, A. et al. ProGen: language modeling for protein generation. Preprint at http://arxiv.org/abs/2004.03497 (2020).

[18] Rives A (2021). Biological structure and function emerge from scaling unsupervised learning to 250 million protein sequences. Proc. Natl Acad. Sci. USA.

[19] Notin, P. et al. Tranception: protein fitness prediction with autoregressive transformers and inference-time retrieval. In *Proc. 39th International Conference on Machine Learning* (eds Chaudhuri, K. et al.) 16990–17017 (PMLR, 2022).

[20] Anand, N. & Huang, P.-S. Generative modeling for protein structures. In *Proc. Advances in Neural Information Processing Systems**31* (eds Bengio, S. et al.) (NeurIPS, 2018).

[21] Lin, Z., Sercu, T., LeCun, Y. & Rives, A. Deep generative models create new and diverse protein structures. In *Machine Learning in Structural Biology Workshop at the 35th Conference on Neural Information Processing Systems* (MLSB, 2021).

[22] Eguchi RR, Choe CA, Huang P-S (2022). Ig-VAE: Generative modeling of protein structure by direct 3D coordinate generation. PLoS Comput. Biol..

[23] Anand, N. & Achim, T. Protein structure and sequence generation with equivariant denoising diffusion probabilistic models. Preprint at https://arxiv.org/abs/2205.15019 (2022).

[24] Trippe, B. L. et al. Diffusion probabilistic modeling of protein backbones in 3D for the motif-scaffolding problem. In *Proc. 11th International Conference on Learning Representations* (eds Kim, B. et al.) (OpenReview.net, 2023).

[25] Wu, K. E. et al. Protein structure generation via folding diffusion. Preprint at https://arxiv.org/abs/2209.15611 (2022). 10.1038/s41467-024-45051-2 PMC10844308 38316764

[26] Watson JL (2023). De novo design of protein structure and function with RFdiffusion. Nature.

[27] Barnes J, Hut P (1986). A hierarchical *O(N log N)* force-calculation algorithm. Nature.

[28] Sohl-Dickstein, J., Weiss, E., Maheswaranathan, N. & Ganguli, S. Deep unsupervised learning using nonequilibrium thermodynamics. In *Proc. 32nd International Conference on Machine Learning* Vol. 27 (eds Bach, F. et al.) 2256–2265 (PMLR, 2015).

[29] Song, Y. et al. Score-based generative modeling through stochastic differential equations. In *International Conference on Learning Representations* (eds Hofmann, K. et al.) (OpenReview.net, 2021).

[30] Gilmer, J., Schoenholz, S. S., Riley, P. F., Vinyals, O. & Dahl, G. E. Neural message passing for quantum chemistry. In *Proc. 34th International Conference on Machine Learning* (eds Precup, D. et al.) 1263–1272 (PMLR, 2017).

[31] Battaglia, P. W. et al. Relational inductive biases, deep learning, and graph networks. Preprint at https://arxiv.org/abs/1806.01261 (2018).

[32] Jing, B., Eismann, S., Suriana, P., Townshend, R. J. L. & Dror, R. Learning from protein structure with geometric vector perceptrons. In *International Conference on Learning Representations* (eds Hofmann, K. et al.) (OpenReview.net, 2021).

[33] Hsu, C. et al. Learning inverse folding from millions of predicted structures. In *Proc. 39th International Conference on Machine Learning* Vol. 162 (eds Chaudhuri, K. et al.) 8946–8970 (PMLR, 2022).

[34] Dauparas J (2022). Robust deep learning–based protein sequence design using ProteinMPNN. Science.

[35] Plaxco, K. W., Simons, K. T. & Baker, D. Contact order, transition state placement and the refolding rates of single domain proteins. *J. Mol. Biol.* **277**, 985–994 (1998). 10.1006/jmbi.1998.1645 9545386

[36] Tanner JJ (2016). Empirical power laws for the radii of gyration of protein oligomers. Acta Crystallogr. D.

[37] Mackenzie CO, Zhou J, Grigoryan G (2016). Tertiary alphabet for the observable protein structural universe. Proc. Natl Acad. Sci. USA.

[38] Zhou J, Panaitiu AE, Grigoryan G (2020). A general-purpose protein design framework based on mining sequence–structure relationships in known protein structures. Proc. Natl Acad. Sci. USA.

[39] Wu, R. et al. High-resolution de novo structure prediction from primary sequence. Preprint at *bioRxiv*10.1101/2022.07.21.500999 (2022).

[40] Lin Z (2023). Evolutionary-scale prediction of atomic-level protein structure with a language model. Science.

[41] Zhang Y, Skolnick J (2005). TM-align: a protein structure alignment algorithm based on the TM-score. Nucleic Acids Res..

[42] Sillitoe I (2021). CATH: increased structural coverage of functional space. Nucleic Acids Res..

[43] Røgen P, Fain B (2003). Automatic classification of protein structure by using Gauss integrals. Proc. Natl Acad. Sci. USA.

[44] Harder T, Borg M, Boomsma W, Røgen P, Hamelryck T (2012). Fast large-scale clustering of protein structures using Gauss integrals. Bioinformatics.

[45] McInnes, L., Healy, J., Saul, N. & Großberger, L. UMAP: Uniform Manifold Approximation and Projection. *J. Open Source Softw.* **3**, 861 (2018).

[46] Wicky BIM (2022). Hallucinating symmetric protein assemblies. Science.

[47] King NP (2014). Accurate design of co-assembling multi-component protein nanomaterials. Nature.

[48] Peyré G, Cuturi M (2019). Computational optimal transport: with applications to data science. Found. Trends Mach. Learn..

[49] Cabantous S, Terwilliger TC, Waldo GS (2005). Protein tagging and detection with engineered self-assembling fragments of green fluorescent protein. Nat. Biotechnol..

[50] Micsonai A (2022). BeStSel: webserver for secondary structure and fold prediction for protein CD spectroscopy. Nucleic Acids Res..

[51] Grigoryan G, DeGrado WF (2011). Probing designability via a generalized model of helical bundle geometry. J. Mol. Biol..

[52] Woolfson DN (2015). De novo protein design: how do we expand into the universe of possible protein structures?. Curr. Opin. Struct. Biol..

[53] Beesley JL, Woolfson DN (2019). The de novo design of α-helical peptides for supramolecular self-assembly. Curr. Opin. Biotechnol..

