# Supplementary material for: Illuminating protein space with a programmable generative model
Source: Nature. 2023 Nov 15;623(7989):1070–8. doi: 10.1038/s41586-023-06728-8 (PMC10686827; doi:10.1038/s41586-023-06728-8)
Supplement: Supplementary file 1 — Extended descriptions of the Chroma model, sampling algorithms, conditioners, in silico evaluations, an ablation study, training pipelines and the experimental validation. It includes appendices, Supplementary figures and Supplementary tables. [file 41586_2023_6728_MOESM1_ESM.pdf]

nature portfolio

https://doi.org/10.1038/s41586-023-06728-8

Supplementary information

Illuminating protein space with a programmable generative model

In the format provided by the authors and unedited

Supplementary Information for: Illuminating protein space with a programmable generative model

John B Ingraham, Max Baranov, Zak Costello, Karl W Barber, Wujie Wang, Ahmed Ismail, Vincent Frappier, Dana M Lord, Christopher Ng-Thow-Hing, Erik R Van Vlack, Shan Tie, Vincent Xue, Sarah C Cowles, Alan Leung, João V Rodrigues, Claudio L Morales-Perez, Alex M Ayoub, Robin Green, Katherine Puentes, Frank Oplinger, Nishant V Panwar, Fritz Obermeyer, Adam R Root, Andrew L Beam, Frank J Poelwijk, Gevorg Grigoryan

Generate Biomedicines, Inc.

Supplementary Information

Table of Contents

A Overview **7**
A.1 Model . . . 7
A.2 Conditioners . . . 9
A.3 Wet lab experiments . . . 10

B Diffusion Models with Structured Correlations **11**
B.1 Correlated diffusion as uncorrelated diffusion in a transformed space . . . 11
B.2 Scheduling diffusion to balance signal and noise . . . 12
B.3 Training with likelihood on an Evidence Lower Bound (ELBO) . . . 14
B.4 Auxiliary training objectives . . . 15
B.5 Sampling with the Reverse-time SDE . . . 18
B.6 Sampling with the Probability Flow ODE . . . 18
B.7 Conditional sampling from the posterior under auxiliary constraints . . . 19
B.8 Related work . . . 20

C Low-Temperature Sampling for Diffusion Models **20**
C.1 Reverse-time SDE with temperature annealing . . . 21
C.2 Annealed Langevin Dynamics SDE . . . 23
C.3 Hybrid Langevin Reverse-time SDE . . . 24

D Polymer-Structured Diffusions **26**
D.1 Diffusion processes predictably affect molecular distances . . . 26
D.2 Covariance model #1: Ideal Chain . . . 28
D.3 Covariance model #2: R_g-confined Globular Polymer . . . 29
D.4 Alternative covariance model: Residue Gas . . . 35

E Random Graph Neural Networks **36**
E.1 Background: efficient N-body simulation . . . 36
E.2 Random graph generation . . . 37
E.3 Computational complexity . . . 37

F Structure from Inter-residue Geometry Predictions **39**

F.1 Background and motivation 39
F.2 Equivariant structure updates via convex optimization 39
F.3 Equivariant prediction of backbone atoms 44
F.4 Time-dependent post-prediction scaling 44

G Chroma Architecture **45**
G.1 Graph neural networks for protein structure 45
G.2 ChromaBackbone 48
G.3 ChromaDesign 48
G.4 Related Work 48

H Training **51**
H.1 Dataset 51
H.2 Optimization 51

I Sampling **52**
I.1 Sampling backbones 52
I.2 Sampling sequences 53

J Evaluation: Unconditional Samples **54**
J.1 Sample generation 54
J.2 Backbone geometry statistics 54
J.3 Tertiary motif analysis 56
J.4 Novelty analysis 60
J.5 Refolding analysis 61
J.6 Sequence design analysis 61

K Evaluation: Conditional Samples **62**
K.1 Refolding substructure-conditioned samples 63
K.2 Refolding symmetry-conditioned samples 64
K.3 Refolding shape-conditioned samples 65
K.4 Refolding class-conditioned samples 65
K.5 Refolding language-conditioned samples 68
K.6 Analysis of structure prediction confidence versus refolding TM 69

L Evaluation: Ablation Study **69**
L.1 Alternate model configurations and training 70

L.2 Ablation results . . . 72

M Programmability: Conditioners framework 74

M.1 Bayes' theorem for score functions . . . 74

M.2 Conditioners: motivation . . . 75

M.3 Conditioners . . . 78

M.4 Example applications of constraint composition . . . 79

M.5 Related work . . . 79

N Programmability: Substructure Constraints 80

N.1 Motivation . . . 80

N.2 Approach . . . 82

O Programmability: Substructure Distances 83

O.1 Motivation . . . 83

O.2 Approach . . . 83

P Programmability: Substructure Motifs 85

P.1 Motivation . . . 85

P.2 Approach . . . 85

Q Programmability: Symmetry 86

Q.1 Motivation . . . 86

Q.2 Symmetry breaking in sampling . . . 86

Q.3 Symmetric transformation as a conditioner . . . 87

Q.4 Practical implementation with scaling and composition . . . 89

Q.5 Additional symmetric samples . . . 90

R Programmability: Shape 91

R.1 Motivation . . . 91

R.2 Approach . . . 92

S Programmability: Classification 94

S.1 Motivation . . . 94

S.2 Model inputs . . . 94

S.3 Featurization . . . 94

S.4 Architecture . . . 94

S.5 Labels and loss functions . . . 95

S.6 Training . . . 95

S.7 Hyperparameters . . . 95

T Programmability: Natural Language Annotations 95

T.1 Motivation . . . 95
T.2 Dataset curation . . . 96
T.3 Model architecture . . . 97
T.4 Model training . . . 98
T.5 Performance . . . 98

U Experimental Validation 100

U.1 Protein design . . . 100
U.2 Experimental methods . . . 100
U.3 Experimental figures . . . 109
U.4 Experimental tables . . . 115

List of Figures

1 Low temperature sampling with Hybrid Langevin SDE . . . 22
2 Low temperature sampling analysis, proteins . . . 25
3 Polymer-structured diffusions for proteins . . . 27
4 Random graph sampling for random graph neural networks . . . 38
5 Equivariant structure updates from inter-residue geometries . . . 40
6 Anisotropic confidence models for predicted inter-residue geometries. . . . 43
7 Chroma architecture . . . 46
8 Randomized autoregression orders with varying spatial clustering . . . 50
9 Random single-chain samples . . . 55
10 Random complex samples . . . 56
11 Structural metrics analysis of unconditional samples . . . 57
12 Novelty analysis of unconditional samples . . . 58
13 The protein space of unconditional samples . . . 59
14 Refolding analysis of unconditional samples . . . 61
15 Sequence recovery evaluation . . . 62
16 Refolding analysis for substructure conditioning . . . 63
17 Refolding analysis for symmetry conditioning . . . 64
18 Refolding analysis for shape conditioning . . . 66
19 Refolding analysis for class conditioning . . . 67
20 Refolding analysis for natural language conditioning . . . 68

21 Evaluation of structure prediction confidence versus agreement 69
22 Ablation study of novel model components 71
23 Programmable design with diffusion conditioners 76
24 Substructural infilling with globular covariance 81
25 Substructural infilling examples 82
26 RMSD conditioning: Motifs can occur in entirely unrelated structural contexts . . 85
27 Constrained transformations for symmetry operations. 90
28 Symmetric complex samples 91
29 Symmetric complexes with poor contacts 92
30 Architecture: ProClass model 94
31 Architecture: ProCap model 96
32 ProCap evaluation metrics 99
33 ProCap-guided sample predictions of CATH class 100
34 In silico scores scatter plot to split GFP and length 107
35 In silico scores partial correlation to split GFP. 107
36 Unconditional protein designs 109
37 Secondary structure conditional designs 110
38 Split-GFP protein solubility assay 111
39 Soluble protein expression confirmation via western blot 112
40 Evaluation of additional set of unconditional protein designs 113
41 Differential scanning calorimetry experiments 114

List of Tables

1 Notation 7
2 Hyperparameters for the backbone network 48
3 Hyperparameters for the design network 49
4 Hyperparameters for sampling 52
5 Structural metrics for backbones 57
6 Conditioners 80
7 Design protocol 101
8 Split-GFP control sequences 115

Symbol	Definition
N	number of atoms or residues
$\mathbf{x}_t \in \mathbb{R}^{N\times 3}$	coordinates sampled at time t
$\mathbf{x}_t^{\mathcal{M}} \in \mathbb{R}^{\lvert\mathcal{M}\rvert\times 3}$	motif-sliced coordinates based on index set $\mathcal{M} \subset [\![1,N]\!]$
$\mathbf{x}_t^{(i)} \in \mathbb{R}^3$	the i_{th} coordinate in $\mathbf{x}_t$
$\mathcal{G} = (\mathcal{V},\mathcal{E})$	a graph composed of sets of vertices and edges
$D^{ij},\ D_{ij}$	Euclidean distance between i and j $\lVert\mathbf{x}^{(i)} - \mathbf{x}^{(j)}\rVert_2$
$\mathbf{z} \in \mathbb{R}^{N\times 3}$	whitened noise, and z_i is the individual noise component
$\Sigma = \mathbf{R}\mathbf{R}^\intercal$	covariance matrix for polymer-structured prior, $[\mathbf{Rz}]_{ik} = \sum_j [\mathbf{R}]_{ij}\mathbf{z}_{jk}$
$\mathbf{T} = (\mathbf{O},\mathbf{t})$	Euclidean transformation with rotation $\mathbf{O}$ and translation $\mathbf{t}$
α_t	time-dependent mean scaling in the forwards diffusion
σ_t	time-dependent variance scaling in the forwards diffusion
h_t	time-dependent generalized drift coefficient
g_t	time-dependent generalized diffusion coefficient
β_t	time-dependent noise schedule for variance-preserving process
λ_t	time-dependent inverse temperature
ψ	Langevin equilibration rate in Hybrid SDE
T	number of integration time steps
$\hat{\mathbf{x}}_\theta$	denoising network in Cartesian space
$\hat{\mathbf{z}}_\theta$	denoising network in the whitened space
$\nabla_{\mathbf{x}} \log p_t(\mathbf{x},t)$	score estimator network
$d\mathbf{w}$, $d\bar{\mathbf{w}}$	forward Brownian noise, reverse Brownian noise

Supplementary Table 1: **Table of notation**

A Overview

A.1 Model

Factorization Chroma is a joint generative model for the all-atom structures of protein complexes given a set of chain lengths. To model this complex set of dependencies across mixed continuous and discrete degrees of freedom, we factorize the joint distribution into parameterized components as

$$\log p_\theta(\mathbf{x},\mathbf{s},\chi) = \underbrace{\log p_\theta(\mathbf{x})}_{\text{backbone likelihood}} + \underbrace{\log p_\theta(\mathbf{s}|\mathbf{x})}_{\text{sequence likelihood}} + \underbrace{\log p_\theta(\chi|\mathbf{x},\mathbf{s})}_{\text{side-chain likelihood}},$$

where $\mathbf{x} \in \mathbb{R}^{4N\times 3}$ represents the backbone heavy atom coordinates (i.e., N, C_α, C, and O), $\mathbf{s} \in [\![20]\!]^N$ represents the discrete sequences over all residues, $\chi \in (-\pi,\pi]^{4N}$ represents the side-chain torsional angles, θ represents model parameters, and N is the total number of residues in the complex[1]. We parameterize these component distributions in terms of two neural networks: a backbone network which uses diffusion modeling to estimate $\log p_\theta(\mathbf{x})$ and a design network which uses discretely supported distributions to estimate $\log p_\theta(\chi,\mathbf{s}|\mathbf{x})$. Both networks are based on a graph neu-

[1]We drop explicit dependence on chain lengths throughout this work to simplify notation.

ral network architecture which takes SE(3)-invariant features as inputs and outputs SE(3)-invariant scalars and SE(3)-equivariant coordinates as needed (Supplementary Appendix G).

Diffusion process Our diffusion modeling approach builds upon standard methods with extensions for correlated diffusion processes (Supplementary Appendix B). Briefly, we define a forwards noising process which destroys structure in data as

$$\mathbf{x}_t \sim p(\mathbf{x}_t|\mathbf{x}_0) = \mathcal{N}\left(\mathbf{x}; \alpha_t \mathbf{x}_0, \left(1-\alpha_t^2\right)\mathbf{R}\mathbf{R}^\intercal\right),$$

where $\mathbf{R}\mathbf{R}^\intercal$ is the covariance matrix of the diffusion process and α_t is a signal-erasing schedule that decays monotonically from 1 to 0 as the time t goes from 0 to 1. This can also be effectively simulated by the Ornstein–Uhlenbeck process

$$\mathrm{d}\mathbf{x} = -\frac{1}{2}\beta_t \mathbf{x}\,\mathrm{d}t + \sqrt{\beta_t}\,\mathrm{d}\mathbf{R}\mathbf{w},$$

where $-\frac{1}{2}\beta_t = \frac{\mathrm{d}\log\alpha_t}{\mathrm{d}t}$ is a time-dependent noising schedule[2]. We design the covariance matrix $\mathbf{R}\mathbf{R}^\intercal$ to respect the distance statistics of natural proteins, including local chain constraints and global density constraints based on the scaling law $R_g \approx 2. \times N^{0.4}$ (Supplementary Appendix D). We also show how to generalize this framework for arbitrary Gaussian noising schedules in Supplementary Appendix B.

Diffusion objective Given this forwards process, we train a neural network $\hat{\mathbf{x}}_\theta(\mathbf{x}_t, t)$ to predict the optimally denoised structure by optimizing a bound on the likelihood of the noise-free structure $\mathbf{x}_0$,

$$\log p(\mathbf{x}_0) \geq -\frac{1}{2}\log\det\left(2\pi e\mathbf{R}\mathbf{R}^\intercal\right) - \frac{1}{2}\mathbb{E}_{p(\mathbf{x}_t|\mathbf{x}_0)p(t)}\left[\mathrm{SNR}_t'\left(\frac{N}{1+\mathrm{SNR}_t} - \|\mathbf{R}^{-1}\left(\hat{\mathbf{x}}_\theta(\mathbf{x}_t,t) - \mathbf{x}_0\right)\|_2^2\right)\right],$$

together with auxiliary training objectives which emphasize accurate denoising of specific substructural features (Supplementary Appendix B). Both the likelihood and auxiliary objectives are weighted in terms of various functions of the time dependent Signal-to-Noise ratio, $\mathrm{SNR}_t = \frac{\alpha_t^2}{1-\alpha_t^2}$. We parameterize the optimal denoiser $\hat{\mathbf{x}}_\theta(\mathbf{x}_t, t)$ in terms of a graph neural network with random long-range connectivity for global context (Supplementary Appendix E) which predicts denoised structures via a weighted consensus of inter-residue geometry predictions (Supplementary Appendix F).

Sampling backbones To sample backbones, we simulate a non-equilibrium reverse diffusion process enriched with equilibrating Langevin dynamics (Supplementary Appendix C) by integrating the stochastic differential equation

$$\mathrm{d}\mathbf{x} = -\frac{1}{2}\beta_t\mathbf{x} - \left(\lambda_t + \frac{\lambda_0\psi}{2}\right)\beta_t\mathbf{R}\mathbf{R}^\intercal\nabla_\mathbf{x}\log p_t\left(\mathbf{x};\theta\right)\mathrm{d}t + \sqrt{\beta_t(1+\psi)}\mathbf{R}\,\mathrm{d}\bar{\mathbf{w}},$$

[2]We describe more generalized noising schedules in Supplementary Appendix B.

where λ_t are λ_0 inverse are temperature parameters, ψ sets the rate of Langevin equilibration per unit time, $d\bar{\mathbf{w}}$ is a reverse-time Wiener process, and $\nabla_{\mathbf{x}} \log p_t(\mathbf{x};\boldsymbol{\theta})$ is the time-dependent score function which can be expressed as an affine transform of the optimal denoiser. These Langevin-enriched dynamics allow us to adjust the time-dependent distribution to account for perturbations such as external conditioning cues or lower sampling temperatures which bias towards high-likelihood states (Supplementary Appendix C).

Sampling side-chains For the design network, we train a graph neural network to predict discrete sequence states via either conditional Potts models or conditional language models and predict side chain conformations via an autoregressive decomposition and an empirical histogram parameterization binned at 10° resolution. Sampling is performed via a combination of penalized Markov Chain Monte Carlo and/or ancestral sampling (Supplementary Appendix I).

Dataset We constructed a dataset of 28,819 protein complex structures from the Protein Data Bank circa March 20, 2022 (Supplementary Appendix H). These complexes were filtered for X-ray crystal structures at resolution ≤ 2.6 *Å* and were then redundancy-reduced via general sequence clustering at 50% identity followed by re-enrichment of 1,726 highly-variable antibody systems with clustering at 10% sequence identity. We split these data into 80%/10%/10% training/validation/test components based on a graph-based annotation overlap reduction procedure.

Training We trained two configurations of the *backbone network* on 8 V100 GPUs for approximately 1.6 and 1.8 million training steps with target batch sizes of approximately 32,000 residues per step, with each model having approximately 19 million parameters (Supplementary Table 2). To test the influence of different components of our framework, we also carried out an ablation study of 7 different model configurations each trained with 8 V100 GPUs and similar batch sizing for approximately 500,000 steps (Supplementary Appendix L, Supplementary Figure 22). Additionally, we trained two configurations of the *design network* on 1 or 8 V100 GPUs with each model having approximately 4 or 14 million parameters, respectively, based on the inclusion of side chain and autoregressive decoding layers.

A.2 Conditioners

To make protein design with Chroma *programmable*, we introduce a *Conditioners* framework that allows for automatic conditional sampling under arbitrary composition of protein specifications. These specifications can come in the forms of restraints, which bias the distribution of states, and constraints, which directly restrict the domain of underlying sampling process (Supplementary Appendix M). We accomplish this by formulating conditional protein design as *sampling under composable transformations* which can affect both the energy and/or the state variables. Briefly, we can express the time-dependent (unnormalized) posterior log-likelihood of structures $\mathbf{x}$ given

conditions $\mathbf{y}$ as

$$\log \tilde{p}(\mathbf{x}_t|y,t) = \underbrace{\log \tilde{p}(\mathbf{x}_t|t)}_{\text{Prior}} + \underbrace{\log p(\mathbf{y}|\mathbf{x}_t,t)}_{\text{Likelihood}}$$
$$= \underbrace{\frac{1}{2}\left\|\sigma_t^{-1}\mathbf{R}^{-1}\left(f(\tilde{\mathbf{x}}_t,U_0;t) - \alpha_t\hat{\mathbf{x}}_t\left(f(\tilde{\mathbf{x}}_t,U_0;t),t\right)\right)\right\|_2^2}_{\text{Diffusion Energy}} + \underbrace{U_f(\tilde{\mathbf{x}}_t,U_0;t)}_{\text{Conditioner Energy}},$$

where $f(\tilde{\mathbf{x}}_t,U_0;t)$ is a function that (optionally) transforms the state and $U_f(\tilde{\mathbf{x}}_t,U_0;t)$ is a function that transforms the energy. These transformation functions are composable and, by leveraging automatic differentiation, we can derive universal conditional samplers from any gradient-based MCMC algorithm such as Langevin dynamics (Supplementary Appendix M.2).

We implement and evaluate several conditioners within this framework capturing a variety of potential protein design criteria including:

- substructure constraints (Supplementary Appendix N)
- substructure distance restraints (Supplementary Appendix O)
- substructure motif restraints (Supplementary Appendix P)
- symmetries across chains (Supplementary Appendix Q)
- arbitrary volumetric shapes (Supplementary Appendix R)
- neural network classifiers (Supplementary Appendix S)
- natural language prompts (Supplementary Appendix T)

A.3 Wet lab experiments

We experimentally analyzed 310 proteins generated by Chroma (Fig. 5A, Supplementary Figure 36, Supplementary Figure 37A, Supplementary Figure 40A) using a split-GFP pooled solubility assay in *E. coli* (Supplementary Figure 38, Supplementary Table 8), quantitating protein solubility scores by Nanopore sequencing-based enrichment analysis after fluorescence-activated cell sorting (Fig. 5B, Supplementary Figure 37B, Supplementary Figure 40B-C) and performing additional assay corroboration by western blot (Supplementary Figure 39, Supplementary Figure 40D). We analyzed purified Chroma proteins by differential scanning calorimetry (Supplementary Figure 37C, Supplementary Figure 41, Extended Data Table 1) and circular dichroism (Fig. 5E-F) to analyze stability and secondary structure components, respectively. We structurally validated two unconditional proteins by X-ray crystallography (Fig. 5C-D, Extended Data Table 2). All experimental details can be found in the Experimental Validation section (Supplementary Appendix U).

B Diffusion Models with Structured Correlations

B.1 Correlated diffusion as uncorrelated diffusion in a transformed space

Correlation and diffusion Most natural data possess a hierarchy of correlation structures, some of which are very simple (e.g., most nearby pixels in natural images will tend to be a similar color) and some of which are very subtle (e.g., complex constraints govern the set of pixels forming an eye or a cat). With finite computing resources and modeling power, it can be advantageous to design learning systems that capture simple correlations as efficiently as possible such that most model capacity can be dedicated to nontrivial dependencies (see Appendix D).

Diffusion models capture complex constraints in the data by learning to reverse a diffusion process that transforms data into noise [54, 55]. While most of these original diffusion frameworks considered the possibility of correlated noise, it is typical in contemporary models to use isotropic noise that is standard normally distributed. In this configuration, models must learn both simple correlations and complex correlations in data *from scratch*.

Whitening transformations and linear generative models One classical approach for removing nuisance correlations in the data is to apply a "whitening transformation", i.e., an affine linear transformation $\mathbf{z} = \Sigma^{-\frac{1}{2}}(\mathbf{x} - \boldsymbol{\mu})$ that decorrelates all factors of variation by subtracting the empirical mean $\boldsymbol{\mu}$ and multiplying by a square root of the inverse covariance matrix $\mathbf{R} = \Sigma^{-\frac{1}{2}}$.

Whitening data can also be related to fitting the data to a Gaussian model $\mathbf{x} = F(\mathbf{z}) = \mathbf{R}\mathbf{z} + \mathbf{b}$ where the whitened factors $\mathbf{z}$ are standard normally distributed as $\mathbf{z} \sim \mathcal{N}(\mathbf{0}, \mathbf{I})$ [56]. The density in the whitened space can be related to the density in the transformed space by the change of variables formula as

$$\begin{aligned}
\log p(\mathbf{x}) &= \log p_{\mathbf{z}}(F^{-1}(\mathbf{x})) - \log \left| \det \frac{\mathrm{d}F}{d\mathbf{x}} \right| \\
&= \log p_{\mathbf{z}}(\mathbf{R}^{-1}(\mathbf{x} - \mathbf{b})) - \log |\det \mathbf{R}| \\
&= \log \mathcal{N}(\mathbf{R}^{-1}(\mathbf{x} - \mathbf{b}); \mathbf{0}, \mathbf{I}) - \log |\det \mathbf{R}| \\
&= \log \mathcal{N}(\mathbf{x}; \mathbf{b}, \mathbf{R}\mathbf{R}^{\top}).
\end{aligned}$$

From uncorrelated diffusion to correlated diffusion If we have a linear Gaussian prior for our data $p(\mathbf{x}) = \mathcal{N}(\mathbf{x}; \mathbf{b}, \mathbf{R}\mathbf{R}^{\top})$ which can be sampled[3] as $\mathbf{x} = \mathbf{R}\mathbf{z}$ with $\mathbf{z} \sim \mathcal{N}(\mathbf{0}, \mathbf{I})$, then an *uncorrelated* diffusion process on the whitened coordinates $\mathbf{z}_t \sim p_t(\mathbf{z}|\mathbf{z}_0)$ will induce a *correlated* diffusion process on the original coordinates $\mathbf{x}_t \sim p_t(\mathbf{x}|\mathbf{x}_0)$. For the typical case of a Gaussian diffusion process with time-dependent noise, the uncorrelated diffusion

$$p(\mathbf{z}_t|\mathbf{z}_0) = \mathcal{N}(\mathbf{z}; \alpha_t \mathbf{z}_0, \sigma_t^2),$$

with time-dependent signal scaling α_t and noise scaling σ_t, will induce correlated Gaussian noise

$$p(\mathbf{x}_t|\mathbf{x}_0) = \mathcal{N}(\mathbf{x}; \alpha_t \mathbf{x}_0, \sigma_t^2 \mathbf{R}\mathbf{R}^{\top}).$$

[3] We assume the data are centered (have zero mean) for ease of notation.

We can also see this relationship infinitesimally in time [55]. If we express the uncorrelated diffusion process in terms of a stochastic differential equation (SDE),

$$\mathrm{d}\mathbf{z} = h_t \mathbf{z}\,\mathrm{d}t + g_t\,\mathrm{d}\mathbf{w},$$

where h_t is a time-dependent drift coefficient, and g_t is a time diffusion coefficient, and $\mathbf{w}$ is a standard Wiener process, then we can also express the express an SDE for the correlated process on $\mathbf{x}_t$ by substituting[4] $\mathbf{x} = \mathbf{R}\mathbf{z}$ as

$$\begin{aligned} \mathrm{d}\mathbf{x} &= \mathbf{R}\mathrm{d}\mathbf{z} \\ &= h_t \mathbf{R}\mathbf{z}\,\mathrm{d}t + g_t \mathbf{R}\,\mathrm{d}\mathbf{w} \\ &= h_t \mathbf{x}\,\mathrm{d}t + g_t \mathbf{R}\,\mathrm{d}\mathbf{w}. \end{aligned}$$

In the next section we explain to how to relate the noise kernel parameters (α_t, σ_t) to the instantaneous parameters (h_t, g_t).

B.2 Scheduling diffusion to balance signal and noise

Two-parameter noising schedules The dynamics of the noise kernel parameters (α_t, σ_t) define a generalized Gaussian noising process that is used across both diffusion modeling [55, 57] and flow-matching [58–60]. Some well-known noise parameterizations in this family include the variance-exploding and variance-preserving processes [55] and the optimal transport (OT) flow process [58–60]. For all of these processes, the corresponding forwards SDE, reverse SDE, and flow ODE (described subsequently) also exist and can be parameterized in terms of parameters (h_t, g_t) as a function of the noise kernel parameters (α_t, σ_t) via the relationship

$$h_t = \frac{\alpha_t'}{\alpha_t}, \quad g_t = \sqrt{2\sigma_t^2 \left(\frac{\sigma_t'}{\sigma_t} - \frac{\alpha_t'}{\alpha_t}\right)},$$

which may also be expressed as

$$h_t = \frac{\mathrm{d}\log\alpha_t}{\mathrm{d}t}, \quad g_t = \sqrt{-\sigma_t^2 \frac{\mathrm{d}\log \mathrm{SNR}_t}{\mathrm{d}t}},$$

where $\mathrm{SNR}_t = \frac{\alpha_t^2}{\sigma_t^2}$. We derive this below.

Linking instantaneous and integrated parameters The above results may be seen via ODEs which govern the time-evolution of the means and variances of the stochastic process. Following [61], for an SDE of the form

$$\mathrm{d}\mathbf{x} = f(\mathbf{x}_t, t)\,\mathrm{d}t + \mathbf{G}(\mathbf{x}, t)\,\mathrm{d}\mathbf{w},$$

where $f(\mathbf{x}_t, t)$ and $\mathbf{G}(\mathbf{x}, t)$ are the drift and diffusion terms, we can express the time-dependent mean and covariance as

$$\begin{aligned} \frac{\mathrm{d}}{\mathrm{d}t}\boldsymbol{\mu}_t &= \mathbb{E}\left[f(\mathbf{x}_t, t)\right] \\ \frac{\mathrm{d}}{\mathrm{d}t}\boldsymbol{\Sigma}_t &= \mathbb{E}\left[f(\mathbf{x}_t, t)(\mathbf{x}_t - \boldsymbol{\mu}_t)^\intercal\right] + \mathbb{E}\left[(\mathbf{x}_t - \boldsymbol{\mu}_t) f(\mathbf{x}_t, t)^\intercal\right] + \mathbb{E}\left[\mathbf{G}(\mathbf{x}_t, t)\mathbf{G}^\intercal(\mathbf{x}_t, t)\right]. \end{aligned}$$

[4] This can be justified by Ito's lemma.

In our framework, the noise kernel is $\mathcal{N}(\alpha_t \mathbf{x}_0, \sigma_t^2 \Sigma)$ and we seek an OU process of with the drift term $f(\mathbf{x}_t, t) = h_t \mathbf{x}_t$, and the correlated diffusion term $\mathbf{G}_t = g_t \mathbf{R}$. If we define $\mathbf{\Sigma} = \mathbf{R}\mathbf{R}^\intercal$, and $\Sigma_t = \sigma_t^2 \Sigma$, we have

$$
\begin{aligned}
\frac{\mathrm{d}}{\mathrm{d}t}\mathbf{\Sigma}_t &= \mathbb{E}\left[h_t \mathbf{x}_t (\mathbf{x}_t - \boldsymbol{\mu}_t)^\intercal\right] + \mathbb{E}\left[-(\mathbf{x}_t - \boldsymbol{\mu}_t) h_t \mathbf{x}_t^\intercal\right] + \mathbb{E}\left[\mathbf{G}(\mathbf{x}_t, t)\mathbf{G}^\intercal(\mathbf{x}_t, t)\right] \\
&= 2h_t \ \mathbb{E}\left[\mathbf{x}_t \mathbf{x}_t^\intercal - \boldsymbol{\mu}_t \boldsymbol{\mu}_t^\intercal\right] + g_t^2 \mathbf{\Sigma} \\
&= 2h_t \ \Sigma_t + g_t^2 \mathbf{\Sigma} \\
&\implies g_t^2 \mathbf{\Sigma} = \frac{\mathrm{d}}{\mathrm{d}t}\mathbf{\Sigma}_t - 2h_t \ \Sigma_t,
\end{aligned}
$$

where the last step comes from the identity

$$
\mathbf{\Sigma_t} = \mathbb{E}[(\mathbf{x}_t - \mu_t)(\mathbf{x}_t - \mu_t)^\intercal] = \mathbb{E}[\mathbf{x}_t \mathbf{x}_t^\intercal - \mathbf{x}_t \mu_t^\intercal - \mu_t \mathbf{x}_t^\intercal + \mu_t \mu_t^\intercal] = \mathbb{E}[\mathbf{x}_t \mathbf{x}_t^\intercal] - \mu_t \mu_t^\intercal.
$$

Now substituting our noise kernel $p(\mathbf{x}_t|\mathbf{x}_0) = \mathcal{N}(\alpha_t \mathbf{x}_0, \sigma_t^2 \Sigma)$, we have h_t in terms of α_t as

$$
\begin{aligned}
\frac{\mathrm{d}}{\mathrm{d}t}\boldsymbol{\mu}_t &= \mathbb{E}\left[f(\mathbf{x}_t, t)\right] = h_t \mathbb{E}\left[\mathbf{x}_t\right] = h_t \mu_t = \alpha_t' \mathbf{x}_0 \\
&\implies h_t = \frac{\alpha_t'}{\alpha_t} = \frac{\mathrm{d}\log \alpha_t}{\mathrm{d}t},
\end{aligned}
$$

and g_t in terms of σ_t as

$$
\begin{aligned}
g_t^2 \mathbf{\Sigma} &= \mathbf{\Sigma}_t' - 2h_t \ \mathbf{\Sigma}_t = \mathbf{\Sigma}_t' - \frac{2\alpha_t'}{\alpha_t} \ \mathbf{\Sigma}_t = 2\sigma_t \sigma_t' \mathbf{\Sigma} - \frac{2\alpha_\mathbf{t}'}{\alpha_\mathbf{t}} \sigma_t^2 \mathbf{\Sigma} \\
&\implies g_t = \sqrt{2\sigma_t^2 \left(\frac{\sigma_t'}{\sigma_t} - \frac{\alpha_t'}{\alpha_t}\right)} = \sqrt{-\sigma_t^2 \frac{\mathrm{d}\log \mathrm{SNR}_t}{\mathrm{d}t}}.
\end{aligned}
$$

This establishes the correspondence between the time evolution of the noise kernel and the underlying diffusion SDE for a general Gaussian noising schedule.

Variance Preserving schedules Throughout this work, we focus on noise that is distributionally matched and use the variance-preserving process [55] which enforces a balance between the creation of noise and destruction of signal as $\sigma_t^2 = 1 - \alpha_t^2$. Setting $h_t = \frac{\alpha_t'}{\alpha_t} \triangleq -\frac{1}{2}\beta_t$, we have

$$
g_t = \sqrt{2\sigma_t^2 \left(\frac{\sigma_t'}{\sigma_t} - \frac{\alpha_t'}{\alpha_t}\right)} = \sqrt{-2(\alpha_t^2 + \sigma_t^2)\frac{\alpha_t'}{\alpha_t}} = \sqrt{\beta_t},
$$

which gives the well-known forwards SDE

$$
\mathrm{d}\mathbf{x} = -\frac{1}{2}\beta_t \mathbf{x}\,\mathrm{d}t + \sqrt{\beta_t}\mathbf{R}\,\mathrm{d}\mathbf{w}.
$$

Optimal Transport schedules The Optimal Transport (OT) schedule proposed in [58] is another type of one-parameter noising process that interpolates between a prior $p(\mathbf{x}_1)$ and the data distribution $p(\mathbf{x}_0)$. It induces 'straight paths' in the corresponding flow ODE. Given the noising parameters $\sigma_t = t$ and $\alpha_t = 1-t$, we can convert the flow ODE to an SDE,

$$\mathrm{d}\mathbf{x}_t = -\frac{1}{1-t}\mathbf{x}_t\,\mathrm{d}t + \sqrt{\frac{2t}{1-t}}\mathbf{R}\,\mathrm{d}\mathbf{w}_t,$$

where $h_t = -\frac{1}{1-t}$ and $g_t = \sqrt{\frac{2t}{1-t}}$. The SDE trajectories samples $p(\mathbf{x}_t|\mathbf{x}_0) = \mathcal{N}(\alpha_t\mathbf{x}_0, \sigma_t^2\Sigma)$. In practice, following [58], one can integrate $0 < t < 1-\varepsilon$ to ensure numerical stability, where ε is a small number.

Schedule considerations With a family of parameterized schedules chosen, it is still important to determine how to set the time scaling of noise addition to the time $t \in (0,1)$. Throughout this work, we used the variance preserving process with the log-linear SNR schedule proposed in [62], which is favorable for likelihood weighted objectives such as ELBO. Many other possible β_t have been explored in the literature, such as the linear and cosine schedule [63], which can be used to implicitly weigh what noise levels receive the most weight during training time and get the most resolution during integration. With that said, it is important to note that importance weighting across time and varying-timestep SDE and ODE solvers mean that all of these choices are largely for convenience of implementation and variance reduction. In the most general case, one could parameterize arbitrary two parameter schedules of α_t and σ_t as monotonic functions satisfying the boundary conditions $(\sigma_0 = 0, \alpha_0 = 1)$ and $(\sigma_1 = 1, \alpha_1 = 0)$.

B.3 Training with likelihood on an Evidence Lower Bound (ELBO)

Denoising loss Diffusion models can be parameterized in terms of a denoising neural network $\hat{\mathbf{x}}_{\boldsymbol{\theta}}(\mathbf{x},t)$ that is trained to predict $\mathbf{x}_0$ given a noisy sample $\mathbf{x}_t$. Typically this is done by minimizing a *denoising loss*

$$\mathcal{L}(\mathbf{x}_0;\boldsymbol{\theta}) = \mathbb{E}_{\mathbf{x}_t\sim p(\mathbf{x}_t|\mathbf{x}_0),t\sim\mathrm{Unif}(0,1)}\left[\tau_t\|\hat{\mathbf{x}}_{\boldsymbol{\theta}}(\mathbf{x}_t,t)-\mathbf{x}_0\|_2^2\right]$$

where τ_t is a time-dependent weighting to emphasize the loss at particular points in time (noise levels) [55]. Training with this loss can be directly related to *score matching* and *noise prediction* which can be cast as alternative parameterizations of the target output of the network [62].

ELBO We train our diffusion models by optimizing a bound on the log marginal likelihood of data together with optional auxiliary losses. As shown in Information-Theoretic Diffusion models [64] and building on Variational Diffusion models [62], we can express a lower bound on the the log-likelihood of data in terms of the weighted average of mean-square error across diffusion time

as

$$\begin{aligned}
\log p(\mathbf{z}_0) &= -\frac{N}{2}\log(2\pi e) + \frac{1}{2}\int_0^\infty \left(\frac{N}{1+\mathrm{SNR}} - \mathrm{mmse}(\mathbf{z}_0, \mathrm{SNR})\right) \mathrm{dSNR} \\
&= -\frac{N}{2}\log(2\pi e) - \frac{1}{2}\int_0^1 \left(\frac{N}{1+\mathrm{SNR}_t} - \mathrm{mmse}(\mathbf{z}_0, \mathrm{SNR}_t)\right) \mathrm{SNR}'_t \,\mathrm{d}t \\
&\geq -\frac{N}{2}\log(2\pi e) - \frac{1}{2}\int_0^1 \left(\frac{N}{1+\mathrm{SNR}_t} - \mathbb{E}_{p(\mathbf{z}_t|\mathbf{z}_0)}\left[\|\hat{\mathbf{z}}(\mathbf{z}_t,t) - \mathbf{z}_0\|_2^2\right]\right) \mathrm{SNR}'_t \,\mathrm{d}t,
\end{aligned}$$

where N is the dimensionality of $\mathbf{z}_0$, the Signal-to-Noise Ratio (SNR) is defined $\mathrm{SNR}_t = \frac{\alpha_t^2}{\sigma_t^2}$ with $\sigma_t^2 = 1 - \alpha_t^2$ for VP diffusions, and mmse is the minimum achievable mean square error under the forwards noising model as a function of the SNR. We can then apply the change of variables formula to transform this bound as

$$\begin{aligned}
\log p(\mathbf{x}_0) &= \log p(\mathbf{z}_0) - \log\det\mathbf{R} \\
&\geq -\frac{N}{2}\log(2\pi e) - \log\det\mathbf{R} - \frac{1}{2}\int_0^1 \left(\frac{N}{1+\mathrm{SNR}_t} - \mathbb{E}_{p(\mathbf{x}_t|\mathbf{x}_0)}\left[\|\mathbf{R}^{-1}(\hat{\mathbf{x}}(\mathbf{x}_t,t) - \mathbf{x}_0)\|_2^2\right]\right) \mathrm{SNR}'_t \,\mathrm{d}t \\
&= \underbrace{-\frac{1}{2}\log\det(2\pi e\mathbf{R}\mathbf{R}^\intercal)}_{\text{Entropy of the Gaussian prior}} \underbrace{- \frac{1}{2}\mathbb{E}_{p(\mathbf{x}_t|\mathbf{x}_0)p(t)}\left[\mathrm{SNR}'_t\left(\frac{N}{1+\mathrm{SNR}_t} - \|\mathbf{R}^{-1}(\hat{\mathbf{x}}(\mathbf{x}_t,t) - \mathbf{x}_0)\|_2^2\right)\right]}_{\text{Deviation from Gaussianity (Bound)}} \\
&\triangleq \mathcal{L}(\mathbf{x};\boldsymbol{\theta}),
\end{aligned}$$

where $p(t)$ is uniformly distributed on $(0,1)$.

It is important to note that, for continuous data, probability density and information content is unbounded and can become pathologically high (e.g., with infinite precision one could encode the entire Protein Data Bank in the decimal expansion of a single coordinate). In practice we may handle this by manipulating the noise schedule to bound the maximum attainable SNR_t [64].

B.4 Auxiliary training objectives

There has been a consistent tension in the diffusion modeling literature between training on likelihood-based objectives, likelihood-related objectives, and auxiliary domain-specific objectives [55, 65]. Here we consider a few objectives in the latter category. Generally, diffusion models can be equivalently treated in the frameworks of *score matching*, *noise prediction*, *denoising*, which all can be considered as different parameterizations of the problem of learning a posterior-optimal denoiser which minimizes mean square denoising error across time.

ELBO-weighted unwhitened MSE While the information content of the structures is measured by a SNR-weighted average of mean square error in *whitened* space, we also consider similarly-weighted objective measuring errors in $\mathbf{x}$-space as

$$\mathcal{L}_{\mathbf{x}}(\mathbf{x}_0;\boldsymbol{\theta}) = -\mathbb{E}_{\mathbf{x}_t\sim p(\mathbf{x}_t|\mathbf{x}_0), t\sim\mathrm{Unif}(0,1)}\left[\omega^{-2}\mathrm{SNR}'_t\,\|\hat{\mathbf{x}}_{\boldsymbol{\theta}}(\mathbf{x}_t,t) - \mathbf{x}\|_2^2\right]. \tag{1}$$

where we set the scale factor ω to give $\mathbf{x}$ units of nanometers. We found this regularization to be important because in practice we care about absolute errors in $\mathbf{x}$ space, i.e. absolute spatial errors,

at least as much as we care about errors in $\mathbf{z}$ space, which will correspond under our covariance models (Supplementary Appendix D) to relative local geometries. These objectives share the same minima, i.e. they will be minimized by the posterior optimal denoiser under the diffusion process, but for an approximately trained parametric model with limited capacity will trade off different errors in which statistics of data are emphasized in reconstruction.

Substructure MSE and Perceptually-motivated metrics As has often been emphasized in the literature in generative models of images, not all bits are equally important to perception or, more generally, sample utility. For example, it takes the same number of bits to encode the *average color of an image* as it does to encode the *color of one single pixel*, but mis-estimation of the average color will generally be much more noticeable to humans.

As a result of this, many diffusion models eschew training purely on likelihood-based metrics, for example using flat weightings of the denoising loss across diffusion time which implicitly emphasize the importance of low-frequency statistics [55]. Other generative models have used domain-specific metrics such as FAPE for proteins [66] as the denoising objective for diffusion training [67].

Here we consider auxiliary training objectives for protein backbone diffusion models which emphasize some conventionally important aspects of structural similarity. Since diffusion models trained to optimality will learn the *posterior mean denoising function*, which minimizes *mean squared error* of reconstruction from the forward process, we consider only squared-error objectives.

Substructure Aligned Squared Error Minimizing ELBO-weighted mean squared error trains a diffusion model to learn all statistics of the data at all length scales, but for proteins we know that there are some substructural statistics which may be stronger and more important to correctly estimate than others. For example, proteins often exhibit substructures, such as secondary structural elements or domains connected by more flexible linkers. We can encourage the denoiser to prioritize these substructural statistics of the data by optimizing the mean squared error *under optimal superposition* as

$$\mathcal{D}_{\text{substructure}}(\mathbf{x},\mathbf{x}') = \sum_{\mathcal{M}_i \in \{\mathcal{M}_i\}} \min_{\mathbf{T}\in \mathrm{SE}(3)} \|\mathbf{x}^{\mathcal{M}_i} - \mathbf{T}\circ \mathbf{x}'^{\mathcal{M}_i}\|_2^2,$$

where $\{\mathcal{M}_i\}$ is a set of substructures and the inner optimization problem can solved via the optimal superposition with a Kabsch or quaternion-based method [68, 69]. We consider the following substructures for measuring aligned squared error:

- **Global structure**. $\mathcal{M} = [\![1, N]\!]$. In this case, the substructure aligned MSE will simply be a rescaling of the squared optimal RMSD after superposition.
- **Fragment structure**. $\mathcal{M}_i = \{i-m, \dots, i+m\}$. We consider fragments of radius $m = 7$ residues centered around each residue i.

Distance Squared Error Many aspects of protein geometry are driven by specific packing and steric interactions that depend more strongly on interatomic distances and less strongly on relative

orientations. We consider a loss measuring the squared error of proteins when represented by distance matrices of their C_α carbon atoms as

$$\mathcal{D}_{\text{distance}}(\mathbf{x},\mathbf{x}') = \sum_{ij}(D^{\text{CA}}_{ij}(\mathbf{x}) - D^{\text{CA}}_{ij}(\mathbf{x}'))^2.$$

Normalizing Auxiliary Losses Across Time and Schedules All of the aforementioned losses can be used as denoising losses by minimizing $\mathbb{E}_{p(\mathbf{x}_0,\mathbf{x}_t,t)}\left[\mathcal{D}(\hat{\mathbf{x}}(\mathbf{x}_t,t),\mathbf{x}_0)\right]$, but (i) an unweighted average will be dominated by loss values at high t and (ii) values of these losses will be incomparable if the noise schedule of the diffusion is changed, complicating evaluation. To address both of these issues, we propose (i) to normalize the losses with an approximate estimate of the time-dependent error magnitude and (ii) to reweight the average with respect to time t as an average with respect to a schedule-invariant statisic via importance weights.

One intuitive schedule-invariant statistic is the **S**ignal to **S**ignal plus **N**oise **R**atio $\text{SSNR}_t \triangleq \frac{\alpha_t^2}{\alpha_t^2+\sigma_t^2} = \frac{\text{SNR}_t}{\text{SNR}_t+1}$. For Variance-Preserving diffusion, this value simplifies to $\text{SSNR}_t = \alpha_t^2 \in [0,1]$. Since t is uniformly distributed on $(0,1)$ and SSNR_t goes from 1 to 0, we can interpret SSNR_{1-t} as a CDF and compute $p(\text{SSNR}_t) = \frac{\mathrm{d}}{\mathrm{d}t}\text{SSNR}_t^{-1}(\text{SSNR}_t)$. We can then compute importance weights as $\frac{1}{p(\text{SSNR}_t)}$ and combine that with normalization to yield normalized denoising training losses as

$$\mathcal{L}_{\mathcal{D}}(\mathbf{x}_0;\boldsymbol{\theta}) = \mathbb{E}_{\mathbf{x}_t,\mathbf{x}'_t\sim p(\mathbf{x}_t|\mathbf{x}_0),t\sim\text{Unif}(0,1)}\left[\frac{1}{p(\text{SSNR}_t)}\frac{\mathcal{D}(\hat{\mathbf{x}}(\mathbf{x}_t,t);\mathbf{x}_0)}{\mathcal{D}(\mathbf{x}'_t;\mathbf{x}_0)}\right].$$

Transform Squared Error Our proposed method for parameterizing predicted structure in terms of predicted inter-residue geometries (Supplementary Appendix F) leverages predicted inter-residue transforms $\mathbf{T}_{ij}$ between every pair of residues on the graph. When training on ELBO, these predicted inter-residue transforms are only indirectly supervised by backpropagation but we can also directly supervise their values towards the true denoised inter-residue geometries to potentially stabilize and accelerate learning. This is not dissimilar from auxiliary prediction of inter-residue distances as done in end-to-end structure prediction methods such as AlphaFold [66]. Training these quantities directly can be useful because (i) they are SE(3) invariant and typically lower-variance targets than raw coordinates and (ii) they are *aligned* with the overall denoising objective in the sense that perfect inter-residue geometry prediction will yield a perfectly denoised structure (assuming sufficient equilibration time of the backbone solver).

We score the agreement between the predicted $\hat{\mathbf{T}}^{\theta}_{ij}(\mathbf{x}_t)$ and actual $\mathbf{T}_{ij}(\mathbf{x}_0)$ inter-residue geometries as the sum of squared errors in the predicted translation vectors and rotation matrices, i.e.

$$\mathcal{L}_{\text{transform}}(\mathbf{x}_0;\boldsymbol{\theta}) = \sum_{ij\in\mathcal{G}(\mathbf{x})}\left\|\mathbf{t}_{ij}(\mathbf{x}_0) - \hat{\mathbf{t}}^{\theta}_{ij}(\mathbf{x}_t)\right\|_2^2 + \left\|\mathbf{R}_{ij}(\mathbf{x}_0) - \hat{\mathbf{R}}^{\theta}_{ij}(\mathbf{x}_t)\right\|_2^2,$$

where the translational disagreement is scaled to give it units of nanometers.

B.5 Sampling with the Reverse-time SDE

In whitened space, we can express the *reverse-time* dynamics for the forwards-time SDE in terms of another SDE [55, 70] that depends on the *score function* of the time-dependent marginals $\nabla_{\mathbf{z}} \log p_t(\mathbf{z})$ as

$$\mathrm{d}\mathbf{z} = h_t \, \mathbf{z} - g_t^2 \, \nabla_{\mathbf{z}} \log p_t(\mathbf{z}) \, \mathrm{d}t + g_t \, \mathrm{d}\bar{\mathbf{w}}.$$

We can similarly express this in the score function of the transformed coordinate system as

$$\mathrm{d}\mathbf{x} = h_t \, \mathbf{x} - g_t^2 \, \mathbf{R}\mathbf{R}^\intercal \nabla_{\mathbf{x}} \log p_t(\mathbf{x}) \, \mathrm{d}t + g_t \, \mathbf{R} \, \mathrm{d}\bar{\mathbf{w}}.$$

For the variance-preserving process, the reverse-time SDE reduces to

$$\mathrm{d}\mathbf{x} = \left(-\frac{1}{2} \, \mathbf{x} - \, \mathbf{R}\mathbf{R}^\intercal \nabla_{\mathbf{x}} \log p_t(\mathbf{x}) \right) \beta_t \, \mathrm{d}t + \sqrt{\beta_t} \, \mathbf{R} \, \mathrm{d}\bar{\mathbf{w}}.$$

We can sample from the diffusion model by sampling the "prior" ($t = 1$ distribution) and then integrating the Reverse-time SDE backwards from $t = 1$ to $t = 0$. We can rewrite the above SDE in directly in terms of our optimal denoising network $\hat{\mathbf{x}}_{\boldsymbol{\theta}}(\mathbf{x}, t)$ (trained as described above) by leveraging the relationship [55, 62] that

$$\nabla_{\mathbf{x}} \log p_t(\mathbf{x}) = \left(\sigma_t^2 \mathbf{R}\mathbf{R}^\intercal \right)^{-1} \left(\alpha_t \hat{\mathbf{x}}_{\boldsymbol{\theta}}(\mathbf{x}, t) - \mathbf{x} \right).$$

This yields the reverse-time SDE for VP diffusions in terms of the optimal denoising network $\hat{\mathbf{x}}_{\boldsymbol{\theta}}(\mathbf{x}, t)$ as

$$\begin{aligned}
\mathrm{d}\mathbf{x} &= \left(-\frac{1}{2} \, \mathbf{x} - \mathbf{R}\mathbf{R}^\intercal \frac{\left(\mathbf{R}\mathbf{R}^\intercal\right)^{-1}}{1 - \alpha_t^2} \left(\alpha_t \hat{\mathbf{x}}_{\boldsymbol{\theta}}(\mathbf{x}, t) - \mathbf{x} \right) \right) \beta_t \, \mathrm{d}t + \sqrt{\beta_t} \, \mathbf{R} \, \mathrm{d}\bar{\mathbf{w}} \\
&= \left(-\frac{1}{2} \, \mathbf{x} - \frac{\alpha_t \hat{\mathbf{x}}_{\boldsymbol{\theta}}(\mathbf{x}, t) - \mathbf{x}}{1 - \alpha_t^2} \right) \beta_t \, \mathrm{d}t + \sqrt{\beta_t} \, \mathbf{R} \, \mathrm{d}\bar{\mathbf{w}} \\
&= \left(\frac{-\alpha_t \hat{\mathbf{x}}_{\boldsymbol{\theta}}(\mathbf{x}, t) + \mathbf{x} - \frac{1}{2} \, \mathbf{x}(1 - \alpha_t^2)}{1 - \alpha_t^2} \right) \beta_t \, \mathrm{d}t + \sqrt{\beta_t} \, \mathbf{R} \, \mathrm{d}\bar{\mathbf{w}} \\
&= \left(\frac{\alpha_t + 1}{2(1 - \alpha_t^2)} \mathbf{x} - \frac{\alpha_t}{1 - \alpha_t^2} \hat{\mathbf{x}}_{\boldsymbol{\theta}}(\mathbf{x}, t) \right) \beta_t \, \mathrm{d}t + \sqrt{\beta_t} \, \mathbf{R} \, \mathrm{d}\bar{\mathbf{w}}.
\end{aligned}$$

B.6 Sampling with the Probability Flow ODE

Probability Flow ODE for deterministic encoding and sampling Remarkably, it is also possible to derive a set of deterministic *ordinary differential equations* (ODEs) whose marginal evolution from the prior is identical to the above SDEs [55, 71]. In the context of our covariance model, this can be expressed either in terms of the score function $\nabla_{\mathbf{x}} \log p_t(\mathbf{x})$ as

$$\frac{\mathrm{d}\mathbf{x}}{\mathrm{d}t} = h_t \mathbf{x} + \frac{1}{2} g_t^2 \mathbf{R}\mathbf{R}^\intercal \nabla_{\mathbf{x}} \log p_t(\mathbf{x}).$$

For the variance-preserving process, this reduces to

$$\frac{\mathrm{d}\mathbf{x}}{\mathrm{d}t} = -\frac{\beta_t}{2}\left(\mathbf{x} + \mathbf{R}\mathbf{R}^\intercal \nabla_{\mathbf{x}} \log p_t(\mathbf{x})\right).$$

We can alternatively express it in terms of the optimal denoiser network $\hat{\mathbf{x}}_{\boldsymbol{\theta}}(\mathbf{x},t)$ as

$$\begin{aligned}
\frac{\mathrm{d}\mathbf{x}}{\mathrm{d}t} &= -\frac{\beta_t}{2}\left(\mathbf{x} + \mathbf{R}\mathbf{R}^\intercal \left((1-\alpha_t^2)\mathbf{R}\mathbf{R}^\intercal\right)^{-1}\left(\alpha_t \hat{\mathbf{x}}_{\boldsymbol{\theta}}(\mathbf{x},t) - \mathbf{x}\right)\right) \\
&= -\frac{\beta_t}{2}\left(\mathbf{x} + (1-\alpha_t^2)^{-1}\left(\alpha_t \hat{\mathbf{x}}_{\boldsymbol{\theta}}(\mathbf{x},t) - \mathbf{x}\right)\right) \\
&= -\frac{\beta_t}{2}\left(\mathbf{x}\left(1 - \frac{1}{1-\alpha_t^2}\right) + \hat{\mathbf{x}}_{\boldsymbol{\theta}}(\mathbf{x},t)\frac{\alpha_t}{1-\alpha_t^2}\right) \\
&= \frac{\beta_t}{2}\left(\mathbf{x}\frac{\alpha_t}{1-\alpha_t^2} - \hat{\mathbf{x}}_{\boldsymbol{\theta}}(\mathbf{x},t)\frac{\alpha_t}{1-\alpha_t^2}\right) \\
&= \frac{1}{2}\frac{\alpha_t\beta_t}{1-\alpha_t^2}\left(\mathbf{x} - \frac{\hat{\mathbf{x}}_{\boldsymbol{\theta}}(\mathbf{x}_t,t)}{\alpha_t}\right).
\end{aligned}$$

The ODE formulation of sampling is especially important because it enables reformulating the model as a Continuous Normalizing Flow [72, 73], which can admit efficient and exact likelihood calculations using the adjoint method [73].

B.7 Conditional sampling from the posterior under auxiliary constraints

Bayesian posterior SDE for conditional sampling An extremely powerful aspect of the reverse diffusion formulation is that it can also be extended to enable conditional sampling from a Bayesian posterior $p(\mathbf{x}|\mathbf{y})$ by combining with auxiliary classifiers $\log p_t(\mathbf{y}|\mathbf{x})$ and without re-training the base diffusion model [55]. When extended to the correlated diffusion case, this gives the SDE

$$\mathrm{d}\mathbf{x} = h_t\,\mathbf{x} - g_t^2\,\mathbf{R}\mathbf{R}^\intercal\left(\nabla_{\mathbf{x}}\log p_t(\mathbf{x}) + \nabla_{\mathbf{x}}\log p_t(\mathbf{y}|\mathbf{x})\right)\,\mathrm{d}t + g_t\,\mathbf{R}\,\mathrm{d}\bar{\mathbf{w}}.$$

For the variance-preserving process, the SDE reduces to

$$\begin{aligned}
\mathrm{d}\mathbf{x} &= \left(-\frac{1}{2}\,\mathbf{x} - \,\mathbf{R}\mathbf{R}^\intercal\left(\nabla_{\mathbf{x}}\log p_t(\mathbf{x}) + \nabla_{\mathbf{x}}\log p_t(\mathbf{y}|\mathbf{x})\right)\right)\beta_t\,\mathrm{d}t + \sqrt{\beta_t}\,\mathbf{R}\,\mathrm{d}\bar{\mathbf{w}} \\
&= \left(\frac{\alpha_t+1}{2(1-\alpha_t^2)}\mathbf{x} - \frac{\alpha_t}{1-\alpha_t^2}\hat{\mathbf{x}}_{\boldsymbol{\theta}}(\mathbf{x},t) - \,\mathbf{R}\mathbf{R}^\intercal\nabla_{\mathbf{x}}\log p_t(\mathbf{y}|\mathbf{x})\right)\beta_t\,\mathrm{d}t + \sqrt{\beta_t}\,\mathbf{R}\,\mathrm{d}\bar{\mathbf{w}}.
\end{aligned}$$

Bayesian posterior ODE for conditional sampling In the context of our covariance model and conditional constraints, the Probability Flow ODE for sampling from the posterior is

$$\frac{\mathrm{d}\mathbf{x}}{\mathrm{d}t} = h_t\mathbf{x} - \frac{1}{2}g_t^2\mathbf{R}\mathbf{R}^\intercal\left(\nabla_{\mathbf{x}}\log p_t(\mathbf{x}) + \nabla_{\mathbf{x}}\log p_t(\mathbf{y}|\mathbf{x})\right).$$

For the variance-preserving process, the ODE reduces to

$$\begin{aligned}
\frac{\mathrm{d}\mathbf{x}}{\mathrm{d}t} &= -\frac{\beta_t}{2}\left(\mathbf{x} + \mathbf{R}\mathbf{R}^\intercal\left(\nabla_{\mathbf{x}}\log p_t(\mathbf{x}) + \nabla_{\mathbf{x}}\log p_t(\mathbf{y}|\mathbf{x})\right)\right) \\
&= \frac{1}{2}\frac{\alpha_t\beta_t}{1-\alpha_t^2}\left(\mathbf{x} - \frac{\hat{\mathbf{x}}_{\boldsymbol{\theta}}(\mathbf{x},t)}{\alpha_t}\right) - \frac{\beta_t}{2}\,\mathbf{R}\mathbf{R}^\intercal\nabla_{\mathbf{x}}\log p_t(\mathbf{y}|\mathbf{x}).
\end{aligned}$$

B.8 Related work

Subspace diffusion models [74] also consider correlated diffusion, with a particular emphasis on focusing the diffusion on the most relevant factors of variation for statistical and computational efficiency. Additionally, latent-space diffusion models [75] might be viewed as learning a transformed coordinate system in which the diffusion process can more efficiently model the target distribution. Our work provides further evidence for how correlated diffusion may be an underutilized approach to distributional modeling and shows how domain knowledge can be incorporated in the form of simple constraints on the covariance structure of the noise process.

C Low-Temperature Sampling for Diffusion Models

Maximum likelihood training of generative models enforces a tolerable probability of *all* data points and, as a result, misspecified or low-capacity models fit by maximum likelihood will typically be overdispersed. This can be understood through the perspective that maximizing likelihood is equivalent to minimizing the KL divergence from the model to the data distribution, which is the mean-seeking and mode-covering direction of KL divergence.

To mitigate overdispersion in generative models, it is common practice to introduce modified sampling procedures that increase sampling of high-likelihood states (mode emphasis, precision) at the expense of reduced sample diversity (mode coverage, recall). This includes approaches such as shrunken encodings in normalizing flows [76], low-temperature greedy decoding algorithms for language models [77], and stochastic beam search [78].

A powerful but often intractable way to trade diversity for quality in generative models is low-temperature sampling. This involves perturbing a base distribution $p(\mathbf{x})$ by exponentiating with an inverse temperature rescaling factor λ and renormalizing as $p_\lambda(\mathbf{x}) = \frac{1}{Z}p(\mathbf{x})^\lambda$. As the inverse temperature becomes large $\lambda \ll 1$, this perturbed distribution will trade diversity (entropy) for sample quality (likelihood) and eventually collapse into the global optimum as $\lambda \to \infty$. Unfortunately, low temperature sampling in the general case will require expensive iterative sampling methods such as Markov Chain Monte Carlo (MCMC) which typically offer no guarantee of convergence in a practical amount of time [79].

Low temperature and diffusion models The issue of trading diversity for sample quality in diffusion models has been discussed previously, with some authors reporting that simple modifications like upscaling the score function and/or downscaling the noise were ineffective [80]. Instead, classifier guidance and classifier-free guidance have been widely adopted as critical components of contemporary text-to-image diffusion models such as Imagen and DALL-E 2 [81–83].

Equilibrium versus Non-Equilibrium Sampling Here we offer an explanation for why these previous attempts at low-temperature sampling did not work and produce a novel algorithm for low-temperature sampling from diffusion models. We make two key observations, explained in the next two sections

1. **Upscaling the score function of the reverse SDE is insufficient** to properly re-weight populations in a temperature perturbed distribution.

2. **Annealed Langevin dynamics *can* sample from low temperature distributions** if given sufficient equilibration time.

C.1 Reverse-time SDE with temperature annealing

The isotropic Gaussian case To determine how the Reverse SDE can be modified to enable (approximate) low temperature sampling, it is helpful to first consider a case that can be treated exactly: transforming a Gaussian data distribution $\mathcal{N}(\mathbf{x}_0; \boldsymbol{\mu}_{\text{data}}, \sigma^2_{\text{data}})$ to a Gaussian prior $\mathcal{N}(\mathbf{x}_1; 0, \sigma^2_{\text{prior}})$. Under the Variance-Preserving diffusion, the time-dependent marginal density will be given by

$$p_t(\mathbf{x}) = \mathcal{N}\left(\mathbf{x}; \alpha_t \boldsymbol{\mu}_{\text{data}},\ \alpha_t^2 \sigma^2_{\text{data}} + (1-\alpha_t^2)\sigma^2_{\text{prior}}\right),$$

which means that the score function $\mathbf{s}_t$ will be

$$\begin{aligned}\mathbf{s}_t &\triangleq \nabla_{\mathbf{x}} \log p_t(\mathbf{x}) \\ &= \frac{\alpha_t \boldsymbol{\mu}_{\text{data}} - \mathbf{x}}{\alpha_t^2 \sigma^2_{\text{data}} + (1-\alpha_t^2)\sigma^2_{\text{prior}}}.\end{aligned}$$

Now, suppose we wish to *modify* the definition of the time-dependent score function so that, instead of transitioning to the original data distribution, it transforms to the perturbed data distribution, i.e. so the it transitions to $\frac{1}{Z_{lambda_0}} p_0(\mathbf{x})^{\lambda_0}$. For a Gaussian, this operation will simply multiply the precision (or equivalently, divide the covariance) by the factor λ_0. The perturbed score function will therefore be

$$\mathbf{s}_t^{\text{perturb}} = \frac{\alpha_t \boldsymbol{\mu}_{\text{data}} - \mathbf{x}}{\alpha_t^2 \sigma^2_{\text{data}}/\lambda_0 + (1-\alpha_t^2)\sigma^2_{\text{prior}}}.$$

Based on this, we can express the perturbed score function as a *time-dependent rescaling* of the original score function with scaling based on the ratios of the time-dependent inverse variances as

$$\mathbf{s}_t^{\text{perturb}} = \mathbf{s}_t \frac{(1-\alpha_t^2)\sigma^2_{\text{prior}} + \alpha_t^2 \sigma^2_{\text{data}}}{(1-\alpha_t^2)\sigma^2_{\text{prior}} + \alpha_t^2 \sigma^2_{\text{data}}/\lambda_0}.$$

Therefore, to achieve a particular inverse temperature λ_0 for the data distribution, we should rescale the learned score function by time-dependent factor

$$\lambda_t = \frac{(1-\alpha_t^2)\sigma^2_{\text{prior}} + \alpha_t^2 \sigma^2_{\text{data}}}{(1-\alpha_t^2)\sigma^2_{\text{prior}} + \alpha_t^2 \sigma^2_{\text{data}}/\lambda_0} \approx \frac{\lambda_0}{\alpha_t^2 + (1-\alpha_t^2)\lambda_0}$$

where in the last step we assumed $\sigma^2_{\text{data}} = \sigma^2_{\text{prior}}$. So one interpretation of the previously observed insufficiency of low temperature sampling based on score-rescaling [80] is that these were hampered by *uniform* (constant temperature or isothermal) rescaling the score function in time instead of a way that accounts for the shift of influence between the prior and the data distribution.

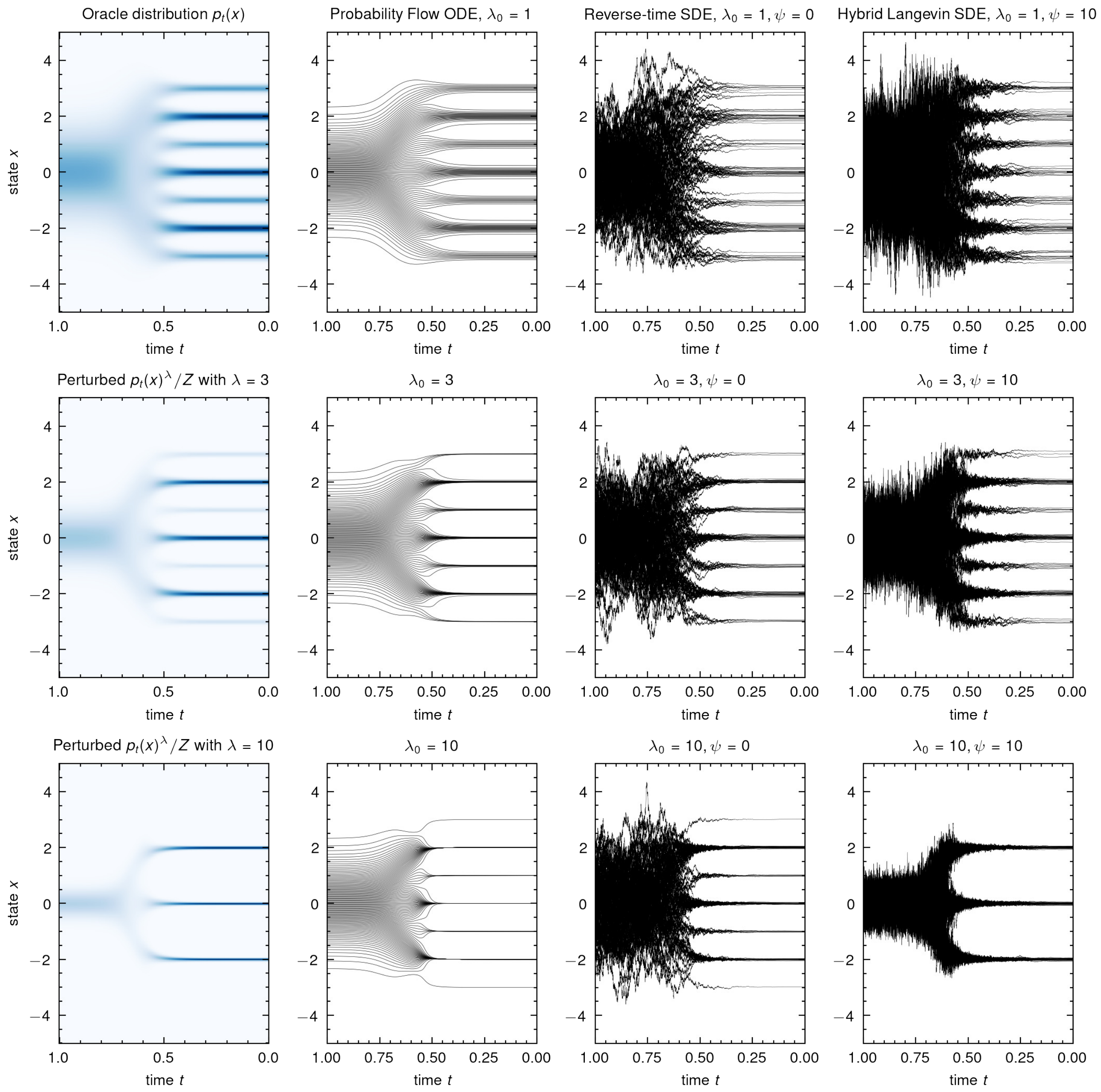

Supplementary Figure 1: **The Hybrid Langevin SDE can sample from temperature-perturbed distributions.** The marginal densities of the diffusion process $p_t(x)$ (top left) gradually transform between a toy 1D data distribution at time $t = 0$ and a standard normal distribution at time $t = T$. Reweighting the distribution by inverse temperature λ_0 as $\frac{1}{Z_{\lambda_0}} p_t(x)^{\lambda_0}$ (left column, bottom two rows) will both concentrate and reweight the population distributions. The annealed versions of the reverse-time SDE and Probability Flow ODEs (middle columns) can concentrate towards local optima but do not correctly reweight the relative population occupancies. Adding in Langevin dynamics with the Hybrid Langevin SDE (right column) increases the rate of equilibration to the time-dependent marginals and, when combined with low-temperature rescaling, successfully reweights the populations (bottom right).

Temperature-adjusted reverse time SDE We can modify the reverse-time SDE by simply rescaling the score function with the above time-dependent temperature rescaling as

$$\mathrm{d}\mathbf{x} = h_t\ \mathbf{x} - g_t^2\ \lambda_t \mathbf{R}\mathbf{R}^\intercal \nabla_{\mathbf{x}} \log p_t(\mathbf{x})\ \mathrm{d}t + g_t\ \mathbf{R}\ \mathrm{d}\bar{\mathbf{w}}.$$

For the variance-preserving process, this reduces to

$$\begin{aligned}\mathrm{d}\mathbf{x} &= \left(-\frac{1}{2}\ \mathbf{x} -\ \lambda_t \mathbf{R}\mathbf{R}^\intercal \nabla_{\mathbf{x}} \log p_t(\mathbf{x})\right) \beta_t\ \mathrm{d}t + \sqrt{\beta_t}\ \mathbf{R}\ \mathrm{d}\bar{\mathbf{w}} \\ &= \left(-\frac{1}{2}\ \mathbf{x} - \lambda_t \frac{\alpha_t \hat{\mathbf{x}}_{\boldsymbol{\theta}}(\mathbf{x},t) - \mathbf{x}}{1-\alpha_t^2}\right) \beta_t\ \mathrm{d}t + \sqrt{\beta_t}\ \mathbf{R}\ \mathrm{d}\bar{\mathbf{w}}.\end{aligned}$$

Temperature adjusted probability flow ODE Similarly for the Probability Flow ODE we can rescale as

$$\frac{\mathrm{d}\mathbf{x}}{\mathrm{d}t} = h_t \mathbf{x} - \frac{1}{2} g_t^2 \lambda_t \mathbf{R}\mathbf{R}^\intercal \nabla_{\mathbf{x}} \log p_t(\mathbf{x}).$$

For the variance-preserving process, this reduces to

$$\begin{aligned}\frac{\mathrm{d}\mathbf{x}}{\mathrm{d}t} &= -\frac{\beta_t}{2} \left(\mathbf{x} + \lambda_t \mathbf{R}\mathbf{R}^\intercal \nabla_{\mathbf{x}} \log p_t(\mathbf{x})\right) \\ &= \frac{\beta_t}{2} \left(\mathbf{x} \frac{\alpha_t + \lambda_t - 1}{1-\alpha_t^2} - \hat{\mathbf{x}}_{\boldsymbol{\theta}}(\mathbf{x},t) \frac{\lambda_t \alpha_t}{1-\alpha_t^2}\right).\end{aligned}$$

Rescaling does not reweight We derived the above rescaling rationale by considering a unimodal Gaussian, which has the simple property that the score of the perturbed diffusion can be expressed as a rescaling of the learned diffusion. This will not be true in general, and sure enough we find that the above dynamics *do drive towards local maxima* but *do not reweight populations based on their relative probability* (Supplementary Figure 1) as true low temperature sampling does. To address this, we next introduce an equilibration process that can be arbitrarily mixed in with the non-equilibrium reverse dynamics. Concurrent with this work, [84] identified this problem as well and proposed several potential solutions based on MCMC.

C.2 Annealed Langevin Dynamics SDE

Instead of reversing the forwards time diffusion in a non-equilibrium manner, we can also use the learned time-dependent score function $\nabla_{\mathbf{x}} \log p_t(\mathbf{x})$ (expressed in terms of the optimal denoiser $\hat{\mathbf{x}}_{\boldsymbol{\theta}}(\mathbf{x},t)$) to do slow, approximately equilibrated sampling with annealed Langevin dynamics [85].

While the annealed Langevin dynamics of [85] was originally framed via discrete iteration, we can recast it in continuous reverse-time with the SDE

$$\mathrm{d}\mathbf{x} = -g_t^2 \frac{\psi}{2} \mathbf{R}\mathbf{R}^\intercal \lambda_0 \nabla_{\mathbf{x}} \log p_t(\mathbf{x})\ \mathrm{d}t + g_t \sqrt{\psi}\ \mathbf{R}\ \mathrm{d}\bar{\mathbf{w}}.$$

For variance-preserving process, this reduces to

$$\mathrm{d}\mathbf{x} = -\frac{\beta_t \psi}{2} \lambda_0 \mathbf{R}\mathbf{R}^\intercal \nabla_\mathbf{x} \log p_t(\mathbf{x})\ \mathrm{d}t + \sqrt{\beta_t \psi}\ \mathbf{R}\ \mathrm{d}\bar{\mathbf{w}},$$

where ψ is an "equilibration rate" scaling the amount of Langevin dynamics per unit time. As $\psi \to \infty$ the system will instantly equilibrate over time (require infinite number of sampling steps), constantly adjusting to the changing score function. In practice, we can think about how to set these parameters by considering a single Euler-Maruyama integration step in reverse time with step size $\frac{1}{T}$ where T is the total number of steps

$$\mathbf{x}_{t-\frac{1}{T}} \leftarrow \mathbf{x}_t + \frac{\beta_t \psi}{2T} \lambda_0 \mathbf{R}\mathbf{R}^\intercal \nabla_{\mathbf{x}_t} \log p_t(\mathbf{x}_t) + \sqrt{\frac{\beta_t \psi}{T}}\ \mathbf{R}\ \boldsymbol{\epsilon} \qquad\qquad \boldsymbol{\epsilon} \sim \mathcal{N}(\mathbf{0}, \mathbf{I}),$$

which is precisely preconditioned Langevin dynamics with step size $\frac{\beta_t \psi}{T}$. For a sufficiently small interval $(t - \mathrm{d}t, t)$ we can keep the target density approximately fixed while increasing T to do an arbitrarily large number of Langevin dynamics steps, which will asymptotically equilibrate to the current density $\log p_t(\mathbf{x})$.

C.3 Hybrid Langevin Reverse-time SDE

We can combine the annealed Reverse-time SDE and the Langevin Dynamics SDE into a hybrid SDE that infinitesimally combines both dynamics. Denoting the inverse temperature as λ_0 and the ratio of the Langevin dynamics to conventional dynamics as ψ, we have

$$\mathrm{d}\mathbf{x} = h_t\ \mathbf{x} - g_t^2 \left(\lambda_t + \frac{\lambda_0 \psi}{2}\right) \mathbf{R}\mathbf{R}^\intercal \nabla_{\mathbf{x}_t} \log p_t(\mathbf{x}_t)\ \mathrm{d}t + g_t \sqrt{(1+\psi)}\ \mathbf{R}\ \mathrm{d}\bar{\mathbf{w}}.$$

For the variance-preserving process, it reduces to

$$\begin{aligned}
\mathrm{d}\mathbf{x} &= \left(-\frac{1}{2}\,\mathbf{x} - \left(\lambda_t + \frac{\lambda_0 \psi}{2}\right) \mathbf{R}\mathbf{R}^\intercal \nabla_{\mathbf{x}_t} \log p_t(\mathbf{x}_t)\right) \beta_t\ \mathrm{d}t + \sqrt{\beta_t (1+\psi)}\ \mathbf{R}\ \mathrm{d}\bar{\mathbf{w}} \\
&= \left(-\frac{1}{2}\,\mathbf{x} - \left(\lambda_t + \frac{\lambda_0 \psi}{2}\right) \mathbf{R}\mathbf{R}^\intercal \frac{(\mathbf{R}\mathbf{R}^\intercal)^{-1}}{1-\alpha_t^2} (\alpha_t \hat{\mathbf{x}}_\theta(\mathbf{x}, t) - \mathbf{x})\right) \beta_t\ \mathrm{d}t + \sqrt{\beta_t (1+\psi)}\ \mathbf{R}\ \mathrm{d}\bar{\mathbf{w}} \\
&= \left(-\frac{1}{2}\,\mathbf{x} - \left(\lambda_t + \frac{\lambda_0 \psi}{2}\right) \frac{\alpha_t \hat{\mathbf{x}}_\theta(\mathbf{x}, t) - \mathbf{x}}{1-\alpha_t^2}\right) \beta_t\ \mathrm{d}t + \sqrt{\beta_t (1+\psi)}\ \mathbf{R}\ \mathrm{d}\bar{\mathbf{w}},
\end{aligned}$$

where we highlight in pink the terms that, when set to unity, recover the standard reverse time SDE.

Representative samples using this modified SDE are shown in Supplementary Figure 2. Without the low temperature modification, this idea is very reminiscent of the Predictor Corrector sampler proposed by [55], where those authors explicitly alternated between reverse-time diffusion and Langevin dynamics while we fuse them into a single SDE.

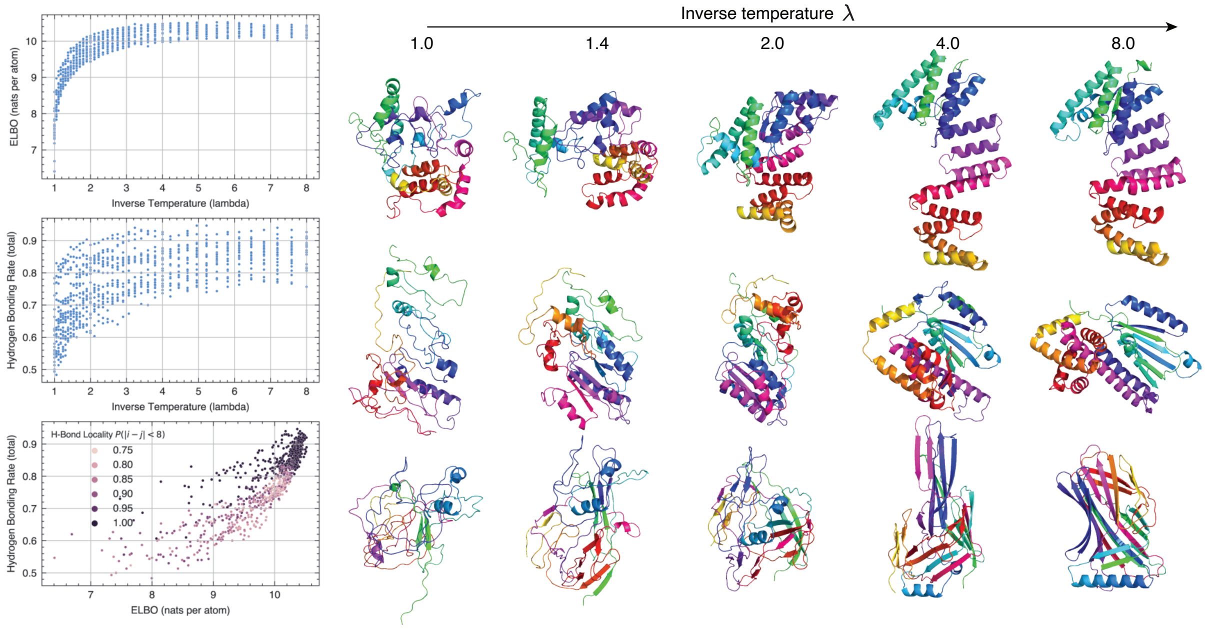

Supplementary Figure 2: **Low-temperature sampling drives towards high-likelihood states with increased secondary structure content.** Increasing the inverse temperature λ increases the likelihood (ELBO) for unconditional samples from the backbone diffusion model (left, top). These high-likelihood states exhibit increased rates of backbone hydrogen bonding that underlie secondary structure (left, middle). We observe that the ELBO itself (which is sequence-independent) is strongly associated with hydrogen bonding rates, and the highest likelihood states are particularly associated with increased *locality* of hydrogen bonding at the primary sequence distance $|i < j| < 8$ (left, bottom). These relationships can be seen within the evolution of single samples under fixed random seeds (each row, right), where structures sampled at higher inverse temperature λ have increased secondary structure content and tighter packing as compact globular folds. The model shown is `ChromaBackbone v0`, while `ChromaBackbone v1` generally has higher secondary structure compositions at lower inverse temperature.

Equilibration is not free Generally speaking, as we increase the amount of Langevin equilibration with ψ, we will need to simultaneously increase the resolution of our SDE solution to maintain the same level of accuracy. However, we found that even a modest amount of equilibration was sufficient to considerably improve sample quality in practice with $\psi \in [1,8]$. With a larger ψ, a smaller time step is needed to ensure the accuracy of SDE integration.

Even more equilibration Lastly, while the Hybrid Langevin-Reverse Time SDE can do an arbitrarily large amount of Langevin dynamics per time interval which would equilibrate asymptotically in principle, these dynamics will still inefficiently mix between basins of attraction in the energy landscape when $0 < t \ll 1$. We suspect that ideas from variable-temperature sampling methods, such as simulated tempering [86] or parallel tempering [87], would be useful in this context and would amount to deriving an augmented SDE system with auxiliary variables for the temperature and/or copies of the system at different time points in the diffusion. Additionally, momentum-aware approaches such as those based on Hamiltonian Monte Carlo [84] may help

increase equilibration rates and thus enable better satisfication of conditioning criteria with fewer objective function evaluations.

D Polymer-Structured Diffusions

Most prior applications of diffusion models to images and molecules leveraged uncorrelated diffusion in which data are gradually transformed by isotropic Gaussian noise. We found this approach to be non-ideal for protein structure applications for two reasons. First, noised samples break simple chain and density constraints that almost all structures satisfy such as basic size scaling laws of the form $R_g \propto N^\nu$, where the scaling exponent is approximately $\nu \approx 0.4$ [88, 89]. These mismatches between the data distribution and the noising process force the model to allocate capacity and training time towards re-learning basic and well-understood constraints. Second, when high-noise samples are highly "out-of-distribution" from the data distribution, this can limit the performance of efficient domain-specific neural architectures for molecular systems, such as sparsely-connected graph neural networks. To this end, we introduce multivariate Gaussian distributions for protein structures that (i) are SO(3) invariant, (ii) enforce protein chain and radius of gyration statistics, and (iii) can be computed in linear time. Throughout this section, we will introduce covariance models for protein polymers (which can be thought of as a un-whitening transform $\mathbf{R}$, see Appendix B) with parameters that can be fit *offline* from training the diffusion model. We provide an overview figure that illustrates the different Gaussian distributions presented in this section, their corresponding diffusion processes, and the respective distance statistics which they capture in Supplementary Figure 3.

D.1 Diffusion processes predictably affect molecular distances

Here we show how variance-preserving diffusion processes (Supplementary Appendix B) will predictably affect molecular geometry as a function of the covariance structure of the noising process. We will use this result to reflect on how the covariance structure should be designed. Squared distance D_{ij}^2 and the squared radius of gyration R_g^2 are both functions that can be expressed as quadratic forms in the coordinates. That means they can be expressed as a function $\mathcal{F}(\mathbf{x}) = \mathbf{x}^\intercal \mathbf{A}\mathbf{x}$ where $\mathbf{A}$ is a matrix weighting the cross-terms as $\mathcal{F}(\mathbf{x}) = \sum_{i,j} A_{ij} x_i x_j$. Suppose that we want to understand the behavior of these quantities as they evolve under the forward process of a diffusion model. Recall that we can write samples from the forward diffusion process as

$$\mathbf{x}_t = \alpha_t \mathbf{x}_0 + \sqrt{1-\alpha_t^2}\mathbf{R}\mathbf{z}, \quad \mathbf{z} \sim \mathcal{N}(0, \mathbf{I}).$$

So we can write the time-expectation of any quadratic form as

$$\begin{aligned}
\mathbb{E}_{p(\mathbf{x}_t|\mathbf{x}_0)}\left[\mathcal{F}(\mathbf{x})\right] &= \mathbb{E}_{\mathbf{z}}\left[(\alpha_t \mathbf{x}_0 + \sqrt{1-\alpha_t^2}\mathbf{R}\mathbf{z})^\intercal \mathbf{A}(\alpha_t \mathbf{x}_0 + \sqrt{1-\alpha_t^2}\mathbf{R}\mathbf{z})\right] \\
&= \mathcal{F}(\alpha_t \mathbf{x}_0) + \mathbb{E}_{\mathbf{z}}\left[\mathcal{F}(\sqrt{1-\alpha_t^2}\mathbf{R}\mathbf{z}) + \alpha_t\sqrt{(1-\alpha_t^2)}\left(\mathbf{x}_0^\intercal \mathbf{R}\mathbf{z} + \mathbf{R}\mathbf{z}^\intercal \mathbf{x}_0\right)\right] \\
&= \alpha_t^2\, \mathcal{F}(\mathbf{x}_0) + \mathbb{E}_{\mathbf{z}}\left[\mathcal{F}(\sqrt{1-\alpha_t^2}\mathbf{R}\mathbf{z})\right] \\
&= \alpha_t^2\, \mathcal{F}(\mathbf{x}_0) + (1-\alpha_t^2)\, \mathbb{E}_{p_{\text{model}}(\mathbf{x})}\left[\mathcal{F}(\mathbf{x})\right].
\end{aligned}$$

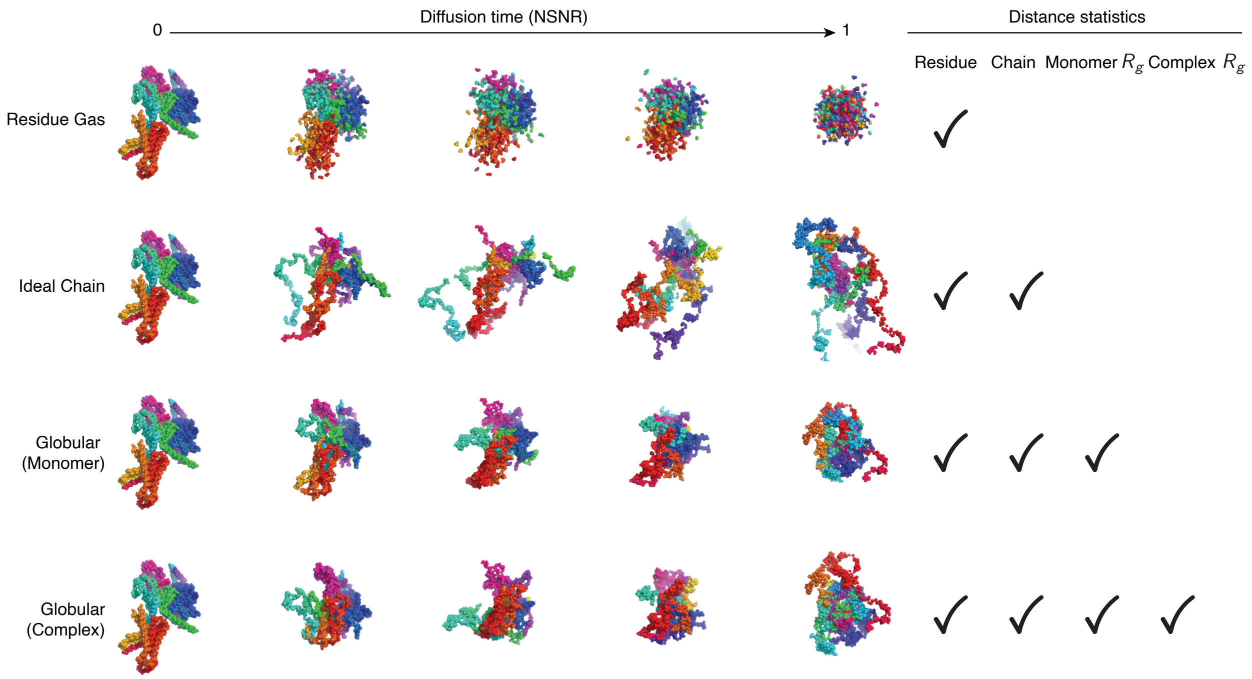

Supplementary Figure 3: **Polymer-structured diffusions capture multiple scales of distance statistics in proteins.** A residue gas covariance model (top row, Appendix D.4) enforces atomic proximity within residues, but ignores chain correlations and length-dependent R_g scaling effects. The ideal chain covariance model (second row, Appendix D.2), a standard entry point for polymer physics, captures atomic proximity along a chain but does not capture the length-dependent R_g scaling driven by polymer collapse. The globular covariance model (third and fourth rows, Appendix D.3), combines chain covariance with an analytic scaling law that reproduces the empirical R_g scaling of globular proteins and complexes. All of these covariance models admit computation of matrix-vector products involving covariance and inverse-covariance matrices with linear time complexity.

The squared distance is a quadratic form, so diffusion processes will simply linearly interpolate to the behavior of the prior as

$$\mathbb{E}_{p(\mathbf{x}_t|\mathbf{x}_0)}\left[D_{ij}^2(\mathbf{x}_t)\right] = \alpha_t^2\, D_{ij}^2(\mathbf{x}_0) + (1-\alpha_t^2)\,\mathbb{E}_{p_{\text{prior}}(\mathbf{x})}\left[D_{ij}^2(\mathbf{x})\right]$$

and squared radius of gyration will similarly evolve under the diffusion as

$$\mathbb{E}_{p(\mathbf{x}_t|\mathbf{x}_0)}\left[R_g^2(\mathbf{x}_t)\right] = \alpha_t^2\, R_g^2(\mathbf{x}_0) + (1-\alpha_t^2)\,\mathbb{E}_{p_{\text{prior}}(\mathbf{x})}\left[R_g^2(\mathbf{x})\right].$$

Punchline Because variance-preserving diffusion models will perform simple linear interpolations between the average squared distances and R_g of the data distribution and of the prior, we should focus on covariance structures that empirically match these properties as closely as possible. Two primary ways will be in the *chain constraint*, i.e., that $D_{i,i+1}(\mathbf{x}_t)$ should always be small and match the data distribution, and the *density constraint* of how $R_g^2(\mathbf{x}_t)$ should behave as a function of protein length and typical packing statistics.

D.2 Covariance model #1: Ideal Chain

In this section, we introduce one of the simplest covariance models that enforces the chain constraint but ignores the R_g scaling. It will interpolate between the data distribution and the ensemble of unfolded random coils.

Noise process We index our amount of noise with a diffusion time $t \in [0,1]$. Given a denoised structure $\mathbf{x}_0$, a level of noise t, and a noise schedule α_t, we sample perturbed structures from a Multivariate Gaussian distribution $p(\mathbf{x}_t|\mathbf{x}_0) = \mathcal{N}(\alpha_t \mathbf{x}_0, (1-\alpha_t^2)\Sigma)$ as

$$\mathbf{x}_t = \alpha_t \mathbf{x}_0 + \sqrt{1-\alpha_t^2}\mathbf{R}\mathbf{z}, \quad \mathbf{z} \sim \mathcal{N}(0, \mathbf{I}),$$

where the covariance matrix enforcing our chain constraint $\Sigma = \mathbf{R}\mathbf{R}^\intercal$ can be expressed in terms of its square root $\mathbf{R}$, which is defined below.

Key to our framework is a matrix $\mathbf{R}$ whose various products, inverse-products, and transpose-products with vectors can be computed in *linear* time. We define the matrix $\mathbf{R}$ in terms of its product with a vector $f(\mathbf{z}) = \mathbf{R}\mathbf{z}$ as

$$f(\mathbf{z})_i = \tilde{x}_i + \delta \tilde{x}_1 - \sum_k \frac{\tilde{x}_k}{N}, \quad \text{where} \quad \tilde{x}_i = a \sum_{k=1}^{i} z_k.$$

The inverse product $f^{-1}(\mathbf{x}) = \mathbf{R}^{-1}\mathbf{x}$ is then

$$f^{-1}(\mathbf{x})_i = \frac{\tilde{x}_i - \tilde{x}_{i-1}}{a}, \quad \text{where} \quad \tilde{x}_i = x_i - x_1 + \frac{1}{\delta} \sum_k \frac{x_k}{N}.$$

This definition of $\mathbf{R}$ induces the following inverse covariance matrix on the noise, which possesses a special structure of

$$\Sigma^{-1} = (\mathbf{R}\mathbf{R}^\intercal)^{-1} = \frac{1}{a^2} \begin{bmatrix} 1 & -1 & & & & \\ -1 & 2 & -1 & & & \\ & -1 & 2 & -1 & & \\ & & \ddots & \ddots & \ddots & \\ & & & -1 & 2 & -1 \\ & & & & -1 & 1 \end{bmatrix} + \frac{1}{(Na\delta)^2}\mathbf{1}\mathbf{1}^\intercal.$$

The parameter a sets the length scale of the chain and the parameter δ sets the allowed amount of translational noise about the origin. This latter parameter is important for training on complexes where each chain may not have a center of mass at 0.

D.2.1 Covariance model #1 has ideal chain scaling $R_g \propto N^{(1/2)}$

Our ideal-chain model is a simple Brownian motion and so the interatomic residual is Gaussian distributed with zero mean and $a^2|i-j|$ variance, i.e.,

$$\mathbf{r}_{ij} \sim \mathcal{N}(0, a^2|i-j|).$$

The expected squared norm for a Multivariate Normal Distribution (MVN) with spherical covariance is $||\boldsymbol{\mu}||_2^2 + k\sigma^2$ where k is the dimensionality, so we have

$$\mathbb{E}_{p(\mathbf{x}_t|\mathbf{x}_0)}\left[D_{ij}^2(\mathbf{x}_t)\right] = \alpha_t^2 \, D_{ij}^2(\mathbf{x}_0) + (1-\alpha_t^2)3a^2|i-j|.$$

When $\alpha_t = 0$, the expected squared distances are those of the data distribution, while when $\alpha_t = T$, they are those on an ideal Gaussian chain.

To compute the expected radius of Gyration, we can use the identity that it is simply half of the root mean square of inter-residue distances

$$\begin{aligned}
\frac{1}{2N^2}\sum_{i,j}\mathbb{E}_{p_{\text{prior}}}\left[||\mathbf{x}_t^j - \mathbf{x}_t^i||_2^2\right] &= +\frac{1}{2N^2}\sum_{i,j}(1-\alpha)3a^2|i-j| \\
&= 3a^2\frac{1}{2N^2}\sum_{i,j}|i-j| \\
&= 3a^2\frac{1}{N^2}\sum_{i=1}^{N}\sum_{j=i}^{N} j-i \\
&= 3a^2\frac{N}{6}\left(\frac{N^2-1}{N^2}\right).
\end{aligned}$$

Therefore, we can also view the mean behavior of the diffusion as linearly interpolating the squared radius of gyration as

$$\mathbb{E}_{p(\mathbf{x}_t|\mathbf{x}_0)}\left[R_g^2(\mathbf{x})\right] = \alpha_t^2\left(R_g^{(0)}\right)^2 + (1-\alpha_t^2)3a^2\frac{N}{6}\left(\frac{N^2-1}{N^2}\right).$$

When $\alpha \to 0$ and $N \ll 0$, the term $\left(\frac{N^2-1}{N^2}\right) \approx 1$ we recover the well-known scaling for an ideal chain with $\mathbb{E}_{p(\mathbf{x}_t|\mathbf{x}_0)}\left[R_g^2(\mathbf{x}_t)\right] = \frac{Nl^2}{6}$ where the segment length is $l = \sqrt{3}a$.

D.3 Covariance model #2: R_g-confined Globular Polymer

In this section we consider how to extend the previous model in a way that preserves the chain constraint while further restricting the scaling of the radius of gyration R_g. We consider a family of two-parameter linear chain models that include the previous model as a special case. Specifically, consider the following linear recurrence

$$\begin{aligned}
x_i &= az_i + bx_{i-1} \\
&= a\sum_{j=2}^{i} b^{i-j}z_j + b^{i-1}x_1.
\end{aligned}$$

Here, the parameter a is a global scale parameter setting the "segment length" of the polymer and b is a "decay" parameter which sets the memory of the chain to fluctuations. Informally, at each step along the chain, we *bury* $1-b$ percent of the way to the origin and *step* in a random direction with step scale a. We recover a spherical Gaussian when $b=0$ and the ideal Gaussian chain when $b=1$.

This system can also be written in matrix form as $\mathbf{x}=\mathbf{R}\mathbf{z}$ with

$$\mathbf{R}=a\begin{bmatrix} vb^0 & & & & & \\ vb^1 & b^0 & & & & \\ vb^2 & b^1 & b^0 & & & \\ \vdots & & \ddots & \ddots & & \\ vb^{N-2} & & & b^1 & b^0 & \\ vb^{N-1} & & \cdots & b^2 & b^1 & b^0 \end{bmatrix}$$

where $v=\sqrt{\mathrm{Var}(x_1)}$.

We can solve for the equilibrium value of v via the condition $\mathrm{Var}(x_1)=a^2v^2=\mathrm{Var}(x_i)=\mathrm{Var}(x_{i-1})$. The solution is

$$\begin{aligned}\mathrm{Var}(x_i)&=a^2\,\mathrm{Var}(z_i)+b^2\,\mathrm{Var}(x_i)\\ \mathrm{Var}(x_i)(1-b^2)&=a^2\\ a^2v^2&=\frac{a^2}{1-b^2}\\ v&=\frac{1}{\sqrt{1-b^2}}.\end{aligned}$$

So our final recurrence is

$$x_i=a\sum_{k=2}^{i}b^{i-k}z_k+a\frac{b^{i-1}}{\sqrt{1-b^2}}z_1.$$

D.3.1 Expected Radius of Gyration $\mathbb{E}[R_g^2]$ as a function of b

To compute the expected Radius of Gyration, we will use the identity $R_g^2(\mathbf{x})=\frac{1}{2N^2}\sum_{i,j}D_{ij}^2(\mathbf{x})$, which we can compute via the variance of the residual between x_i and x_j. Assuming $j>i$, we have

$$\frac{x_j-x_i}{a}=\sum_{k=i+1}^{j}b^{j-k}z_k+\sum_{k=2}^{i}(b^{j-k}-b^{i-k})z_k+\frac{b^{j-1}-b^{i-1}}{\sqrt{1-b^2}}z_1,$$

the variance of which is

$$\begin{aligned}
\frac{1}{a^2}\mathbb{E}\left[D_{ij}^2(\mathbf{x})\right] &= \frac{1}{a^2}\mathrm{Var}(x_j - x_i) \\
&= \mathrm{Var}\left(\sum_{k=2}^{j} b^{j-k} z_k + \frac{b^{j-1}}{\sqrt{1-b^2}} z_1 - \sum_{k=2}^{i} b^{i-k} z_k - \frac{b^{i-1}}{\sqrt{1-b^2}} z_1\right) \\
&= \mathrm{Var}\left(\sum_{k=i+1}^{j} b^{j-k} z_k + \sum_{k=2}^{i}\left(b^{j-k} - b^{i-k}\right) z_k + \frac{b^{j-1} - b^{i-1}}{\sqrt{1-b^2}} z_1\right) \\
&= \sum_{k=i+1}^{j} b^{2(j-k)} + \sum_{k=2}^{i} (b^{j-k} - b^{i-k})^2 + \frac{(b^{j-1} - b^{i-1})^2}{1-b^2} \\
&= \frac{2(1-b^{j-i})}{1-b^2}.
\end{aligned}$$

So the expected R_g^2 is

$$\begin{aligned}
\frac{1}{a^2}\mathbb{E}\left[R_g^2(\mathbf{x})\right] &= \frac{1}{a^2}\mathbb{E}\left[\frac{1}{N^2}\sum_{i=1}^{N}\sum_{j=i}^{N} D_{ij}^2(\mathbf{x})\right] \\
&= \frac{1}{N^2}\sum_{i=1}^{N}\sum_{j=i}^{N}\frac{1}{a^2}\mathbb{E}\left[D_{ij}^2(\mathbf{x})\right] \\
&= \frac{1}{N^2}\sum_{i=1}^{N}\sum_{j=i}^{N}\frac{2(1-b^{j-i})}{1-b^2} \\
&= \frac{2b^{N+1} - b^2 N(N+1) + 2b(N^2-1) - N(N-1)}{(b-1)^3(b+1)N^2} \\
&\approx \left(\frac{6b}{N} + 1 - b^2\right)^{-1} \qquad \text{for } b \text{ on } (0,1) \text{ and } N \gg 1 \\
&= \frac{N}{6b + N(1-b^2)}.
\end{aligned}$$

The approximation in the penultimate step works quite well in practice and becomes more accurate with growing N, which we can verify with the limit

$$\forall b \in (0,1) \lim_{N\to\infty} \frac{2b^{N+1} - b^2 N(N+1) + 2b(N^2-1) - N(N-1)}{(b-1)^3(b+1)N^2}\left(\frac{6b}{N} + 1 - b^2\right) = 1.$$

Limiting Behaviors We can verify that this result reproduces the expected limiting behavior of an ideal unfolded chain when $b \to 1$ as

$$\lim_{b\to 1}\frac{1}{a^2}\mathbb{E}\left[R_g^2(\mathbf{x})\right] = \frac{N}{6},$$

and of a standard normal distribution when $b \to 0$ as

$$\lim_{b\to 0}\frac{1}{a^2}\mathbb{E}\left[R_g^2(\mathbf{x})\right] = 1.$$

R_g^2 Scaling To finish up, we can add back in our global scaling factor a to give

$$\mathbb{E}_{\mathbf{x}\sim\mathbf{p}_{\text{prior}}(\mathbf{x})}\left[R_g^2(\mathbf{x})\right] \approx \frac{Na^2}{6b+N(1-b^2)}.$$

D.3.2 How to implement any R_g^2 scaling

Empirical analysis and biophysical models suggest that protein radii of gyration R_g will scale with the number of residues N with scaling law

$$R_g = rN^{\nu},$$

where $r \approx 2.0Å$ and $\nu \approx 0.4$ [88, 89].

Given this expected behavior of R_g^2 as a function of N, we can solve for the value of $b(N)$ that implements the correct scaling by solving

$$\mathbb{E}_{\mathbf{x}\sim\mathbf{p}_{\text{prior}}(\mathbf{x})}\left[R_g^2(\mathbf{x})\right] = (rN^{\nu})^2 = \frac{Na^2}{6b+N(1-b^2)}.$$

This gives a quadratic equation with the solution

$$b_{\text{effective}}(N,a,r,\nu) = \frac{3}{N} \pm N^{-\nu}\sqrt{N^{2(\nu-1)}(N^2+9) - \frac{a^2}{r^2}},$$

where the positive branch is the relevant one to us (the negative branch corresponds to a pathological solutions for small N), giving us the final result

$$b_{\text{effective}}(N,a,r,\nu) = \frac{3}{N} + N^{-\nu}\sqrt{N^{2(\nu-1)}(N^2+9) - \frac{a^2}{r^2}}.$$

D.3.3 Standardizing the translational variance

Initializing the above recurrence relationship at equilibrium yields diverging marginal variance as $b \to 1$. We can arbitrarily re-tune the *translational variance* of each chain with the following mean-deflation operation enforcing $\sum_k \frac{x_k}{N} = (1-\xi)\sum_k \frac{\tilde{x}_k}{N}$ as

$$x_i = \tilde{x}_i - \xi \sum_k \frac{\tilde{x}_k}{N}.$$

This operation has the inverse

$$\tilde{x}_i = x_i + \frac{\xi}{1-\xi}\sum_k \frac{x_k}{N}.$$

D.3.4 Setting the parameters

First, we set the dimension-wise segment scaling factor $a = 1.559$ by fitting uniformly random ϕ, ψ chains with ideal geometry. We then dynamically set b for each chain to satisfy its predicted R_g scaling with the relationship $b_{\text{effective}}(N_{\text{atoms}}, a, r, \nu) = \frac{3}{N_{\text{atoms}}} + N_{\text{atoms}}^{-\nu}\sqrt{N_{\text{atoms}}^{2(\nu-1)}(N_{\text{atoms}}^2 + 9) - \frac{a^2}{r^2}}$, $\nu = 0.4$, $r = 0.66$, and $N_{\text{atoms}} = 4N_{\text{residues}}$. We have two procedures for setting the values of ξ, leading to two different named covariance models:

1. **Monomer R_g scaling**. Set ξ so that the translational variance of each chain is unity. This will cause chains to have a realistic radius of gyration but pile up at the origin.
2. **Complex R_g scaling**. Set ξ per chain by solving for the translational variance that also implements the correct whole-complex R_g scaling as a function of the number of residues. This will cause chains to preserve a realistic complex-level radius of gyration and also intra-chain radius of gyration that scales as that of individual globular proteins.

D.3.5 Covariance factors and their inverses

When also including a centering transform, we can factorize the square root of the covariance matrix, $\Sigma^{\frac{1}{2}} \triangleq \mathbf{R}$, as a product of three matrices,

$$
\begin{aligned}
\mathbf{R} &= a\mathbf{R}_{\text{center}}\mathbf{R}_{\text{sum}}\mathbf{R}_{\text{init}} \\
&= a\left(\mathbf{I} - \frac{\xi}{N}\mathbf{1}\mathbf{1}^{\mathsf{T}}\right)
\begin{bmatrix}
b^0 & & & & & \\
b^1 & b^0 & & & & \\
b^2 & b^1 & b^0 & & & \\
\vdots & & \ddots & \ddots & & \\
b^{N-2} & & & b^1 & b^0 & \\
b^{N-1} & & \cdots & b^2 & b^1 & b^0
\end{bmatrix}
\begin{bmatrix}
\frac{1}{\sqrt{1-b^2}} & & & & & \\
 & 1 & & & & \\
 & & 1 & & & \\
 & & & \ddots & & \\
 & & & & 1 & \\
 & & & & & 1
\end{bmatrix}.
\end{aligned}
$$

Each of these matrices can be multiplied with a vector with linear time and space complexity, as $\mathbf{R}_{\text{center}}$ is a global shift, $\mathbf{R}_{\text{sum}}$ is a simple linear filter, and $\mathbf{R}_{\text{init}}$ is a single-element adjustment. Similarly, we can build up the inverse of the matrix square root as

$$
\mathbf{R}^{-1} = \frac{1}{a}\mathbf{R}_{\text{init}}^{-1}\mathbf{R}_{\text{sum}}^{-1}\mathbf{R}_{\text{center}}^{-1},
$$

where the factor-wise inverses are

$$
\mathbf{R}_{\text{init}}^{-1} =
\begin{bmatrix}
\sqrt{1-b^2} & & & & & \\
 & 1 & & & & \\
 & & 1 & & & \\
 & & & \ddots & & \\
 & & & & 1 & \\
 & & & & & 1
\end{bmatrix},
$$

and, via solving $x_i = \sum_{j=1}^{i} b^{i-j} z_j$ for z_i,

$$\mathbf{R}_{\text{sum}}^{-1} = \begin{bmatrix} 1 & & & & & \\ -b & 1 & & & & \\ & -b & 1 & & & \\ & & \ddots & \ddots & & \\ & & & -b & 1 & \\ & & & & -b & 1 \end{bmatrix},$$

and, via the matrix inversion lemma,

$$\mathbf{R}_{\text{center}}^{-1} = \mathbf{I} + \frac{\xi}{(1-\xi)N}\mathbf{1}\mathbf{1}^{\mathsf{T}}.$$

D.3.6 Covariance determinant

Computing likelihoods of protein chains under the multivariate normal prior introduced in this section or computing the Diffusion ELBO from Appendix B requires computation of the determinant of the covariance matrix. Fortunately, the simple form of our covariance model leads to a simple form for the determinant. With a chain length of N, we have

$$\begin{aligned} \log\det\mathbf{R} &= N\log a && + \log\det\mathbf{R}_{\text{center}} + \log\det\mathbf{R}_{\text{sum}} && + \log\det\mathbf{R}_{\text{init}} \\ &= N\log a && + \log\det\left(\mathbf{I} + \left(-\frac{\xi\mathbf{1}}{N}\right)\mathbf{1}^{\mathsf{T}}\right) + N\log b^0 && + -\frac{1}{2}\log(1-b^2) \\ &= N\log a && + \log\left(1 + \mathbf{1}^{\mathsf{T}}\left(-\frac{\xi\mathbf{1}}{N}\right)\right) + 0 && + -\frac{1}{2}\log(1-b^2) \\ &= N\log a && + \log(1-\xi) + 0 && + -\frac{1}{2}\log(1-b^2), \end{aligned}$$

where $\log\det\mathbf{R}_{\text{center}}$ follows from the matrix determinant lemma. Thus, $\det\mathbf{R} = \frac{a^N(1-\xi)}{\sqrt{1-b^2}}$.

D.3.7 Inverse covariance and intuition

We may examine the inverse of the globular covariance matrix to build intuition on the underlying factors driving correlations in our system. For simplicity, we analyze[5] the simpler case of the uncentered covariance model with $\Sigma_{\text{uncentered}} = a\mathbf{R}_{\text{sum}}\mathbf{R}_{\text{init}}a\mathbf{R}_{\text{init}}^{\mathsf{T}}\mathbf{R}_{\text{sum}}^{\mathsf{T}}$. It will be helpful to define $\mathbf{D} \triangleq \mathbf{R}_{\text{init}}^{-\mathsf{T}}\mathbf{R}_{\text{init}}^{-1}$ and to note that the inverse sum operator can be expressed as $\mathbf{R}_{\text{sum}}^{-1} = \mathbf{I} - b\mathbf{P}$, where $\mathbf{P}$ is a nilpotent shift matrix with ones on the first lower diagonal. We then have

$$\mathbf{D} = \begin{bmatrix} 1-b^2 & & & & & \\ & 1 & & & & \\ & & 1 & & & \\ & & & \ddots & & \\ & & & & 1 & \\ & & & & & 1 \end{bmatrix},$$

[5] We thank Rian Kormos for this proof and analysis.

and

$$\begin{aligned}
\Sigma_{\text{uncentered}}^{-1} &= \frac{1}{a^2}\mathbf{R}_{\text{sum}}^{-\mathsf{T}}\mathbf{R}_{\text{init}}^{-\mathsf{T}}\mathbf{R}_{\text{init}}^{-1}\mathbf{R}_{\text{sum}}^{-1} \\
&= \frac{1}{a^2}(\mathbf{I}-b\mathbf{P}^{\mathsf{T}})\mathbf{D}(\mathbf{I}-b\mathbf{P}) \\
&= \frac{1}{a^2}\left(\mathbf{D}-b\left(\mathbf{P}^{\mathsf{T}}\mathbf{D}+\mathbf{D}^{\mathsf{T}}\mathbf{P}\right)+b^2\mathbf{P}^{\mathsf{T}}\mathbf{D}\mathbf{P}\right) \\
&= \frac{1}{a^2}\left(\mathbf{D}-b\left(\mathbf{P}^{\mathsf{T}}+\mathbf{P}\right)+b^2\mathbf{P}^{\mathsf{T}}\mathbf{P}\right) \\
&= \frac{1}{a^2}\begin{bmatrix}
1 & -b & & & & \\
-b & 1+b^2 & -b & & & \\
 & -b & 1+b^2 & -b & & \\
 & & \ddots & \ddots & \ddots & \\
 & & & -b & 1+b^2 & -b \\
 & & & & -b & 1
\end{bmatrix},
\end{aligned}$$

where the penultimate line follows from the behavior of the shift operator $\mathbf{P}$.

We can identify within this precision matrix a linear combination of two well-known precision matrices: the precision of Brownian motion, i.e. the chain Laplacian matrix, and the precision for a spherical Gaussian, i.e. an identity matrix, along some nuisance boundary conditions as

$$\Sigma^{-1} = \frac{1}{a^2}\left(b\begin{bmatrix}
1 & -1 & & & & \\
-1 & 2 & -1 & & & \\
 & -1 & 2 & -1 & & \\
 & & \ddots & \ddots & \ddots & \\
 & & & -1 & 2 & -1 \\
 & & & & -1 & 1
\end{bmatrix} + (1-b)^2\mathbf{I} + \begin{bmatrix}
b(1-b) & & \\
 & & \\
 & & \\
 & & b(1-b)
\end{bmatrix}\right).$$

This provides another simple characterization of our globular covariance model as being the result of a combination of 'chain springs' holding the polymer together locally along with 'burial springs' pulling the chain to the origin. This simple energetic structure has been leveraged in prior biophysical 'toy models' of hydrophobic collapse in proteins [90].

D.4 Alternative covariance model: Residue Gas

One useful parameterization of protein structure that strikes a balance between capturing the strong spatial dependencies induced by covalent bonds while avoiding the accumulated lever effects of internal coordinates is the so-called "Residue Gas" approach of AlphaFold [66]. In this parameterization, each residue is treated as a rigid body with local geometries fixed to their ideal values. This will ensure idealized *intra*-residue geometries by construction, though *inter*-residue covalent bond geometries, i.e. $C_i - N_{i+1}$ bonds, will need to be fixed by the predictor.

Prior work applying diffusion models for protein backbones has modeled the C_α carbons as independently distributed with a fixed variance, i.e. $\mathbf{x}_1^{C\alpha}, \ldots, \mathbf{x}_N^{C\alpha} \sim \mathcal{N}\left(0, \sigma_{C_\alpha}^2\right)$ [67, 91]. While

previous frame-based approaches then model the remaining N, C, O atoms locked to the C_α carbon with variable rotation and ideal geometry, we can simply model these atoms as normally distributed around C_α with a fixed standard deviation σ_{intra}. At full noise levels this will induce an isotropic distribution over implied frame orientations while keeping these atoms close to the parent C_α, and as such can be considered an off-ideality relaxation of frame diffusion models [67] or an all-backbone-atom extension of i.i.d. C_α diffusion models [91].

This sequential Gaussian dependency structure within residues will imply that all coordinates are jointly Gaussian with square root of the covariance matrix

$$\mathbf{R}_{\text{gas}} = \begin{bmatrix} \mathbf{R}_{\text{residue}} & & & \\ & \mathbf{R}_{\text{residue}} & & \\ & & \ddots & \\ & & & \mathbf{R}_{\text{residue}} \end{bmatrix}$$

and with block diagonal elements

$$\mathbf{R}_{\text{residue}} = \begin{bmatrix} \sigma_{\text{intra}} & \sigma_{C_\alpha} & 0 & 0 \\ 0 & \sigma_{C_\alpha} & 0 & 0 \\ 0 & \sigma_{C_\alpha} & \sigma_{\text{intra}} & 0 \\ 0 & \sigma_{C_\alpha} & 0 & \sigma_{\text{intra}} \end{bmatrix}.$$

In our experiments we set the intra-residue standard deviation to $\sigma_{\text{intra}} = 1$ and the residue standard deviation to $\sigma_{C_\alpha} = 10$. As can be seen in Supplementary Figure 3, this covariance implies trajectories that are extremely similar to frame-based diffusion [67], but with the added benefit that we can treat non-ideal bond stretch and angle fluctuations. We do lose the guarantee of fixed internal ideal geometries, but this only requires learning the equivalent of ~ 6 additional numbers.

E Random Graph Neural Networks

Prior approaches to predicting or generating protein structure have relied on neural network architectures with $\mathcal{O}(N^2)$ or $\mathcal{O}(N^3)$ computational complexity [66, 67, 91], in part motivated by the need to process the structure at multiple length scales simultaneously and/or to reason over triples of particles as is done during distance geometry methods. Here, we introduce an effective alternative to these approaches with sub-quadratic complexity by combining Message Passing Neural Network [92] layers with random graph generation processes. We design random graph sampling methods that reproduce the connectivity statistics of efficient N-body simulation methods, such as the Barnes-Hut algorithm [93].

E.1 Background: efficient N-body simulation

One of the principal lessons of computational physics is that N-body simulations involving $\mathcal{O}(N^2)$ dense interactions (e.g. gravitational simulations and molecular physics) can often be effectively simulated with only $\mathcal{O}(N \log N)$-scaling computation. Methods such as Barnes-Hut [93] and the Fast Multipole Method take advantage of a common particular property of (and inductive bias for)

physical systems that *more distant interactions can be modeled more coarsely for the same level of accuracy*. For example, in cosmological simulations, you can approximate the gravitational forces acting on a star in a distant galaxy by approximating that galaxy as a point at its center of mass.

So far, most *relational* machine learning systems [94] for protein structure have tended to process information in a manner that is either based on local connectivity (e.g. a k-Nearest Neighbors or cutoff graphs) [95] or all-vs-all connectivity [66, 67, 91]. The former approach is natural for highly spatially localized tasks such as structure-conditioned sequence design and the characterization of residue environments, but it is less clear if local graph-based methods can effectively reason over global structure in a way that is possible with fully connected Graph Neural Networks, such as Transformers [96]. Here we ask if there might be reasonable ways to add in long-range reasoning while preserving sub-quadratic scaling simply by random graph construction.

Related work Our method evokes similarity to approaches that have been used to scale Transformers to large documents by combining a mixture of local and deterministically [97] or randomly sampled long-range context [98]. Distant-dependent density of context has also been explored in multiresolution attention for Vision transformers [99] and in dilated convolutional neural networks [100].

E.2 Random graph generation

We propose to build scalable graph neural networks for molecular systems by sampling random graphs that mix short and long-range connections. We define the graph $\mathcal{G} = (\mathcal{V}, \mathcal{E})$ where $\mathcal{V}$ is the node set and $\mathcal{E}$ is the edge set. A protein can be represented as a point set $\mathbf{x} \in \mathbb{R}^{N \times 3}$. We define the process of constructing the geometric graph as $\mathcal{G}(\mathbf{x}) = (\mathcal{V}, \mathcal{E}(\mathbf{x}))$ with $|\mathcal{V}| = N$. Different from the usual graph construction scheme, the edges are generated stochastically, and $\mathcal{E}(\mathbf{x})$ describes the process. We consider schemes in which edges for each node are sampled without replacement from the set of possible edges, weighted by an edge propensity function based on spatial distance (Fig. 4). In practice, we implement this weighted sampling without replacement using Gumbel Top-k sampling [78] (Algorithm 1). Throughout this work, we use hybrid graphs which include the 20 nearest neighbors per node together with 40 randomly sampled edges under the inverse cubic edge propensity function so that both short-range and long-range interactions are sampled with appropriate rates.

E.3 Computational complexity

Under the inverse cubic attachment model, the cumulative edge propensity as a function of distance will scale as $\int_{D_{\text{min}}}^{D_{\text{max}}} \frac{1}{r^3} r^2 dr = \int_{D_{\text{min}}}^{D_{\text{max}}} \frac{1}{r} dr = \log D_{\text{max}} - \log D_{\text{min}}$. As we increase the total size (radius) of the system by D_{max}, we only need to increase the total number of edges per node by a factor of $\log D_{\text{max}}$ to keep up with the increase in total edge propensity (and to therefore ensure that increasingly distant parts of the system do not "steal" edge mass from closer parts of the system). This means that, even if we were to scale to extremely large systems such as large, solvated molecular dynamics systems with millions of atoms, the total amount of computation required for a system of N atoms will scale as $\mathcal{O}(N \log N)$. In practice, we found that for protein sizes considered in

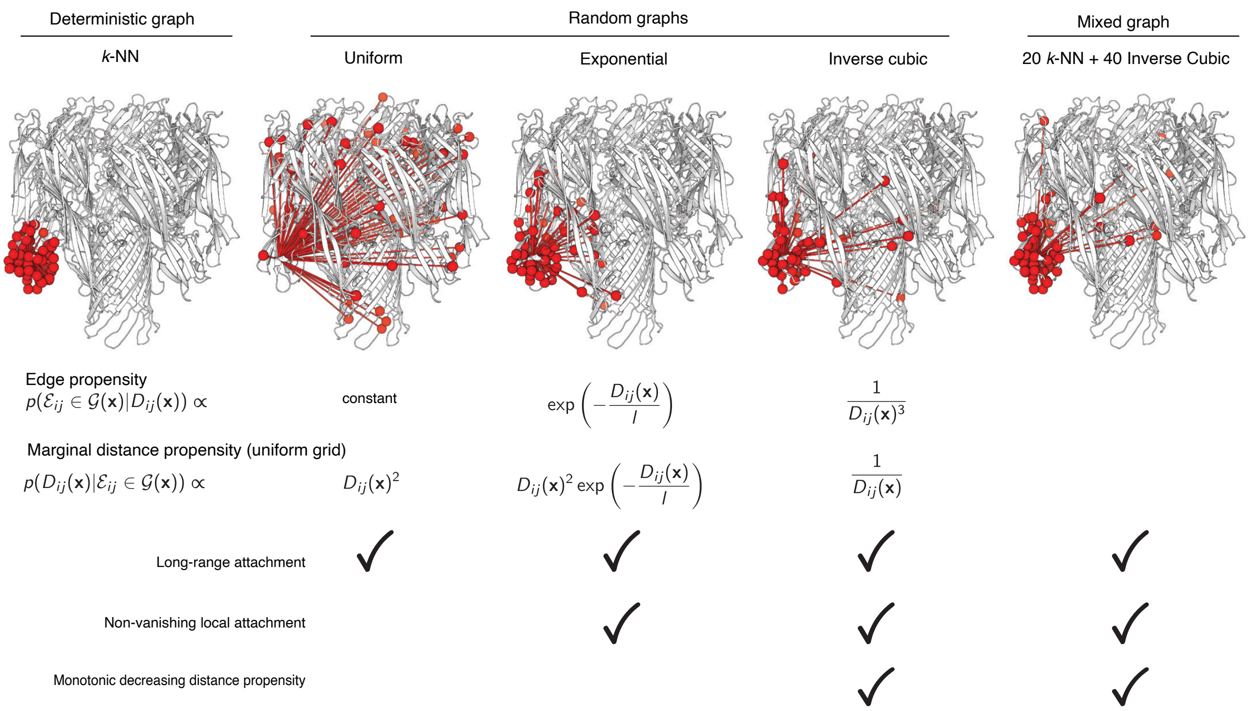

Supplementary Figure 4: **Random graphs with distance-weighted attachment efficiently capture long-range context.** Contemporary graph neural networks for learning from molecular systems achieve efficiency via spatial locality, e.g. with a spatial k-Nearest Neighbors graphs or cutoff graph (top left, $\mathcal{O}(Nk)$). We propose methods that retain this efficiency while incorporating long-range context through random edge sampling weighted by spatial distance (middle columns). We consider three different graph sampling schemes: (i) Uniformly random sampling (middle left) introduces long-range context but at the expense of vanishing local attachment. (ii) Exponential distance weighting (middle center), which can be related to dilated convolutions [100], includes both short- and long-range attachment but introduces a *typical* length scale as it induces Gamma-distributed distances. (iii) Inverse cubic distance weighting (middle right), which is the effective connectivity scaling of fast N-body methods such as Barnes-Hut [93], retains a balance of both short and long-term distances with a marginal distance propensity that gently and monotonically decays with D. In practice, we combine inverse cubic sampled random graphs with deterministic k-NN graphs to guarantee coverage of the k closest nodes while adding in long-range context (top right).

this work (complexes containing up to 4000 residues[6]) it was sufficient to simply set the number of edges per node to a constant $k = 60$, which means that the graph and associated computation will scale within this bounded size as $\mathcal{O}(N)$. This is a considerable improvement on previous approaches for global learning on protein structure such as methods based on fully connected graph neural networks [91] $\mathcal{O}(N^2)$ or Evoformer-based approaches [66] which scale as $\mathcal{O}(N^3)$. These sparse graphs also combine favorably with our method for synthesizing updated protein structures

[6]In some of our symmetry examples we find that models still generalize well to systems larger than they were trained on.

Algorithm 1 Random graph generation

Require: Inter-node distances $\{D_{ij}\}_{i,j=1}^{N}$, inverse temperature $\lambda_{\mathcal{G}}$, attachment propensity $\log p((i,j) \in \mathcal{E}(\mathbf{x})|D_{ij}) \propto e^{c(D_{ij})}$, number of edges to sample k
  for each $i \in [N]$ **do**
    for each $j \in [N]$ **do**
      $U_{ij} \sim \mathrm{Uniform}(0,1)$ ▷ Sample uniform noise per edge
      $Z_{ij} \leftarrow \lambda_{\mathcal{G}}\, c(D_{ij}) - \log\left(-\log\left(U_{ij}\right)\right)$ ▷ Perturb log probabilities with Gumbel noise
    end for
    $\mathcal{E} \leftarrow \bigcup_{i}^{N} \{(i,j)|\, j \in \mathrm{TopK}(\mathbf{Z}_i)\}$ ▷ Sample top k edges
  end for

based on predicted inter-residue geometries (Section F).

F Structure from Inter-residue Geometry Predictions

F.1 Background and motivation

Prior neural network layers for generating molecular geometries in proteins have typically relied on either (i) direct prediction of backbone internal coordinates (i.e., dihedral angles) [101, 102], which incurs accumulating errors along the chain in the form of "lever effects" that hinder performance beyond small systems; (ii) prediction of inter-residue geometries followed by offline optimization [103, 104], which builds on the successes of predicting protein structure from contacts [105] but is difficult to make end-to-end trainable; or (iii) iterative local coordinate updates based on the entire molecular system [66, 106], which can benefit from end-to-end learning but also face computational and stability challenges that may come with that.

Predicting structure as predicting constraints In principle, protein structures arise from a balance of competing intra- and inter-molecular forces. In that sense, protein structure may be regarded of as the solution to a constraint satisfaction problem with many competing potential interactions across multiple length scales. It is therefore natural to think about protein structure prediction as a so-called "Structured Prediction" problem [107], in which predictions are cast as the low-energy configurations of a learned potential function. Structured Prediction formulations of tasks often learn in a data efficient manner because it can be simpler to characterize the *constraints* in a system the the *outcomes* of those constraints. This perspective can be leveraged for molecular geometries via differentiable optimization or differentiable molecular dynamics [106, 108, 109], but these approaches are often unstable and can be cumbersome to integrate as part of a larger learning system.

F.2 Equivariant structure updates via convex optimization

Here we introduce a framework which combines the benefits of inter-residue geometry prediction and end-to-end differentiable optimization in an efficient and stable formulation based on convex

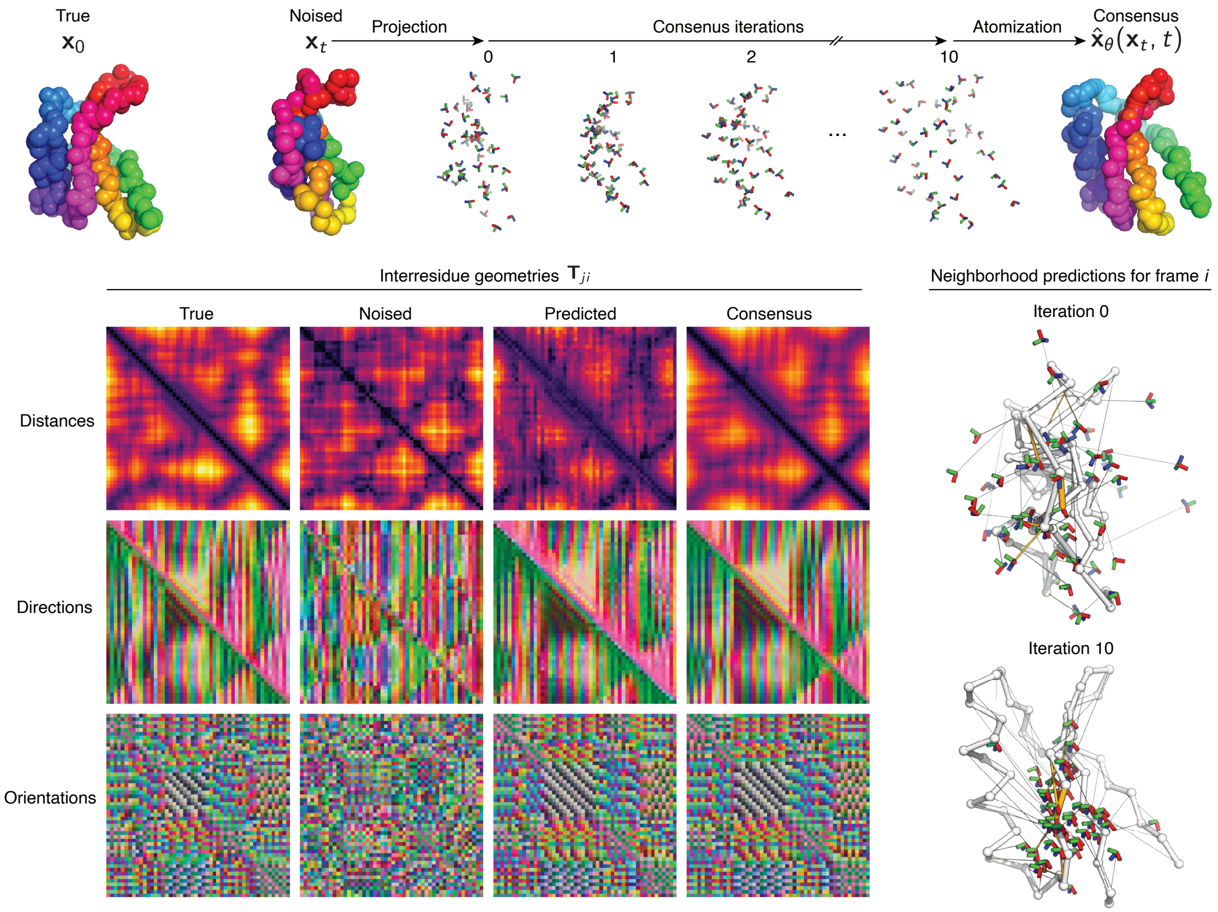

Supplementary Figure 5: **An iterative consensus algorithm resolves coordinates from predicted inter-residue geometries.** An initially noised structure (top left) is processed by a graph neural network which predicts denoised inter-residue geometries between every pair of residues on the graph (bottom left), along with confidence weights for each prediction (not shown, Appendix F). The problem of finding the optimal structure satisfying the confidence-weighted inter-residue geometry predictions forms a convex problem which can be solved by iteratively replacing residue poses with their neighborhood weighted-average consensus pose (parallel coordinate descent, top). The equilibrated poses are then imputed with relative local atom positioning also predicted by the graph neural network, forming the overall denoised structure prediction $\hat{\mathbf{x}}_\theta(\mathbf{x}_t, t)$ (top right). This entire procedure can be optimized end-to-end via automatic differentiation. As the parallel coordinate descent iterations proceed, the initially discordant geometry predictions for any given residue (right center, orange tube widths denote confidence), i.e. $\{\mathbf{T}_j \circ \hat{\mathbf{T}}_{ji}\}_{j \in N(i)}$ begin to coalesce (right bottom). The inter-residue direction and orientation visualizations (bottom left) map the normalized translation vector and rotation matrix of $\mathbf{T}_{ij}$ to RGB colors, respectively (using the last three elements of a quaternion representation of the rotation matrix).

optimization. We show how predicting pairwise inter-residue geometries as pairwise rigid translation transformations with potentially anisotropic uncertainty models induces a convex optimization problem which can be solved by a simple iteration that quickly drives towards a global consensus configuration. Throughout this section, we will build on the widely adopted approach representing

the rigid orientations of residues in proteins via coordinate reference frames [66, 67, 106].

The key idea of our update is that we ask the network to predict a set of inter-residue geometries $\mathbf{T}_{ij}$ together with confidences w_{ij} (which will initially be simple but can be extended to anisotropic uncertainty) and we then attempt to either fully or approximately solve for the consensus structure that best satisfies this set of pairwise predictions. We visualize the method in Supp. Fig. 5.

Transform preliminaries Let $\mathbf{T} = (\mathbf{O}, \mathbf{t}) \in \mathrm{SE}(3)$ be a transformation consisting of a rotation by an orthogonal matrix $\mathbf{O} \in \mathrm{SO}(3)$ followed by a translation by a vector $\mathbf{t} \in \mathbb{R}^3$. These transformations form a group with identity, inverse, and composition given by

$$\begin{aligned} \mathbf{T}_{id} &= (\mathrm{I},\, \mathbf{0}), \\ \mathbf{T}^{-1} &= \left(\mathbf{O}^{-1},\, -\mathbf{O}^{-1}\mathbf{t}\right) \\ \mathbf{T}_a \circ \mathbf{T}_b, &= (\mathbf{O}_a,\, \mathbf{t}_a) \circ (\mathbf{O}_b,\, \mathbf{t}_b) \quad = (\mathbf{O}_a\mathbf{O}_b,\, \mathbf{O}_a\mathbf{t}_b + \mathbf{t}_a)\,. \end{aligned}$$

We denote the transformation to the frame of each residue a as $\mathbf{T}_a$, and denote the relative transformation from residue a to residue b as

$$\mathbf{T}_{ab} \triangleq \mathbf{T}_a^{-1} \circ \mathbf{T}_b \; = \left(\mathbf{O}_a^{-1}\mathbf{O}_b,\, \mathbf{O}_a^{-1}(\mathbf{t}_b - \mathbf{t}_a)\right).$$

These relative transformations satisfy equations

$$\begin{aligned} \mathbf{T}_{ab} \circ \mathbf{T}_{bc} &= \mathbf{T}_{ac}, \\ \mathbf{T}_{ba} &= \mathbf{T}_{ab}^{-1}. \end{aligned}$$

Converting from backbones to transforms We represent the rigid *pose* of a residue as an absolute translation and rotation in space $\mathbf{T}_i \triangleq (\mathbf{O}_i, \mathbf{t}_i)$. We can compute these residue poses by building an orthonormal basis from three backbone coordinates at a residue i, i.e. from the set of atoms $\left\{\mathbf{x}_i^{\mathrm{N}}, \mathbf{x}_i^{\mathrm{C}\alpha}, \mathbf{x}_i^{\mathrm{C}}\right\}$. To do this, we define the vectors $\mathbf{v}_1 = \mathbf{x}_i^{\mathrm{N}} - \mathbf{x}_i^{\mathrm{C}\alpha}$ and $\mathbf{v}_2 = \mathbf{x}_i^{\mathrm{C}} - \mathbf{x}_i^{\mathrm{C}\alpha}$, and then build an orthonormal basis as

$$\mathbf{u}_1 = \frac{\mathbf{v}_1}{\|\mathbf{v}_1\|}, \qquad \mathbf{u}_2 = \frac{\mathbf{v}_2}{\|\mathbf{v}_2\|},$$

$$\mathbf{n}_1 = \mathbf{u}_1, \qquad \mathbf{n}_2 = \frac{\mathbf{n}_1 \times \mathbf{u}_2}{\|\mathbf{n}_1 \times \mathbf{u}_2\|}, \qquad \mathbf{n}_3 = \frac{\mathbf{n}_1 \times \mathbf{n}_2}{\|\mathbf{n}_1 \times \mathbf{n}_2\|},$$

which gives the final transform as

$$\mathbf{T}_i = \left([\mathbf{n}_1, \mathbf{n}_2, \mathbf{n}_3]^\intercal, \quad \mathbf{x}_i^{\mathrm{C}\alpha}\right).$$

We note that pose representations are SE(3) equivariant but are not invertible unless one forces coordinates to adopt ideal geometries, as is the choice in many structure prediction and diffusion methods [66, 67, 110, 111]. Many backbone geometries with differing internal bond lengths and angles) will give rise to the same transform $\mathbf{T}_i$ (though it is also true that many structures are not resolved at a resolution to meaningfully distinguish these degrees of freedom). Nevertheless, we can retain the benefits of both coarse transformation frames for prediction and fine all-atom granularity via a hierarchical decomposition in which we predict coarse residue-transform based inter-residue geometries along with sub-frame deviations from ideality, which can be in turn be composed (equivariantly) to yield the final structure.

Convex problem How can we define a *consensus structure* given a set of predictions of inter-residue geometries, some of which may agree and some of which may disagree? This problem is naturally formulated as an optimization problem. Given a collection of pairwise inter-residue geometry predictions and confidences $\{\mathbf{T}_{ij}, w_{ij}\}_{ij\in\mathcal{E}}$, we score a candidate structure $\{\mathbf{T}_i\}_{i=1}^N$ via a weighted loss U that measures the agreement between the current pose of each residue $\mathbf{T}_i$ and the predicted pose of the residue given each neighbor $\mathbf{T}_j$ and the predicted geometry $\mathbf{T}_{ji}$ as

$$\begin{aligned} U\left(\{\mathbf{T}_i\};\{w_{ij},\mathbf{T}_{ij}\}\right) &= \sum_{i,j} w_{ij}\left\|\mathbf{T}_i - \mathbf{T}_j \circ \mathbf{T}_{ji}\right\|^2 \\ &= \sum_{i,j} w_{ij}\left\|\mathbf{O}_i - \mathbf{O}_j\mathbf{O}_{ji}\right\|^2 + w_{ij}\left\|\mathbf{t}_i - (\mathbf{O}_j\mathbf{t}_{ji} + \mathbf{t}_j)\right\|^2. \end{aligned}$$

Note that we define a norm on the discrepancy between two Euclidean transforms $\mathbf{T}_a, \mathbf{T}_b$ as $\|\mathbf{T}_a - \mathbf{T}_b\|^2 \triangleq \|\mathbf{O}_a - \mathbf{O}_b\|^2 + \|\mathbf{t}_a - \mathbf{t}_b\|^2$. We wish to optimize each local pose $\mathbf{T}_i$ with neighbors fixed as

$$\mathbf{T}_i^\star \leftarrow \arg\min_{\mathbf{T}_i} U\left(\{\mathbf{T}_i\};\{w_{ij},\mathbf{T}_{ij}\}\right).$$

This problem of finding the local "consensus pose" for a residue $\mathbf{T}_i^\star$ given its neighborhood is a convex optimization problem, the solution to which can be realized analytically as a weighted average with projection,

$$\mathbf{T}_i^\star = \left(\mathrm{Proj}_{\mathrm{SO(3)}}\left(\sum_j p_{ij}\mathbf{O}_j\mathbf{O}_{ji}\right), \sum_j p_{ij}(\mathbf{O}_j\mathbf{t}_{ji} + \mathbf{t}_j)\right), \quad \text{where } p_{ij} = \frac{w_{ij}}{\sum_j w_{ij}}$$

and the projection operator may be implemented via SVD as in the Kabsch algorithm [68] for optimal RMSD superposition. If we iterate this update multiple times to all positions in parallel, we obtain a parallel coordinate descent algorithm which can rapidly equilibrate towards a global consensus (Supplementary Figure 5).

Two-parameter uncertainty models The above iteration leverages an *isotropic* uncertainty model in which the error model for the translational component is spherically symmetric and coupled to the uncertainty in the rotational component of the transform. We may also consider *anisotropic* uncertainty models where these confidences are decoupled. In the first of these, we decouple the weight w_{ij} into separate factors for the translational and rotational components of uncertainty as w_{ij}^{T} and $w_{ij}^{\angle}$, respectively. The overall error model being optimized is then

$$U\left(\{\mathbf{T}_i\};\{w_{ij},\mathbf{T}_{ij}\}\right) = \sum_{i,j} w_{ij}^{\angle}\left\|\mathbf{O}_i - \mathbf{O}_j\mathbf{O}_{ji}\right\|^2 + w_{ij}^{\mathsf{T}}\left\|\mathbf{t}_i - (\mathbf{O}_j\mathbf{t}_{ji} + \mathbf{t}_j)\right\|^2.$$

This makes intuitive sense when the network will possess high confidence about the relative position of another residue but not its relative orientation, and may still be solved analytically by weighted averaging with projection.

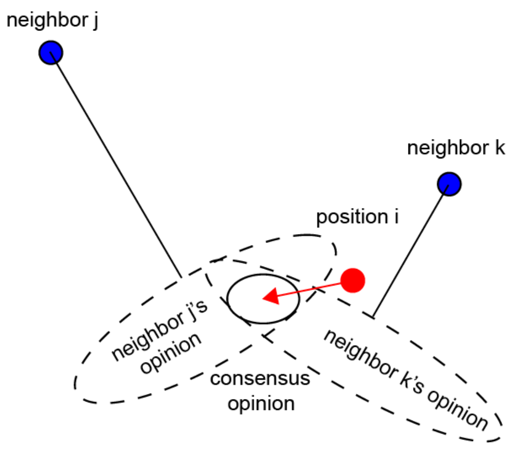

Supplementary Figure 6: **Anisotropic confidence models capture asymmetric uncertainty in predicted inter-residue geometries.** Position i is forced towards its consensus position which is the mean of a fusion of anisotropic Gaussians. Here we visualize the covariance ellipses of component the Gaussians, i.e. the inverses of the precision matrices predicted by our network.

Three-parameter uncertainty models In a more sophisticated form of anisotropic uncertainty, we extend this framework to ellipsoidal error models bespoke to each ij, while retaining a closed-form iteration update using approaches from sensor fusion. We parameterized this anisotropic error model by separating this precision term w into three components: $w_{ij}^{\angle}$ for rotational precision and two components for position: $w_{ij}^{\parallel}$ for radial distance precision, and $w_{ij}^{\perp}$ for lateral precision. The radial and lateral precision terms are each eigenvalues of the full 3x3 precision matrix $\mathbf{P}_{ij}$ for translation errors (i.e., inverse covariance matrix under a multivariate normal error model):

$$\mathbf{P}_{ij} = w_{ij}^{\parallel}\pi_{ij} + w_{ij}^{\perp}(I - \pi_{ij}), \qquad \pi_{ij} = \frac{(\mathbf{O}_j\mathbf{t}_{ji})(\mathbf{O}_j\mathbf{t}_{ji})^\intercal}{(\mathbf{O}_j\mathbf{t}_{ji})^\intercal(\mathbf{O}_j\mathbf{t}_{ji})}$$

where π_{ij} is the projection matrix onto the radial direction from $\mathbf{t}_j$ to the predicted position $\mathbf{O}_j\mathbf{t}_{ji} + \mathbf{t}_j$ of $\mathbf{t}_i$, and $\mathrm{I} - \pi_{ij}$ is the projection matrix onto lateral translations (spanned by the remaining two eigenvectors). These anisotropic terms finally combine as

$$\begin{aligned} U\left(\{\mathbf{T}_i\}; \{w_{ij}, \mathbf{T}_{ij}\}\right) &= \sum_{i,j} \left(\mathbf{O}_j\mathbf{t}_{ji} + \mathbf{t}_j - \mathbf{t}_i\right)^\intercal \mathbf{P}_{ij} \left(\mathbf{O}_j\mathbf{t}_{ji} + \mathbf{t}_j - \mathbf{t}_i\right) + w_{ij}^{\angle} \left\|\mathbf{O}_i - \mathbf{O}_j\mathbf{O}_{ji}\right\|^2 \\ &= \sum_{i,j} w_{ij}^{\parallel} \left\|\pi_{ij}(\mathbf{O}_j\mathbf{t}_{ji} + \mathbf{t}_j - \mathbf{t}_i)\right\|^2 \\ &\qquad + w_{ij}^{\perp} \left\|(I - \pi_{ij})(\mathbf{O}_j\mathbf{t}_{ji} + \mathbf{t}_j - \mathbf{t}_i)\right\|^2 + w_{ij}^{\angle} \left\|\mathbf{O}_i - \mathbf{O}_j\mathbf{O}_j\right\|^2. \end{aligned}$$

As we expect that the radial precision always exceeds the lateral precision, our neural predictor outputs three positive parameters ($w^{\perp}$, $w^{\parallel} - w^{\perp}$, $w^{\angle}$). Whereas the isotropic objective above is solved by weighted averaging, the anisotropic translation part of this objective is solved by a standard Gaussian product operation from sensor fusion [112],

$$\mathbf{t}_i^{\star} = \mathbf{t}_i + \left(\sum_j \mathbf{P}_{ij}\right)^{-1} \sum_j \mathbf{P}_{ij}(\mathbf{O}_j\mathbf{t}_{ji} + \mathbf{t}_j - \mathbf{t}_i).$$

We illustrate this anisotropic Gaussian fusion operation in Supplementary Figure 6.

Algorithm 2 Equivariant Consensus Structure from Inter-residue Geometries

Require: $\{\mathbf{T}_{ij}, w_{ij}\}_{ij\in\mathcal{E}_{\mathcal{G}}(\mathbf{x})}$ ▷ Predicted inter-residue geometries and confidence weights
Require: $\{\mathbf{t}_{i\mathrm{N}}, \mathbf{t}_{i\mathrm{C}_\alpha}, \mathbf{t}_{i\mathrm{C}}, \mathbf{t}_{i\mathrm{O}}\}_{i=1}^{N}$ ▷ Predicted local atomic geometries
Require: $\{\mathbf{T}_i\}_{i=1}^{N}$ ▷ Initial residue poses
Require: M ▷ Number of parallel coordinate descent iterations

$\forall i,j, \quad p_{ij} \leftarrow \frac{w_{ij}}{\sum_j w_{ij}}$ ▷ Compute confidence weights
for each $m \in 1 \dots M$ **do**
$\quad \forall i \quad \mathbf{T}_i \leftarrow \left(\mathrm{Proj}_{\mathrm{SO}(3)}\left(\sum_j p_{ij}\mathbf{O}_j\mathbf{O}_{ji}\right), \ \sum_j p_{ij}(\mathbf{O}_j\mathbf{t}_{ji} + \mathbf{t}_j)\right)$ ▷ Locally optimize poses
end for
for each ATOM $\in \{\mathrm{N}, \mathrm{C}_\alpha, \mathrm{C}, \mathrm{O}\}$ **do**
$\quad \forall i \quad (\mathbf{0}, \ \mathbf{x}_i^{\mathrm{ATOM}}) \leftarrow \mathbf{T}_i \circ (\mathbf{0}, \mathbf{t}_{i\mathrm{ATOM}})$ ▷ Build atoms
end for
return x ▷ Output atomic backbone geometry

F.3 Equivariant prediction of backbone atoms

The parallel coordinate descent procedure optimizes residue poses $\{\mathbf{T}_i\}$ but our diffusion model (Supplementary Appendix D) requires unconstrained *atomic* prediction of all backbone heavy atoms. We can straightforwardly augment the above predictions in an equivariant manner by predicting *local coordinates* $\{\mathbf{t}_{i\mathrm{N}}, \mathbf{t}_{i\mathrm{C}_\alpha}, \mathbf{t}_{i\mathrm{C}}, \mathbf{t}_{i\mathrm{O}}\}_{i=1}^{N}$ for each atom position relative to the parent residue pose from graph node embeddings. To simplify learning, we parameterize these predictions as residual updates from the ideal backbone geometry positions. To build the final atomic structure, we simply right-compose these local coordinate predictions $\mathbf{t}_{i\mathrm{ATOM}}$ with each parent pose $\mathbf{T}_i$ as

$$(\mathbf{0}, \ \mathbf{x}_i^{\mathrm{ATOM}}) = \mathbf{T}_i \circ (\ \mathbf{0}, \ \mathbf{t}_{i\mathrm{ATOM}}).$$

We schematize this combined method in Algorithm 2. These predictions will be equivariant because they are right-composed with the parent residue poses, which are equivariant because they are built from relative, equivariant projection from the initial geometry $\mathbf{x}_t$.

F.4 Time-dependent post-prediction scaling

It has been helpful in prior diffusion modeling works to parameterize the denoising network output in a way that can behave as an identity function for low noise levels early in training [65]. We found this to be helpful as well and parameterized the final prediction as

$$\hat{\mathbf{x}}_\theta(\mathbf{x}_t, t) = \eta_t \tilde{\mathbf{x}}_\theta(\mathbf{x}_t, t) + (1 - \eta_t)\mathbf{x}_t,$$

where $\tilde{\mathbf{x}}_\theta(\mathbf{x}_t, t)$ is the output from the inter-residue consensus and the time dependent 'gate' η_t was set in two ways:

- **Output Scaling A** Set η_t to scale as $\sqrt{1-\text{SSNR}_t}$ with a learnable offset by parameterizing as $\eta_t = S\left(S^{-1}(\text{SSNR}_t) + u_t^\theta\right)$ where $S(\cdot)$ is the sigmoid function and u_t^θ is parameterized by a small MLP.
- **Output Scaling B** Set η_t to scale as $\sqrt{1-\text{SSNR}_t}$ with a learnable offset by parameterizing as $\eta_t = 1 - \left(1 - S\left(S^{-1}(\text{SSNR}_t) + u_t^\theta\right)\right)\mathbb{I}(\text{SSNR}_t > \texttt{CUTOFF})$ where $S(\cdot)$ is the sigmoid function, u_t^θ is parameterized by a small MLP, and $\texttt{CUTOFF} = 0.99$. This is similar to the previous scaling but almost always disabled except for the highest values of the signal-to-noise ratio.

G Chroma Architecture

Chroma builds a joint distribution of the sequence and and all-atom structure of protein complexes via the factorization

$$\log p(\mathbf{x},\mathbf{s},\chi) = \underbrace{\log p(\mathbf{x})}_{\text{backbone likelihood}} + \underbrace{\log p(\mathbf{s}|\mathbf{x})}_{\text{sequence likelihood}} + \underbrace{\log p(\chi|\mathbf{x},\mathbf{s})}_{\text{side-chain likelihood}} .$$

We model these likelihoods with two networks: a *backbone network* trained as a diffusion model to model $p(\mathbf{x})$ and a *design network* which models sequence and side chain chains conditioned on backbone structure. Both networks are based on a common graph neural network architecture, and we visualize the overall system in Supplementary Figure 7. We list important hyperparameters for the backbone network in Supplementary Table 2 and for the design network in Supplementary Table 3. We design sequences by extending the framework of [95] and factorizing joint rotamer states autoregressively in space, and then locally autoregressively per side-chain χ angle within a residue as done in [113]. For the sequence decoder, we explore both autoregressive decoders of sequence (pictured in Supplementary Figure 7) and conditional random field decoding of sequence, which was also explored in concurrent work [114].

G.1 Graph neural networks for protein structure

Graph Neural Network All of our neural network models are based on graph neural networks [94] that reason over 3D structures of proteins by transforming them into attributed graphs built from rigid transformation invariant (SE(3)-invariant) features. The building block from which these models are built is presented in Algorithm 3. This approach has been pursued in several prior works for sequence design [95, 115, 116] and our primary architectural innovations to extend this to all-atom protein complex generative modeling are two-fold:

- We propose *random graph neural networks* that add in long-range connections and reasoning while preserving sub-quadratic computational complexity (Supplementary Appendix E)
- We introduce a method for efficiently and differentiably generating protein structures from predicted inter-residue geometries based on parallel coordinate descent (Supplementary Appendix F)

Featurization We represent protein structure as an attributed graph with node and edge embeddings computed as SE(3)-invariant features of the input backbone. For the node features we encode

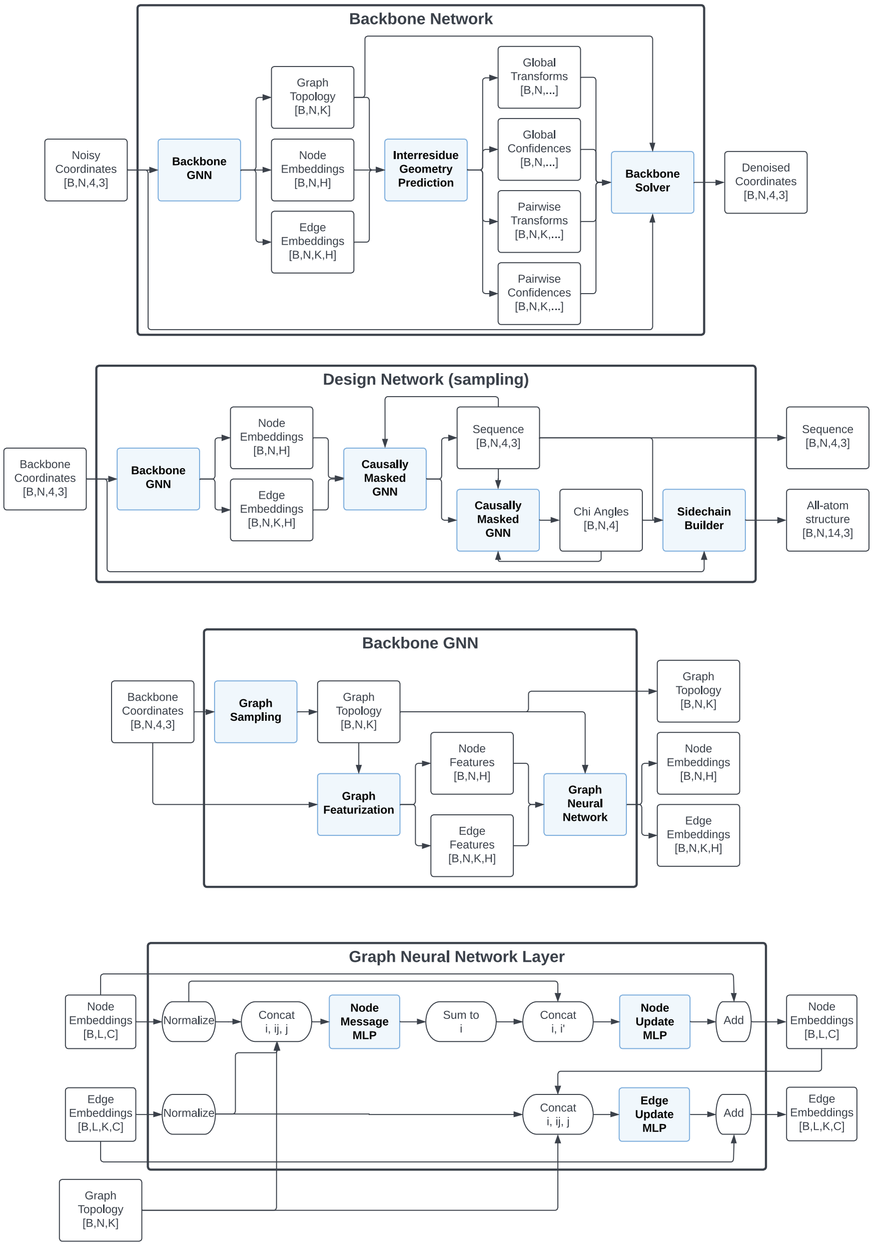

Supplementary Figure 7: **Chroma is composed of graph neural networks for backbone denoising and sidechain design.**

local geometry via bond lengths and the backbone dihedral angles lifted to the unit circle via paired sin and cos featurization. We encode the inter-residue geometries between each pair of nodes (i, j) with the following edge features:

- **Inter-atomic distances**: Distances between all atoms at residues i and j, i.e. the 8×8 distance matrix, lifted into a radial basis via $f_i(D_{ab}) \triangleq e^{(D_{ab}-\mu_i)^2/\sigma_i^2}$ for $1 \leq i \leq 20$ and centers μ_i spaced linearly on $[0, 20]$ and $\sigma_i = 1$.

Algorithm 3 Graph Neural Network Layer

Require: $\mathbf{n}_i$, $\mathbf{e}_{ij}$ ▷ Node and edge embeddings with shapes (B,N,C) and (B,N,K,C)
Require: $\mathcal{N}(i)$ ▷ Graph topology specifying neighbors of each residue
for each $i \in [L]$ **do**
  $\tilde{\mathbf{n}}_i \leftarrow \text{NodeLayerNorm}(\mathbf{n}_i)$
  $\tilde{\mathbf{e}}_{ij} \leftarrow \text{EdgeLayerNorm}(\mathbf{e}_{ij})$

  $\mathbf{p}_{ij} \leftarrow \text{Concatenate}_{j\in\mathcal{N}(i)}(\tilde{\mathbf{n}}_i, \tilde{\mathbf{n}}_j, \tilde{\mathbf{e}_{ij}})$
  $\mathbf{m}_{ij} \leftarrow \text{MessageMLP}(\mathbf{p}_{ij})$
  $\mathbf{m}_i \leftarrow \text{Aggregate}_j(\mathbf{m}_{ij})$

  $\mathbf{p}_i \leftarrow \text{Concatenate}(\tilde{\mathbf{n}}_i, \mathbf{m}_i)$
  $\mathbf{n}_i \leftarrow \mathbf{n}_i + \text{NodeUpdateMLP}\ (\mathbf{p}_i)$
end for
for each $i \in [N]$ **do**
  for each $ij \in \mathcal{N}(i)$ **do**
    $\mathbf{p}_{ij} \leftarrow \text{Concatenate}_{j\in\mathcal{N}(i)}(\mathbf{n}_i, \mathbf{n}_j, \tilde{\mathbf{e}}_{ij})$
    $\mathbf{e}_{ij} = \mathbf{e}_{ij} + \text{EdgeUpdateMLP}(\mathbf{p}_{ij})$
  end for
end for
return $\mathbf{n}_i, \mathbf{e}_{ij}$ ▷ Updated node and edge embeddings

- **Inter-atomic directions**: The unit vector from $\mathbf{x}_i^{C\alpha}$ at residue i to atom b in residue j, concatenated over all atoms $b \in \{N, C, C_\alpha, O\}$ in j.
- **Chain distance**: Tuple encoding (1) chain distance featurized as $(\log(|i-j|+1)$ for residues i, j lying along the same chain, else 0, and (2) a binary flag indicating if i and j are in different polymer chains.
- **Transform features**: For two frames $\mathbf{T}_a = (\mathbf{O}_a, \mathbf{t}_a)$ and $\mathbf{T}_b = (\mathbf{O}_b, \mathbf{t}_b)$ let $\mathbf{T}_{a\to b}$ denote the transform that maps coordinates in frame T_a to coordinates in frame T_b. For each residue i, define two frames, a local frame T_i and chain frame $T_{c(i)}$. The chain frame is obtained by using the Grahm-schmidt construction to pass to an orthonormal set of vectors $[n_1, n_2, n_3]$ starting with $N - C_\alpha$ and $C_\alpha - C$ vectors averaged across the chain. The following transforms are computed: $\mathbf{T}_{i\to j}, \mathbf{T}_{i\to c(j)}, \mathbf{T}_{c(i)\to c(j)}$. For each of these transforms, the features $\log(\|\mathbf{t}\|), \mathbf{t}$, and quaternion$(\mathbf{O})$ are computed and concatenated.

Equivariance Because the input features are SE(3) invariant and the update layer (see section F for details) is SE(3) equivariant, the ChromaBackbone network is SE(3) equivariant and the ChromaDesign network is SE(3) invariant.

**Category**	**Hyperparameter**	**Value in** `ChromaBackbone v0`	**Value in** `ChromaBackbone v1`
Diffusion Process	Covariance Model	Globular Monomer	Globular Complex
	Noise Schedule	Log-linear SNR (-7,13.5) [62]	Log-linear SNR (-7,13.5)
Graph Features	Node Features	Internal Coordinates	Internal Coordinates
	Edge Features	Atom distances, Atom directions, Chain distances, Transforms	Atom distances, Atom directions, Chain distances, Transforms
	Edges per Node, k	60	60
	Number of Nearest Neighbor Edges	20	20
	Number of Random Edges	40	40
	Random Edge Type	Inverse Cubic	Inverse Cubic
Graph Neural Network	Number of GNN layers	12	12
	Node Embedding Dimension	512	512
	Edge Embedding Dimension	256	256
	Node MLP Dimension	512	512
	Edge MLP Dimension	128	128
	Dropout p	0.1	0.1
Denoising Solver	Inter-residue Parameterization	Direct T_{ij} prediction	Update from $T_{ij}(\mathbf{x}_t)$
	Uncertainty Model	Isotropic (1-parameter)	Decoupled (2-parameter)
	Number of Iterations	3	10
	Post-Process Scaling	A	B
Loss Function	Likelihood Loss	ELBO	ELBO
	Auxilliary Losses	ELBO-weighted MSE	$\mathcal{D}_{\text{global}}$, $\mathcal{D}_{\text{fragment}}$, D_{ij} SE, $\hat{T}_{ij}$ SE
Total Number of Parameters		18.6M	18.6M
Total Number of Training Steps		1.6M	1.8M

Supplementary Table 2: **ChromaBackbone Hyperparameters.**

G.2 ChromaBackbone

The backbone network parameterizes an estimate of the optimal denoiser $\hat{\mathbf{x}}_\theta(\mathbf{x}_t, t)$ and combines a graph neural network described in the previous section with the inter-residue consensus layer described in Appendix F. We trained two major versions used throughout this work (aside from the ablation study), with hyperparameters described in Supplementary Table 2.

G.3 ChromaDesign

The design network parameterizes the conditional distribution of sequence and χ angles given structure $p_\theta(\mathbf{s}, \chi|\mathbf{x})$ by combining the graph neural network encoder described in the previous section with sequence and side-chain decoding layers. To enable robust sequence prediction and the potential for use as a conditioner, we train ChromaDesign with *diffusion augmentation*, i.e. we predict sequence and chi angles given a noisy structure $\mathbf{x}_t$ and a time t as $p_\theta(\mathbf{s}, \chi|\mathbf{x}_t, t)$. We consider both a Potts decoder architecture which admits compact and fast constrained sampling with conditioning or auxiliary objectives, as well as an autoregressive decoder architecture for capturing higher-order dependencies in the sequence and modeling sidechain conformations given sequence and structure.

G.4 Related Work

Generative models based on diffusion There has been broad interest in generative models of protein structure, and diffusion models have seen particularly rapid adoption towards the problem. This has included diffusion models for protein monomers represented as coarse C_α coordinates [91], internal coordinates [102], and rigid frames [117, 118], as well as for protein complexes represented as rigid frames [119]. Beyond backbone-only models, there have also been joint gen-

Category	Hyperparameter	Value in `ChromaDesign Potts`	Value in `ChromaDesign Multi`
Diffusion Process	Covariance Model	None	Globular Complex
	Noise Schedule	N/A	Log-linear SNR (-7,13.5)
Graph Features	Node Features	Internal Coordinates	Internal Coordinates
	Edge Features	Atom distances, Atom directions, Chain distances	Atom distances, Atom directions, Chain distances, Transforms
	Number of edges per node, k	40	60
	Number of kNN edges	40	60
	Number of inverse cubic edges	0	0
Graph Neural Network	Number of GNN layers	6	10
	Node embedding dimension	128	128
	Edge embedding dimension	128	128
	Node MLP hidden dimension	512	512
	Edge MLP hidden dimension	128	128
	Dropout p	0.1	0.1
	Label smoothing	0.1	0.1
Sequence Decoder	Type	Potts model, First order	Potts model, First order, Autoregressive
Sidechain Decoder	Type	N/A	Autoregressive
Chi decoder	Number of χ bins	N/A	36
Total Number of Parameters		3.9M	13.8M

Supplementary Table 3: **ChromaDesign Hyperparameters.**

erative frameworks which model all-atom protein structure with mixed diffusions over backbone, sequence, and side-chain degrees of freedom [67, 120]. Furthermore, we are beginning to see experimental validation of diffusion-based models for structure and/or sequence [119, 121] and for partially joint sequence-structure models that combine a language model prior with deterministic structure prediction [122].

One common theme of generative models for proteins thus far has been *dense reasoning* in which, to generate complex molecular systems like proteins or protein complexes, learning frameworks must reason over all possible pairs of interactions in a system. While these approaches will, by construction, always be able to perform as well as sparsely-connected approaches, Chroma provides evidence that simpler frameworks based entirely on sparse reasoning and knowledge of domain structure can be sufficient to build a complete joint model for complex multi-molecular systems such as protein complexes. We anticipate that this sufficiency argument may be important for two reasons: Firstly, subquadratic scaling $\mathcal{O}(N\log N)$ of algorithms has been a foundational paradigm for modeling the physical world from molecular [123] to cosmological systems [93]. Second, and perhaps more speculatively, it may be argued that, given multiple algorithms with similar performance, simpler and more computationally efficient algorithms are more likely to be robust and to generalize [124].

Potts Decoder In the Potts formulation of the ChromaDesign network, we factorize the conditional distribution of sequence as a conditional Potts model, a type of conditional random field [56], with likelihood

$$p_\theta(\mathbf{s}|\mathbf{x}) = \frac{1}{Z(\mathbf{x},\boldsymbol{\theta})} \exp\left(-\sum_i \mathbf{h}_i(s_i;\mathbf{x}) - \sum_{i<j} \mathbf{J}_{ij}(s_i,s_j;\mathbf{x})\right),$$

where the conditional fields $\mathbf{h}_i(s_i;\mathbf{x})$ and conditional couplings $\mathbf{J}_{ij}(s_i,s_j;\mathbf{x})$ are parameterized by the node and edge embeddings of the graph neural network, respectively. We train the Potts decoder with diffusion augmentation to predict sequence given a noisy structures $\mathbf{x}_t$ and a time t

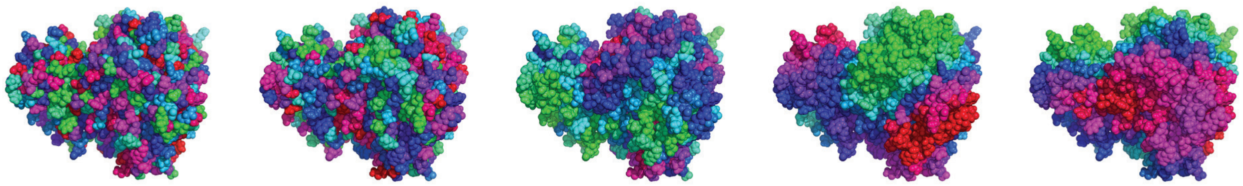

Supplementary Figure 8: **Randomized autoregression orders with spatial smoothing vary the typical spatial context for sequence modeling.** Uniformly random autoregression orders (left) are spatially uncorrelated and as a result induce highly disordered contexts which are unlike the conditionals used during substructure design tasks. Uniformly random orderings can be transformed into spatially coherent orderings by applying tunable spatial smoothing to the original ordering values, followed by ARGSORT. We apply spatial smoothing with by local neighborhood averaging on a k-NN graph. Intermediate strengths of spatial smoothing produce locally coherent orderings (middle), while strong smoothing producing crystallization-like, coherent traversals of the entire structure (right). We uniformly sample $\mu_{\text{smooth}} \sim \mathcal{U}(0,1)$ at training time.

as

$$p_\theta(\mathbf{s}|\mathbf{x}_t,t) = \frac{1}{Z(\mathbf{x}_t,t,\boldsymbol{\theta})} \exp\left(-\sum_i \mathbf{h}_i(s_i;\mathbf{x}_t,t) - \sum_{i<j} \mathbf{J}_{ij}(s_i,s_j;\mathbf{x}_t,t)\right).$$

Advantages of the Potts decoders include that they admit fast global optimization even when combined with conditioning constraints or co-objectives via as simulated annealing or gradient-based samplers ([125]) and that they have been highly validated experimentally as sufficient generative models for generating diverse and functional samples when trained on protein families. A disadvantage is that they are limited beyond modeling second-order effects and require many more iterations of Monte Carlo sampling than one-shot ancestral sampling of autoregressive models.

Autoregressive Decoder We build on the theme of using graph neural networks with autoregressive decoders for sequence design [106, 115, 116] and factorize the conditional distribution of sequence given structure autoregressively as

$$p_\theta(\mathbf{s}|\mathbf{x}) = \prod_i p_\theta(s_{\pi_i}|s_{\pi_{i-1}},\ldots,s_{\pi_1},\mathbf{x}),$$

where $\boldsymbol{\pi}$ is a permutation specifying an decoding order for the sequence. We sample random traversals with a randomly sampled amount of spatial correlation, as shown in Supplementary Figure 8, that may better align with conditionals encountered at design time and enable more spatially structured decompositions that mix more effectively in causally-masked message passing. We train the autoregressive decoder with diffusion augmentation to predict sequence given a noisy structure $\mathbf{x}_t$ and a time t as

$$p_\theta(\mathbf{s}|\mathbf{x}_t,t) = \prod_i p_\theta(s_{\pi_i}|s_{\pi_{i-1}},\ldots,s_{\pi_1},\mathbf{x}_t,t).$$

Sidechain Decoding We model the conditional distribution of side chain conformations given sequence and backbone structure by modeling theχ angles with an autoregressive decomposition

as

$$p_\theta(\chi|\mathbf{s},\mathbf{x}) = \prod_i p_\theta(\chi_{\pi_i}|\chi_{\pi_{i-1}},\dots,\chi_{\pi_1},\mathbf{s},\mathbf{x}),$$

where the conditional joint distributions $p_\theta(\chi_{\pi_i}|\chi_{\pi_{i-1}},\dots,\chi_{\pi_1},\mathbf{s},\mathbf{x})$ at each residue locally factorize as up to four discrete, sequential decisions as in [113]. We model model these with empirical histograms for each angular degree of freedom binned at 36 bins, i.e. with 10° angular resolution. During sampling, we convert the discrete binned probability masses into linearly interpolated probability densities, giving a distribution over angles that is fully supported on the hyper-torus. We train the sidechain decoder with diffusion augmentation to predict chi angles from a sequence $\mathbf{s}$, a noisy structure $\mathbf{x}_t$, and a time t as

$$p_\theta(\chi|\mathbf{s},\mathbf{x}_t,t) = \prod_i p_\theta(\chi_{\pi_i}|\chi_{\pi_{i-1}},\dots,\chi_{\pi_1},\mathbf{s},\mathbf{x}_t,t).$$

H Training

H.1 Dataset

Processing We constructed our training dataset from a filtered version of the Protein Data Bank [126] queried on 2022-03-20. We filtered for non-membrane X-ray protein structures with a resolution of 2.6 $\AA$ or less and reduced redundancy by clustering homologous sequences with USEARCH [127] at 50% sequence identity and selecting one sequence per cluster. Additionally, because antibody folds are highly diverse in both sequence and structure and highly releveant to biotherapeutic development, we enriched our redundancy-reduced set 1726 non-redundant antibodies that were clustered at a 90% sequence identity cutoff. This yielded 28,819 complex structures which were transformed into their biological assemblies by favouring assembly ID where the authors and software agreed, followed by authors and finally by software only. Missing side-chain atoms were added with pyRosetta [128].

Splitting We split the data set with into 80%/10%/10% train, validation and test splits by minimizing the sequence similarity overlap using entries of PFAM family ID, PFAM clan ID [129], UniProt ID [130] and MMSEQ2 cluster ID at a 30% threshold [131]. To accomplish this, we construct a similarity graph in which each PDB entry is represented by a node connected to other entries that share at least one identical annotation. Connected sub-graphs are identified and broken apart by iteratively deleting the most central annotations until there are 50 or fewer connected nodes. Using this procedure, we increased the fraction of test annotations with no representation in the training set (versus a random split) from 0.1% to 9% for Pfam clan, from 10% to 59% for Pfam family, from 50% to 82% for MMSEQ30 cluster, and from 70% to 89% for Uniprot ID.

H.2 Optimization

Backbone network We trained `ChromaBackbone v1` on 8 Tesla V100-SXM2-16GB using the Adam optimizer [132] to optimize a sum of the regularized ELBO loss (Supplementary Appendix B) and an unweighted sum of the losses described in (Supplementary Appendix B.4). We linearly annealed the learning rate from 0. to 2×10^{-4} over the first 10,000 steps and trained for a total of

Experiments	Sample type	T	λ	ψ	Backbone Model	Design Model	Complexity Penalty
Computational	Unconditional	500	10	2	Multiple	Multiple	LCP
	Ablation Study	500	10	2	Multiple	`ChromaDesign Potts`	LCP
	Substructure	400	$8^{\diamond}$	$2^{\bowtie}$	`ChromaBackbone v1`	`ChromaDesign Multi`	LCP
	Symmetry	$500^{\star}$	8	$8^{\bowtie}$	`ChromaBackbone v1`	`ChromaDesign Multi`	LCP
	Shape	$3000^{\star}$	10	2,3	`ChromaBackbone v1`	`ChromaDesign Multi`	LCP
	Classification	2000	10	2	`ChromaBackbone v1`	`ChromaDesign Multi`	LCP
	Language	500	10	2	`ChromaBackbone v1`	`ChromaDesign Multi`	LCP
Wet Lab	Unconditional I	1000	10	2	`ChromaBackbone v0.4999`	`ChromaDesign Potts`	UP
	Unconditional II	2000	10	0.1	`ChromaBackbone v0.4998`	`ChromaDesign Potts`	LCE
	Conditional I	Multiple	10	2	`ChromaBackbone v0.4999`	`ChromaDesign Potts`	UP
	Conditional II	2000	10	0.9	`ChromaBackbone v0.4999`	`ChromaDesign Potts`	UP

Supplementary Table 4: **Sampling hyperparameters.** We review all configurations for sampling used across both *in silico* and wet lab experiments. The ($\star$) symbol in the T column indicates integrating with an improved Euler-like integrator. The ($\diamond$) symbol in the λ_0 column corresponds to keeping inverse temperature fixed (isothermal sampling) throughout integration instead of the annealing presented in Appendix C. The ($\bowtie$) symbol in the ψ column indicates that the annealed Langevin dynamics is used instead of the reverse diffusion SDE.

1,796,493 steps. Due to the linear scaling memory footprint of our model, we dynamically pack complexes into minibatches to approach a target number of residues per batch which was 4,000 residues per GPU and thus 32,000 residues per step. We estimated the final model parameters with an exponential moving average (EMA) of per-step parameter values with a decay factor of 0.999 [133]. We trained `ChromaBackbone v0` similarly but without EMA estimation, and we refer to checkpoints from specific epochs of training as `ChromaBackbone v0.XXXX` where `XXXX` is the epoch number.

Design network We trained `ChromaDesign Potts` and `ChromaDesign Multi` with the same framework as the backbone networks but a few specific modifications: We trained `ChromaDesign Potts` in a time-invariant manner on uncorrupted samples $\mathbf{x}_0$ to optimize a pairwise composite log-likelihood approximation of the Potts log-likelihood [134], averaged to nats per residue. We trained `ChromaDesign Multi` in a time-aware manner on samples $\mathbf{x}_t$ from the diffusion process. As a training objective we used the sum of the pairwise composite log likelihood loss for the Potts decoder (residue-averaged) along with the average per residue log likelihood losses for the three other decoder 'heads': the autoregressive sequence decoder, the marginal sequence decoder (which independently predicts each residue identity s_i from structure $\mathbf{x}_t$), and the autoregressive side chain predictor.

I Sampling

I.1 Sampling backbones

We sampled proteins from Chroma by first generating backbone structures and then designing sequences conditioned on the backbone. Unless otherwise specified, we generated structures by integrating the reverse diffusion SDE (appendix Q.2) with $\lambda_0 = 10$. For constrained conditioners (e.g. substructure, symmetry), we opt for annealed Langevin dynamics (see appendix M.2 for more details).

I.2 Sampling sequences

For all design tasks, we experimented with both autoregressive and Potts-based sequence sampling but ultimately decided on Potts-based samples as they facilitated more thorough global optimization with sequence complexities penalties. It has been widely observed that low temperature sampling from likelihood-based models often biases towards low complexity sequences [77], and we also have observed this phenomenon to happen on occasion during conditional sequence design. While it is not impossible that low-complexity sequences may still fold *in silico* and *in vivo*, we wish to be able to control the level of sequence complexity at the time of design.

We control sequence complexity via penalized Markov-Chain Monte Carlo (MCMC) with our conditional Potts models. We define the total energy as the sum of the conditional Potts energy plus an optional sequence complexity penalty, and sample sequences using 10 independent cycles of simulated annealing Monte Carlo (MC), each with $4000 \cdot N$ steps, where N is the length of the protein.

Robust design The `ChromaDesign Multi` network is trained with *diffusion augmentation* such that it can predict sequence given structures as though the structures arose from the diffusion ensemble at time t. This time conditioning serves as a kind of amortized tunable amount of data augmentation during design. Throughout this work, we sample sequences with $t = 0$, but note that $t > 0$ is useful for increasing sequence design robustness.

I.2.1 Unique Permutations (UP) restraint

The first restraint type that we used is based on the number of unique permutations of the designed sequence, Ω [135]:

$$\Omega = \log\left(\frac{L!}{\prod_{i=1}^{N} n_i!}\right),$$

where N is the number of different amino acids in the sequence, n_i is the number of occurrences of amino acid type i. This restraint simply applied a linear penalty when Ω dropped below a desired threshold $\hat{\Omega}$:

$$C_1 = \begin{cases} \hat{\Omega} - \Omega, & \text{if } \Omega < \hat{\Omega} \\ 0, & \text{otherwise} \end{cases}$$

We chose $\hat{\Omega}$ to be one standard deviation below the empirical mean for PDB sequences of length L. Specifically, we found that the empirical mean and standard deviation among PDB sequences to depend on L as $2.855 \cdot \left(1 + 9.927 \cdot L^{-0.894}\right)^{-1}$ and $0.287 \cdot \left(1 + 0.0447 \cdot L^{0.810}\right)^{-1}$, respectively.

I.2.2 Local Composition Entropy (LCE) restraint

Our second sequence complexity restraint was based on the mean sequence entropy over all local windows:

$$C_2 = \frac{L}{L - w + 1} \sum_{i=1}^{L-w+1} S_i,$$

where w is window length (we used $w = 30$ throughout this study) and S_i is the entropy of the i-th window.

I.2.3 Local Composition Perplexity (LCP) restraint

Our third sequence complexity restraint also used local-window entropies, but applied a quadratic penalty on corresponding perplexities when the entropy fell below a predefined threshold

$$C_3 = \frac{L}{L-w+1} \sum_{i=1}^{L-w+1} \left(e^{\hat{S}} - e^{S_i}\right)^2 \Delta\left(S_i < \hat{S}\right). \quad (2)$$

where $\hat{S}$ is the threshold entropy value and $\Delta\left(S_i < \hat{S}\right)$ is an indicator variable of whether S_i falls below $\hat{S}$. Here, we used $w = 30$ and as $\hat{S}$ we chose the 5th percentile of 30-residue local window entropies in PDB sequences (~ 2.32 nats). All three restraints effectively restricted the sampling of sequences from Potts models to regions of expected sequence complexity for native-like sequences, with the last two having the advantage of not introducing potentially undesired global inter-residue correlations.

J Evaluation: Unconditional Samples

J.1 Sample generation

We generated three sets of unconditional protein samples using Chroma. All sets used the same parameters: 500 steps, $\lambda_0 = 10$, and $\psi = 2$. Of these three sets, we used `ChromaBackbone v0` and `v1` to generate two sets of single-chain proteins and `ChromaBackbone v1` to generate one set of multi-chain proteins. The single-chain sets each contained 50,000 samples and the lengths were drawn from a "1/length" distribution, where the probability of a protein chain's length was inversely proportional to its length constrained to a minimal length of 50 and a maximal length of 1,000 residues. This length distribution induces the property that any random residue in the generated data will be equally likely to belong to any particular length protein.

The multi-chain set contained 10,000 samples with the length distribution taken from the empirical statistics of chain lengths in PDB complexes. Specifically, for each Chroma sample, we drew a random protein complex from the PDB and took the number of chains and their lengths from that complex. Supplementary Figures 9 and 10 show randomly-chosen (i.e., non-cherry picked) samples from the resulting sets for single-chain and multi-chain examples, respectively.

J.2 Backbone geometry statistics

We evaluated the structural validity of Chroma-generated single-chain structures by characterizing their secondary structure and residue interactions alongside a non-redundant subset of structures from the PDB (Supplementary Table 5). Secondary structure classification (α-helix, β-strands, and coil) was performed using Stride [140]. We defined two residues as interacting if their C_α atoms were within 8 $\AA$ of each other, and computed the mean number of long-range residue contacts. We also computed contact order [141] and radius of gyration [89], length normalizing them according to their corresponding empirical power laws (see Supplementary Table 5). Supplementary Figure 11 panels a-d show the distributions of these metrics.

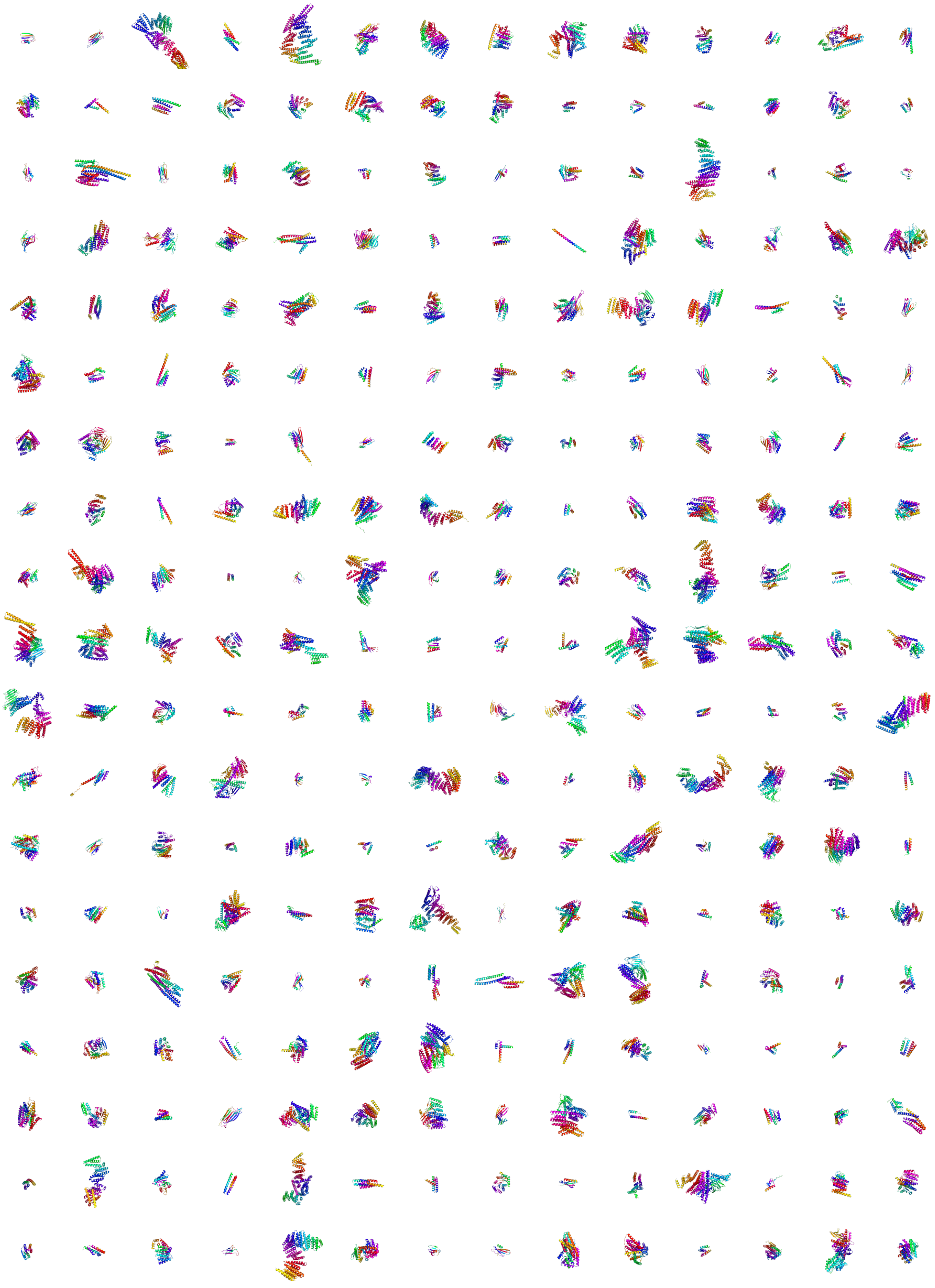

Supplementary Figure 9: **Random single-chain samples from `ChromaBackbone-v1`.**

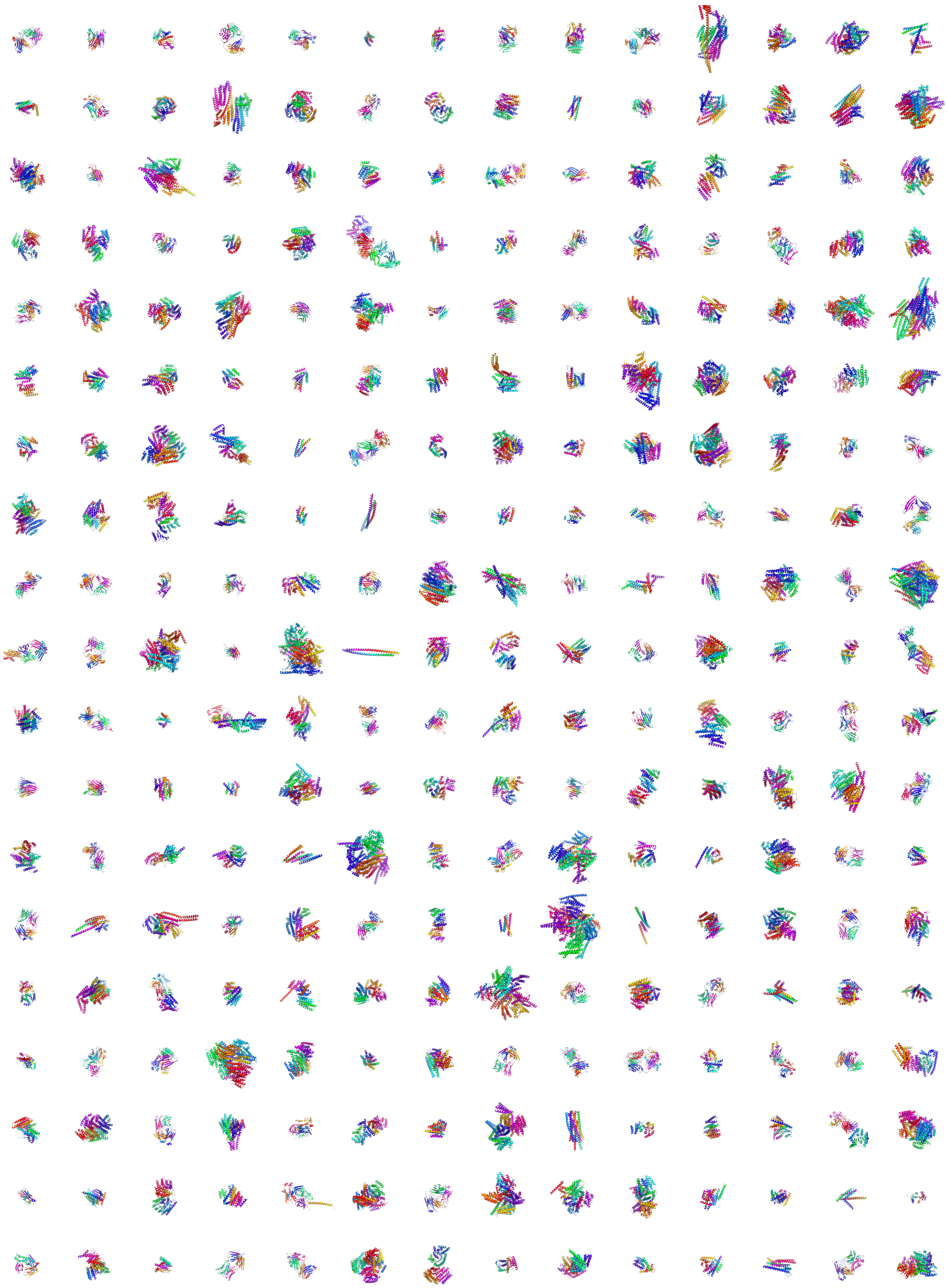

Supplementary Figure 10: **Random complex samples from `ChromaBackbone-v1`.**

J.3 Tertiary motif analysis

Natural protein structures exhibit considerable degeneracy in their use of local tertiary backbone geometries, such that relatively few local tertiary motifs account for the majority of the observed

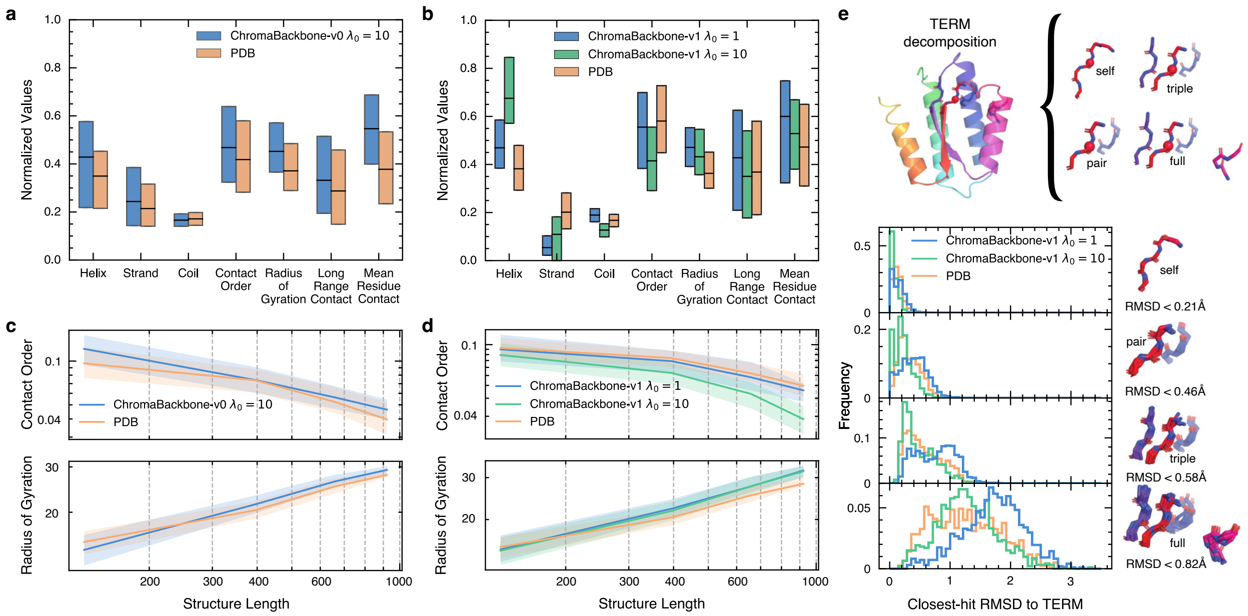

Supplementary Figure 11: **Unconditional backbone samples reproduce both low and high order structural statistics of natural proteins. a** and **c**, Distributions of structural properties (in **a**) and length-dependent scaling of contact order [136] and radius of gyration (in **b**) computed on a set of 50,000 single-chain samples from the unconditional `ChromaBackbone v0` at inverse temperature $\lambda_0 = 10$, compared to the corresponding metrics for PDB structures. **b** and **d**, Distributions of structural properties (in **b**) and length-dependent scaling of contact order and radius of gyration (in **d**) computed on a set of 50,000 single-chain samples from the unconditional `ChromaBackbone v1` at inverse temperature $\lambda_0 = 10$ and a set of 500 single-chain samples from the unconditional `ChromaBackbone v1` at inverse temperature $\lambda_0 = 1$, compared to the corresponding metrics for PDB structures. Box plots in **a** and **b** show medians and inter-quartile ranges. **e,** The distribution of closest-match RMSD for TERMs of increasing order originating from native or Chroma-generated backbones (at inverse temperature λ_0 of 1 or 10, as indicated).

Metric	Description	Normalization
Secondary structure content (SS_i)	Fraction of residues mapping into Helix, Strand, or Coil secondary-structure classes	none
Mean Residue Contact (C_{mean})	Average number of contacts per residue	none
Long-range Residue Contact (C_{long})	Average number of long-range contacts per residue. Contacts are long-range if they involve residues separated in sequence by 24 or more positions	none
Contact Order (CO)	Average sequence distance between contacting residues normalized by the total length of the protein; higher contact orders generally indicate longer folding times	$CO/N^{-0.3}$ [141]
Radius of Gyration (R_g)	Root mean square distance of structure's atomic coordinates from its center of mass	$R_g/N^{0.4}$ [89]

Supplementary Table 5: **Structural metrics used for characterizing backbone geometries**

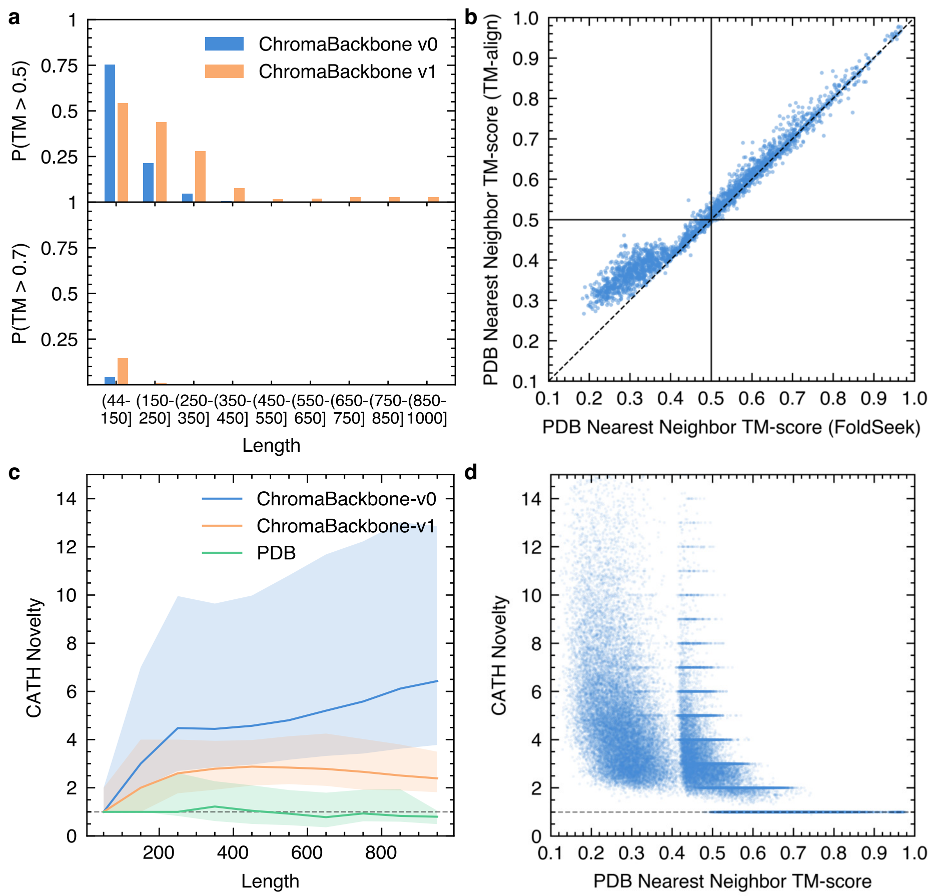

Supplementary Figure 12: **Unconditional backbone samples demonstrate structural novelty across different metrics and protein sizes.** `ChromaBackbone v0` and `v1` datasets used for this evaluation are defined in Appendix J.1. **a,** Fraction of backbones with PDB nearest neighbor TM-scores above 0.5 (top) or 0.7 (bottom) by length, for `ChromaBackbone v0` and `v1`. **b,** CATHdb nearest neighbor TM-scores by TM-align and FoldSeek agree closely. **c,** Length-normalized number of CATH domains required for 80% coverage versus structure length for `ChromaBackbone v0`, `v1`, and PDB. **d,** Length-normalized number of CATH domains required for 80% coverage versus PDB nearest neighbor TM-score (by FoldSeek) for both `ChromaBackbone v0` and `v1`.

structure space [142]. These tertiary motifs, or TERMs, consist of a central residue, its backbone-contiguous neighbors, neighboring residues capable of contacting the central residue, and their backbone-contiguous neighbors [142, 143]. Depending on how many contacting residues are combined into the motif, TERMs can be distinguished as self, pair, triple, or higher-order, corresponding to having zero, one, two, or more contacting neighbors (Supplementary Figure 11e, top), respectively. To compare the local geometry of Chroma-generated backbones with that of native structures, we randomly sub-sampled self, pair, triple, and full TERMs (i.e., TERMs containing all contacting residues for a given central residue) within Chroma backbones and identified the closest neighbor (by backbone RMSD) to each within the "search database"—i.e., the training set used for Chroma. We performed a similar analysis on a set of native proteins not contained within the search database–i.e., the test set used for Chroma. Although the test and training sets had been

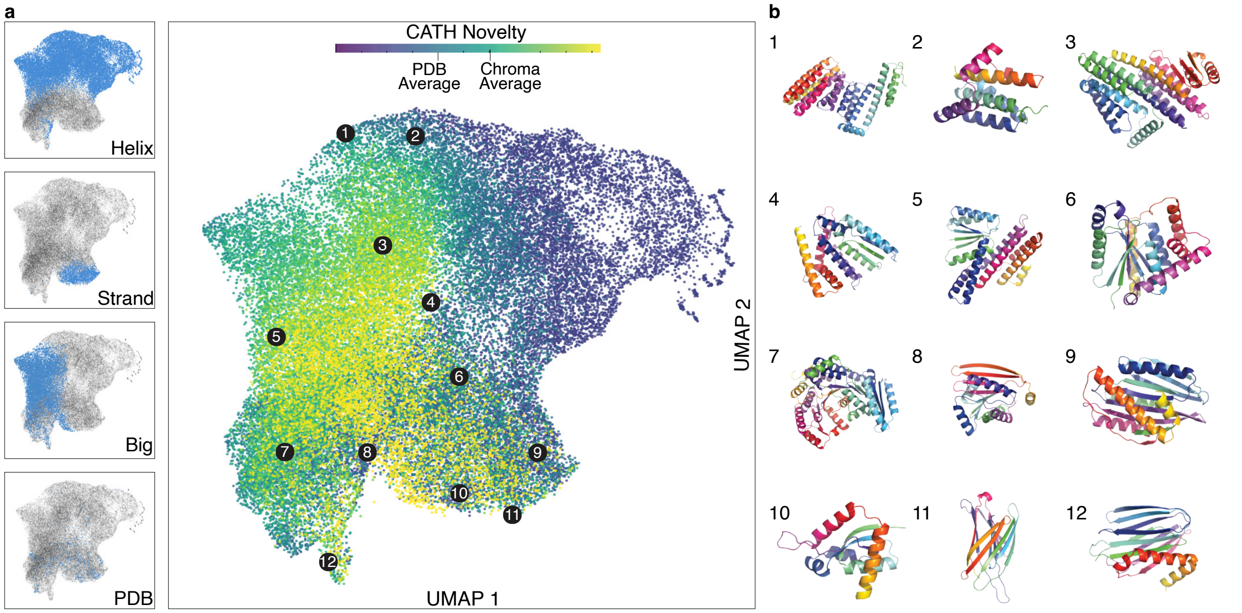

Supplementary Figure 13: **Unconditional backbone samples span natural protein space while also frequently demonstrating high novelty.** **a**. We co-embedded $\approx 50,000$ samples from `ChromaBackbone v1` along with a small set of about ≈ 500 samples from our PDB test set using UMAP [137] on 31 global fold descriptors derived from knot theory [138, 139]. We visualize in the largest embedding plot all of these points colored by our length-adjusted CATH novelty metric, which estimates the normalized number of CATH domains needed to achieve a greedy cover at least 80% of residues at TM > 0.5. We use this score because it continues to grade the novelty of longer proteins which almost all have a PDB nearest-neighbor TM < 0.5. On average Chroma has a CATH novelty score of 2.7 and PDB has a CATH novelty score of 1.9. The four embedding insets (left) demonstrate the specific distributions of properties of interest by highlighting populations of structures that are mainly helices, strands, large (> 500 residues), or from the PDB test set. **b**, We highlight twelve proteins from across the embedding space with a high novelty score (with embedding locations numbered)

split by chain-level sequence homology, we took further care to exclude any apparent homologs of native TERMs from consideration as matches. To this end, we compared the local 31-amino acid sequence windows around each TERM segment and its corresponding match, with any pairings reaching 60% or more sequence identity not being allowed to participate in a match.

Supplementary Figure 11e shows the distribution of closest-neighbor RMSDs for TERMs derived from both native and Chroma-sampled backbones that were generated at inverse temperatures $\lambda_0 = 10$ and $\lambda_0 = 1$. The distributions of nearest-neighbor RMSD were very close for low-temperature samples from Chroma and native proteins, indicating that Chroma geometries are valid and likely to be as designable as native proteins, including complex motifs with four or five disjoint fragments (see Supplementary Figure 11e, bottom panel). Because native amino-acid choices are driven by these local geometries [144], and adherence to TERM statistics has been previously shown to correlate with structural model accuracy and success in *de-novo* design [143, 144], this argues for the general designability of Chroma-generated backbones in a model-

independent manner. Notably, the samples from Chroma at its natural temperature (i.e., $\lambda_0 = 1$) still utilize precedented low-order TERMs, while their geometries do begin to depart from native for higher-order motifs.

J.4 Novelty analysis

We assessed the novelty of Chroma-generated samples by comparing them to natural protein folds from CATHdb S40 [145] and PDB100 with FoldSeek (5-53465f0) [146]. For each sample, we identified the closest hit in the PDB (with the highest TM-score) by using FoldSeek to search against the highest resolution experimental structure within each cluster of PDB100. We estimated novelty by computing fractions of entries with TM-scores above 0.5, 0.7 or 0.9; see Supplementary Figure 12a for results using `ChromaBackbone v0` and `v1`.

Additionally, we aligned all Chroma-generated samples against the full CATHdb dataset (all-to-all) using FoldSeek. We greedily determined the number of domains needed to cover at least 80% of the query by identifying the hits with the highest number of residues within 5 $\AA$ of the query that were not already covered. The number of domains required increases with query size given that CATH domains typically have a length ranging between 50 and 200 amino acids. We defined a length-normalized CATH novelty metric as the number of domains required to cover 80% of the query divided by $\frac{\max(L,300)}{300}$, where L is protein length. As a baseline, we analyzed our PDB test set using the same approach (see Supplementary Figure 12c,d).

Finally, we embedded single-chain structures from Chroma and the test set in 31 Gauss Integral dimensions using the `pdb2git` program from the Phaistos suite [139, 147]. Discarding the structures that failed to embed, the remaining 47,786 Chroma samples and 561 natural folds were projected onto a two-dimensions space using UMAP [137] with default parameters of 25 neighbors and a minimal distance of 0.5 (see Supplementary Figure 13).

J.4.1 TM-align versus FoldSeek

While the TM-scores produced by the program TM-align [148] are a well-established standard for comparing structures, we used FoldSeek for computational efficiency (allowing all-to-all comparisons) and tuned it to closely reproduce TM-align results. Specifically, by comparing a subset of 3,000 unconditional structures to the $\sim$ 32k structurally conserved domains from CATHdb S40 set with TM-align and FoldSeek, we found that using FoldSeek with parameters:

```
--alignment-type 1 --min-seq-id 0 -s 20 -e inf --max-seqs 17000 -k 5
--num-iterations 2
```

provided the best trade-off between compute time and retrieval. There is an overall good agreement between the two programs when the highest TM-score is above 0.45, with the median difference of -0.003, 95 %CI [-0.013,-0.0003]. FoldSeek tends to overestimate novelty below this cutoff by a median difference of -0.057, 95 %CI [-0.08,-0.024]. Comparison between FoldSeek and TM-align is summarized in Supplementary Figure 12b.

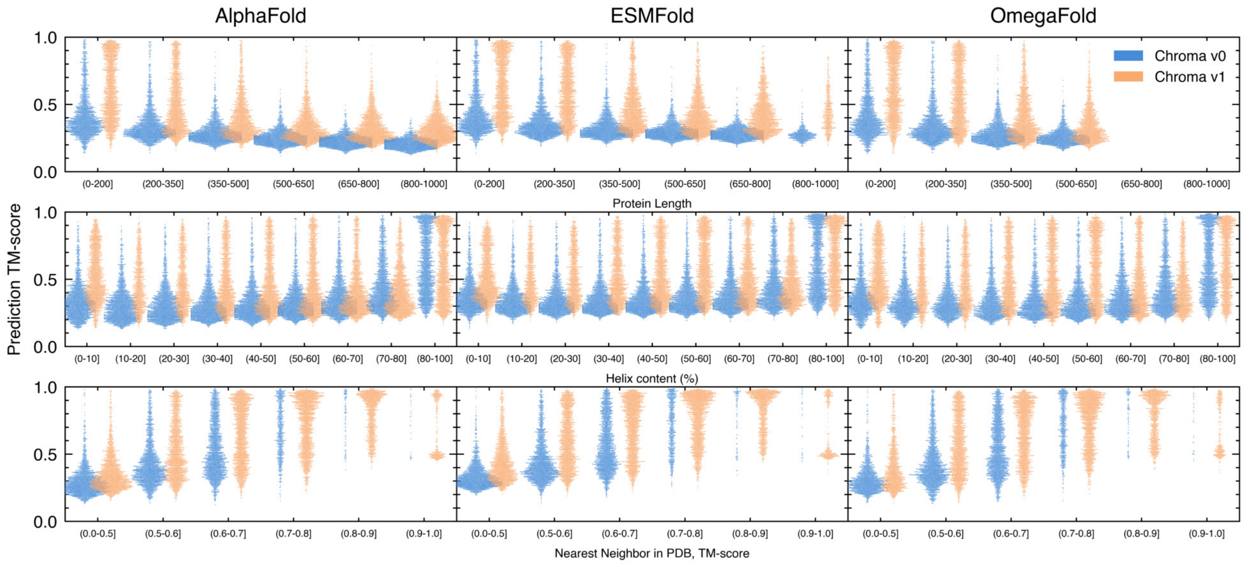

Supplementary Figure 14: `ChromaBackbone v0` **and** `v1` **refolding TM-scores across length, secondary structure, and novelty.** TM-scores between Chroma-generated structures and structures predicted for the corresponding designed sequence using AlphaFold, ESMfold, and OmegaFold, across length, helical content, and novelty. A maximum of 2000 points per model and bin is shown. Due to hardware limitations, ESMFold predictions were restricted to proteins shorter than 848 amino acids and OmegaFold predictions were restricted to proteins shorter than 618 amino acids.

J.5 Refolding analysis

We generated one sequence for each of the sampled single-chain unconditional structures (see section J.1) for both `ChromaBackbone v0` and `v1`. We used the Potts decoder of `ChromaDesign Multi` as described in section I.2 (conditioned on $t = 0$.) in conjunction with the Local Composition Perplexity restraint described in equation 2. We then used AlphaFold [66, 149], ESMFold [150], and OmegaFold [111] to predict structures of each the designed sequences. A summary of the results is presented in Supplementary Figure 14. While shorter sequences refold successfully more frequently, there is a non-trivial fraction of even very long designs (e.g., 800-1000 residues) that do refold quite accurately (Supplementary Figure 14, top row). Interestingly, helix content does not appear to be a strong predictor of refolding (Supplementary Figure 14, middle row), but the distance to the nearest neighbor in the PDB does (Supplementary Figure 14, bottom row). Validation through refolding is most challenging for novel structures, as both the generation and prediction tasks are most challenging in this limit and require strong generalization of the underlying methodology.

J.6 Sequence design analysis

We used `ChromaDesign Potts` and `ChromaDesign Multi` to generate protein sequences on the test set using different complexity penalty methods, reflecting the experimental validation approach. We assessed sequence recovery for all residues, as well as over exposed, core, and interface

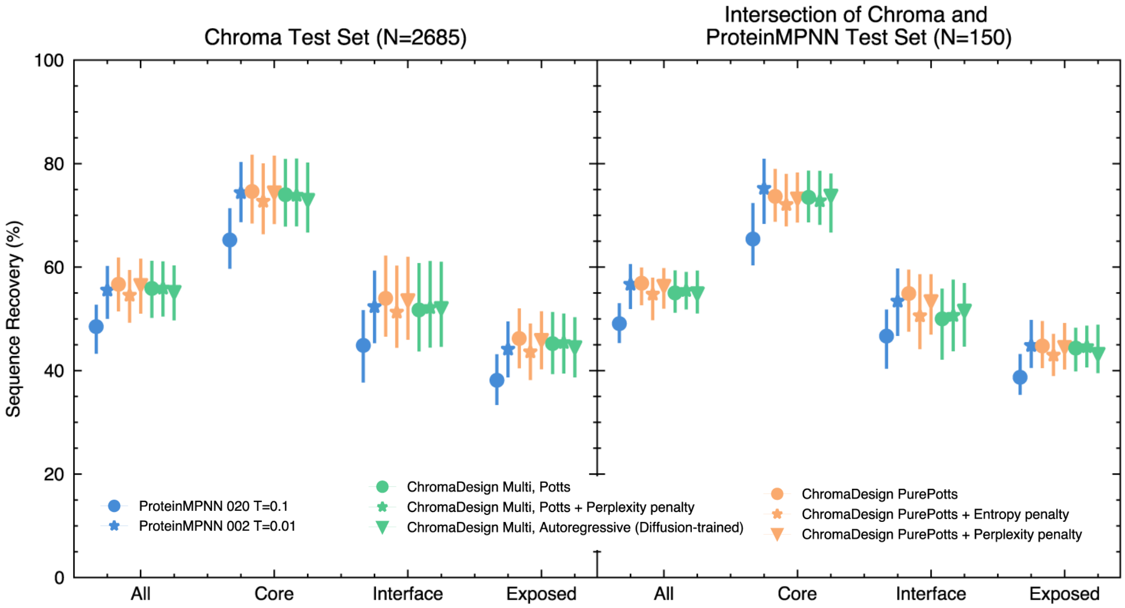

Supplementary Figure 15: **ChromaDesign and ProteinMPNN have comparable sequence recovery on natural proteins.** We plot the median and interquartile ranges of per-protein sequence recoveries when evaluated on the Chroma test set (left) and an intersection of the Chroma and ProteinMPNN test sets (right).

regions. We compared performance to ProteinMPNN [116] using the 002 checkpoint at a temperature of 0.01, as well as the 020 checkpoint at a temperature of 0.1. Considering that a substantial portion of Chroma's test set was incorporated into ProteinMPNN's training set, performance was assessed on the overlapping entries of both test sets. A summary of the performances is shown in Supplementary Figure 15. Chroma designs and ProteinMPNN 002 exhibited comparable performance across all regions and subsets, while ProteinMPNN 020 tended to have lower sequence recovery. Neither of the complexity penalty methods appeared to have a meaningful impact on the performance of ChromaDesign.

K Evaluation: Conditional Samples

In this section, we demonstrate the effectiveness of our integrated approach of programmable generation and design in creating protein structures capable of refolding *in silico*. We focus on evaluating our methods against state-of-the-art protein structure models such as AlphaFold [66, 149], ESMFold [150], and OmegaFold [111]. Our expectation is that the proteins generated by our design exhibit novel structures and sequences. Therefore, we do not anticipate multiple sequence alignment (MSA) hits, prompting us to deploy AlphaFold with a high number of cycles. To compare refolded structures with generated ones, we compute the Template Modeling (TM) score using the TM-align software [148]. For each generated backbone, we design one sequence following the methodology described in section U.1 and report the TM score between the original and refolded backbones.

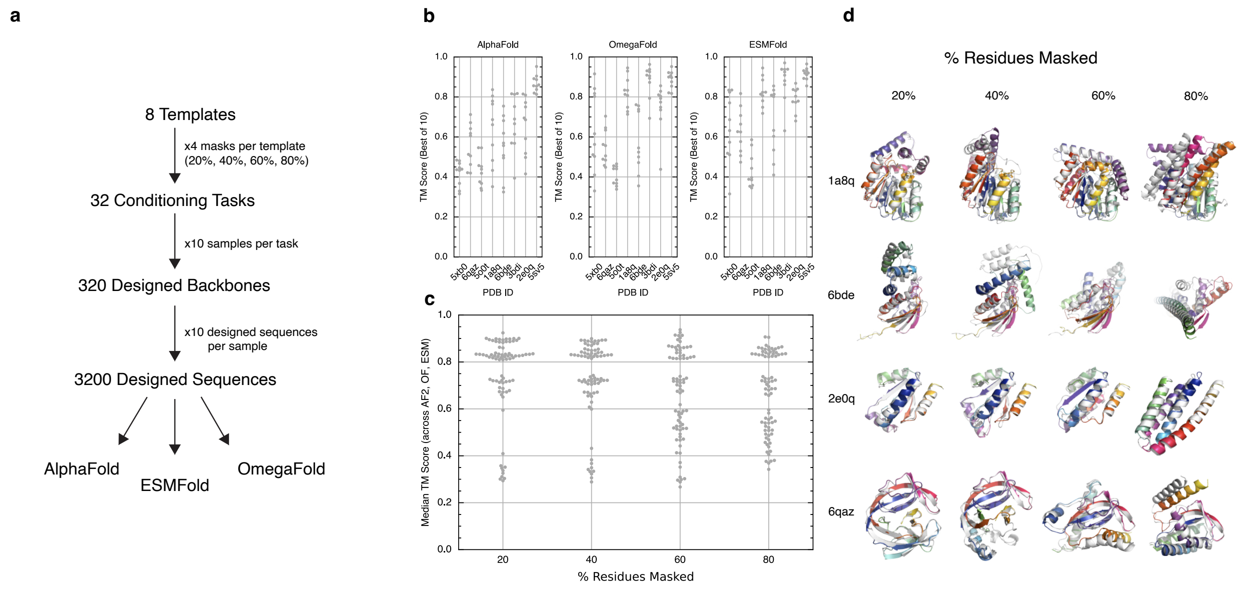

Supplementary Figure 16: **Substructure-conditioned samples can refold *in silico*.** **a**, Schematic outlining the refolding pipeline generating these data **b**, Best-of-ten TM scores for each sampled backbone for each PDB, aggregated across task for each structure prediction method (AlphaFold2, OmegaFold, and ESMFold). Substructure-conditioned samples are able to achieve best-of-ten TM scores higher than 0.5 for every PDB considered with each structure prediction method. **c**, Median TM score (across structure predictors) per task. Distribution of median TM score shifts down as more of the protein backbone is occluded. **d**, Example samples along with predicted structures (drawn in white) for three PDBs across each task.

K.1 Refolding substructure-conditioned samples

Eight PDBs were selected for this evaluation by sampling from the test set restricted to monomers with lengths between 60 and 500 amino acids and no missing structural data. For each template, we explore refolding rate on four conditional generation tasks, each of which consists of masking out a fraction (20%, 40%, 60%, and 80%) of the residues and conditioning on the atomic coordinates of the unmasked residues. Masks are obtained by shifting a plane normal to the first principal component of the atomic coordinates until the desired percentage of residues are masked. For a formal description of the conditioning task as well as the method under evaluation, see section N. For each of the 32 conditioning tasks, ten backbones were sampled subject to a filtering which excluded samples containing discontinuities, clashes, or stereochemical violations, resulting in a total of 320 backbones. For each backbone, ten sequences were designed using the method described in I, and the resulting 3200 sequences are refolded with AlphaFold2 [66], OmegaFold [111], and ESMFold [110].

TM scores between predicted and designed models are evaluated, the results are summarized in Supplementary Figure 16. Calling a backbone a hit at a best-of-ten TM score cutoff of 0.5, we see non-zero hit rate across all PDBs considered for all three structure prediction methods, with 100% hit-rate achieved on several PDB-task-structure-predictor combinations. At a TM score cutoff of

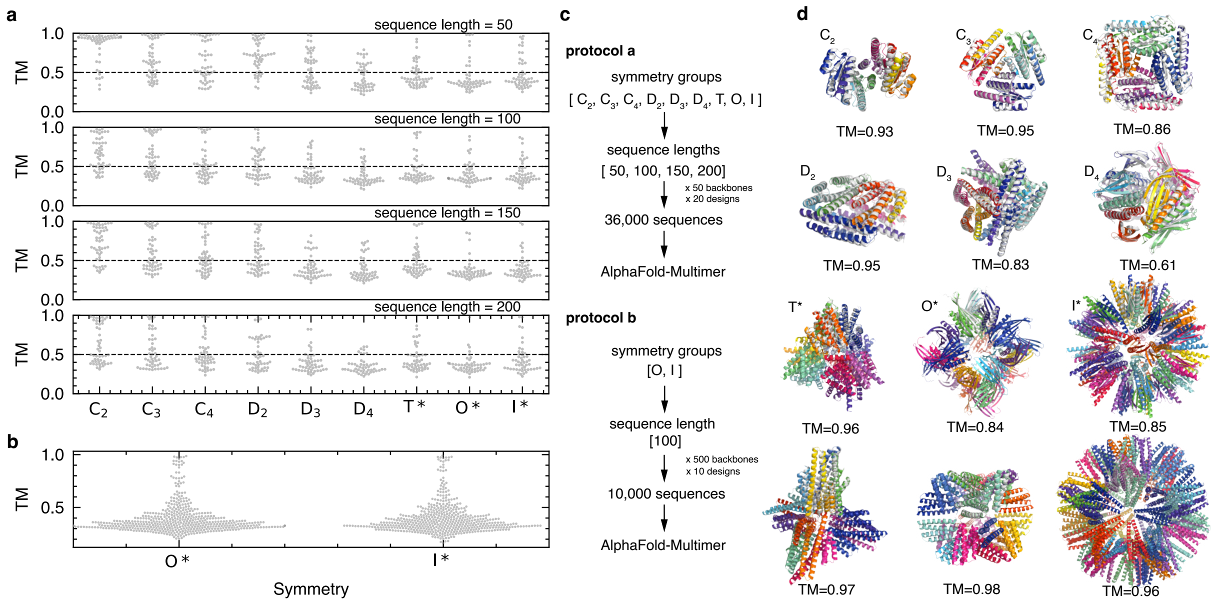

Supplementary Figure 17: **Symmetry-conditioned samples can refold *in silico*.** **a**, **b**, Refolding statistics for all the generated backbones (no filtering). **c**, Computational protocol for refolding analysis. **d**, representative refolded samples for different symmetry groups.

0.8, when restricting our analysis to the task of masking out 60% of the template backbone, we see non-zero hit rate on half of the sampled PDBs for each of the three structure prediction methods. We see that refolding becomes less likely as more of the template is masked (and hence more of the monomer backbone is infilled).

K.2 Refolding symmetry-conditioned samples

We investigated the designability of symmetric assemblies generated by `ChromaBackbone v1` using AlphaFold v2 and our refolding experiments showed remarkable refolding rate, indicating that Chroma can generate highly designable assemblies. Our study involved two sets of refolding experiments across various point groups, including Cyclic (C_2, C_3, C_4), Dihedral (D_2, D_3, D_4), Tetrahedral (T), Octahedral (O), and Icosahedral (I) groups. For each symmetry group, we explored single chain lengths of 50, 100, 150, and 200 residues. For each combination of symmetry and backbone length, we generated 50 backbones **without applying any filtering**, resulting in a total of 1,800 backbones. To ensure consistency, we used the sequence design method described in appendix I, which enforced identical sequences for all chains. For each backbone, we sampled 20 sequences and used AlphaFold v2 for folding prediction, employing 10 cycles without Multiple Sequence Alignment (MSA). This process produced a total of 36,000 structure predictions.

In the case of higher-symmetry groups (T, O, and I), which consist of 12, 24, and 60 subunits, respectively, we limited our validation to symmetric trimers that respect C_3 symmetry. This choice was reasonable due to the presence of a three-fold axis in these groups. Unfortunately, reliable and rapid structure prediction models for large and high-symmetry assemblies are currently unavailable. It is important to note that while the poorer refolding results observed in the trimer-only

setting might result from excluding interface interactions from neighboring chains, this does not necessarily imply that the designed proteins will not assemble. Based on the results obtained from the aforementioned protocol, we observed a considerable number of successfully refolded designs across the selected symmetry groups and sequence lengths (see Supplementary Figure 17). The probability of success in refolding, defined as a TM-score greater than 0.5, was found to be higher for assemblies with a smaller number of subunits and shorter chain lengths. We have included selected refolded structures in Supplementary Figure 17.

Furthermore, we conducted a separate set of refolding validation experiments that focused specifically on assemblies with O and I symmetries, generating 500 backbones instead of 50. We observed a notable number of successful trimer refoldings. However, how trimer refolding correlates with assembly formation success rate requires further investigation.

K.3 Refolding shape-conditioned samples

While shape-conditioned samples may drive towards folds that are highly atypical of what is found in natural proteins, we sought to characterize to what extent they can be refolded *in silico*. For each of the 26 letters in the Latin alphabet and each of the 10 digits in the Arabic numeral system, we sampled 120 backbones representing a combination of sizes (length 500, 750, or 1000) and conditioner hyperparameters. For the conditioner hyperparameters, we considered two configurations: (i) one with fixed point cloud scaling, $\psi = 2$, and the hybrid SDE and (ii) the other with autoscaling, $\psi = 3$ and purely annealed Langevin dynamics. Ultimately we found both methods gave a large number of refolding hits so these may be primarily regarded as a mechanism for diversity. For each of the backbones sampled in this workflow we sampled 5 sequences and refolded with all three structure prediction methods. We outline the overall workflow and results in Supplementary Figure 18.

Remarkably, we observe refolding with high TM-scores across all 36 shape classes and all 3 structure prediction methods (Supplementary Figure 18), even though samples were at minimum 500 residues long which is often a difficult regime for *in silico* refolding. Every shape shown in Figure 3c scored at least an ESMfold TM-score of 0.65 (many higher than this), and when we visualize the ESMfold models with the highest TM-score correspondence to our Chroma designs in Supplementary Figure 18d, we see that many of them successfully refold into the intended 3D shapes. We emphasize that the only information being passed to ESMfold is the amino acid sequence, and we in no way use ESMfold during the sampling process itself other than for the final selection of models to examine based on agreement. Thus, it would appear that both structure predictors and Chroma are capturing sufficiently similar sequence-structure relationships to agree on how they might be leveraged to propose folds.

K.4 Refolding class-conditioned samples

Refolding on CATH class (fold) conditioned samples works *in silico*. To illustrate the performance of the ProClass conditioner and provide *in silico* evidence that the designs can be made in the lab, a computational sampling protocol was run as illustrated in Supplementary Figure 19 (left). Three canonical folds were selected: beta barrel, Rossmann fold, and IG fold. For each fold, 2000 conditional backbones were sampled. For each fold, the top 100 samples (evaluated by p(fold)

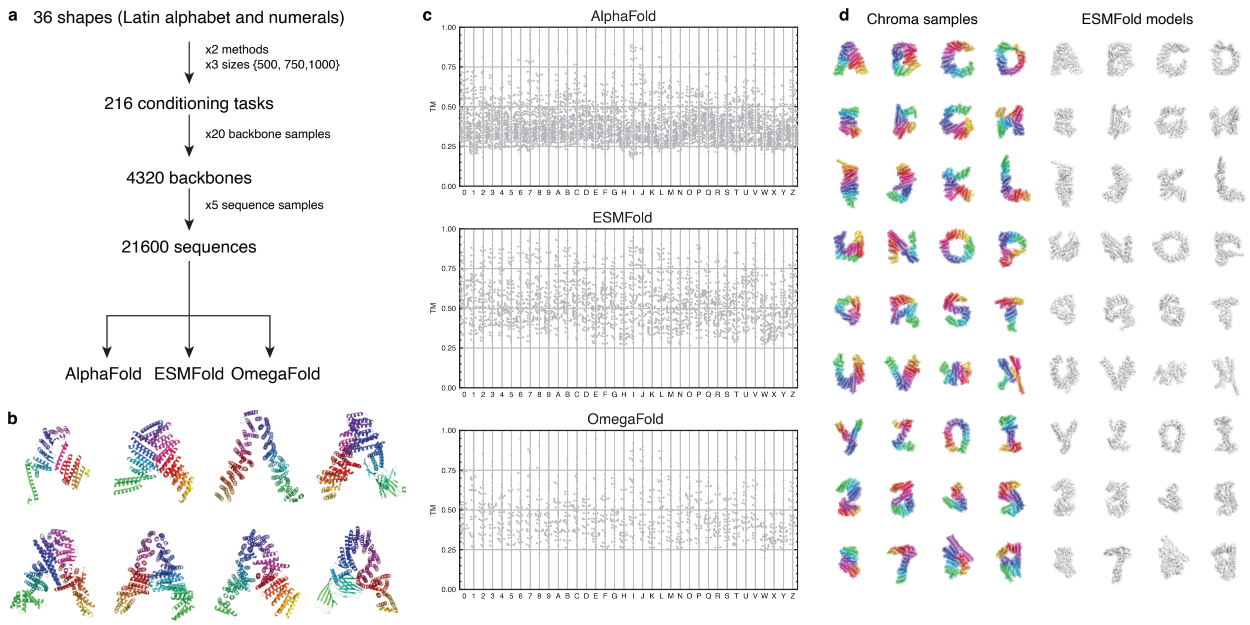

Supplementary Figure 18: **Shape-conditioned samples can refold *in silico*.** **a**, Experimental protocol for refolding analysis of shape-conditioned samples. **b**, Samples for even the same shape cue, such as the letter *A*, can exhibit large topological variations. **c**, Top TM-scores per backbone out of 5 designed sequences across three different folding methods. **d**, The ESMfold models (white) with the highest level of TM-score agreement with the Chroma model (rainbow).

under ProClass) were selected for design and refolding. Sequence design was performed 100 times for each backbone, then each of the resulting 30,000 resulting sequences were folded by three folding models: AlphaFold, OmegaFold, and ESMFold. To evaluate if the refolding was successful for each model a TM score was calculated against the generated backbone. If that TM score was greater than 0.5 it was considered a successful refolding event. Overall success of a backbone was evaluated by choosing the best TM score out of 100 designs.

Choosing hyperparamers that allow for successful optimization of the backbones requires tuning. Two key hyperparameters are guidance scale, and `max_norm`. Both need to be tuned to achieve high-quality samples. The guidance scale rescales the gradient of the conditioner for sampling, while `max_norm` provides a maximum gradient norm above which the gradient is clipped. If the guidance scale is too low the sample looks like an unconditioned sample. If the guidance scale is too high, it breaks local backbone bond length constraints and the sampled protein might explode. `max_norm` choices that are too low result in gradient clipping in a way that prevents optimization. If `max_norm` is chosen to be too high, random outlier gradients can cause the sampling trajectory to fail, as occasionally the gradients explode and destroy the sample. This random gradient explosion does not occur for all conditional sampling problems, and so is evaluated on a case-by-case basis.

The conditional parameters depend on various other sampling hyperparameters, so must be determined for each sampling problem separately. The best choice for guidance scale tends to vary based on inverse temperature, Langevin factor, and the number of steps. Practically, the guidance

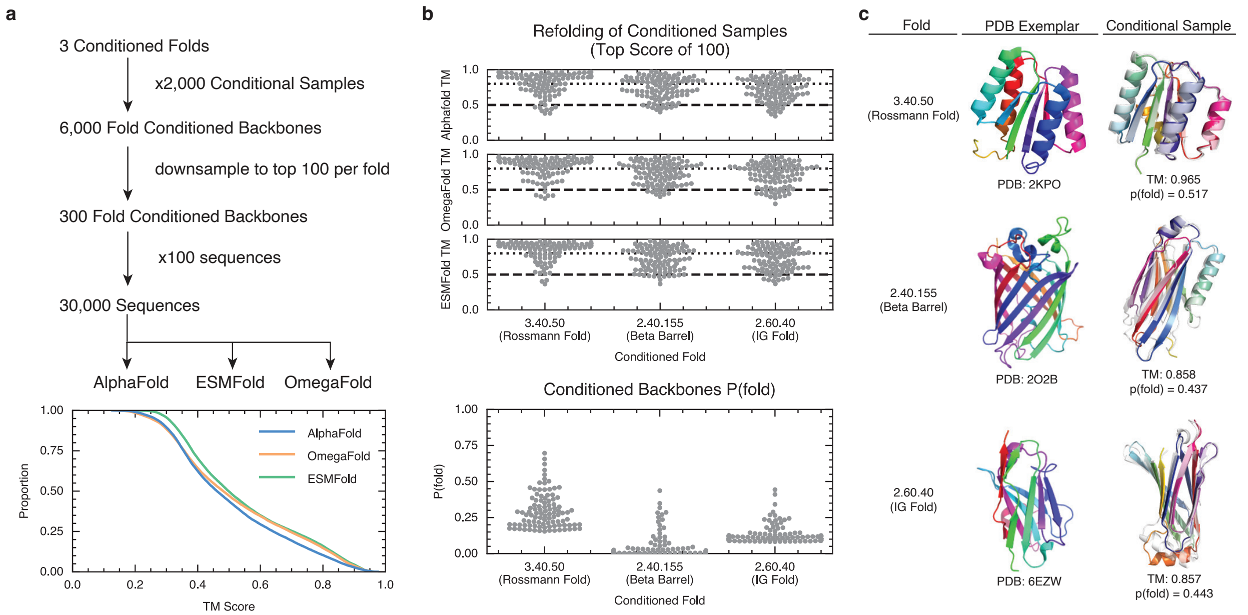

Supplementary Figure 19: **Class-conditioned samples can refold *in silico*.** **a**, Conditional generation protocol diagram. Three canonical folds were chosen to conditionally design the beta barrel, Rossmann fold, and IG fold. 2000 conditional samples were generated for each fold. The best 100 of each fold were selected for downstream refolding analysis. There is close agreement in TM score for all folding algorithms for these samples. **b**, Each backbone was designed 100 times and refolded under each folding model. Almost all of the structures refold with a TM score greater than 0.5 in best of 100 sequence designs. In the bottom plot, Conditioned backbones have a range of probabilities of being the correct fold. In general conditioning on CAT class requires many samples before high quality examples are generated. Some are easier to optimize than others. **c**, A selection of the best examples for each fold in conditional design. The middle column illustrates an example of the same class from the PDB for reference. The right column is an exemplar protein generated from Chroma. In white is the refolded structure, in rainbow is the sampled backbone.

scale and `max_norm` are found by a small sampling hyperparameter search. A small number of seed-controlled samples are run at different choices of `guidance scale` and `max_norm` (e.g. 0.1, 1, 10, 100). Then the best performing values are chosen for a production run. We chose `max_norm` to be 10.0, and use a guidance scale of 0.1 for the refolding experiments described in Supplementary Figure 19(a).

Refolding successes were observed across all three conditioned folds. Refolding had high agreement across models, as seen in Supplementary Figure 19(left bottom). Further in Supplementary Figure 19(middle), about 40% of the designs meet the threshold for refolding success. For a design to be considered successful, it also has to have a high p(fold). Qualitatively this cutoff can vary on what is acceptable, however, the best samples tend to be close to 1. In Supplementary Figure 19(b bottom), the top 100 backbones are seen to vary substantially in the best optimization performance achieved. Some CAT annotations are very difficult to optimize, whereas others are relatively easy and good samples can be found quickly. In all three cases, structures that refolded and match the desired fold were found. These examples can be seen in Supplementary Figure 19(c).

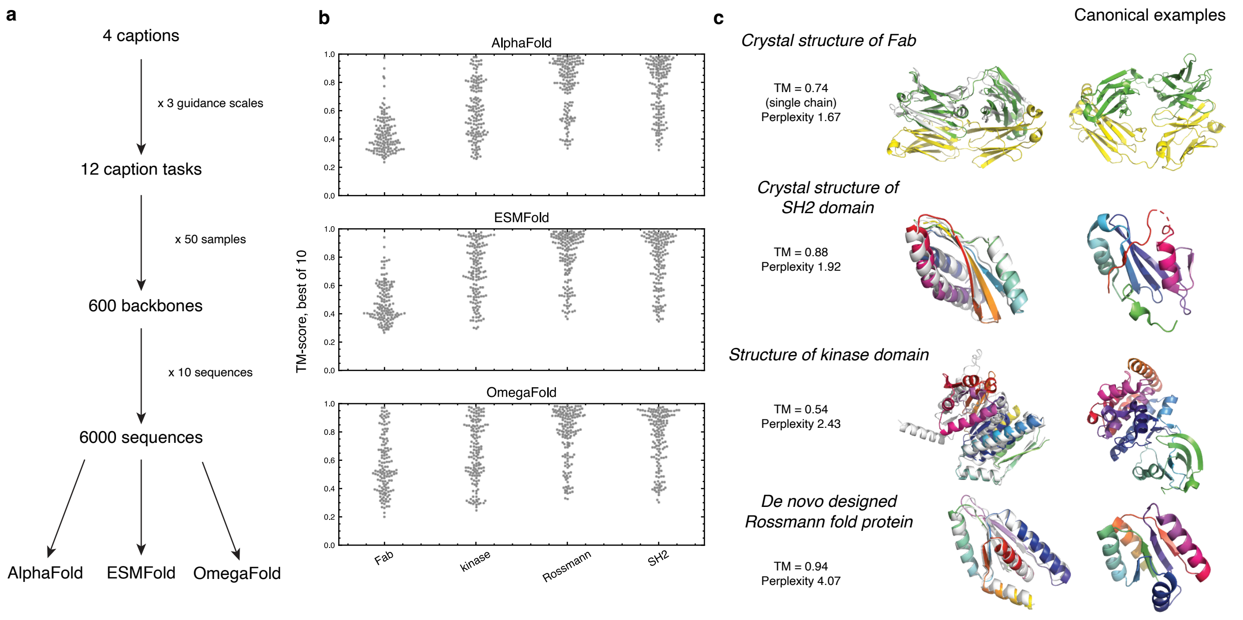

Supplementary Figure 20: **Natural language-conditioned samples can refold *in silico*.** **a**, Schematic that outlines the refolding pipeline generating these data. Structures conditioned on the caption "Crystal structure of Fab" have two chains of 200 residues in length. Structures conditioned on SH2 domain, kinase domain and Rossmann fold captions have single chains of length 110, 300 and 125 residues, respectively. In all cases, a task token is passed to the caption model specifying that the caption represents the entire structure; see Appendix T for further details. Other sampling parameters are listed in Supplementary Table 4. **b**, Best-of-ten TM scores for structures sampled with guidance from each caption and refolded using different structure prediction methods (AlphaFold2, ESMFold, and OmegaFold). OmegaFold is run on only the first chain for complexes. **c**, Chroma backbones (rainbow) superimposed on OmegaFold predicted structures (white), alongside examples from the PDB for each caption for comparison.

K.5 Refolding language-conditioned samples

To demonstrate the designability of samples conditioned on natural language, we draw backbones with reverse diffusion guided by the gradients of a model that predicts $p(y|\mathbf{x}_t)$, where y is a particular caption. Details about the underlying model are given in Appendix T. For each of the four captions, we sample 50 backbones using three different guidance scales (1, 10, and 100) to combine the conditioning gradient with the gradient from the diffusion model. The backbone length and number of chains are chosen separately per caption to be similar to representative examples in the PDB. Subsequently, we design ten sequences for each backbone and refold as in appendix K.1. For backbones with more than one chain, when using OmegaFold we only fold the first chain, rather than the entire complex. We find that larger guidance scales can result in incoherent backbones with particularly low likelihood, and reject those structures derived from backbones with ELBO below 0. For the remaining backbones, refolding performance is approximately the same regardless of guidance scale, and a scale of 10 tends to provide an acceptable balance between effective conditioning and ELBO. The TM scores between each surviving designed backbone and its best refolded structure are shown in Supplementary Figure 20.

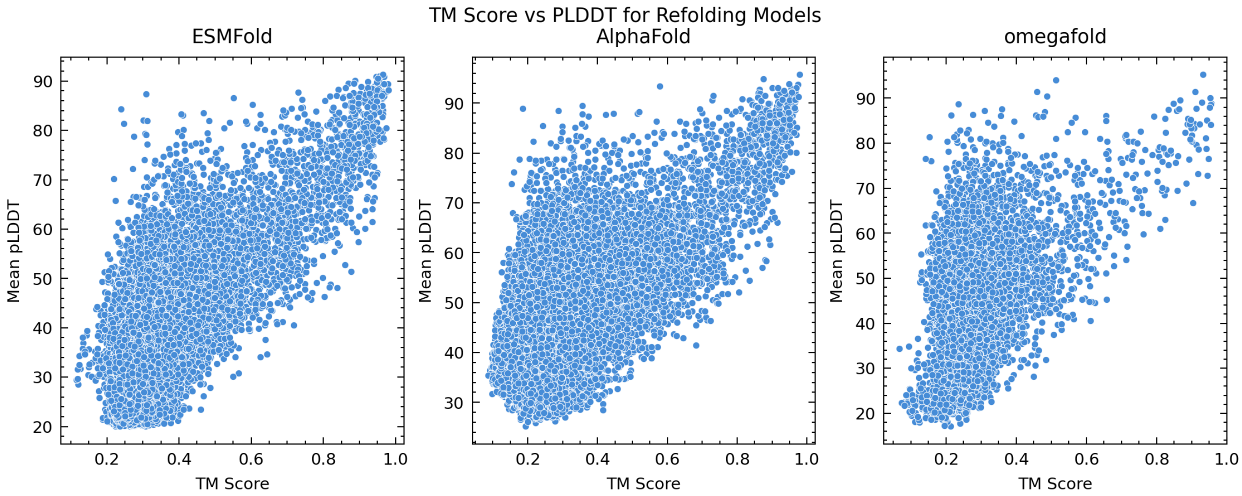

Supplementary Figure 21: **The agreement of predicted structures with designs (TM-score) is correlated to model confidence (pLDDT).** We evaluated ESMFold, AlphaFold, and OmegaFold models on the 35,000 unconditional samples generated in the ablation study, which represent model behaviors and biases across several different configurations. We see across these data that structure predictions with high correspondence between Chroma models and refolded predictions are also generally higher confidence predictions, suggesting a general self-consistency between the sequence structure relationships being modeled across these different systems.

We find examples of designability for structures conditioned on each caption, although the success rate varies considerably. The single chain structures refold with larger TM scores to their Chroma predictions than complexes (antibody example). Nevertheless, for all captions we observe instances where our design protocol is successful, as measured by refolding with a TM score above 0.5. We also show some comparisons of Chroma and successfully refolded structures in the right panel of Supplementary Figure 20, alongside canonical examples of each caption from the PDB.

K.6 Analysis of structure prediction confidence versus refolding TM

We observe a correlation between TM-Score and pLDDT for ESMFold, AlphaFold, and OmegaFold across 35,000 unconditional samples generated in the ablation study. For all three predictors, we see a correlation between predictor *consistency*, i.e. the TM-score between the generated protein and the refolded protein, and predictor *confidence*, i.e. the pLDDT of the predicted structural model model. We visualize this correspondence in Supplementary Figure 21.

L Evaluation: Ablation Study

To better understand the influence of our proposed covariance model (Supplementary Appendix B), graph neural network topology (Supplementary Appendix E), atomic output layer parameterization (Supplementary Appendix F), and losses (Supplementary Appendix B), we trained multiple variants of the model that ablate and modify different components as detailed in Supplementary

Figure 22. These ablations were evaluated through the lenses of likelihood and sample quality to holistically evaluate their effects on model performance.

L.1 Alternate model configurations and training

In this section, we briefly review the components of the model that we modified as well as their respective variations.

Model component: Covariance. We consider two covariance models for defining the diffusion process, which are are visualized in Supplementary Figure 3 and described in Appendix D:

- **Covariance variant: ResidueGas.** In this model, the coordinates of each C_α are independently and identically normally distributed with standard deviation $10\AA$ (along each x, y, z dimension). The other coordinates of the N, C, and O atoms within the residue are then distributed normally around C_α with $1\AA$ conditional standard deviation. This can be considered an off-frame relaxation of frame diffusion models [67] or an all-backbone-atom extension of IID C_α diffusion models. [91].
- **Covariance variant: Globular.** This covariance model captures spatial proximity constraints in the form of correlations *within* atoms in a residue and *between* residues in a chain, while also respecting global length-dependent R_g scaling effects that arise from polymer collapse. This version includes Complex R_g scaling.

Model component: Graph We consider two kinds of graph structure, which are visualized in Supplementary Figure 4 and described in Appendix E:

- **Graph variant: *k*-NN.** This used a graph topology based on the 60 nearest neighbors in the current structure.
- **Graph variant: Random Graph.** This used a hybrid graph topology for which 20 of the edges are the nearest neighbors in the current structure and 40 of the edges are sampled according to the inverse cubic attachment model.

Model component: Output. We consider three kinds of output parameterization varying from the consensus update visualized in Supplementary Figure 5 and described in Appendix F:

- **Output variant: PairFrameA.** This uses the inter-residue geometry parameterization with three equilibration steps and one uncertainty parameter per i, j that is coupled to both translation and rotation. The predicted transforms $\mathbf{T}_{ij}$ are parameterized as linear projections from the final edge embeddings, and the coordinates are post-processed with time-dependent scaling method A.
- **Output variant: PairFrameB.** This uses the inter-residue geometry parameterization with ten equilibration steps and two uncertainty parameters per i, j, one for translation and one for rotation. The predicted transforms $\mathbf{T}_{ij}$ are parameterized as residual updates to the transforms of the current structure based on the final edge embeddings and the coordinates are post-processed with time-dependent scaling method B.

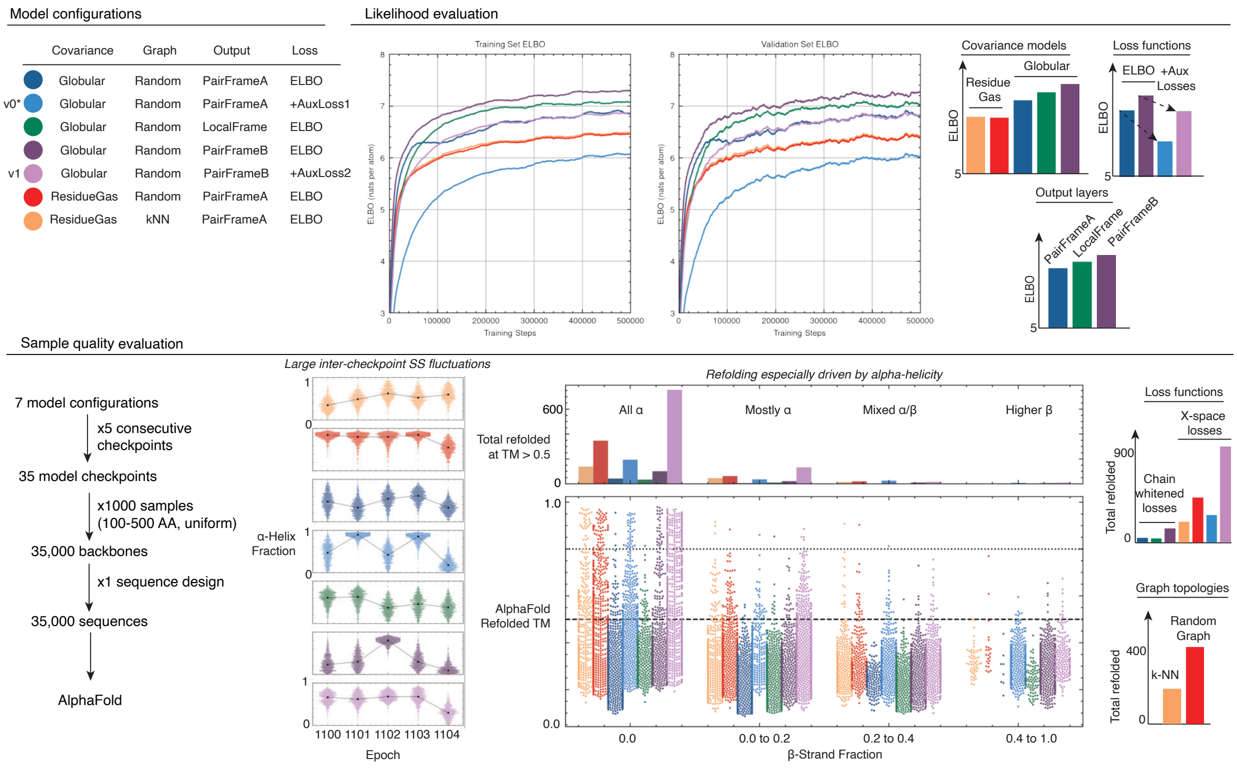

Supplementary Figure 22: **Ablation study demonstrates utility of novel model components as measured by likelihood and sample quality.** We trained seven models composing different configurations of proposed components and baselines, modifying the covariance model (Supplementary Appendix B), graph neural network topology (Supplementary Appendix E), atomic output parameterization (Supplementary Appendix F), and losses (Supplementary Appendix B) (top left). We indicate the two configurations corresponding to `ChromaBackbone v0` and `ChromaBackbone v1`, where `v0` has one additional change of using the globular monomer version of the globular covariance scaling. Training for ∼500,000 steps on 8 V100 GPUs with a batch size of ∼32,000 residues per step suggests that there is little generalization gap between the training and validation sets (top middle, windowed averaged training curves across 100 epochs). From the perspective of likelihood (top right), globular covariance is favorable to residue gas covariance (Supplementary Appendix D), inter-residue geometry prediction layer is favorable to local frame updates if tuned appropriately (Supplementary Appendix F), and auxiliary losses incur a cost to ELBO (Supplementary Appendix B). When we applied these trained models to generate unconditional samples (bottom left), we observed large fluctuations in secondary structure composition between adjacent checkpoints (bottom, middle left). When aggregating across these checkpoints, we observed that refolding by AlphaFold was highly dependent on the fraction of α-helices in the sampled structure (bottom, middle right). In spite of this, the refolding rate of samples based on a model with random graph topologies was higher than those of a model based on k-NN topologies (Supplementary Appendix E) and losses weighted in x-space induced better refolding than losses weighted only in chain-whitened space (bottom right).

- **Output variant: LocalFrame.** This uses a local frame-transform update to the coordinates that is parameterized based on the final node embeddings. The coordinates are post-

processed with time-dependent scaling method A.

We consider three kinds of losses, described in Appendix B:

- **ELBO** This is a pure likelihood loss, which is a weighted average squared error loss in whitened space together with additional additive terms to account for normalization and change of variables. It is measured in Nats per atom in Cartesian space and is comparable across different diffusion models.
- **+AuxLoss1** To the base ELBO loss, we add the ELBO-weighted unwhitened loss (Equation 1) that measures mean squared error in Cartesian space.
- **+AuxLoss2** To the base ELBO loss, we add the SSNR-weighted global MSE loss, the SSNR-weighted 7mer fragment MSE loss, the Distance MSE loss, and the Inter-residue Transform MSE loss.

Training For each of the model configurations in the ablation study, we trained on batch sizes of 32,000 residues by leveraging data parallelism across 8 V100 GPUs for $\sim 500,000$ steps, which is approximately ~ 1500 epochs and ~ 28 days of wall clock time. Models were trained with the Adam optimizer [132] and a learning rate of 2×10^{-4} with an initial linear warm-up phase of 10,000 steps. After each epoch, we evaluated one-sample estimates of ELBO and other losses across the full training and evaluation sets.

L.2 Ablation results

Likelihood analysis While it is clear that sample quality evaluations are very important for diffusion models generally [55] and also specifically in the case of protein generative models [91, 103], we first compare the different model variants from the point of view of likelihood. Likelihood measures have been broadly useful in deriving scoring functions for criticizing proteins and can, in certain instances, form a useful framework to make contact with free-energy quantities arising from statistical physics. We expect that models which behave well from the point of view of likelihood may also be useful as scoring functions to be used more broadly in protein design and modeling.

We visualize the trajectories of ELBO (Supplementary Appendix B) for the training and validation sets in Supplementary Figure 22. While the trajectories are smoothed with a 100-epoch moving average because they are noisy one-sample estimates per datapoint, there is a clear and consistent separation between the different model configurations. We make three observations:

First, there is a consistent improvement as measured by ELBO of the globular covariance models over the residue gas models. In some ways, this is to be expected from theory, because the information theoretic diffusion likelihood can be rewritten in terms of the bits accounted for by the prior plus additional corrections to account for non-Gaussianity [64]. Therefore a prior that better fits the data distribution, such as our globular covariance model that is based on the empirical scaling of real proteins, should do better as measured by likelihood even if the learned denoisers can account for a similar number of bits.

Second, we see similar but modest improvements in likelihood across the three different output layer parameterizations, where **PairFrameB** is favorable to **LocalFrame** which is favorable to **PairFrameA**. Thus we see evidence suggestive of favorable performance for our inter-residue geometry prediction over purely local prediction, though this can depend on tuning and is potentially confounded by the fact that **PairFrameB** also changes the output scaling at the same time. Optimizing the output layer will likely warrant further investigation.

Finally, we observe that adding auxiliary non-ELBO losses to otherwise purely ELBO-based training reduces ELBO performance.

Sample quality analysis. To evaluate each of the model configurations from the point of view of sample quality, we performed a large scale sample-and-refolding analysis. For each of the seven model configurations, we took five checkpoints from consecutive epochs around epoch 1100, sampled 1000 backbones per checkpoint with lengths uniformly distributed between 100 and 500 amino acids. We note that this epoch corresponds to $\sim 360,000$ training steps, which is approximately one quarter of the total training time of the `ChromaBackbone v0.4998` that was used in our broader refolding experiments. We expect that the total refolding rates reported in this section may be generally lower than our production model.

We observe large epoch-to-epoch fluctuations in secondary structure biases of the samples (Supplementary Figure 22, bottom center left). This is reminiscent of behaviors previously observed in other diffusion models [133], in which a batch of images may be all tinted one color, then another, even when the underlying denoising function is only changing slightly. These macroscopic fluctuations arising from microscopic changes may be intuitively understood as a tendency of the sampling process to amplify small per-time-step discrepancies. This phenomenon has previously been addressed by exponential moving averaging (EMA) of the checkpoints [62, 133], and we anticipate this is a worthwhile direction for future work.

Nevertheless, when we aggregate across checkpoints, we observe a few trends. All of the models trained with denoising losses that measure squared error in Cartesian space, which includes both the auxiliary loss models and the residue gas models, tend to have higher refolding rates than the models which were trained only with a chain whitened losses. This aligns with classical intuition on proteins in the sense that chained whitened coordinates emphasize local geometries in proteins while Cartesian coordinates much more directly measure the absolute positioning of coordinates in space that underlie contacts and interatomic distances. We also observe that the random graph neural networks have considerably higher folding rates than a purely k-NN based model, and that the best performing model overall combined our new diffusion and output parameterizations together with several new auxiliary loss functions. Thus, as has been a common lesson in the diffusion modeling literature, non-likelihood based losses or denoising weightings can be important to driving sample quality measures [55].

Low-temperature sampling remains essential All of the model configurations in this ablation study can generate samples which successfully refold and, in that sense, none of these changes qualitatively break model performance. We emphasize that the same cannot be said about low-temperature sampling, as all of these experiments were sampled with $\lambda = 10$. As shown in Supplementary Figure 2, low temperature sampling is important to generate high likelihood samples

which are sufficiently compact and structured to have a chance at refolding.

M Programmability: Conditioners framework

Overview In principle, the set of proteins satisfying a given set of functional constraints can be described using Bayes' Theorem,

$$p(\text{protein}|\text{function}) \propto p(\text{protein}) \times p(\text{function}|\text{protein})$$

where the **posterior** distribution of proteins $p(\text{protein}|\text{function})$ is proportional to the **likelihood**, i.e. the probability of satisfying the set of functional constraints $p(\text{function}|\text{protein})$ times the **prior** probability of the protein molecule being able to host function $p(\text{protein})$. This characterization has been appreciated for several decades [151], but leveraging it is challenging in practice for two reasons. First, developing tractable and accurate priors over the space of possible proteins has proven extremely difficult owing to the tremendous complexity in a single protein system (a complex can easily have $> 10^4$ atoms) and the intractabilities of marginalizing out low level details. Secondly, even with an accurate prior, sampling from the space of polypeptide conformations is highly difficult as it will typically involve a rugged landscape for which global optimization is infeasible.

One potential way to simplify the difficult inverse problem posed by protein design is given by contemporary methods from machine learning. In particular, diffusion models simplify conventionally intractable inverse problems by learning a sequence of distributions that gradually transform from a complex data distribution turns into a simple and tractable distribution [54, 85]. This has enabled transformative applications in text-to-image modeling [82, 83].

M.1 Bayes' theorem for score functions

Bayes' Theorem can be directly applied to Bayesian inversion with diffusion models where we can derive the time-dependent *posterior score* $\nabla_{\mathbf{x}} \log p_t(\mathbf{x}|\mathbf{y})$ as the sum of the original *prior score* $\nabla_{\mathbf{x}} \log p_t(\mathbf{x})$ and the *likelihood score* $\nabla_{\mathbf{x}} \log p_t(\mathbf{y}|\mathbf{x})$ as

$$\begin{aligned}
\nabla_{\mathbf{x}} \log p_t(\mathbf{x}|\mathbf{y}) &= \nabla_{\mathbf{x}} \log \frac{p_t(\mathbf{x})p_t(\mathbf{y}|\mathbf{x})}{p_t(\mathbf{y})} \\
&= \nabla_{\mathbf{x}} \log p_t(\mathbf{x}) + \nabla_{\mathbf{x}} \log p_t(\mathbf{y}|\mathbf{x}) - \cancel{\nabla_{\mathbf{x}} \log p_t(\mathbf{y})} \\
&= \nabla_{\mathbf{x}} \log p_t(\mathbf{x}) + \nabla_{\mathbf{x}} \log p_t(\mathbf{y}|\mathbf{x}).
\end{aligned}$$

This formulation can treat arbitrary combinations of conditions if we model the joint event $\mathbf{y}$ as factorizing into independent sub-events $\mathbf{y}_1, \ldots, \mathbf{y}_M$. Then we have the posterior score

$$\nabla_{\mathbf{x}} \log p_t(\mathbf{x}|\mathbf{y}_1, \ldots, \mathbf{y}_M) = \nabla_{\mathbf{x}} \log p_t(\mathbf{x}) + \sum_{i=1}^{M} \nabla_{\mathbf{x}} \log p_t(\mathbf{y}_i|\mathbf{x}).$$

These posterior scores can directly substitute the usual score function in the posterior SDE and ODE described in Appendix B.

Joint programmable sampling of sequence and structure While we focus on classifier conditioning of backbone structures throughout this work, it is also straightforward to extend the above picture to include joint gradient-based sequence and structure sampling by leveraging new discrete sampling methods based on locally gradient-adjusted MCMC proposals [125, 152]. It is an important distinction that *joint sequence-structure sampling at inference time does not require joint sequence-structure diffusion at training time*; all we require for joint sampling is access to a time-dependent *joint likelihood* $p_t(\mathbf{x}, \mathbf{s})$. Our current Chroma model satisfies this as $p_t(\mathbf{x}_t, \mathbf{s}_t) = p_t(\mathbf{x}_t)p_t(\mathbf{s}|\mathbf{x}_t)$, which may be leveraged as

$$\nabla_{\mathbf{x},\mathbf{s}} \log p_t(\mathbf{x}, \mathbf{s}|\mathbf{y}_1, \ldots, \mathbf{y}_M) = \nabla_{\mathbf{x},\mathbf{s}} \log p_t(\mathbf{x}, \mathbf{s}) + \sum_{i=1}^{M} \nabla_{\mathbf{x},\mathbf{s}} \log p_t(\mathbf{y}_i|\mathbf{x}, \mathbf{s}).$$

M.2 Conditioners: motivation

Motivation: Constraints versus Restraints Bayes' theorem can incorporate both soft restraints, which reweight the posterior but do not restrict its support, and hard constraints, which can completely eliminate certain regions of space. Hard constraints are just as useful and sometimes more natural than soft restraints in protein design, for example when conditioning on precise coordinates of a small molecule binding substructure or when exactly enforcing symmetries across large systems. Nevertheless, unconstrained gradient-based sampling algorithms such as Langevin dynamics or diffusion SDEs (Supplementary Appendix B) do not directly apply to constrained posteriors without special modifications. Here, we seek a framework that can support both restraints and constraints in concert with fully general sampling algorithms.

Requirements We propose four desiderata for a *programmable protein design* framework:

- **Compositionality.** Problems are expressed as *design programs* which are composed from "building blocks" encoding different required attributes.
- **Restraints.** Building blocks should be able to express soft restraints (e.g. classifier guidance) as a special case.
- **Constraints.** Building blocks should be able to express hard constraints, such as manifold constraints, as a special case.
- **Automatic sampling.** It should be feasible to automatically synthesize a valid sampling algorithm for any *design program* without requiring additional logic to be implemented by the user.

Design specifications as energy functions The Bayesian picture, as well as classical protein design approaches [151], formulate protein design problems in terms of *energy functions* which express the (unnormalized) negative log-posterior probability density of a protein system given a set of conditions. We can similarly cast posterior diffusions in terms of a time-dependent total

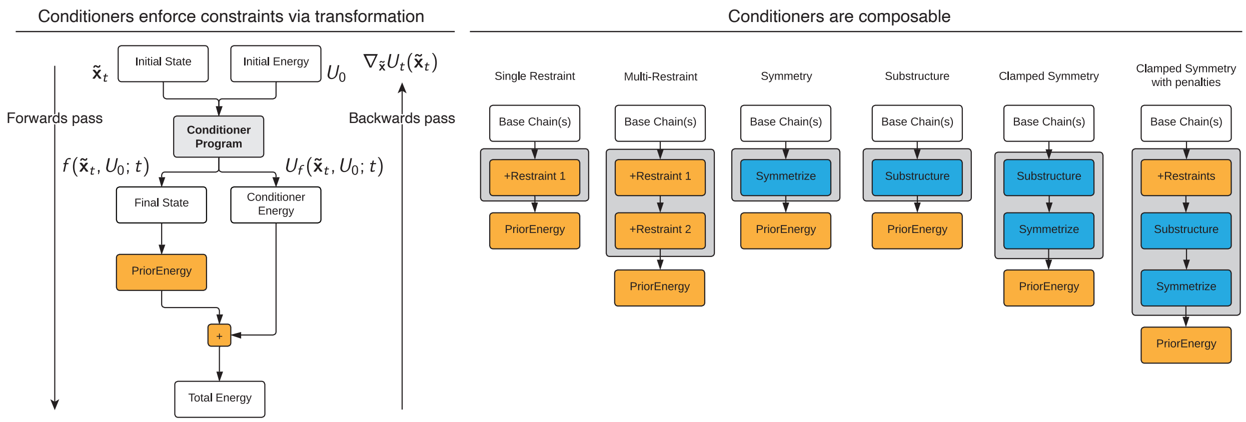

Supplementary Figure 23: **Conditioners parameterize protein design problems, facilitate automatic sampling algorithms, and are composable.** (Left) Conditioners are functions which map an unconstrained system consisting of an initial state $\tilde{\mathbf{x}}_t$ and energy $U_0 = 0$ to a transformed state $\mathbf{x}_t = f(\tilde{\mathbf{x}}_t, U_0; t)$ and an updated energy $U_f(\tilde{\mathbf{x}}_t, U_0; t)$. Gradient-based sampling with respect to unconstrained $\tilde{\mathbf{x}}_t$ on the Conditioner-adjusted Diffusion energy (left) will induce constrained dynamics on $\mathbf{x}_t$. Many kinds of restraints and constraints can be realized in this framework (right), and because of matched input-output types, simple Conditioners can be composed into complex Conditioners to jointly satisfy multiple design objectives within a complex protein design problem.

energy as

$$\begin{aligned} U(\mathbf{x}_t; \mathbf{y}, t) &= -\log p_t(\mathbf{x}_t) - \log p_t(\mathbf{y}|\mathbf{x}_t) + C_1 \\ &= \underbrace{\frac{1}{2}\left\|\sigma_t^{-1}\mathbf{R}^{-1}\left(\mathbf{x}_t - \alpha_t \hat{\mathbf{x}}_t\left(\mathbf{x}_t, t\right)\right)\right\|_2^2}_{\text{Diffusion Energy}} + \underbrace{\log p_t(\mathbf{y}|\mathbf{x}_t)}_{\text{Restraint Energy}} + C_2, \end{aligned}$$

where the gradient of the total energy with respect to $\mathbf{x}$ will yield the negative posterior score function[7].

Constraints via linear transformations How can we encode constraints such as symmetry and substructure? Many constraints, including these, can be enforced via affine transformation functions of the form $f(\tilde{\mathbf{x}}) = \mathbf{A}\tilde{\mathbf{x}} + \mathbf{b}$ which map points in unconstrained Euclidean space $\tilde{\mathbf{x}} \in \mathbb{R}^N$ to points in a constrained space $f(\tilde{\mathbf{x}}) \in \Omega \subseteq \mathbb{R}^M$. We can then run Langevin dynamics (Supplementary Appendix B) with the gradient of constrained energy $U(f(\tilde{\mathbf{x}}_t); \mathbf{y}, t)$ with respect to the unconstrained coordinate $\tilde{\mathbf{x}}_t$ as

$$\mathrm{d}\tilde{\mathbf{x}} = -\frac{g_t^2 \psi}{2} \lambda_t \mathbf{R}\mathbf{R}^\intercal \nabla_{\tilde{\mathbf{x}}} U(f(\tilde{\mathbf{x}}_t); \mathbf{y}, t)\, \mathrm{d}t + g_t \sqrt{\psi}\, \mathbf{R}\, \mathrm{d}\bar{\mathbf{w}}.$$

[7] We may choose to stop gradient flow through the denoiser model, which saves compute cost and recovers the behavior of the score functions from training time. This will lead to a non-conservative vector field (as is standard practice for diffusion models), but allowing gradients to flow through the denoiser restores energy conservation [153].

For variance-preserving schedule, the SDE reduces to

$$\mathrm{d}\tilde{\mathbf{x}} = -\frac{\beta_t \psi}{2} \lambda_t \mathbf{R}\mathbf{R}^\intercal \nabla_{\tilde{\mathbf{x}}} U(f(\tilde{\mathbf{x}}_t); \mathbf{y}, t)\ \mathrm{d}t + \sqrt{\beta_t \psi}\ \mathbf{R}\ \mathrm{d}\bar{\mathbf{w}}.$$

The constrained dynamics of $\mathbf{x}_t$ will then evolve according to the SDE[8]

$$\begin{aligned}
\mathrm{d}\mathbf{x} &= \mathbf{A}\mathrm{d}\tilde{\mathbf{x}} \\
&= \mathbf{A}\left(-\frac{\beta_t \psi}{2} \lambda_t \mathbf{R}\mathbf{R}^\intercal \nabla_{\tilde{\mathbf{x}}} U(f(\tilde{\mathbf{x}}_t); \mathbf{y}, t)\ \mathrm{d}t + \sqrt{\beta_t \psi}\ \mathbf{R}\ \mathrm{d}\bar{\mathbf{w}}\right) \\
&= -\frac{\beta_t \psi}{2} \lambda_t \mathbf{A}\mathbf{R}\mathbf{R}^\intercal \nabla_{\tilde{\mathbf{x}}} U(f(\tilde{\mathbf{x}}_t); \mathbf{y}, t)\ \mathrm{d}t + \sqrt{\beta_t \psi}\ \mathbf{A}\mathbf{R}\ \mathrm{d}\bar{\mathbf{w}} \\
&= -\frac{\beta_t \psi}{2} \lambda_t \mathbf{A}\mathbf{R}\mathbf{R}^\intercal \mathbf{A}^\intercal \nabla_{\mathbf{x}} U(\mathbf{x}_t; \mathbf{y}, t)\ \mathrm{d}t + \sqrt{\beta_t \psi}\ \mathbf{A}\mathbf{R}\ \mathrm{d}\bar{\mathbf{w}},
\end{aligned} \tag{3}$$

which is precisely Langevin dynamics with a modified mass matrix $(\mathbf{A}\mathbf{R}\mathbf{R}^\intercal\mathbf{A}^\intercal)^{-1}$ [154, 155] which will sample from the constrained domain Ω.

Nonlinear constraints: Exact sampling Many constraint sets cannot be expressed as the images of affine transformations [156]. One such example relevant to protein design is box constraints, where some subsets of atoms may be confined to contiguous finite regions of space. To enforce these constraints while still sampling from the intended energy function, we can simply design a nonlinear function f that implements the constraint and then adjust the total energy for sampling with the log-volume adjustment factor given by the multivariate change of variables formula: $\log\left|\det \frac{\partial f}{\partial \tilde{\mathbf{x}}}\right|$. This works so long as f is continuously differentiable and bijective onto the constrained space and the constrained space has the same dimension as the domain of f. It is further possible to extend this to also consider non-dimension-preserving transforms, e.g. with certain embedded Riemannian manifolds, for which we refer the reader to [157].

This transformed MCMC approach may be useful even when the nonlinear transformation function is fully unconstrained, for example, if it is a learned normalizing flow model of a particular class of structures of interest, in which case it will induce a dynamics similar to latent diffusion models [75].

Nonlinear constraints: Beyond If we are willing to sacrifice exact sampling from the true energy function, we may also discard the log-determinant adjustment and absorb the bias induced by running Langevin dynamics in a transformed space. These dynamics will still be exactly confined to the range of f, but may potentially be biased by change-of-volume effects as well as non-bijectivity. However, this opens up a large number of possibilities which are simple to implement by the user, as they only require a differentiable function f that implements the desired constraints which need not have an inverse and which can be differentiated by automatic differentiation. We have found this latter paradigm useful, as one can quickly realize more complex functionalities such as restricting sampling of subsets of a system to rigid body motions, to satisfying complex constraints such as optimal transport by differentiable inner optimization, and beyond.

[8] The first step can be justified by Ito's lemma.

M.3 Conditioners

The previously described restraints and constraints for Langevin dynamics share a common form of implementation: they modify the system coordinates $\mathbf{x}$ and/or the total energy U. This suggests a natural "building block" for a protein programming framework: *transformation functions* which input and output system states $(\mathbf{x}, U)$.

We define a **conditioner** as a function $\mathcal{F} : \mathbb{R}^N \times \mathbb{R} \to \Omega \subseteq \mathbb{R}^M \times \mathbb{R}$ which maps state-energy pairs in unconstrained input space $\mathbb{R}^N \times \mathbb{R}$ to potentially constrained state-energy pairs in $\Omega \subseteq \mathbb{R}^M \times \mathbb{R}$. For ease of notation, we further refer to Conditioners component-wise $\mathcal{F} = (f, U_f)$ in terms of a **state update function** $f : \mathbb{R}^N \times \mathbb{R} \to \Omega_f \subseteq \mathbb{R}^M$ and an **energy update function** $U_f : \mathbb{R}^N \times \mathbb{R} \to \Omega_U \subseteq \mathbb{R}$.

Conditioned Diffusion To sample from Conditioner-biased diffusion problems, we will use a gradient-based sampling algorithm, such as Langevin dynamics or Hamiltonian Monte Carlo, on the Conditioner-transformed instance of the energy

$$U(\tilde{\mathbf{x}}_t; U_f, f, t) = \underbrace{\frac{1}{2} \left\| \sigma_t^{-1} \mathbf{R}^{-1} \left(f(\tilde{\mathbf{x}}_t, U_0; t) - \alpha_t \hat{\mathbf{x}}_t \left(f(\tilde{\mathbf{x}}_t, U_0; t), t \right) \right) \right\|_2^2}_{\text{Diffusion Energy}} + \underbrace{U_f(\tilde{\mathbf{x}}_t, U_0; t)}_{\text{Conditioner Energy}},$$

where the gradient $\nabla_{\tilde{\mathbf{x}}} U(\tilde{\mathbf{x}}_t; U_f, f, t)$ for sampling is computed with respect to the unconstrained coordinates $\tilde{\mathbf{x}}_t$. These gradients and dynamics can be computed efficiently even for complex composed conditioners by leveraging modern automatic differentiation frameworks, as shown in Supplementary Figure 23.

Desiderata The **Conditioner** formulation satisfies all of our desiderata:

- **Compositionality.** Let $\mathcal{F}_1 : \mathbb{R}^{N_1} \times \mathbb{R} \to \Omega_1 \subseteq \mathbb{R}^{M_1} \times \mathbb{R}$ and $\mathcal{F}_2 : \mathbb{R}^{N_2} \times \mathbb{R} \to \Omega_2 \subseteq \mathbb{R}^{M_2} \times \mathbb{R}$ be Conditioners and assume $N_1 = M_2$[9]. Then $\mathcal{F}_3 = \mathcal{F}_1 \circ \mathcal{F}_2$ is a Conditioner with $\mathcal{F}_3 : \mathbb{R}^{N_2} \times \mathbb{R} \to \Omega_1 \subseteq \mathbb{R}^{M_1} \times \mathbb{R}$.
- **Restraints.** Generalized restraints may be realized with state update $f(\mathbf{x}, U) = \mathbf{x}$ (Identity function) and energy update $U_f(U, \tilde{\mathbf{x}}_t, t) = U - \log p(\mathbf{y}|\mathbf{x}, t)$.
- **Constraints: Linear Transforms.** Distribution-preserving linear transform constraints may be realized with state update $f(\mathbf{x}, U) = \mathbf{A}\mathbf{x} + \mathbf{b}$ and energy update $U_f(U, \tilde{\mathbf{x}}_t, t) = U$ (Identity function).
- **Constraints: Non-Linear Transforms.** Distribution-preserving nonlinear domain constraints may be realized with bijective and differentiable state update $f : \mathbb{R}^N \times \mathbb{R} \to \Omega_f \subseteq \mathbb{R}^M$ and energy update $U_f(U, \tilde{\mathbf{x}}_t, t) = U + \log \det \left| \frac{\partial f}{\partial \tilde{\mathbf{x}}} \right|$ (Change of volume adjustment).
- **Automated Sampling.** Any gradient-based sampling algorithm may be used in concert with the Conditioner-adjusted energy and an annealing schedule on the diffusion time t.

[9]Composition of blocks will require that their inputs and outputs can be shape-compatible, just as in the case of composing differentiable blocks in neural networks. For example, two substructure constraints by definition must be expressed in a way that can be jointly realized with one set of protein chain lengths.

Conditioners for sequence and structure As noted in the previous section, the Conditioner framework is also straightforwardly applied to joint sampling of sequence and structure, where we define the joint energy

$$U(\mathbf{x}_t;\mathbf{y},t) = \underbrace{\frac{1}{2}\left\|\sigma_t^{-1}\mathbf{R}^{-1}\left(f(\tilde{\mathbf{x}}_t,U_0;t) - \alpha_t\hat{\mathbf{x}}_t\left(\mathbf{x}_t,t\right)\right)\right\|_2^2}_{\text{Diffusion Energy}} - \underbrace{\log p(f_s(\tilde{\mathbf{s}}_t))|f_x(\mathbf{x}_t),t)}_{\text{Sequence Likelihood}} + \underbrace{U_f(\tilde{\mathbf{x}}_t,\tilde{\mathbf{s}}_t,U_0,t)}_{\text{Conditioner Energy}},$$

where gradient and dynamics are computed in unconstrained space $\tilde{\mathbf{x}}_t, \tilde{\mathbf{s}}_t$ and we can use approaches such as Discrete Langevin sampling [125, 152] to sample from sequence space while leveraging gradients for building locally-informed proposals. Sequence and structure gradients can be computed in one pass via automatic differentiation frameworks.

Thus, we can perform joint sequence and structure sampling conditioned on a target objective without needing to train a joint diffusion on sequence and structure at the same time; all we require is a valid *joint posterior for sequence and structure conditioned on function* which may be realized, for example, with a conditional language model for sequence given structure together with a diffusion model for the backbone structure joint marginal.

M.4 Example applications of constraint composition

We list the composable Conditioners explored in this work in Supplementary Table 6. Some practical protein design problems that might be realized as composite constraints under this framework are

***De-novo* binders** Combine (i) substructure conditioning on antigen, (ii) optional scaffold constraint on binder, and (iii) contact constraints on epitope/paratope.

Enzyme miniaturization Use motif conditioning to graft an active site into a novel scaffold or known scaffold (e.g. via combining with a substructure conditioner).

Nanostructure control Use combinations of shape and substructure conditioners to sample novel designable folds or complexes satisfying nanometer-scale target geometries.

Nanomaterial design Combine symmetry conditioners or shape conditioners control with substructure condtioners to produce functionalized nanomaterials with periodic structure.

M.5 Related work

Energy functions for specifying multi-objective design problems have a long history in and out of protein design, which we do not attempt to review here. Concurrent with this work, [158] proposed a framework for programmatic design of proteins by introducing a grammar for problem specifications which can be compiled into deep energy functions that are sampled via annealed MCMC in sequence space. Some advantages of our Conditioners framework include that it admits efficient gradient-based sampling, that it can exactly enforce hard constraints on continuous degrees of freedom during sampling, and that it supports the fast-convergence properties of diffusion annealing which “tunnels” from a unimodal $t = 1$ base distribution into the multimodal $t = 0$ target posterior. Beyond protein design, the idea of using MCMC with diffusion models to sample from complex

**Conditioner**	$f(\tilde{\mathbf{x}}, U, t)$	$U_f(\tilde{\mathbf{x}}, U, t)$	**Examples and applications**
Symmetry constraint	$\mathbf{G}\tilde{\mathbf{x}}$	U	Large assemblies
Substructure constraint	$\bar{\mathbf{R}}\mathbf{R}^{-1}\tilde{\mathbf{x}} + \bar{\mu}$	$U + \lVert \hat{\mathbf{x}}_\theta(f(\tilde{\mathbf{x}}, U, t), t)^{\mathcal{M}} - \mathbf{x}_t^{\mathcal{M}} \rVert_2^2$	Substructure grafting
Substructure distances	$\tilde{\mathbf{x}}$	$U - \log p_t(d_{ij} \vert \tilde{\mathbf{x}})$	Interface and contact constraints
Substructure motif	$\tilde{\mathbf{x}}$	$U + \eta \log\left(1 + e^{\zeta[\rho(\mathbf{x}_t) - \rho_{max}]}\right)$	Motif-conditioned scaffolds
Shape constraint	$\tilde{\mathbf{x}}$	$U + \mathrm{ShapeLoss}_t(\mathbf{x}, \mathbf{r})$	Molecular shape control
Sequence	$\tilde{\mathbf{x}}$	$U - \log p_t(\mathrm{sequence} \vert \tilde{\mathbf{x}})$	Sequence constraints
Secondary structure	$\tilde{\mathbf{x}}$	$U - \log p_t(\mathrm{ss} \vert \tilde{\mathbf{x}})$	Topological constraints
Domain classification	$\tilde{\mathbf{x}}$	$U - \log p_t(\mathrm{domain} \vert \tilde{\mathbf{x}})$	Pfam, CATH, Taxonomy
Text caption	$\tilde{\mathbf{x}}$	$U - \log p_t(\mathrm{caption} \vert \tilde{\mathbf{x}})$	Natural language prompting
Likelihood restraint	$\tilde{\mathbf{x}}$	$U - \log p_t(\cdot \vert \tilde{\mathbf{x}})$	Biasing towards specifications
Linear constraint	$\mathbf{A}\tilde{\mathbf{x}} + \mathbf{b}$	U	Exactly enforcing specifications
Nonlinear constraint	$f(\tilde{\mathbf{x}})$	$U + \log\det \frac{\mathrm{d}f}{\mathrm{d}\tilde{\mathbf{x}}}$	Exactly enforcing specifications

Supplementary Table 6: **Conditioners for Chroma.**

composed energy functions was explored in [84], which also presents useful tools for negation and other primitive composition operations.

Our framework introduces a versatile conditioning mechanism that accommodates additional modalities such as natural languages [75] and 3D densities [159], allowing users to designate either parametric or non-parametric classifiers as restraint energies. Moreover, it facilitates a composable structure for sampling within constrained domains and manifolds, bearing a resemblance to MCMC methods for structured spaces. In our methodology, we initiate sampling from an unconstrained space, followed by mapping these samples onto the corresponding constrained space. This mapping procedure echoes the ideas of Mirror Langevin Dynamics [160, 161], where the constrained transformation operates as a ”mirror map”. For sampling linear subspaces, our approach recovers preconditioned Langevin Dynamics [155], achieved via mass matrix conditioning [154].

N Programmability: Substructure Constraints

N.1 Motivation

Many protein design tasks including imputation of missing structural data, redesign of an enzyme scaffold given an active site, and redesign of the CDRs of a known antibody framework require exact specification of the known structural coordinates. In this section, we describe a method that allows for such specification as a hard constraint on the reverse diffusion trajectories.

We began by exploring methods for substructural conditioning that bias sampling by adding a conditional score term $\nabla \log_{\mathbf{x}} p_t(\mathbf{y}|\mathbf{x})$ to the drift component in the reverse SDE (Appendices P O). In practice, we found that these methods do not always result in samples that satisfy the condition $\mathbf{y}$ exactly. Often to enforce $\mathbf{y}$ in these regimes one must upweight the conditional score relative to the prior score function which can result in a reduction in the likelihood (or ELBO) of the samples drawn, or even in numerical instability.

The method presented below is motivated by the approach described in [154] where the equilibrium

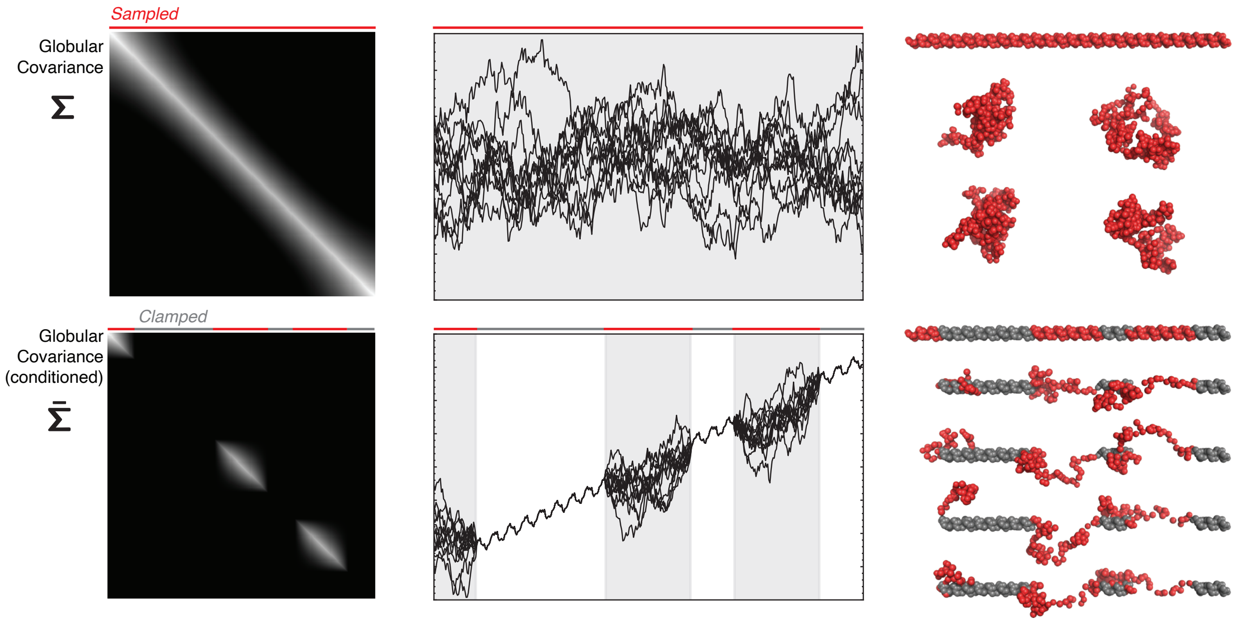

Supplementary Figure 24: **The globular covariance model admits analytic conditioning** (Left) Heatmaps illustrating comparison of unconditional (top) globular covariance matrix $\mathbf{R}\mathbf{R}^\intercal$ and conditioned (bottom) covariance matrix $\bar{\mathbf{R}}\bar{\mathbf{R}}^\intercal$. (Middle) X-coordinate plotted against residue index of samples drawn from unconditional (top) and conditional (bottom) prior. (Right) Initial samples X_0 and noised samples drawn from $p(X_1|X_0)$ for the unconditional (top) and conditional (bottom) priors. Conditioned-on structural residues are drawn in gray and correspond to the same residues that are conditioned in the covariance matrix and line plot.

states of a system are sampled by simulating the dynamics of an auxiliary system with a modified mass matrix. If the mass matrix is chosen appropriately, the original system's configuration space can be sampled more efficiently.

The method works by integrating a modified Annealed Langevin Dynamics SDE (see appendix C) backwards in time to sample from $p_0(\mathbf{x}|\mathbf{x_1})$, where the dynamics are modified using a mass matrix that assigns higher mass to particles closer (in chain distance) to known coordinates and assigning infinite mass to known atoms. Samples drawn using this method satisfy $\mathbf{y}$ with probability 1.

Let $\mathcal{S}, \mathcal{M} \subset [1, \cdots, N]$ denote the atoms comprising the unknown scaffold and known motif, respectively, throughout this section.

N.1.1 Related work

Song *et al.* [55] present a replacement method for drawing approximate conditional samples from $p(\mathbf{x}_0^{\mathcal{S}}|\mathbf{x}_0^{\mathcal{M}})$ in which one samples a sequence of noised motifs $\bar{\mathbf{x}}_{1:T}^{\mathcal{M}} \sim q(\mathbf{x}_{1:T}^{\mathcal{M}}|\mathbf{x}_{(0)}^{\mathcal{M}})$, then runs diffusion backward in time but at each time step replacing $\mathbf{x}_t^{\mathcal{M}} \leftarrow \mathbf{x}_t^{\mathcal{M}}$ before sampling $\mathbf{x}_{t-1} \sim p(\mathbf{x}_{t-1}|\mathbf{x}_t)$. [91] demonstrated that this method introduces an irreducible error that is exacerbated by the correlation introduced by q and proposes a particle filtering-based approach that furnishes arbitrarily accurate conditional samples given sufficient computation. Informally, the

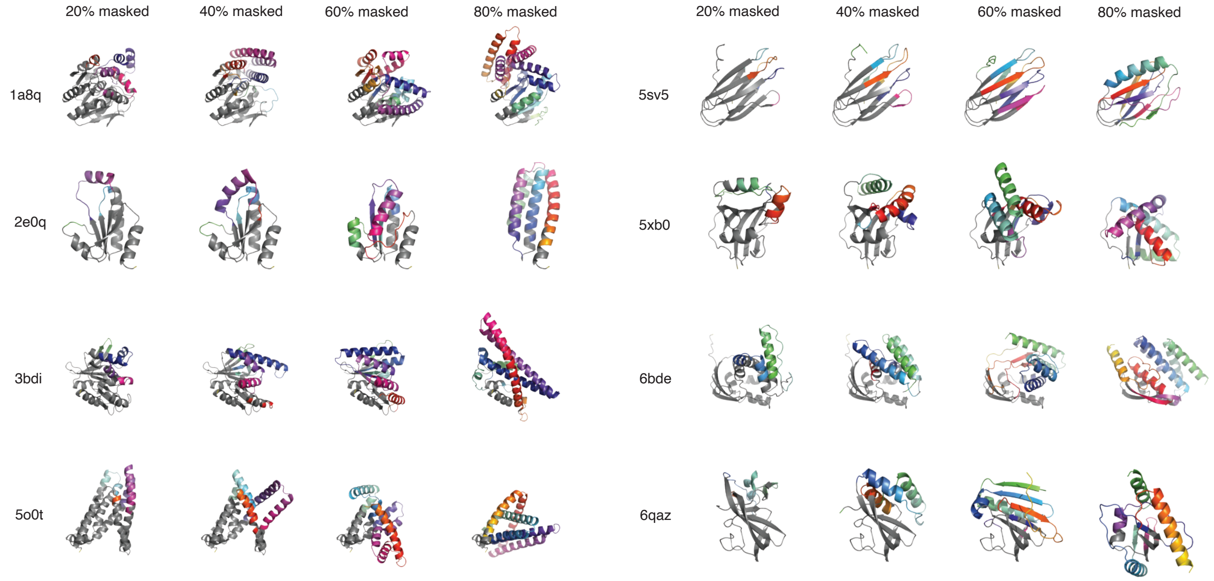

Supplementary Figure 25: **Examples of substructure-conditioned Chroma samples** Example conditional samples drawn by conditioning on substructures of 8 PDBs sampled from the Chroma test split. Conditioned-on portions of the structure are defined by splitting the protein by a plane normal to the first principal component of the atom coordinates and are drawn in grey. The plane is shifted to condition on a specified fraction of the residues for each column.

error introduced by the replacement method arises from imputing noised motifs that are highly unlikely given the corresponding noised scaffold.

N.2 Approach

It is known that for $\mathbf{x} \sim \mathcal{N}(\mu, \Sigma)$, if we partition the coordinates as above into subsets $\mathcal{M}, \mathcal{S}$ and follow the Gaussian conditioning formula to write $\mathbf{x} = \begin{bmatrix} \mathbf{x}^{\mathcal{S}} \\ \mathbf{x}^{\mathcal{M}} \end{bmatrix}$ with $\mu = \begin{bmatrix} \mu^{\mathcal{S}} \\ \mu^{\mathcal{M}} \end{bmatrix}$ and $\Sigma = \begin{bmatrix} \Sigma_{\mathcal{SS}} & \Sigma_{\mathcal{SM}} \\ \Sigma_{\mathcal{MS}} & \Sigma_{\mathcal{MM}} \end{bmatrix}$ such that $(\mathbf{x}^{\mathcal{S}} | \mathbf{x}^{\mathcal{M}} = \mathbf{a}) \sim \mathcal{N}(\bar{\mu}, \bar{\Sigma})$ where

$$\bar{\mu} = \mu^{\mathcal{S}} + \Sigma_{\mathcal{SM}} \Sigma_{\mathcal{MM}}^{-1} (\mathbf{a} - \mu^{\mathcal{M}})$$

and

$$\bar{\Sigma} = \Sigma_{\mathcal{SS}} - \Sigma_{\mathcal{SM}} \Sigma_{\mathcal{MM}}^{-1} \Sigma_{\mathcal{MS}},$$

where inverse matrices are understood to denote pseudo-inverses. We also compute the Cholesky factorization $\bar{\mathbf{R}}\bar{\mathbf{R}}^{\intercal} = \bar{\Sigma}$. To draw an approximate conditional sample from $p(\mathbf{x}_0^{\mathcal{S}} | \mathbf{x}_0^{\mathcal{M}} = a)$ we proceed as follows: we sample $\mathbf{x}_1^{\mathcal{S}} \sim \mathcal{N}(\bar{\mu}, \bar{\Sigma})$ from the conditional prior, set $\mathbf{x}_1^{\mathcal{M}} = a$, and integrate a modified Annealed Langevin Dynamics SDE (see section C.2)

$$\mathrm{d}\mathbf{x} = -\frac{g_t^2 \psi}{2} \bar{\mathbf{R}} \bar{\mathbf{R}}^\intercal \nabla_{\mathbf{x}} \log p_t(\mathbf{x})^{\lambda_0} \, \mathrm{d}t + g_t \sqrt{\psi} \, \bar{\mathbf{R}} \, \mathrm{d}\bar{\mathbf{w}}$$

backwards in time, where the matrices $\bar{\mathbf{R}}, \bar{\mathbf{R}}^\intercal$ are broadcast to the correct size with the conditioned on rows and columns filled by zeroes. Supplementary Figure 24 illustrates $\bar{\mathbf{R}}, \bar{\mathbf{R}}^\intercal$ as well as samples from a conditional prior.

Additionally, we have found it helpful to incorporate a reconstruction-guidance based gradient term as in [162]. We have found that, while this can introduce some instability to the sampling, it improves sample quality. To do so, in our conditioner formulation we define

$$U_f(\tilde{\mathbf{x}}_t, U, t) = U + \|\hat{\mathbf{x}}_\theta(\mathbf{x}_t, t)^{\mathcal{M}} - \mathbf{x}_t^{\mathcal{M}}\|_2^2,$$

where

$$\mathbf{x}_t = f(\tilde{\mathbf{x}}_t) = \mathbf{A}\tilde{\mathbf{x}}_t + \mathbf{b} = \bar{\mathbf{R}}\mathbf{R}^{-1}\tilde{\mathbf{x}}_t + \bar{\mu}.$$

See section M.2 for a derivation that under this f, evolving $\tilde{\mathbf{x}}$ according to the *unmodified* Annealed Langevin SDE induces dynamics on $\mathbf{x}_t$ equivalent to the mass-modified dynamics presented above.

O Programmability: Substructure Distances

O.1 Motivation

In some instances, it may be useful to generate diverse protein chains or complex structures under the constraints that one or more specific residue pairs be in spatial proximity (i.e., form a "contact"). Such a conditioner could be used, for example, to design binders by ensuring that the desired binding site is being engaged. Or it could be used to enforce some desired topological properties–i.e., the proximity of N- and C-termini (e.g., for ease of circular permutation). Assuming that we are interested in conditioning on a contact between atoms i and j within the diffusion conditioning framework, we wish to compute the probability that the distance between two atoms in the fully denoised structure D_0^{ij} is below a desired cutoff c, i.e. $D_0^{ij} < c$, given a noised sample at time t and the corresponding distance d_t^{ij}.

O.2 Approach

The Bayesian approach to diffusion conditioning approach would be to build an estimate of the time-dependent likelihood $p_t(\mathbf{y}|\mathbf{x}(t))$ to classify noisy inputs. In the case of a contact classifier, we can build an analytic approximation for $p_t(D_0^{ij} < c|\mathbf{x}_t)$ as follows. First, we choose a prior $p(\mathbf{x}_0)$ that captures distance statistics in the PDB giving rise to an tractable posterior denoising distribution $p(\mathbf{x}_0|\mathbf{x}_t)$. With a Gaussian prior for $\mathbf{x}_0$, which we can use our globular covariance model for, we arrive at a Gaussian posterior for $p(\mathbf{x}_0|\mathbf{x}_t)$ and can further model the posterior distances $p(D_0^{ij}|\mathbf{x}_t)$ with a non-central chi-squared distribution. This allows us to compute the desired $p(D_0^{ij} < c|\mathbf{x}_t)$ using the CDF of the non-central chi-squared distribution.

First, we can build a Gaussian approximation of a prior for protein chains $p(\mathbf{x}_0)$ with our globular covariance model (Appendix D.3) as

$$p(\mathbf{x}_0) \sim \mathcal{N}(\mathbf{0}, \mathbf{R}\mathbf{R}^\intercal).$$

Then, according to our forward process, we have a forwards transition kernel for the likelihood as

$$p(\mathbf{x}_t|\mathbf{x}_0) \sim \mathcal{N}(\alpha_t \mathbf{x}_0, \sigma_t^2 \mathbf{R}\mathbf{R}^\intercal).$$

We can now apply Bayes' Theorem as

$$\begin{aligned} p(\mathbf{x}_0|\mathbf{x}_t) &\propto \mathcal{N}(\mathbf{x}_0; \mathbf{0}, \mathbf{R}\mathbf{R}^\intercal)\ \mathcal{N}(\mathbf{x}_t; \alpha_t \mathbf{x}_0, \sigma_t^2 \mathbf{R}\mathbf{R}^\intercal) \\ &\propto \mathcal{N}(\mathbf{x}_0; \mathbf{0}, \mathbf{R}\mathbf{R}^\intercal)\ \mathcal{N}\left(\mathbf{x}_0; \frac{\mathbf{x}_t}{\alpha_t}, \frac{\sigma_t^2}{\alpha_t^2}\mathbf{R}\mathbf{R}^\intercal\right) \\ p(\mathbf{x}_0|\mathbf{x}_t) &= \mathcal{N}(\mathbf{x}_0; \alpha_t \mathbf{x}_t, \sigma_t^2 \mathbf{R}\mathbf{R}^\intercal). \end{aligned}$$

We can therefore express a sample from the posterior $\mathbf{x}_0 \sim p(\mathbf{x}_0|\mathbf{x}_t)$ as

$$\mathbf{x}_0 = \alpha_t \mathbf{x}_t + \sigma_t \mathbf{R}\mathbf{z},$$

where $\mathbf{z} \sim \mathcal{N}(\mathbf{0}, \mathbf{I})$. Assuming $j > i$, we have

$$\mathbf{x}_0^j - \mathbf{x}_0^i = \alpha_t(\mathbf{x}_t^j - \mathbf{x}_t^i) + \sigma_t([\mathbf{R}\mathbf{z}]_j - [\mathbf{R}\mathbf{z}]_i).$$

From the R_g scaling analysis of the globular covariance model (Supplementary Appendix D.3) we have

$$\begin{aligned} \sigma_{ij}^2 &\triangleq \mathrm{Var}([\mathbf{R}\mathbf{z}]_j - [\mathbf{R}\mathbf{z}]_i)) \\ &= \frac{2a^2(1-b^{j-i})}{1-b^2}, \end{aligned}$$

and therefore the inter-atomic residual will be Gaussian distributed as

$$\frac{\mathbf{x}_0^j - \mathbf{x}_0^i}{\sigma_t \sigma_{ij}} \sim \mathcal{N}\left(\frac{\alpha_t(\mathbf{x}_t^j - \mathbf{x}_t^i)}{\sigma_t \sigma_{ij}}, \mathbf{I}\right).$$

The squared inter-atomic distance is a squared 2-norm of this residual, which will therefore follow a non-central Chi Squared distribution with 3 degrees of freedom as

$$\left\| \frac{\mathbf{x}_0^j - \mathbf{x}_0^i}{\sigma_t \sigma_{ij}} \right\|_2^2 = \frac{(D_0^{ij})^2}{\sigma_t^2 \sigma_{ij}^2} \sim \text{NonCentralChiSquared}\left(\frac{\alpha_t (d_t^{ij})^2}{\sigma_t^2 \sigma_{ij}^2}, k=3\right).$$

We can therefore apply distance restraints by adjust the total energy by

$$\log p(D_0^{ij} < C|\mathbf{x}_t, t) = \log\left(1 - Q_{\frac{3}{2}}\left(\frac{\sqrt{\alpha_t} d_t^{ij}}{\sigma_t \sigma_{ij}}, \frac{C}{\sigma_t \sigma_{ij}}\right)\right),$$

where Q is the Marcum Q-function.

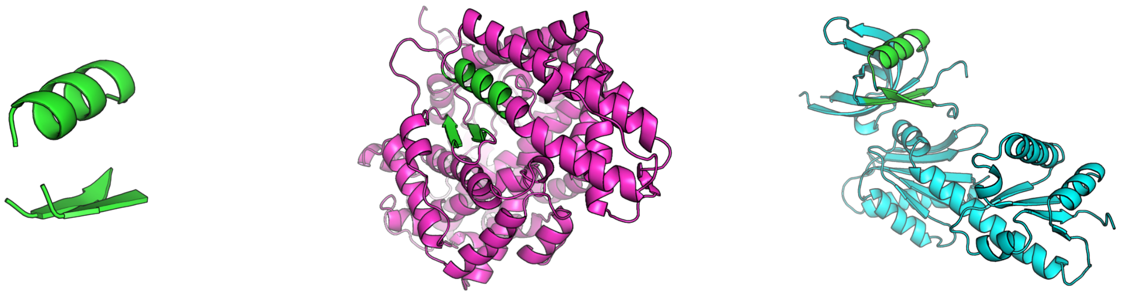

(a) (b) (c)

Supplementary Figure 26: **Motifs can occur in entirely unrelated structural contexts.** **a,** An example motif composed of three disjoint segments. **b,** PDB entry 3NXQ harbors the motif with a backbone RMSD of 0.45 *Å*. **c,** PDB entry 3OBW harbors the motif with a backbone RMSD of 0.64 *Å*.

P Programmability: Substructure Motifs

P.1 Motivation

It would be very useful for a variety of protein engineering applications to condition structure generation on the presence of a particular structural "motif." By this we mean an arbitrary substructure, composed of any number of disjoint backbone segments, that we would like to exist within our final generated structure. In practice, such a motif could represent a functional constellation of residues or a metal/small-molecule binding site—this could be useful for designing enzymes or other functional proteins, by exploring ideas around a core functional mechanism. In another example, the motif could correspond to a "scaffolding" part of the molecule that we would want to preserve—e.g., the binding scaffold that can admit different loop conformations. Or the motif could represent a desired epitope that we would like to faithfully present on the surface of a generated protein in the context of vaccine design. Fig. 26 shows an example motif and two unrelated native protein structures in which this motif is found with low RMSD.

P.2 Approach

To determine whether a pre-specified motif is present within a given structure S is simple–one can, for example, find the substructure of S with the lowest optimal superposition root-mean-squared-deviation (RMSD) to the motif and ask whether this RMSD value is below a desired cutoff; this can be done using previously published algorithms [163, 164]. To enable conditional generation based on the presence of a motif then, we employ a form of reconstruction guidance based on the best RMSD to the motif in the present de-noised structure. Specifically, at time t we define the best-match RMSD to the target motif with coordinates $\mathbf{x}^{\mathcal{M}}$ as

$$\rho\left(\mathbf{x}_t\right) = \min_{\pi \in \Pi} \min_{\mathbf{T} \in \mathrm{SE}(3)} \frac{\|\mathbf{x}_t^{\mathcal{M}} - \mathbf{T} \circ \hat{\mathbf{x}}_\theta\left(\mathbf{x}_t, t\right)^{\mathcal{M}_\pi}\|_2}{\sqrt{|\mathcal{M}_\pi|}}, \tag{4}$$

where the outer minimization is over the combinatorial space Π of alignment permutations π of motif disjoint segments onto the current structure $\mathbf{x}_t$ and the inner minimization is over the optimal

superposition of the motif given a specific alignment π. The actual calculation is done using a branch-and-bound search similar to the one defined in Zhou *et al.* [163] rather than an explicit minimization over permutations. With this, we then modify the energy within our conditioner formulation (see section M) as

$$U_f(\mathbf{x}_t, U, t) = U + \eta \log\left(1 + e^{\zeta[\rho(\mathbf{x}_t) - \rho_{max}]}\right),$$

where ρ_{max} is the threshold RMSD below which we desire to find the motif in the final generated structure, and η and ζ are parameters (we used $\eta = 50$ and $\zeta = 4$ in this work). With this modification, auto-differentiation of the resulting energy to obtain the score function creates gradients that pull the system towards containing the motif in question. Note that the location of the motif within the generated structure needs not be specified, as equation 4 optimizes over all possible alignments at each step of the reverse diffusion. On the other hand, it is also easy to introduce constraints to the possible matching alignments, such as either relative constraints on the mapping of individual segments of the motif (e.g., first and second segments must be separated by anywhere between 3 and 20 residues) or absolute constraints on the location of the motif (e.g., first segment must match in the first 100 residues of the generated structure). This can be easily accommodated by modifying the parameters of the search in equation 4 as shown previously [163].

Q Programmability: Symmetry

Q.1 Motivation

The functions of many proteins are often realized through self-assembly into large higher-order structures. One of the most powerful and widely employed tools for this in nature is symmetric assembly, observed in everything from large membrane pores that gate transfer of materials in and out of cells to icosahedral viral capsids which can encapsulate an entire genetic payload [165]. Similarly, incorporating symmetry into computational design of proteins holds great promise for building large functional complexes [166]. To realize this potential within our diffusion framework, we propose a method to directly constrain sampling to any prescribed discrete Euclidean symmetries.

Related work Incorporating group equivariance in machine learning has been an important topic in the machine learning community [167]. Incorporating symmetries is critical in molecular simulations [168, 169]. In this work, we propose a method for incorporating symmetry for point set sampling with applications in the generation of large-scale protein complexes with arbitrary discrete symmetry groups.

Q.2 Symmetry breaking in sampling

Group theory lays the foundation for describing symmetries in mathematics, physics, and biology. [170–172] Let $G = [g]_{i=0}^{M}$ be a collection of symmetry operations that form a group such as point groups and space groups. For point sets in $\mathbb{R}^3$, these symmetry operations can be represented as a set of orthogonal transformations, i.e. rotations and/or reflections.

To generate symmetric protein complexes, we want to sample structures $\mathbf{x} \in \mathbb{R}^{M \times N \times 3}$ that are built from $M = |G|$ identical single chain proteins $\mathbf{x} \in \mathbb{R}^{N \times 3}$ where N is the number of residues for each subunit. The SDE solving process produces final samples with:

$$\mathbf{x}_0 = \texttt{sdesolve}(\mathbf{x}_T)$$

For sample generation to respect symmetries for an arbitrary group G, the SDE/ODE dynamics needs to be G-invariant up to a permutation of subunits. Let $\cdot$ represent the symmetric operations (rotation, reflection, and translation) performed on point sets in $\mathbb{R}^3$, we define the sampling procedure $\texttt{sdesolve} : \mathbb{R}^{M \times N \times 3} \to \mathbb{R}^{|G| \times n \times 3}$ with $\mathbf{x}_0 = \texttt{sdesolve}(\mathbf{x}_T)$ being the desired samples. The sampling procedure should follow the following G-invariance condition:

$$\texttt{sdesolve}(g\mathbf{x}_T) = g\texttt{sdesolve}(\mathbf{x}_T) = \sigma(g)\,\texttt{sdesolve}(\mathbf{x}_T)\;, \forall g \in G$$

where g indicates a group element in G and we impose an arbitrary order on G and our method is equivariant to the permutation of subunits. $\sigma(g)$ is the induced permutation operation that satisfies the relation: $g\mathbf{G} = \sigma(g)\mathbf{G}$, as computed from the group multiplication table (also called the Caley table).

The first equality in appendix Q.2 is trivially satisfied if $f(\cdot)$ or the underlying gradient update is $\mathrm{E}(3)$ equivariant, as G consists of only orthogonal transformations and translations. However, the second equality is generally not satisfied. For molecular simulations where Hamiltonian dynamics is used, the second equality can be satisfied if (i) the energy function is $\mathrm{E}(3)$ invariant, and (ii) the initial $\mathbf{x}_T$ and $\frac{\mathrm{d}\mathbf{x}_T}{\mathrm{d}t}$ are symmetric, i.e $g\mathbf{x}_T = \sigma(g)\,\mathbf{x}_T, g\frac{d\mathbf{x}_T}{dt} = \sigma(g)\,\frac{d\mathbf{x}_T}{dt}$. At each successive time step, x_t automatically satisfies the prescribed G-symmetry. This approach confines both the position and momentum update to ensure that the sampled configurations remain symmetric.

However, this is not the case for SDE/ODE sampling in our framework. We list three origins of the symmetry-breaking error if appendix Q.2 is used: (i) the denoising network uses distances as features and is automatically $\mathrm{E}(3)$ equivariant. However, because protein feature graphs are generated probabilistically, each subunit protein has a different geometric graph, despite the protein's overall symmetry. (ii) Our polymer structured noise is randomly sampled from $\mathcal{N}(\mathbf{x}_T; \mu, \Sigma)$, so each subunit protein has different chain noise. (iii) The sampling procedure requires solving an ODE/SDE which is vulnerable to accumulated integration error. Integration error can induce unwanted geometric drifts such as rotation and translation[173], and be a substantial symmetry breaking force.

Q.3 Symmetric transformation as a conditioner

Basic case. We propose the symmetric sampling approach as a constrained transformation formalism implemented as a conditioner block, as delineated in the referenced literature. Using the representations of G, we demonstrate the building of protein symmetric assemblies from an asymmetric unit (AU) chain $\tilde{\mathbf{x}}$ through symmetrization. We commence with the mathematical formulation of the transformation, subsequently elucidating the induced linear transformation on the intrinsic gradient dynamics.

Representing G as $M \times 3 \times 3$ rotation matrices $\mathbf{G}$, we define the constrained transformation as

$$\mathbf{x}_t = f\left(\tilde{\mathbf{x}}_t, t\right) = \texttt{symmetrize}\left(\tilde{\mathbf{x}}_t\right) = \mathbf{G}\tilde{\mathbf{x}}_t,$$

with the equivalent indexed tensor multiplication as

$$[\mathbf{x}_t]_{m,n,i} = \sum_j \mathbf{G}_{m,i,j}[\tilde{\mathbf{x}}_t]_{n,j},$$

where n is the index of the elements of the group, m is the index of atoms in AU, and i, j are Euclidean indices. The associated diffusion energy transformation is

$$\frac{1}{|G|}U_f\left(\mathbf{x}_t\right) = \frac{1}{2|G|}\left\|\sigma_t^{-1}\mathbf{R}^{-1}\left(f(\tilde{\mathbf{x}}_t, U_0; t) - \alpha_t \hat{\mathbf{x}}_t\left(\mathbf{x}_t, t\right)\right)\right\|_2^2 = -\frac{1}{|G|}\log p_t(\mathbf{x}_t).$$

The energy is averaged with $|G|$ to account for the diffusion energy in individual AU with M atoms. We can compute the Jacobian of the transformation $f : \mathbb{R}^{M\times 3} \to \mathbb{R}^{N\times M\times 3}$ as

$$\frac{\mathrm{d}f\left(\mathbf{x}_t\right)}{\mathrm{d}\tilde{\mathbf{x}}_t} = \mathbf{G} \to \frac{\mathrm{d}[f\left(\mathbf{x}_t\right)]_{m,n,i}}{\mathrm{d}[\tilde{\mathbf{x}}_t]_{n',j}} = \mathbf{G}_{m,i,j}\delta_{n,n'}.$$

To derive the transformed dynamics, we inspect one solver step for the reverse Langevin dynamics (identical analysis can be done for reverse diffusion), which is

$$\tilde{\mathbf{x}}_{t+\mathrm{d}t} = \tilde{\mathbf{x}}_t - \frac{1}{2}\mathbf{R}\mathbf{R}^\intercal\left[\frac{\mathrm{d}f\left(\tilde{\mathbf{x}}_t\right)}{\mathrm{d}\tilde{\mathbf{x}}_t}\right]^\intercal \frac{\mathrm{d}U_f\left(\mathbf{x}_t\right)}{\mathrm{d}\mathbf{x}_t}\mathrm{d}t + \mathbf{R}\mathrm{d}\bar{\mathbf{w}},$$

and show the induced gradient transform with its associated indexed representation

$$\frac{\mathrm{d}U_f\left(\mathbf{x}_t\right)}{\mathrm{d}\tilde{\mathbf{x}}_t} = \left[\frac{\mathrm{d}f(\tilde{\mathbf{x}}_t)}{\mathrm{d}\tilde{\mathbf{x}}_t}\right]^\intercal \frac{\mathrm{d}U_f\left(\mathbf{x}_t\right)}{\mathrm{d}\mathbf{x}_t} = \mathbf{G}^\intercal \frac{\mathrm{d}U_f\left(\mathbf{x}_t\right)}{\mathrm{d}\mathbf{x}_t},$$

$$\frac{\mathrm{d}U_f\left(\mathbf{x}_t\right)}{\mathrm{d}[\tilde{\mathbf{x}}_t]_{n,j}} = \sum_m \sum_i \mathbf{G}_{m,i,j}\left[\frac{\mathrm{d}U_f\left(\mathbf{x}_t\right)}{\mathrm{d}\mathbf{x}_t}\right]_{m,n,i}.$$

Observe that in the gradient transformation, the summation occurs over indices i, contrasting with the index j used in the forward transformation to account for the index transposition $[\cdot]^\intercal$ between i and j. For orthogonal transformation, the transposition is also equivalent to the inverse of the individual rotation matrix in $\mathbf{G}$. This method inherently pulls the gradients back to the AU. The computation of the transformed gradient can be adeptly handled using auto-differentiation, specifically as vector-Jacobian products. Furthermore, the gradients accumulated in AU are also averaged by the number of chains in the tessellated domain by dividing the gradient by $|G|$.

We then analyze the transformed solver step with the pull-back gradient transform and get

$$\begin{aligned} f\left(\tilde{\mathbf{x}}_t + \mathrm{d}\tilde{\mathbf{x}}_t\right) &= f\left(\tilde{\mathbf{x}}_t - \frac{1}{2}\mathbf{R}\mathbf{R}^\intercal\left[\frac{\mathrm{d}f(\tilde{\mathbf{x}}_t)}{\mathrm{d}\tilde{\mathbf{x}}_t}\right]^\intercal \frac{1}{|G|}\frac{\mathrm{d}U(\mathbf{x}_t)}{\mathrm{d}\mathbf{x}_t}\mathrm{d}t + \mathbf{R}\mathrm{d}\bar{\mathbf{w}}\right) \\ &= \underbrace{\mathbf{G}}_{\text{symmetrize}}\underbrace{\left(\tilde{\mathbf{x}}_t - \frac{1}{2}\mathbf{R}\mathbf{R}^\intercal\mathbf{G}^{-1}\frac{1}{|G|}\frac{\mathrm{d}U(\mathbf{x}_t)}{\mathrm{d}\mathbf{x}_t}\mathrm{d}t + \mathbf{R}\mathrm{d}\bar{\mathbf{w}}\right)}_{\text{folding to AU}}. \end{aligned}$$

The constrained transformation has a nice interpretation: the solver step first folds the infinitesimal change back, followed by symmetrization. Note that this method is equivariant to permutations of group elements in $\mathbf{G}$ because the gradients are pulled back to AU and tessellated following the order of group elements in $\mathbf{G}$.

Another option to pull the gradients is to perform a "broadcasting" operation from a single AU (indexed with u) of $\mathbf{x}$. This is also a valid gradient transformation that ensures G-invariance. This equation is an example of constrained transformations in eq. (3), and in practice we apply the temperature adjustment described in Supplementary Appendix C.

$$f\left(\tilde{\mathbf{x}}_t + \mathrm{d}\tilde{\mathbf{x}}_t\right) = f\left(\tilde{\mathbf{x}}_t - \frac{1}{2}\mathbf{R}\mathbf{R}^\intercal [\mathbf{G}]_u^{-1} \left[\frac{\mathrm{d}U(\mathbf{x}_t)}{\mathrm{d}\mathbf{x}_t}\right]_u \mathrm{d}t + \mathbf{R}\mathrm{d}\bar{\mathbf{w}}\right).$$

Symmetry operation compositions. The conditioner formalism facilitates the composability of constrained transformations, paving the way for intricate protein geometrical designs. For instance, by strategically combining rotations and translations, we can craft periodic protein assemblies. This technique enables the design of both crystals and quasi-crystals through prescribed tiling operations. Moreover, by combining rotational symmetries with translations, one can engineer protein assemblies exhibiting hierarchical symmetries, producing fractal-like assembly structures, as depicted in the bottom row of Supplementary Figure 28.

Q.4 Practical implementation with scaling and composition

Subsampling. For efficient memory sampling of large symmetric assemblies, we consider reducing the number of chains using chain subsampling techniques. This approach allows us to focus on updating a specific subset, denoted as $\mathcal{S} \subset [1, ..., |G|]$, of subunits in $\mathbf{x}_T$, thereby conserving both memory and computational time.

Given a designated subunit i, the subset $\mathcal{S}$ is derived by selecting the k-nearest neighbor (k-NN) subunits. This selection is determined by the distances between the geometric centers of the subunits, ensuring the incorporation of short-range interactions between them. Through this method, K subunits are chosen, where K represents the count of neighbors the denoiser interacts with during each integration phase.

This randomized selection not only ensures that the gradient update remains globally consistent but also prevents potential structural clashes and suboptimal contact formations. For a visual illustration of the composed constrained transformation process, refer to Supplementary Figure 27, which provides an illustrative example of symmetric sampling in C_4.

Interestingly, this procedure, at its core an index selection mechanism, can also be depicted as a linear transformation using a sparse matrix comprised of 0s and 1s. By harnessing interchain distances, we are equipped to select $K < |G|$ chains following an exhaustive symmetric tessellation. This method of subsampling aligns with established techniques in molecular simulations that employ periodic boundary conditions. To further understand the subsampling process, it is interesting to note that, much like the tessellation method, the subsampling can be described as

$$\mathbf{x}_t = f(\tilde{\mathbf{x}}_t, t) = \tilde{\mathbf{x}}_t^{\mathcal{S}} = \texttt{subsample}(\tilde{\mathbf{x}}_t) = S\,\tilde{\mathbf{x}}_t,$$

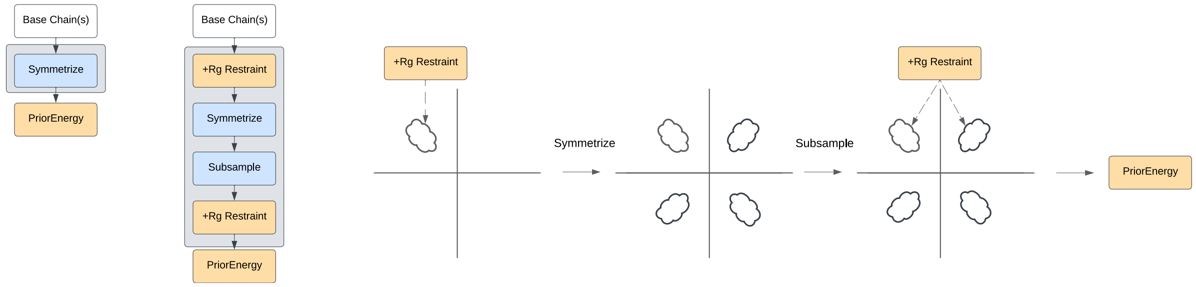

Supplementary Figure 27: Constrained transformations for symmetry operations.

and

$$\frac{\mathrm{d}f(\mathbf{x}_t)}{\mathrm{d}\tilde{\mathbf{x}}_t} = S \in [0,1]^{MN \times KN},$$

where S is the chain selection matrix of size $(KN \times K)$ where $K < M$ is the number of chains selected during sampling.

R_g **energy restraints.** The conditioner formalism provides the flexibility to seamlessly incorporate the restraint energy during energy updates. To ensure optimal contact and packing, we can integrate an R_g penalty through a harmonic or flat-bottom potential. This serves to maintain both the interchain distance and the Asymmetric Unit (AU) Radius of Gyration within a reasonable range via the restraint energy

$$U_f(\mathbf{x}_t, U, t) = U + U_{R_g}(\mathbf{x}_t) = U + ||\mathrm{R}_g(\mathbf{x}_t) - \langle \mathrm{R}_g \rangle||_2^2.$$

The proposed samplers can also be combined with other conditioners (substructure, natural language, shape, etc.) to realize symmetric assembly design with controllable functions.

Composed transformation. Putting this together, the composed transformation is as follows

$$\mathbf{x} = \texttt{subsample}(\texttt{symmetrize}(\tilde{\mathbf{x}}))$$
$$U_f(\mathbf{x}, U, t) = U + U_{R_g}(\tilde{\mathbf{x}}) + U_{R_g}(\texttt{subsample}(\texttt{symmetrize}(\tilde{\mathbf{x}})),$$

We include the schematic of the composed conditioner blocks in Supplementary Figure 27. For implementation, this can be easily implemented in a composable function.

Q.5 Additional symmetric samples

We include more generated samples for selected point groups including C_n (cyclic symmetry), D_n (dihedral symmetry), T (tetrahedral symmetry), O (octahedral symmetry), I (icosahedral symmetry). For all the samples we use the reverse Langevin dynamics $\lambda_0 = 8$ with the Heun SDE solver that integrates from 1 to 0 for 500 steps. We used subunit k-NN sampling with $K = 5$. When $K > |G|$, we set $K = |G| - 1$. We provide additional samples categorized by the imposed symmetry group in Supplementary Figure 28 with a range of sequence lengths per subunit. Our method strictly imposes symmetries.

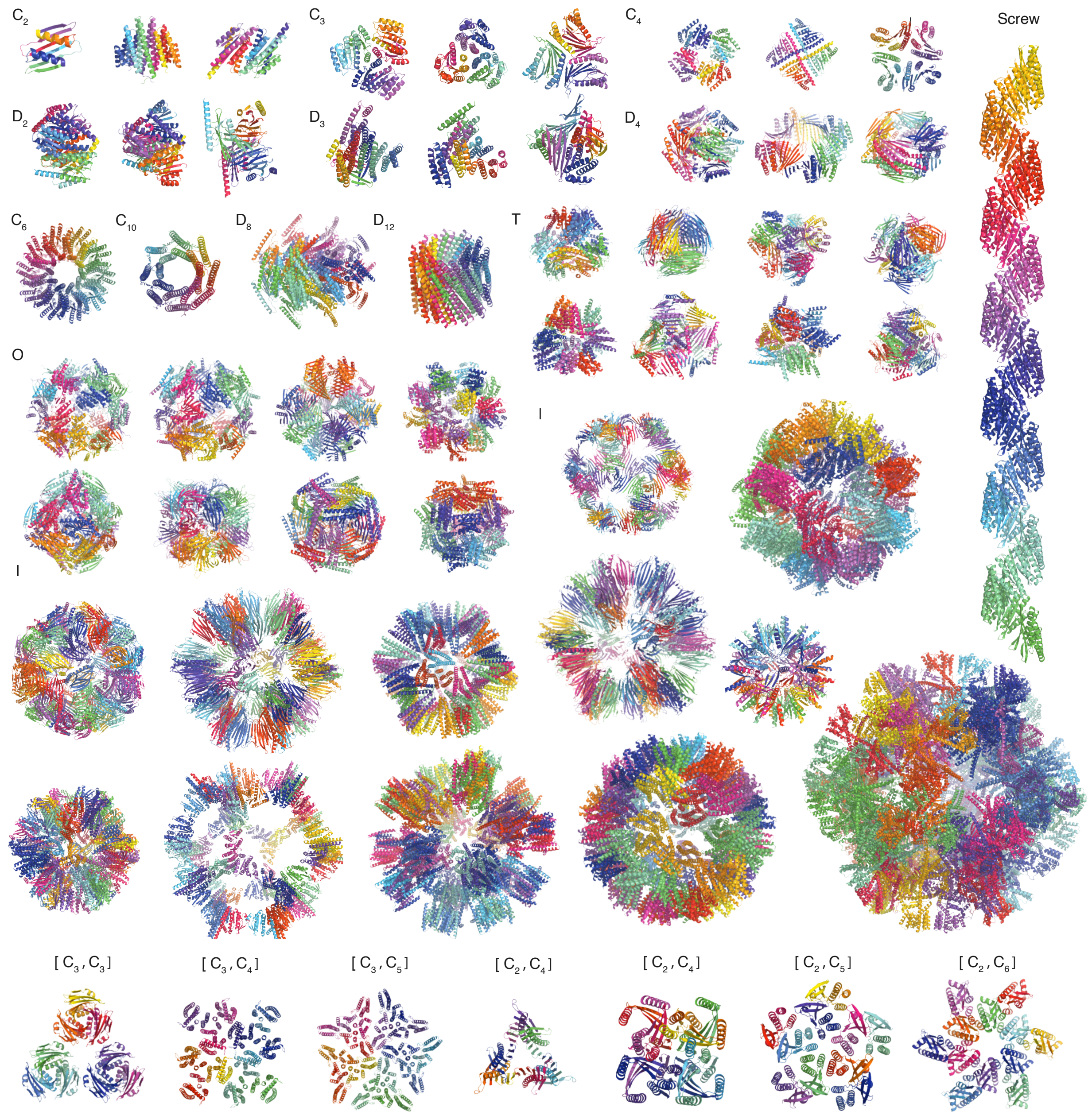

Supplementary Figure 28: **Additional generated complexes based on imposed symmetry groups.**

R Programmability: Shape

R.1 Motivation

Proteins often realize particular functions through particular shapes, and consequently being able to sample proteins subject to generic shape constraints would seem to be an important capability. Pores allow molecules to pass through biological membranes via a doughnut shape, scaffolding

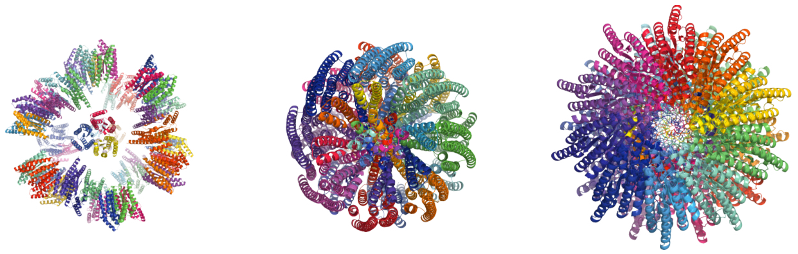

Supplementary Figure 29: **Examples of poor packing in sampled symmetric complexes.** Underpacking or overpacking can occur occasionally, and may be partially addressed by density restraints.

proteins spatially organize molecular events across the cell with precise spacing and interlocking assemblies, and receptors on the surfaces of cells interact with the surrounding world through precise geometries. Here, we aim to explore and test generalized tools for conditioning on volumetric shape specifications within the diffusion framework.

R.2 Approach

Our shape conditioning approach is based on optimal transport [174], which provides tools for identifying correspondences and defining similarities between objects, such as the atoms in a protein backbone and a point cloud sampled from a target shape. We leverage two tools in particular: (i) the Wasserstein distance [174], which measures point cloud correspondences in Euclidean space and (ii) the Gromov-Wasserstein distance, which can measure the correspondences between objects in different domains by comparing their intra-domain distances or dissimilarities. Because Gromov-Wasserstein distance leverages *relational* comparisons, it can measure correspondences between unaligned objects of different structure and dimensionality such as a skeleton graph and a 3D surface [175] or unsupervised word embeddings in two different languages [176].

Bounding degeneracy We initially experimented with restraints based purely on the Wasserstein distance and a target point cloud, which can estimated with the Sinkhorn algorithm [174], but found that the huge degeneracy in potential volume-filling conformations would often lead to jammed or high-contact-order solutions when using a modest amount of MCMC sampling. While long-run Langevin sampling or similar approaches could allow gentle annealing into a satisfactory configuration in principle, we sought to accelerate convergence by breaking this degeneracy with a very coarse ”space-filling plan” for how the fold should map into the target point cloud, which the prior can then realize with a specific protein backbone.

Mapping 1D to 3D We can leverage Gromov-Wasserstein (GW) optimal transport to answer the question “How would an *idealized* protein fill a given space in a target 3D volume?”. To do so, we (i) built an idealized distance matrix for a protein based on the 1D to 3D distance scaling law[10]

[10]This scaling law was fit on a large single-domain protein 6HYP.

of $D^{\text{ideal}}(|i-j|) = 7.21 \times |i-j|^{0.32}$, (ii) computed the distance matrix for our target shape, and (iii) solved for the Gromov-Wasserstein optimal transport given these two distance matrices [174] yielding a coupling matrix $K_{\text{GromovWasserstein}}$ with dimensionality $N_{\text{atoms}} \times N_{\text{points}}$. This coupling map sums to unity and captures the correspondence between each point in the protein and in the shape. We use a small amount of entropy regularization to solve the optimal transport problem [174].

Optimal Transport loss In the inner loop of sampling, we can combine the Gromov-Wasserstein coupling with simple Wasserstein couplings as a form of regularization towards our fold "plan". Our final loss is then

$$\text{ShapeLoss}(\mathbf{x}, \mathbf{r}) = \sum_{i,j} \left(K_{ij}^{\text{GW}} + K_{ij}^{\text{W}}(\mathbf{x}, \mathbf{r}) \right) \|\mathbf{x}_i - \mathbf{r}_j\|,$$

where we compute the Wasserstein optimal couplings K_{ij}^{W} with the Sinkhorn algorithm [174]. This yields a fast, differentiable loss that can be used directly for sampling.

Time-dependent scaling We weight the $\text{ShapeLoss}(\mathbf{x}, \mathbf{r})$ term with the scaling factor

$$w_t^{(\text{shape})} = \text{Clamp}(\sqrt{\text{SNR}_t}, [0.001, 3.0]),$$

and then add its gradient directly to the loss during sampling. So the weighted objective is

$$\text{ShapeLoss}_t(\mathbf{x}, \mathbf{r}) = \text{Clamp}(\sqrt{\text{SNR}_t}, [0.001, 3.0]) \sum_{i,j} \left(K_{ij}^{\text{GW}} + K_{ij}^{\text{W}}(\mathbf{x}, \mathbf{r}) \right) \|\mathbf{x}_i - \mathbf{r}_j\|.$$

Scaling point clouds to protein sizes The Wasserstein and Gromov-Wasserstein losses are sensitive to the point cloud length scales, but our shapes will not in general be correctly sized to the protein that we wish to design them with. Of the methods that we explored to deal with this, two that demonstrated some success were

- **Fixed volume scaling**. We estimate an approximate volume of our point cloud via on a hard-sphere probe with radius set on typical nearest neighbor distance. We correct for sphere overlaps via second-order inclusion-exclusion formulas. We then resize the point cloud geometry to match ideal protein geometry scaling of approximately $\approx 128\mathring{A}^3$ per residue and then adjust by a manually tuned factor (in practice anywhere from 0.3-1.0).
- **Autoscaling** We use the fixed scaling approach for GW distance calculation but also make our loss scale invariant during optimization by computing the loss with a version of the current structure that has been rescaled to have the same radius of gyration as the target point cloud.

Generating point clouds for characters We rendered Latin letters and Arabic numerals in the Liberation Sans font, extruded these 2D images into 3D volumes, and then sampled isotropic point clouds from these volumes. The shape logo in Fig. 1C (silhouette of the "Stanford bunny") was created using data from The Stanford 3D Scanning Repository (`http://graphics.stanford.edu/data/3Dscanrep/`).

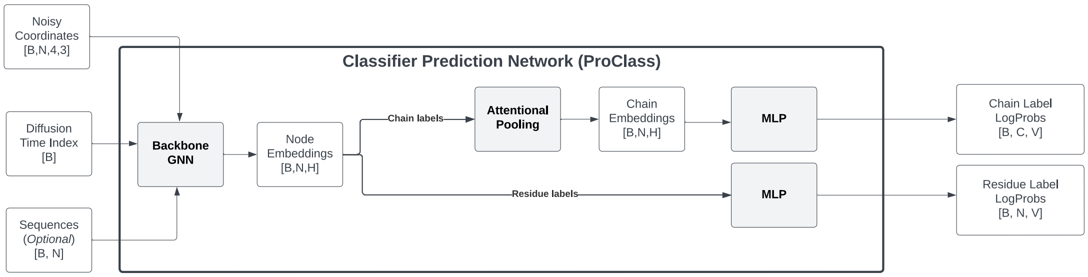

Supplementary Figure 30: **ProClass model architecture.**

S Programmability: Classification

S.1 Motivation

Protein databases provide a rich structured set of descriptions of various aspects of proteins. Proteins are classified in these databases in terms of various aspects of their sequence, structure, and functions. We can use any of these structured descriptors to generate proteins with structurally and semantically useful features. Some of these descriptors, particularly ones that correspond with protein function, may induce diffuse and complex structure changes that resist simple description. To this end, we explore using a multi-property protein classifier as a conditioner for generation, attempting to provide the ability to directly design proteins with desired categorical descriptions. We see this as an initial step towards programmability of protein function.

S.2 Model inputs

We passed noised backbone coordinates obtained from the PDB as input to the model, along with a scalar $0 < t < 1$ denoting the time during diffusion that the noise was sampled at. The model can optionally consume sequence information if available.

S.3 Featurization

We encoded the diffusion time with a random Fourier featurization (e.g., see [177]). When providing a sequence, we encoded it with a learnable embedding layer of amino acid identity. Finally, we passed backbone coordinates to extract 2-mer and chain-based distances and orientations as described in Supplementary Appendix G. We passed the sum of these components to the neural network.

S.4 Architecture

The encoder is a message-passing neural network. We formed the graph by taking K=20 nearest neighbors and sampling additional neighbors from a distribution according to a random exponential method.

We passed node and edge embeddings to each layer, with each node being updated by a scaled sum of messages passed from its neighbors. We obtained the message to pass from node i to node

j by stacking the embeddings at node i, those at node j, and $\mathcal{E}$, and passing these to a multi-layer perceptron (1 hidden layer). We updated edges similarly. In each layer, we also applied layer normalization (along the channel dimension) and dropout (dropout probability=0.1).

After processing by the MPNN, we passed node embeddings to a different classification head for each label. For each head corresponding to a chain-level label, we pooled residues from each chain using an attentional pooling layer. We then passed the resulting embeddings to an MLP with 1 hidden layer to output logits for each label.

S.5 Labels and loss functions

We trained the model to predict the following labels: CATH, PFAM, Funfam, Organism, Secondary Structure, Interfacial Residue. We quantified the loss for each label prediction using cross entropy, and summed all components with equal weights.

S.6 Training

We trained the model for 300 epochs with an Adam optimizer [132] with default momentum settings (betas=(0.9,0.999)). We linearly annealed the learning rate from 0 up to 0.0001 over the first 10,000 steps and then kept it constant. During training, we first sampled a time stamp $0 < t < 1$ uniformly, then sampled noise from the globular covariance distribution, injected this noise into the backbone coordinates, and fed the result to the model. Next, we predicted labels, computed losses, and updated parameters with the Adam optimizer.

S.7 Hyperparameters

Our classification model has 4 layers, with node feature dimension 512 and edge feature dimension 192. Our node update MLP has hidden dimension 256 with 2 hidden layers, and our edge update MLP has hidden dimension 128 with 2 hidden layers.

T Programmability: Natural Language Annotations

T.1 Motivation

Recent advances in text-to-image diffusion models such as DALL-E 2 [83] and Imagen [82] have produced qualitatively impressive results using a natural language interface. Given the open availability of pre-trained language models and a corpus of protein captions form large scientific databases such as the PDB [126] and UniProt [178], we explore the possibility of creating a natural language interface to protein backbone generation. To do this, we build a protein captioning model (ProCap), which predicts $p(y|\mathbf{x}_t)$, where y is a text description of a protein and $\mathbf{x}_t$ is a noised protein backbone. This conditional model, when used in conjunction with the structural diffusion model presented in the main text, can be used as a text-to-protein backbone generative model.

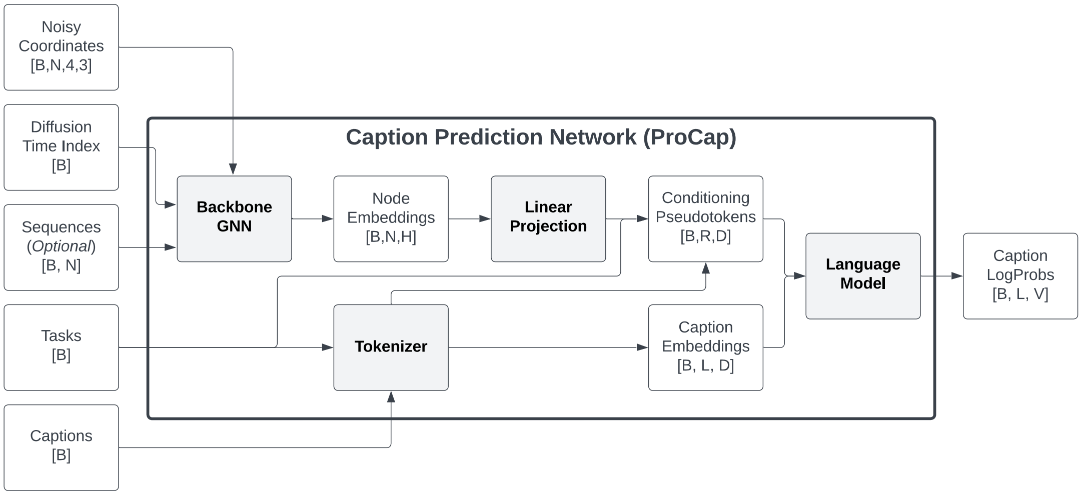

Supplementary Figure 31: **ProCap model architecture.** ProCap connects a pretrained graph neural network encoder to an autoregressive language model trained on a large data corpus including scientific documents. We use the 125M parameter GPT-Neo as the language model, with internal dimension $D = 768$. Conditioning is achieved with pseudotokens generated from encodings of protein complex 3D backbone coordinates (batch size B, number of residues N, embedding dimension H) and a task token indicating whether a caption describes the whole complex or a single chain. The R relevant pseudotokens for each caption, consisting of the chain/structure residue tokens and the task token, are passed to the language model along with the caption. When used in the forward mode, ProCap can describe the protein backbone by outputting the probabilities of each word in the language model's vocabulary of size V for each of the L tokens of a caption. When used in conjunction with the prior model, it can be used for text-to-protein backbone synthesis. In training, ProCap uses a masked cross entropy loss applied only to the caption logits.

T.2 Dataset curation

To build a caption model, we begin by curating a paired dataset of protein structures and captions from both the PDB and UniProt databases. Caption information is collected for the structures used for the backbone diffusion model training, as well as the individual chains within these structures. For each structure, we use the PDB descriptive text as an overall caption. For each chain in a structure, we obtain a caption by concatenating all available functional comments from UniProt. Structures containing more than 1000 residues are not used, corresponding to a minority (10%) of all structures. The final set used to train and validate the caption model contains approximately 45 thousand captions, including those from both PDB and UniProt. Unlike the backbone model, the splits used for training are completely random. The small size of the dataset constrained architecture choices to those with relatively few free parameters.

T.3 Model architecture

T.3.1 Architecture overview

To predict captions given noised structures, we construct ProCap using a pretrained language model and a pretrained protein encoder. The pretrained language model is the GPT-Neo 125 million parameter model [179]. GPT-Neo was trained on the Pile [180] which contains articles from arXiv and PubMed. Its choice is motivated to maximize the chance that the model would begin training with some understanding of protein-related text. We also use the pretrained graph neural network encoder from ProClass, the protein structure classification model introduced above, to encode protein backbones. Analogously to the choice of the language model, the purpose of the structure encoder is to start ProCap with semantic knowledge of protein structure. To condition the autoregressive language model, GPT-Neo, pseudotokens are formed from structures using the ProClass encoder and prepended to the caption as context, similar to [181].

T.3.2 Data embedding

Here, we describe the embedding of task, caption, and structure data into a shared tensor representation for input to the language model. We encode captions and task tokens using a modified version of the GPT-Neo tokenizer, whose vocabulary we augment with a special token to distinguish between prediction tasks involving single chains and those relating to entire structures. We convert structure inputs into pseudotokens with the same shape as text embeddings through the graph neural network encoder of the pre-trained ProClass model. We then concatenate the task, structure, and caption embeddings into a representation to pass to the language model to obtain logits representing the probabilities of caption tokens. We train our model on a standard masked cross entropy loss of the caption. Fig. 31 details the overall architectural flow. We proceed to discuss the details of the embedding procedure.

Structure encoding in ProCap relies on a pretrained ProClass model. This classifier model consists of a GNN with multiple heads to extract different class information, as described previously. We use the GNN portion of the classifier network to obtain embeddings of each residue in the latent space of the classifier, with the intent that the pre-trained classifier weights should help ProCap learn the relationship between structures and captions. Besides the 3D information of the atoms in each structure, we input the diffusion timestep (noise level) to the GNN via a Fourier featurization layer which converts the diffusion time to a vector with the same dimension as the GNN node embedding space using randomly chosen frequencies between 0 and 16. To allow for ProCap to learn the optional use of sequence information, in 25% of the training data we pass sequences along with structures. In these cases, we convert the amino acid information for each residue through a single embedding layer with output size equal to that of the GNN node embedding space dimension, adding the result to the time step vector.

We add task tokens to the model to allow for captions of both single chain and full complex captions. For the prediction of UniProt captions describing single chains within structures, we pass only the embeddings of the residues in the relevant chain to the language model. For the prediction of the PDB captions related to entire structures, we pass all residue embeddings. In addition, we use a linear layer after the ProClass embeddings to go between the ProClass latent space and the embedding space of the language model, which are of different dimensionality. Finally, in order to

help the model distinguish between PDB and UniProt prediction tasks, we prepend the encodings of entire structures with an embedding vector of a newly defined PDB marker token. We normalize the components of all structure vectors such that each one has zero mean and unit variance.

In summary, the ProCap architecture consists of a pre-trained GNN model for structure embedding and a pre-trained language model for caption embedding, with a learnable linear layer to interface between the two and a learnable language model head to convert the raw language model outputs to token probabilities.

T.4 Model training

We train ProCap to be compatible with conditional generation using the structural diffusion prior model. Like the other conditional models in this paper, we noise each structure according to the schedule of the structural diffusion model. During ProCap training, we freeze the graph neural network encoder weights from the pre-trained ProClass model. As we add a <|PDB|> task token to the GPT-Neo vocabulary to cue the model to predict whole complex captions from the PDB, we allow the language model to learn in order to optimize the encoding of this new token and refine the embeddings of existing ones. Given the relatively small training data size, we also experimented with training ProCap with the language model frozen except for its head. As we found that the average perplexity achieved on the validation set was generally inferior when freezing language model weights, in our final training run we optimize all weights of the language model.

We performed training on a single V100 with a constant learning rate of 5×10^{-5} and the Adam optimizer with hyperparameters $\beta_1 = 0.9$, $\beta_2 = 0.999$. We evaluate loss on our validation set after every 2000 training examples. Over 24 epochs, the validation cross entropy loss reaches a minimum of approximately 2.44, and the weights from this checkpoint are used to assess model performance.

T.5 Performance

In order to test ProCap as a generative model, we draw high-quality conditional and corresponding unconditional low-temperature samples from the model. To that end, we employ a structural denoising approach in a similar fashion to the method described in [55]. Specifically, we use the hybrid reverse diffusion SDE of Appendix C to evolve noisy random sample structures drawn from the diffusion model prior, with gradients of the ProCap loss with respect to structure added to the gradients of the structure diffusion model. When the size of the ProCap gradients is too small relative to those from the prior model, there is little appreciable difference between a caption-conditioned sample and an unconditional sample drawn from the same seed. We thus scale the ProCap gradients by a guidance scale of up to 100 and find that the resulting samples are better conditioned, analogously to previous work on classifier guidance [80]. At even larger guidance scales, the coherence of the samples breaks down as the base model's gradients are overwhelmed.

We present examples of our generated samples in the main text. To evaluate ProCap model performance, we measure the improvement in caption loss during the SDE evolution between the unconditioned and conditioned samples. As an independent check, we also examine the gain in the TM-score between our sample (conditioned over unconditioned) and a target PDB structure which

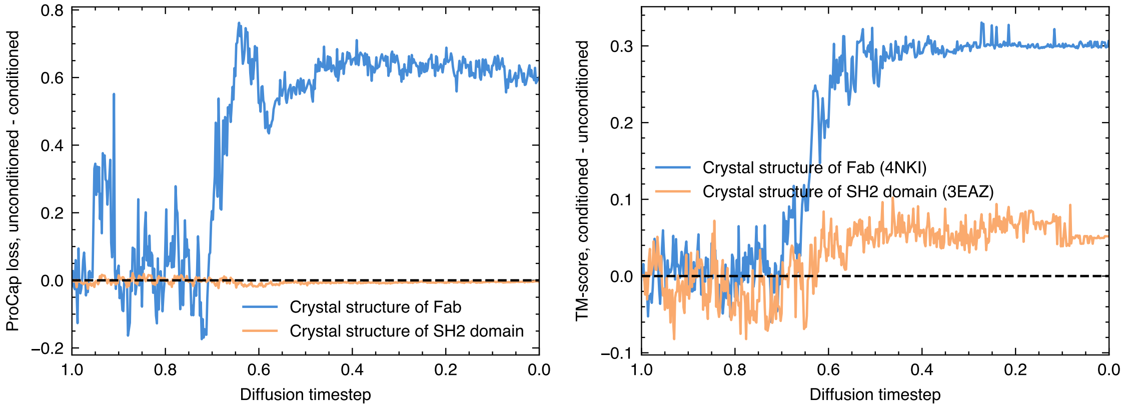

Supplementary Figure 32: **ProCap evaluation metrics show effect of natural language conditioning compared to unconditioned samples from the same noised seed structure.** (Left) The caption model cross-entropy loss as a function of diffusion timestep, for two sample trajectories with and without the use of caption gradients. (Right) The TM-score between sampled structures and example structures from the PDB corresponding to the captions used for conditioning.

exemplifies the caption being used for conditioning. Finally, we analyze the generated structures visually for structural coherence. Qualitatively, starting from the same noisy random structure, the diffusion model yields denoised structures which demonstrate desirable characteristics including secondary structure elements, both with and without guidance from the caption model.

The caption loss and TM-score metrics for example sampling trajectories are shown in Fig. 32. Both are initially quite noisy, and the conditioned and unconditioned samples are equally likely at high t to have lower ProCap loss and/or better alignment with the target structure. However, over the course of the reverse diffusion, the effect of the conditioning is demonstrated. It is particularly notable that the TM-score is relatively stable at low t, indicating a regime where the SDE evolution is fine-tuning structural details rather than making large-scale changes. In addition, we see that the impact of classifier guidance can vary widely, possibly owing to the intricate balance between the gradients over the diffusion trajectory. It remains challenging to robustly generate samples with natural language conditioning in a systematic fashion; nevertheless, our results serve as a proof of concept of guided diffusion using text input.

As a final check of the ProCap model, we ask whether samples generated guided by natural language suggestive of a particular CATH topology are seen as being representative of that topology, as measured by the model of appendix S. In Supplementary Figure 33, we compare the ProCap perplexity and ProClass probability of an immunoglobulin fold (CATH 2.60.40) for backbones generated using the caption “Crystal structure of Fab”. We see a strong correlation between the negative log probability of the relevant topology and the ProCap loss, suggesting that ProCap shows signs of understanding the meaning of natural language captions at the level of CATH topologies.

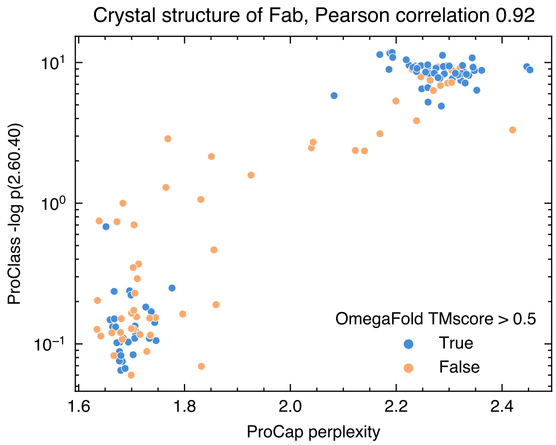

Supplementary Figure 33: **ProCap perplexity shows correlation with ProClass loss.** From a group of samples generated with classifier guidance from ProCap using an antibody-related caption, we plot the resulting perplexity of each backbone against its probability of an immunoglobulin fold (CATH 2.60.40). We estimate the fold probability of a backbone using the classification model described in appendix S, after the backbone is generated. Successful refolding can take place regardless of perplexity, as described further in appendix K.5.

U Experimental Validation

U.1 Protein design

Four sets of designs were generated for experimental validation: two unconditional sets (Unconditional I and II) and two sets conditioned on CATH class or topology (Conditioned I and II, respectively). The full protocol for each of these involved generating a set of Chroma backbones (either unconditionally or conditioned), designing sequences for each backbone (10 per backbone for Unconditional I and 1 per backbone for the rest), and sub-selecting a smaller set of designs to be experimentally characterized (see Supplementary Table 7 for details). Importantly, no sub-selection based on refolding or structural energy calculations was performed. Further, the protocols were run in an automated fashion with no manual intervention or selection of designs. All experimentally addressed protein sequences are included in Zenodo dataset `https://doi.org/10.5281/zenodo.8285063`.

U.2 Experimental methods

U.2.1 DNA design

Chroma protein sequences were backtranslated and codon optimized for mammalian expression, but omitting *E.* coli rare codons AGA and AGG to enable flexibility in the choice of expression host. DNA sequences were ordered as eBlocks from Integrated DNA Technologies, Inc. and cloned into either mammalian or bacterial expression plasmids using Golden Gate Assembly (NEB E1601L), with recipient vector information detailed in corresponding subsequent sections. All DNA sequences are included in Zenodo dataset `https://doi.org/10.5281/zenodo.8285063`.

Supplementary Table 7: **Design protocol details**

	**Unconditional I**	**Unconditional II**	**Conditioned I**	**Conditioned II**
**Length**	[100; 450], uniform	[100; 950], uniform	100 or 200, uniform	150
**Model**	`ChromaBackbone v0.4999`	`ChromaBackbone v0.4998`	`ChromaBackbone v0.4999`	`ChromaBackbone v0.4999`
**Integration Parameters**	1000 steps of SDE, $\lambda_0 = 10$, $\psi = 2$	1000 steps of SDE, $\lambda_0 = 10$, $\psi = 0.1$	200, 600, or 2000 steps of SDE, $\lambda_0 = 10$, $\psi = 2$	2000 steps of HMC, $\lambda_0 = 10$, $\psi = 0.9$
**Conditioning**	N/A	N/A	CATH class: α, β, or mixed α/β	CATH topo 2.40.155
**Backbones generated**	500	2000	335 per class	54
**Filter**[1]	$\log p(\mathbf{s})$	R'_g	$\mathcal{L}(\mathbf{x})$, $\log p(\mathbf{s})$, $\log p(\chi)$	$\mathcal{L}(\mathbf{x})$, $\log p(\mathbf{s})$, $\log p(\chi)$
**Final designs**	172	96	36	6

[1] $\mathcal{L}(\mathbf{x})$–Chroma ELBO; $\log p(\mathbf{s})$–Chroma Design A sequence log-likelihood; $\log p(\chi)$–Chroma Design B chi-angle log-likelihood; R'_g–ratio of observed over expected radius of gyration, for given sequence length.

U.2.2 Pooled split-GFP solubility assay

Split-GFP components GFP1-10 and GFP11 were codon optimized for expression in *E*. coli and cloned into the pNAS1b vector [182] under araBAD and pLtetO promoters, respectively. Chroma protein-encoding eBlocks were introduced into the split-GFP vector using pooled Golden Gate Assembly, resulting in gene cassettes under the pLtetO promoter with a C-terminal GFP11 fusion tag. The final encoded library protein sequences were as follows: MGSSHHHHHHSSGLVP RGS-[Chroma protein]-GSDGGSGGGS-[GFP11]. Pooled plasmid libraries were cloned using ElectroMAX DH10B cells (Invitrogen 11635018), and subsequently transformed into BL21 strain T7 Express Competent *E*. coli (NEB C2566I).

BL21 cells electroporated with the split-GFP plasmid library were recovered for 1 h in SOC medium (NEB B9020S) and inoculated directly into 50 mL terrific broth (TB, Gibco A1374301) + 100 ng/µL carbenicillin and grown at 37°C, 230 rpm for 16 h. Cells were then diluted to OD_{600} = 0.2 and grown at 37°C, 230 rpm until OD_{600} = 0.8. Split-GFP system components were then induced using 0.1 % w/v L(+)-arabinose (Thermo Scientific TS36518-0250) and 100 ng/µL anhydrotetracycline (Sigma Aldrich 37919-100MG-R) and cells were grown for an additional 3 h. In parallel, cultures of BL21 cells expressing either dihydrofolate reductase (DHFR, a positive control) or human beta-3 adrenergic receptor (ADRB3, a negative control GPCR `https://www.uniprot.org/uniprotkb/P13945`) in the split-GFP vector were grown and induced under the same conditions as the library (Table 8). These control cells were then spiked into the library population at a 1:1000 ratio. 5 mL of the library with control spike-in cell mixture were set aside for miniprep and sequencing analysis of pre-FACS populations. 5 µL of the cell mixture was then washed twice with 1 mL cold PBS and then sorted into 4 different gated populations representing a range of GFP fluorescence values on a BD FACSAria. Gating parameters were determined empirically based on clonal positive and negative control cells, as shown in Supplementary Figure 38c. At least 50,000 cells were collected per gate and recovered for 1 h in 1 mL

SOC at 37°C, 230 rpm. Recovered cells were inoculated in 5 mL TB + 100 ng/µL carbenicillin and grown for an additional 16 h. Plasmid populations were then isolated by miniprep (Machery-Nagel 740588.50). For the fluorescence stability experiment shown in Supplementary Figure 38d, cells recovered after FACS and regrown overnight were then subjected to a second round of identical experimentation in which split-GFP components were induced and cells were re-examined on the BD FACSAria.

U.2.3 Nanopore sequencing and analysis

Plasmid libraries from each population (i.e. the cells harboring the split-GFP Chroma library + spike-in controls prior to sorting and each of the 4 bin-sorted populations) were digested using HindIII and desalted using 0.7x v/v AMPure XP beads (Beckman Coulter A63880). 200 fmol of each library (assuming an average length of 6kb) underwent DNA repair and end prep using manufacturer guidelines for R9 MinION flow cells (Oxford Nanopore FLO-MIN106D). The DNA was then purified with AMPure XP beads at a 1:1 ratio then quantified by Qubit 4 Fluorometer (ThermoFisher). 500 ng of DNA from each library underwent barcode ligation (Oxford Nanopore barcoding kit EXP-NBD104), followed by another 1:1 AMPure XP bead purification. Each library was then pooled in equimolar ratios and loaded onto the MinION flow cell. One experiment was performed using only proteins UNC_001 through UNC_172 (unconditional designs, 13.68 million reads) and two additional experiments were performed using all Chroma proteins pooled together (unconditional and semantically conditioned designs, 18.28 million reads total).

Sequencing reads were basecalled using Bonito Basecaller v0.6.1 (`https://github.com/nanoporetech/bonito`) with ONT Chemistry r9.4.1 and accuracy mode 'high'. Raw fastq files were generated and demultiplexed using a custom script. Demultiplexed reads were filtered for reads longer than 400 bp using SeqKit v2.3.1 (`https://bioinf.shenwei.me/seqkit/`) [183] and aligned to reference Chroma sequences using Minimap2 v2.23 (`https://github.com/lh3/minimap2`) [184] to generate BAM alignment files. BAMs were sorted and indexed using samtools v1.16.1 (`https://github.com/samtools/samtools`). For each BAM file, pysam v0.20.0 (`https://github.com/pysam-developers/pysam`) was used to count reads aligned with each reference sequence. For sequence enrichment analysis, enrichment scores were assigned to each protein in each sorting bin by dividing normalized read counts for a given protein in a given bin by normalized read counts of that protein in the pre-FACS library. Split-GFP bin scores were assigned to each protein, j, as

$$\text{score}_j = \sum_{i=0}^{n=3} \frac{\text{enrichment score}_i}{\text{sum of enrichment scores}_j} \times bin_i$$

.

For pooled assay score calculations, proteins in the set containing UNC_001 through UNC_172 (smaller unconditional designs) were analyzed alone (i.e. not considering read counts for other protein sequences) to enable triplicate data analysis between all three experiments. The set containing UNC_173 through UNC_268 (larger unconditional designs) were also analyzed alone for the duplicate experiments performed. Given the small number of semantically conditioned designs, these proteins were analyzed with all other proteins. Raw and processed read counts are included in Zenodo dataset `https://doi.org/10.5281/zenodo.8285063`.

U.2.4 Soluble protein expression confirmation via western blot

The top and bottom 20 scoring unconditional proteins from set UNC_001 through UNC_172 (smaller unconditional designs) and the top 10 proteins from set UNC_173 through UNC_268 (larger unconditional designs) were cloned into an *E. coli*-based overexpression vector based on pET (kanR, pBR22 origin, T7 promoter for Chroma protein expression) by Golden Gate Assembly, resulting in the following protein expression format: MGS-[Chroma protein]-GSENLYFQG SAWSHPQFEK, which includes a C-terminal TEV cleavage site and Strep-tag. Plasmids were transformed into the BL21 derivative T7 Express Competent *E. coli* (NEB C2566I). Recovered cells were inoculated into 1 mL TB + 50 ng/μL kanamycin and cells were grown in a 96-well deep well plate at 37°C, 230 rpm for 16 h. Cells were then diluted to OD_{600} = 0.15 in 1 mL TB and grown to OD_{600} = 0.8. Protein expression was induced with 400 μM isopropyl ß-D-1-thiogalactopyranoside (Teknova NC1601425) and cells were grown for an additional 3 h. Cells were spun down at 500 x g, media was discarded, and cell pellets were stored at -80°C for 1 day. Cell pellets were then thawed on ice for 5 min and pellets were resuspended in 40 μL lysis buffer consisting of 50 mM NaCl (Invitrogen AM9760G), 50 mM Tris pH 7.4 (Invitrogen 15567027), 1x BugBuster (Millipore 70584-3), 5% glycerol (Fisher G331), 1x cOmplete protease inhibitor cocktail (Roche 11873580001), and 1 mM dithiothreitol (Sigma 10197777001). Cells were allowed to lyse on ice for 10 min, then spun at 500 x g for 15 minutes to clear lysates.

1 μL of each lysate was run on a NuPAGE 12% or 4-12% Bis-Tris mini protein gel (ThermoFisher NP0341 or NP0322) in MES buffer (Novex NP0002) and transferred to a PDVF membrane (iBlot, ThermoFisher IB401002). Membranes were blocked in 5% milk powder in TBST for 1 h at 23°C, shaking. Membranes were then treated with either 1:5000 Streptactin-HRP (IBA-Lifesciences 2-1502-001) or anti-Strep-tag-HRP (StrepMAB-Classic HRP conjugate, IBA-Lifesciences 2-1509-001) in 5% milk in TBST for 1 h at 23°C, shaking. Membranes were then treated with ECL western blotting substrate (SuperSignal West Dura, ThermoFisher 34075) and visualized on an iBright FL1500 imaging system (ThermoFisher). As some proteins expressed at much higher levels than others, some blots were rerun with 10- to 100-fold lysate dilutions to enable qualitative visualization of proteins at various expression levels on the same blot. Proteins were considered to be detected if a band was visible at approximately the anticipated molecular weight.

U.2.5 Protein purification

For *E. coli*-based protein expression (for all tested proteins except SEM_011), 1 L of Gibco Terrific Broth (ThermoFisher A1374301) + 50 ng/μL kanamycin was inoculated with *E. coli* BL21 (NEB C3010I) containing the bacterial expression vector of the desired protein with C-terminal Strep-tag (plasmid information in previous section). Cells were allowed to grow to log phase at 37°C with shaking before induction with 400 μM IPTG (Teknova I3502) and further incubation for 3 hours at 37°C.

Cells were harvested by centrifugation at 4,000 x g for 30 minutes, resuspended in lysis buffer (20 mM Tris pH 8, 150 mM NaCl, 1x Halt protease inhibitor cocktail ThermoFisher 1861279, 1x Benzonase Nuclease Sigma-Aldrich E1014) and lysed by sonication. Lysates were cleared with centrifugation at 15,000 x g for 30 min, passed through a 0.2 μm filter, and incubated overnight at 4°C, with shaking, with 5 mL Strep-Tactin XT 4Flow High-capacity resin (IBA Lifesciences

2-5030).

After incubation, resin was loaded on gravity column and allowed to flow through, then washed with 2 x 10 CV Strep-Tactin XT wash buffer W (IBA Lifesciences 2-1003) and eluted with 2x 1 CV Strep-Tactin XT elution buffer BXT (IBA Lifesciences 2-1042). Elution fractions were pooled and incubated with TEV (Sigma Aldrich T4455) at a 1:100 v/v concentration ratio to protein, overnight at room temperature.

The sample was then buffer exchanged back into Strep-Tactin XT wash buffer W using a Zeba desalting column (Thermo Scientific 89893) and incubated with 5 mL Strep-Tactin XT resin and 1 mL cOmplete His-Tag Purification Resin (Millipore Sigma 5893801001) for 1 hr at 4°C before flowing through a gravity column to remove TEV and uncleaved protein.

Samples were then concentrated to a volume of approximately 5 mL and purified via size exclusion chromatography (SEC) on a HiLoad 16/600 Superdex 75 Column (Cytiva GE28-9893-33) into a final buffer of 20 mM Tris pH 7.5 100 mM NaCl. Fractions were collected, purity was assessed by SDS-PAGE, and appropriate fractions were pooled.

For mammalian-based protein expression of SEM_011, a gBlock encoding the protein was introduced into a plasmid for transient transfection via Golden Gate Assembly under the CMV promoter with an N-terminal signal peptide, based on vector pcDNA3.4 (ThermoFisher, A14697). The protein also had a C-terminal TEV cleavage site and Strep-tag identical to the configuration used for bacterial expression described above. 100 mL of Expi293F cells (ThermoFisher A14635) in Expi293 expression medium was transfected with the construct containing C-terminal Strep tag following manufacturer's guidelines. Cells were transfected on Day 0 at a density of $3x10^6$ viable cells/mL with 100 µg of plasmid DNA and placed in shaker at 37°C, 8% CO_2. At 24 h post-transfection, cells were fed with transfection enhancers and returned to shaker for expression until day 5.

Expression supernatant was harvested at 70% cell viability by centrifugation at 4,000 x g for 30 min. Supernatant was clarified further through a 0.22 µM filter for immediate purification. 2 mL of Strep-Tactin XT 4Flow High-capacity resin (IBA Lifesciences 2-5030) was added to the supernatant and placed on a roller for 24 h at 4°C for batch binding.

After incubation, resin was loaded on gravity column and allowed to flow through, then washed with 7.5 CV Strep-Tactin XT wash buffer W (IBA Lifesciences 2-1003) and eluted with 2 x 2.5 CV Strep-Tactin XT elution buffer BXT (IBA Lifesciences 2-1042). Eluted protein was concentrated to 2.5 mL using Amicon Ultra-15 3 kDa spin concentrators (Millipore UFC900324) followed by buffer exchange into PBS pH 7.4 using PD-10 desalting columns packed with Sephadex G-25 resin (Cytiva 17085101). Desalted protein was incubated overnight with TEV protease (Sigma Aldrich T4455) at a 1:100 v/v concentration ratio to protein, overnight at 4°C.

The sample was then incubated with 1 mL Strep-Tactin XT resin and 1 mL cpmplete His-Tag Purification Resin (Millipore Sigma 5893801001) for 1 h at 4°C before flowing through a gravity column to remove TEV and uncleaved protein.

Cleaved protein was then concentrated to a volume of approximately 1 mL and purified via size exclusion chromatography (SEC) on a 10/300 Superdex 75 Increase Column (Cytiva 29148721)

into a final buffer of 20 mM Tris pH 7.5 100 mM NaCl. Fractions were collected, purity was assessed by SDS-PAGE, and appropriate fractions were pooled.

U.2.6 Circular dichroism

CD spectra to capture protein secondary structure were acquired using a 1 mm pathlength cuvette (JASCO Part #0556) on a JASCO CD-1500 spectropolarimeter at 20°C. To capture far-UV CD, proteins were buffer exchanged into 10 mM $NaPO_4$ pH 8.5 (Thermo Scientific), concentrated to 0.3-0.5 mg/mL using 10 kDa molecular weight cutoff Amicon Ultra-4 Centrifugal filters (Millipore UFC801024), and read in the UV spectral range of 190 – 250 nm. The CD scale used was 200 mdeg/1.0 dOD with a DIT of 4 s, a bandwidth of 1 nm, a data pitch of 0.5 nm, and a scanning speed of 50 nm/min with 1 accumulation. Background spectra were acquired across this same spectral range for 10 mM $NaPO_4$ pH 8.5 (J61151.AP) without protein added and was manually subtracted after conversion. To estimate secondary structure content, deconvolution of far-UV spectra was performed using the Beta Structure Selection (BeStSel, https://bestsel.elte.hu/index.php) Internet server [185]. Percent beta sheets predicted as shown in Fig. 5 were the sum of predictions for parallel, antiparallel, and turn content of each protein.

U.2.7 Differential scanning calorimetry

Protein thermal stability was assessed using a MicroCal PEAQ-DSC Automated calorimeter (Malvern Panalytical). For sample analysis, 325 µL of each sample and matching buffer was loaded into a 96-well deep well plate (Malvern Panalytical WEL190010-010), sealed with a silicone plate seal (Malvern Panalytical WEL190020-010), and loaded into the PEAQ-DSC Peltier stack with the thermostat held at 4°C.

Thermal scans were performed from 20 – 110°C using a scan rate of 210°C/h. At the beginning and end of the run, the sample and reference cells of the calorimeter were cleaned with a 20% w/v Contrad 70 (Decon Labs #1002) using a standard SCAN procedure at the same scan rate. Additionally, every third sample injection a buffer-buffer injection at the same scan rate was performed.

Data analysis was conducted using the dedicated PEAQ DSC Analysis tab (Malvern Panalytical). Baseline correction was performed by subtraction of the corresponding buffer-buffer scan and sample thermograms were further baseline corrected using the spline function to assess pre- and post-dissociation baselines. Peak integration was then performed using a non-two-state model to identify T_{onset}, T_m, and $\Delta H_{cal}/\Delta H_{VH}$.

U.2.8 Protein crystallization and X-ray crystallography

Diffraction quality crystals of UNC_079 were obtained by hanging drop diffusion at 4°C by mixing 750 nL protein (15 mg/ml in 20 mM Tris pH 8, 100 mM NaCl) with 750 nL reservoir solution (2.1 M DL-malic acid, pH 7.0) over 500 µL reservoir. The drop containing crystals was mixed with glycerol to 20% before flash freezing. X-ray diffraction data was collected at 100 K at a wavelength of 0.97624 Å at the PETRA3 synchrotron on the P13 beamline [186] (Helix BioStructures, LLC). The data were processed using DIALS [187] and Aimless [188] in $P4_32_12$ space group with 1 molecule in the ASU. A structure was able to be phased using the Chroma-generated model using

PhaserMR [189] and was fully refined using phenix.refine [190] to a resolution of 1.1 Å. Data collection and refinement statistics are listed in Extended Data Table 2.

Diffraction quality crystals of UNC_239 were obtained by hanging drop diffusion at 4 °C by mixing 750 nL protein (27 mg/ml in 20 mM Tris pH 8, 100 mM NaCl) with 750 nL reservoir solution (0.2 M ammonium acetate, 0.1 M Tris pH 8.5, 25% w/v polyethylene glycol) over 500 µL reservoir. The drop containing crystals was mixed with ethylene glycol to 20% before flash freezing. X-ray diffraction data was collected at 100 K at a wavelength of 0.97624 Å at the PETRA3 synchrotron on the P13 beamline [186]. The data were processed using DIALS [187] and Aimless [188] in $P2_12_12_1$ space group with 2 molecules in the ASU. A structure was able to be phased using the Chroma-generated model using PhaserMR [189] and was fully refined using phenix.refine [190] to a resolution of 2.36 Å. Data collection and refinement statistics are listed in Extended Data Table 2.

U.2.9 *In-silico* score comparison to split-GFP

Split-GFP values from both unconditional design sets were compared to ChromaDesign Pure-Potts/Multi potts and autoregressive negative log-likelihood, ChromaBackbone v0/v1 ELBO, TM-score to predicted AlphaFold, ESMFold and OmegaFold structures, AlphaFold mean pLDDT and FoldSeek highest TM-score to the training set. ESMFold and OmegaFold structure prediction failed for design with lengths above 848 and 631 in the second unconditional set. Experimental values and most of the *in silico* scores have a strong dependency to design length (Supplementary Figure 34 top panel). Scores for the first unconditional set were length normalized by fitting them to design length with LOWESS smoothing and evaluating the Pearson correlation between scores residuals to split-GFP values (Supplementary Figure 34 bottom panel). Moreover, 95% confidence intervals of partial Spearman correlation against each unconditional set were evaluated using the *pingouin* python package[191] with design length being the covariate (Supplementary Figure 35).

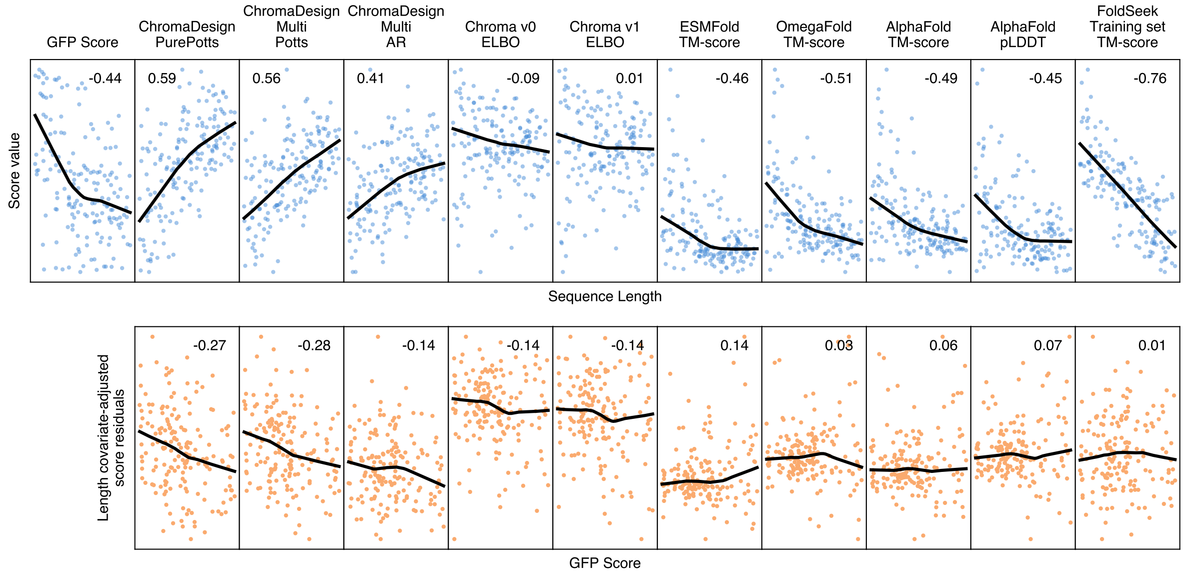

Supplementary Figure 34: ***In silico* scores compared to Unconditional I split-GFP and sequence length. Top**) Scatter plot of each score compared to design length **Bottom**) Score residuals after lowess smoothing compared to split GFP values. Pearson correlation is written in each plot. LOWESS fit shown in black

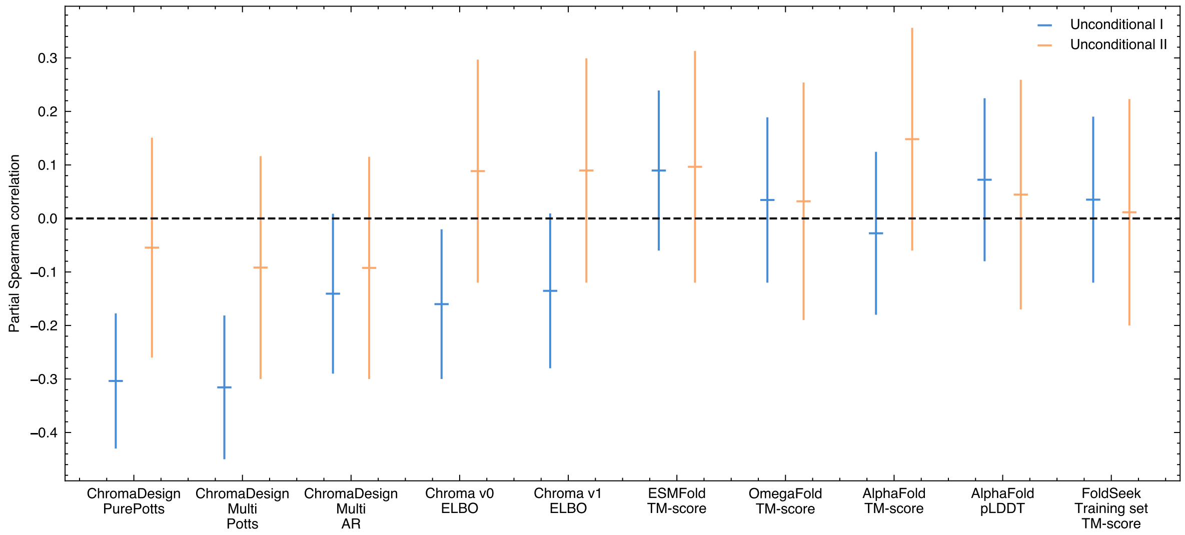

Supplementary Figure 35: ***In silico* scores partial Spearman correlation to split GFP controlling for sequence length** Horizontal bar is the median partial Spearman correlation of each score and the vertical bar their 95% confidence interval.

U.2.10 Novelty assessment of crystallized proteins

Both crystal structures were queried against the PDB (May 2023) for structural homologs with FoldSeek. TM-scores were recomputed for both the query and target using FoldSeek-provided translation and rotation matrices. Internal benchmarking to replicate the CATH coverage analysis with FoldSeek instead of all-vs-all TMalign revealed that the following parameters `--alignment-type 1 --min-seq-id 0 -s 20 -e inf --max-seqs 20000 -k 5 --num-iterations 2` provide the best tradeoff between compute time and retrieval.

U.3 Experimental figures

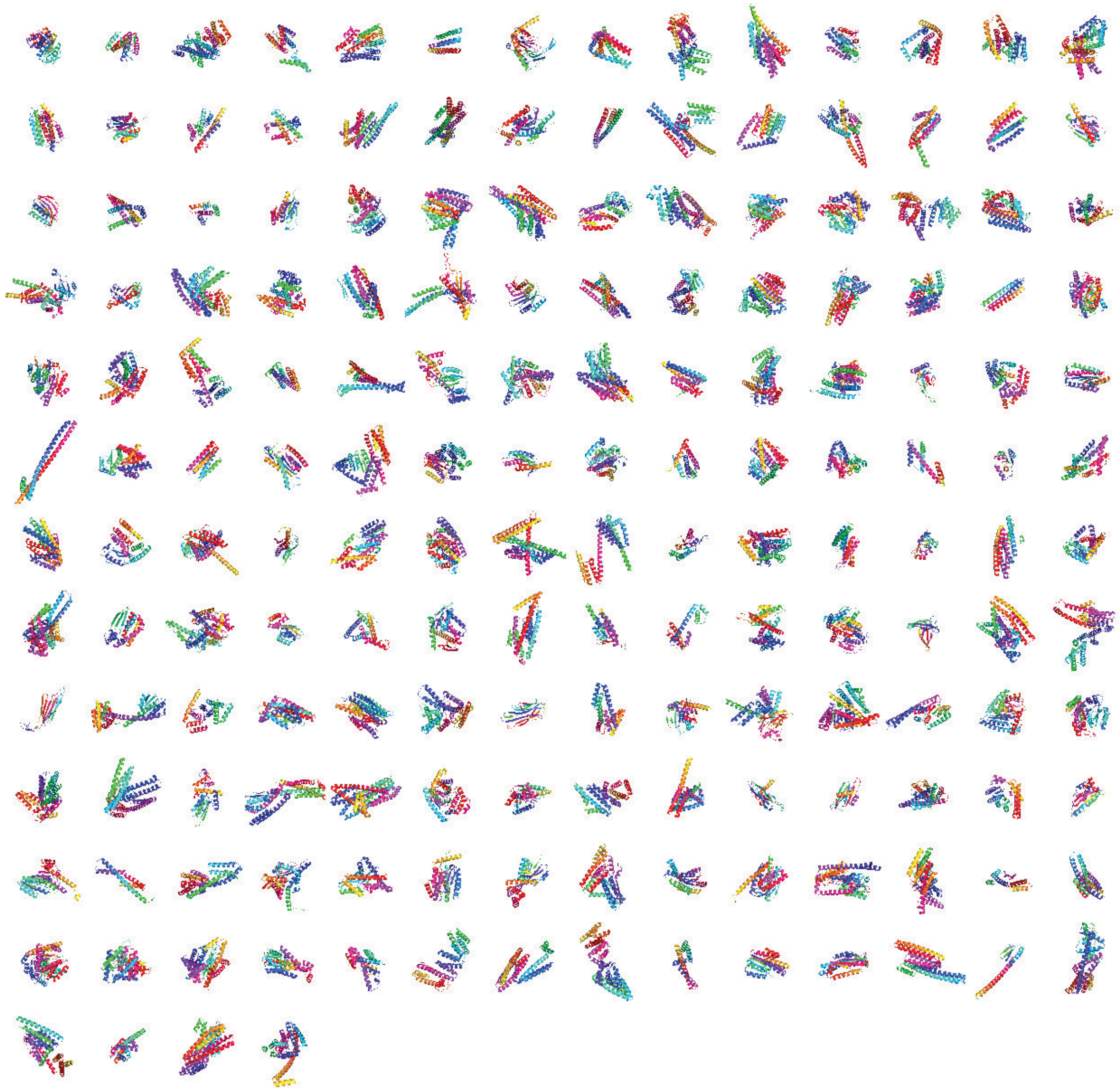

Supplementary Figure 36: **Unconditional protein designs.** 172 unconditional Chroma proteins (UNC_001 through UNC_172) constructed for experimental validation between 100 and 450 amino acids in length.

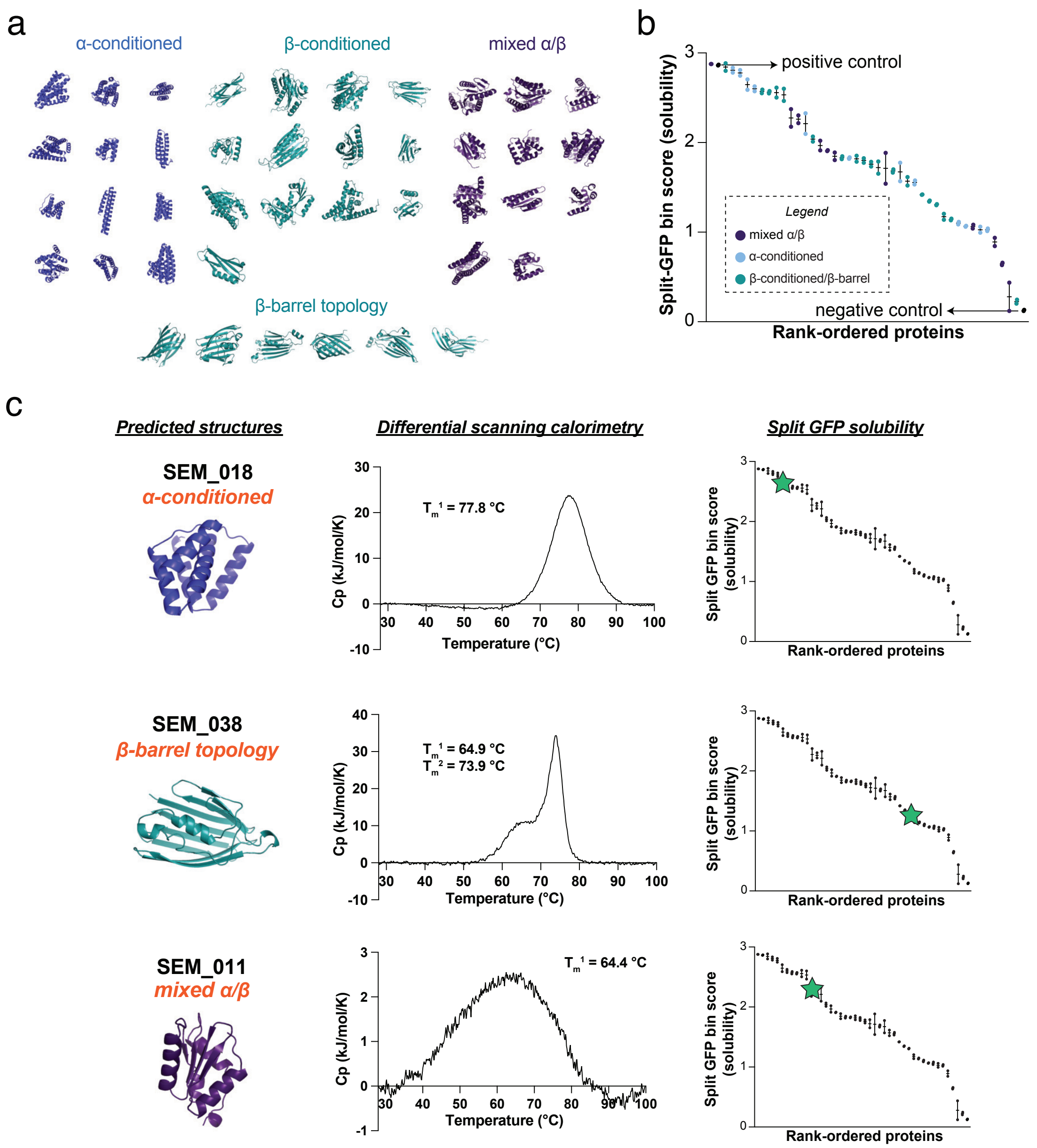

Supplementary Figure 37: **Secondary structure conditional designs.** **a**, 42 proteins were designed based on secondary structure composition (SEM_001 through SEM_042). **b**, Split-GFP rank-ordered bin scores for designed proteins conditioned on secondary structure content. Individual data points for two independent biological replicates shown. **c**, Differential scanning calorimetry data for one protein from each of various secondary structure design classes. Split-GFP data shown for reference.

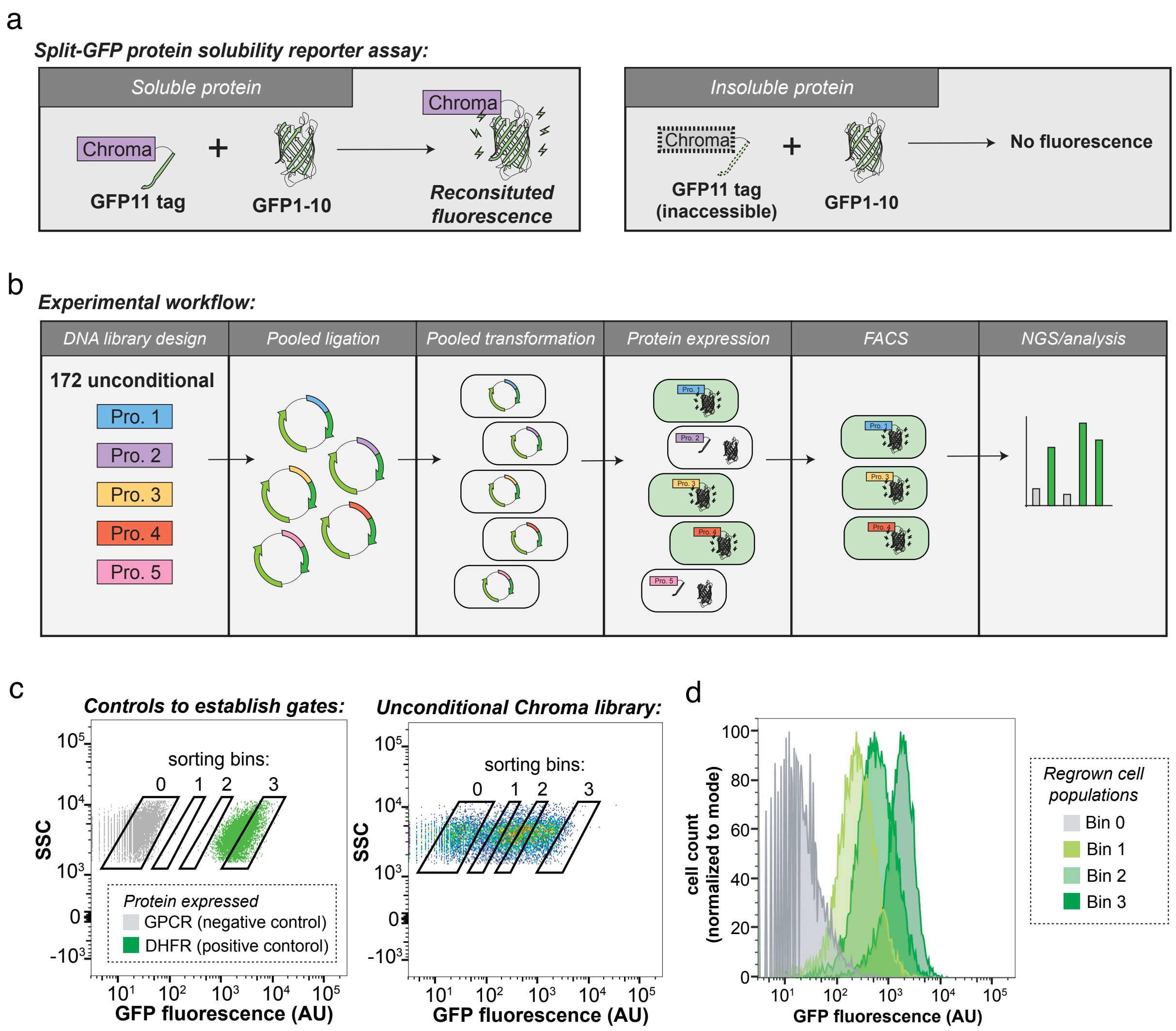

Supplementary Figure 38: **Split-GFP protein solubility assay. a**, Schematic of split-GFP reporter concept. Co-expression of soluble protein fused to the GFP11 tag with the GFP1-10 protein results in restoration of GFP fluorescence. **b**, Split-GFP experimental workflow to determine protein solubility. FACS = fluorescence-activated cell sorting; NGS = next-generation sequencing. **c**, FACS gating strategy informed by positive and negative control cells. Chroma library was sorted into 4 different gates based on GFP fluorescence for subsequent sequencing enrichment analysis. **d**, Flow cytometry of sorted cell populations that were regrown and split-GFP components were re-induced to evaluate signal stability within sorted populations.

Western blots: Streptactin-HRP

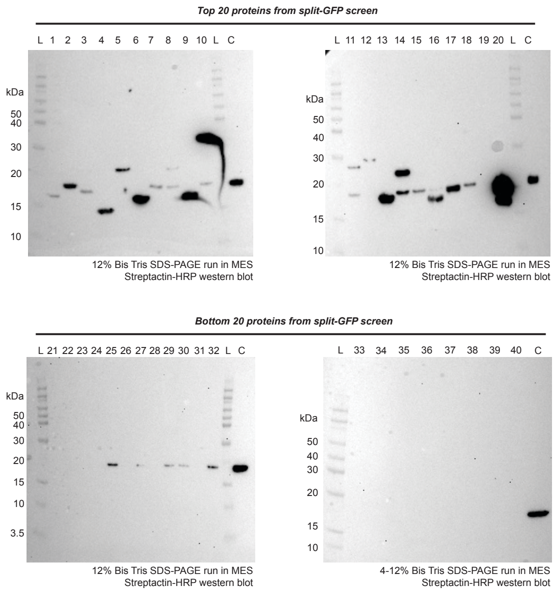

Lane	PRO-ID	MW (kDa)	Observed	
1	UNC_113	15.6	Yes	
2	UNC_163	17.5	Yes	
3	UNC_102	17.7	Yes	
4	UNC_083	14.1	Yes	
5	UNC_159	24.2	Yes	
6	UNC_096	14.9	Yes	
7	UNC_077	18.4	No	
8	UNC_017	22.5	Yes	
9	UNC_153	17.2	Yes	
10	UNC_092	33.4	Yes	Top 20
11	UNC_002	23.3	Yes	
12	UNC_121	27.1	Yes	
13	UNC_079	17.9	Yes	
14	UNC_154	25.0	Yes	
15	UNC_086	31.0	No	
16	UNC_073	17.0	Yes	
17	UNC_018	18.7	Yes	
18	UNC_146	34.4	No	
19	UNC_028	20.5	No	
20	UNC_119	16.1	Yes	
21	UNC_138	31.7	No	
22	UNC_051	34.9	No	
23	UNC_037	44.5	No	
24	UNC_166	32.7	No	
25	UNC_045	52.2	No	
26	UNC_097	31.0	No	
27	UNC_057	36.5	No	
28	UNC_087	45.5	No	
29	UNC_114	38.0	No	
30	UNC_033	42.0	No	Bottom 20
31	UNC_112	49.5	No	
32	UNC_050	33.1	No	
33	UNC_063	41.9	No	
34	UNC_098	27.0	No	
35	UNC_103	22.7	No	
36	UNC_066	44.4	No	
37	UNC_107	17.6	No	
38	UNC_030	25.2	No	
39	UNC_019	40.4	No	
40	UNC_061	32.9	No	

Western blots: Anti-Strep-tag-HRP

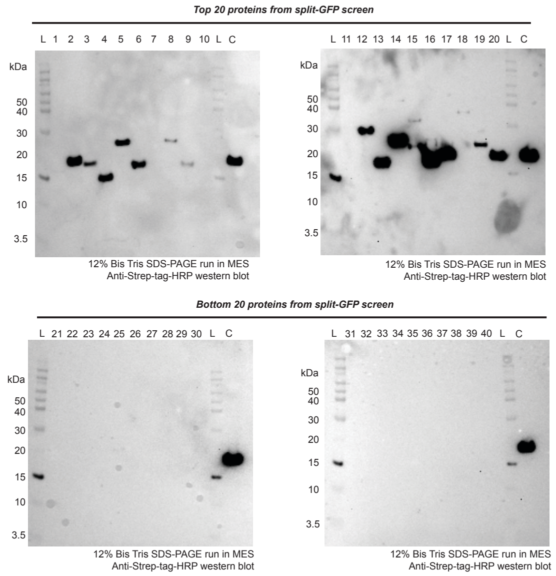

Lane	PRO-ID	MW (kDa)	Observed	
1	UNC_113	15.6	Yes	
2	UNC_163	17.5	Yes	
3	UNC_102	17.7	Yes	
4	UNC_083	14.1	Yes	
5	UNC_159	24.2	Yes	
6	UNC_096	14.9	Yes	
7	UNC_077	18.4	No	
8	UNC_017	22.5	Yes	
9	UNC_153	17.2	Yes	
10	UNC_092	33.4	No	Top 20
11	UNC_002	23.3	No	
12	UNC_121	27.1	Yes	
13	UNC_079	17.9	Yes	
14	UNC_154	25.0	Yes	
15	UNC_086	31.0	Yes	
16	UNC_073	17.0	Yes	
17	UNC_018	18.7	Yes	
18	UNC_146	34.4	Yes	
19	UNC_028	20.5	Yes	
20	UNC_119	16.1	Yes	
21	UNC_138	31.7	No	
22	UNC_051	34.9	No	
23	UNC_037	44.5	No	
24	UNC_166	32.7	No	
25	UNC_045	52.2	No	
26	UNC_097	31.0	No	
27	UNC_057	36.5	No	
28	UNC_087	45.5	No	
29	UNC_114	38.0	No	
30	UNC_033	42.0	No	Bottom 20
31	UNC_112	49.5	No	
32	UNC_050	33.1	No	
33	UNC_063	41.9	No	
34	UNC_098	27.0	No	
35	UNC_103	22.7	No	
36	UNC_066	44.4	No	
37	UNC_107	17.6	No	
38	UNC_030	25.2	No	
39	UNC_019	40.4	No	
40	UNC_061	32.9	No	

Supplementary Figure 39: **Soluble protein expression confirmation via western blot.** The top 20 and bottom 20 hits from the split-GFP solubility screen on proteins UNC_001 through UNC_172 were reformatted to contain a C-terminal Strep-tag. Protein expression from *E. coli* lysates was detected by western blot using Streptactin or anti-Strep-tag antibody. Representative blots shown from two independent biological experiments. Lane designations: L = ladder; C = control protein (same on each blot)

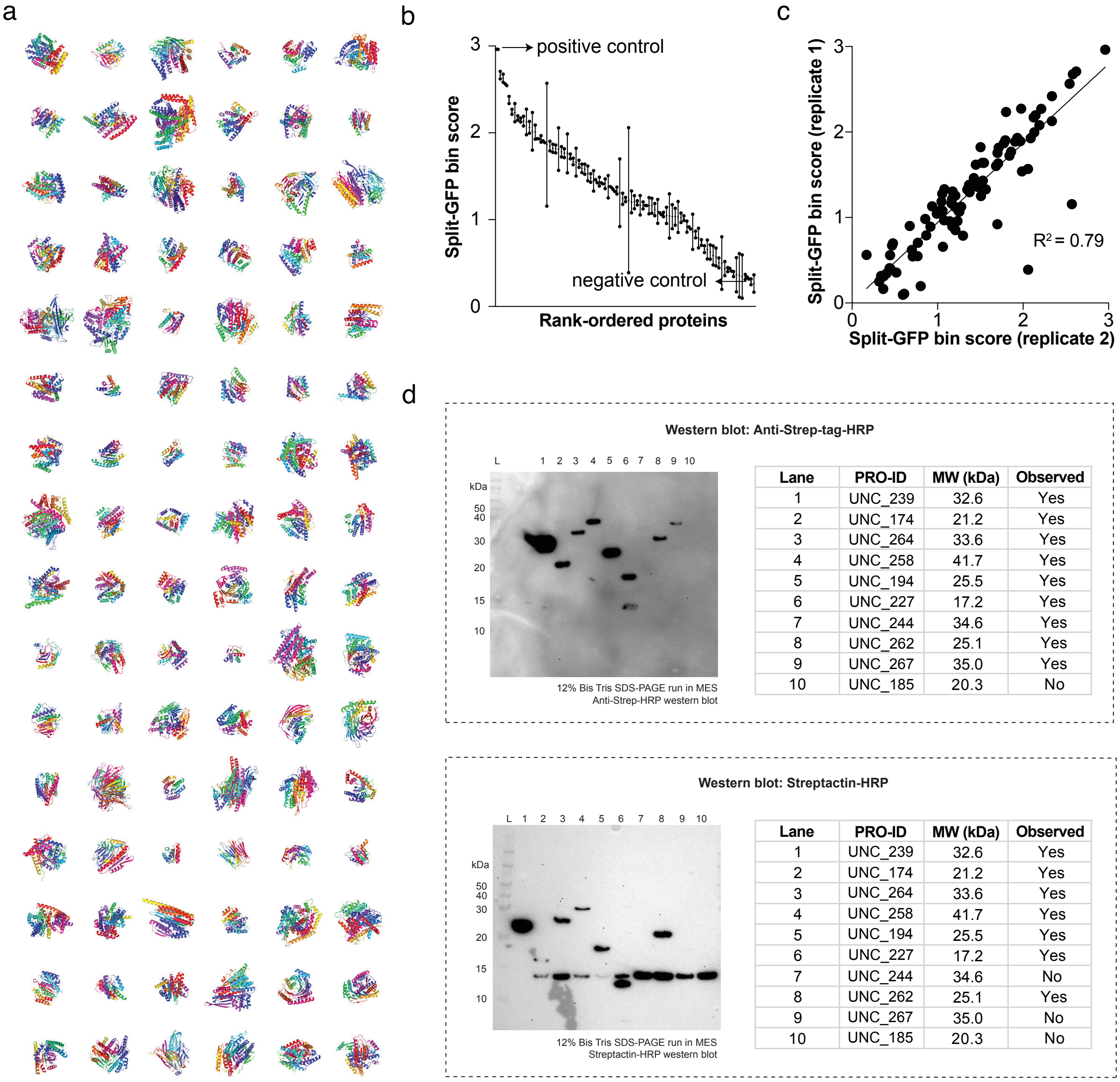

Lane	PRO-ID	MW (kDa)	Observed
1	UNC_239	32.6	Yes
2	UNC_174	21.2	Yes
3	UNC_264	33.6	Yes
4	UNC_258	41.7	Yes
5	UNC_194	25.5	Yes
6	UNC_227	17.2	Yes
7	UNC_244	34.6	Yes
8	UNC_262	25.1	Yes
9	UNC_267	35.0	Yes
10	UNC_185	20.3	No

Lane	PRO-ID	MW (kDa)	Observed
1	UNC_239	32.6	Yes
2	UNC_174	21.2	Yes
3	UNC_264	33.6	Yes
4	UNC_258	41.7	Yes
5	UNC_194	25.5	Yes
6	UNC_227	17.2	Yes
7	UNC_244	34.6	No
8	UNC_262	25.1	Yes
9	UNC_267	35.0	No
10	UNC_185	20.3	No

Supplementary Figure 40: **Evaluation of additional set of unconditional protein designs. a**, 96 additional unconditional designs up to 950 amino acids in length (UNC_173 through UNC_268) were evaluated experimentally. **b**, Rank-ordered split-GFP bin scores for additional unconditional proteins. Individual data points for two biological replicates shown. **c**, Reproducibility of split-GFP bin scores between two independent biological replicates. **d**, Western blot-based confirmation of soluble protein expression from *E.* coli lysates for the top 10 scoring proteins in this set using either Streptactin or anti-Strep-tag antibody for detection. Representative blots shown from two independent biological experiments. Lane designations: L = ladder

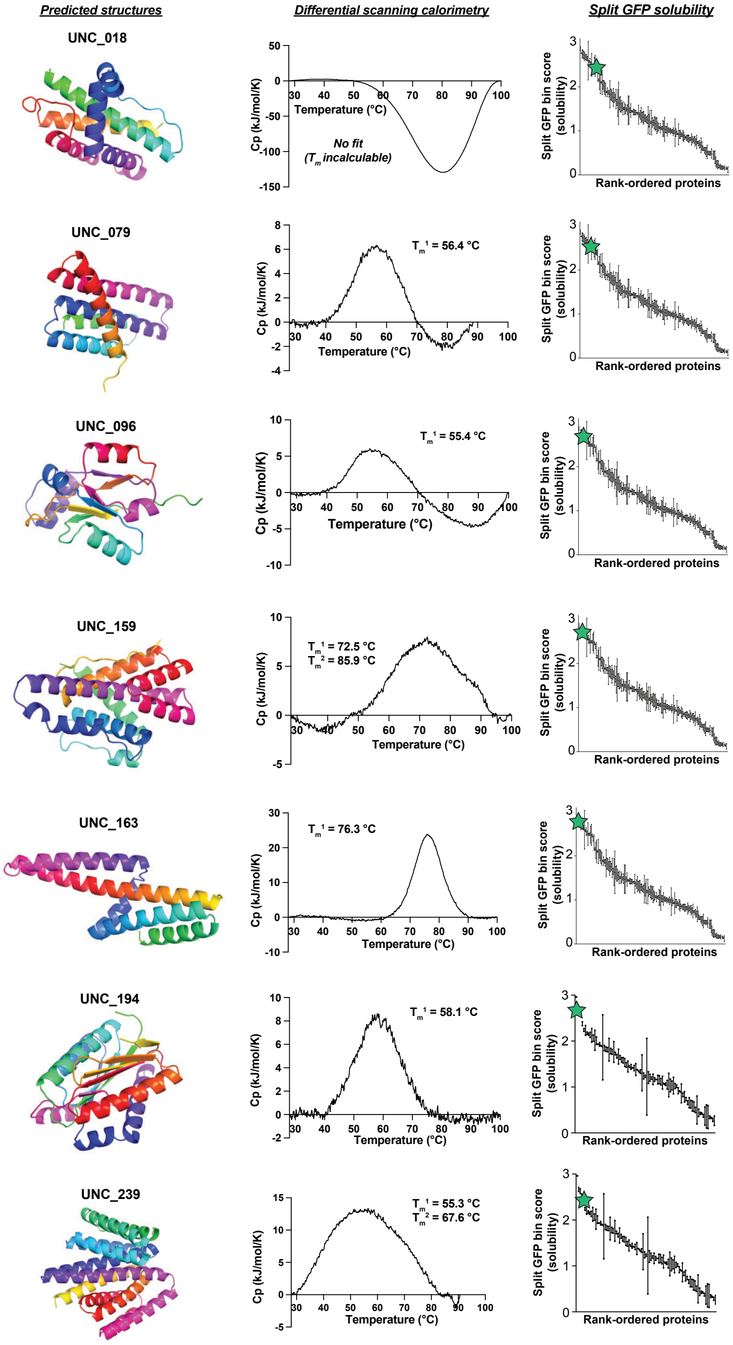

Supplementary Figure 41: **Differential scanning calorimetry experiments.** Evaluation of 7 SEC-purified unconditional proteins by differential scanning calorimetry. Split-GFP solubility score shown for reference, with individual data points for two biological replicates shown.

U.4 Experimental tables

Protein name	DNA seq	Amino acid seq
dihydrofolate reductase	ATGATCAGTCTGATTGCGGCGTTAGCGGTAGATC<br>GCGTTATCGGCATGGAAAACGCCATGCCGTGGAA<br>CCTGCCTGCCGATCTCGCCTGGTTTAAACGCAACA<br>CCTTAAATAAACCCGTGATTATGGGCCGCCATAC<br>CTGGGAATCAATCGGTCGTCCGTTGCCAGGACGC<br>AAAAATATTATCCTCAGCAGTCAACCGGGTACGG<br>ACGATCGCGTAACGTGGGTGAAGTCGGTGGATGA<br>AGCCATCGCGGCGTGTGGTGACGTACCAGAAATC<br>ATGGTGATTGGCGGCGGTCGCGTTTATGAACAGT<br>TCTTGCCAAAAGCGCAAAAACTGTATCTGACGCA<br>TATCGACGCAGAAGTGGAAGGCGACACCCATTTC<br>CCGGATTACGAGCCGGATGACTGGGAATCGGTAT<br>TCAGCGAATTCCACGATGCTGATGCGCAGAACTC<br>TCACAGCTATTGCTTTGAGATTCTGGAGCGGCGG	MISLIAALAVDRVIGMENAMPWNLPADLAWFKRNT<br>LNKPVIMGRHTWESIGRPLPGRKNIILSSQPGTDDRV<br>TWVKSVDEAIAACGDVPEIMVIGGGRVYEQFLPKA<br>QKLYLTHIDAEVEGDTHFPDYEPDDWESVFSEFHDA<br>DAQNSHSYCFEILERR
human beta-3 adrenergic receptor	ATGGCCCCATGGCCGCACGAGAATTCTAGTTTAG<br>CTCCTTGGCCTGATTTGCCCACGCTTGCTCCGAAC<br>ACAGCAAATACTAGTGGGTTACCGGGTGTGCCAT<br>GGGAAGCCGCCCTGGCAGGCGCACTTTTAGCGCT<br>GGCCGTTCTGGCGACAGTTGGCGGAAACCTTTTA<br>GTAATTGTTGCGATCGCTTGGACTCCGCGTTTGCA<br>AACCATGACGAATGTATTCGTGACCTCCTTGGCC<br>GCCGCCGACTTGGTTATGGGCTTGTTAGTTGTACC<br>ACCCGCAGCCACCCTTGCACTTACCGGACACTGG<br>CCCTTGGGAGCAACCGGGTGCGAGTTATGGACAT<br>CCGTAGATGTTCTTTGTGTGACCGCCTCAATTGAA<br>ACGCTGTGTGCATTAGCAGTGGACCGTTACTTGG<br>CTGTAACAAACCCCCTTCGTTACGGTGCACTGGTA<br>ACTAAGCGTTGTGCGCGTACGGCGGTGGTTTTGG<br>TGTGGGTCGTGAGCGCGGCCGTTTCCTTTGCGCCA<br>ATTATGAGTCAGTGGTGGCGTGTAGGTGCCGATG<br>CTGAGGCACAACGTTGTCACTCAAATCCTCGCTGT<br>TGTGCGTTCGCTTCTAACATGCCGTATGTCCTGTT<br>ATCTAGTAGTGTTTCTTTCTACCTGCCCTTATTGG<br>TTATGCTGTTCGTCTATGCTCGTGTGTTCGTTGTA<br>GCGACTCGTCAACTTCGCTTACTTCGCGGAGAATT<br>AGGTCGCTTTCCACCGGAGGAATCCCCTCCTGCCC<br>CATCTCGTAGTTTGGCCCCGGCGCCTGTTGGAACG<br>TGTGCGCCACCAGAAGGTGTTCCGGCATGTGGAC<br>GCCGCCCGGCCCGCTTATTGCCTTTACGTGAACAT<br>CGCGCCTTATGTACGTTAGGACTGATCATGGGGA<br>CTTTTACGCTGTGCTGGCTTCCGTTCTTCCTTGCCA<br>ACGTTCTGCGTGCACTTGGCGGCCCATCACTGGTT<br>CCTGGACCCGCGTTCCTGGCTCTTAACTGGTTGGG<br>CTATGCGAACTCTGCATTCAATCCTTTAATCTATT<br>GCCGCTCCCCCGATTTCCGCTCGGCGTTTCGCCGT<br>TTGTTGTGTCGCTGCGGACGCCGTTTGCCCCCGGA<br>ACCATGCGCCGCAGCGCGTCCCGCGTTATTTCCAT<br>CCGGCGTGCCAGCGGCGCGCTCATCTCCGGCGCA<br>ACCGCGTCTGTGCCAACGCCTGGACGGTGCCTCA<br>TGGGGGGTTTCT	MAPWPHENSSLAPWPDLPTLAPNTANTSGLPGVPW<br>EAALAGALLALAVLATVGGNLLVIVAIAWTPRLQT<br>MTNVFVTSLAAADLVMGLLVVPPAATLALTGHWP<br>LGATGCELWTSVDVLCVTASIETLCALAVDRYLAV<br>TNPLRYGALVTKRCARTAVVLVWVVSAAVSFAPIM<br>SQWWRVGADAEAQRCHSNPRCCAFASNMPYVLLS<br>SSVSFYLPLLVMLFVYARVFVVATRQLRLLRGELGR<br>FPPEESPPAPSRSLAPAPVGTCAPPEGVPACGRRPAR<br>LLPLREHRALCTLGLIMGTFTLCWLPFFLANVLRAL<br>GGPSLVPGPAFLALNWLGYANSAFNPLIYCRSPDFR<br>SAFRRLLCRCGRRLPPEPCAAARPALFPSGVPAARSS<br>PAQPRLCQRLDGASWGVS

Supplementary Table 8: **Control sequences used in split-GFP assay**

References

54. Sohl-Dickstein, J., Weiss, E., Maheswaranathan, N. & Ganguli, S. *Deep unsupervised learning using nonequilibrium thermodynamics* in *International Conference on Machine Learning* (2015), 2256–2265.
55. Song, Y. *et al. Score-Based Generative Modeling through Stochastic Differential Equations* in *International Conference on Learning Representations* (2021). `https://openreview.net/forum?id=PxTIG12RRHS`.
56. Murphy, K. P. *Machine learning: a probabilistic perspective* (MIT press, 2012).
57. Ho, J., Jain, A. & Abbeel, P. Denoising diffusion probabilistic models. *Advances in Neural Information Processing Systems* **33,** 6840–6851 (2020).
58. Lipman, Y., Chen, R. T., Ben-Hamu, H., Nickel, M. & Le, M. *Flow Matching for Generative Modeling* in *The Eleventh International Conference on Learning Representations* (2022).
59. Liu, X., Gong, C. & Liu, Q. Flow straight and fast: Learning to generate and transfer data with rectified flow. *arXiv preprint arXiv:2209.03003* (2022).
60. Albergo, M. S., Boffi, N. M. & Vanden-Eijnden, E. Stochastic interpolants: A unifying framework for flows and diffusions. *arXiv preprint arXiv:2303.08797* (2023).
61. Särkkä, S. & Solin, A. *Applied stochastic differential equations* (Cambridge University Press, 2019).
62. Kingma, D., Salimans, T., Poole, B. & Ho, J. Variational diffusion models. *Advances in neural information processing systems* **34,** 21696–21707 (2021).
63. Nichol, A. Q. & Dhariwal, P. *Improved denoising diffusion probabilistic models* in *International Conference on Machine Learning* (2021), 8162–8171.
64. Kong, X., Brekelmans, R. & Ver Steeg, G. *Information-Theoretic Diffusion* in *The Eleventh International Conference on Learning Representations* (2022).
65. Karras, T., Aittala, M., Aila, T. & Laine, S. Elucidating the design space of diffusion-based generative models. *arXiv preprint arXiv:2206.00364* (2022).
66. Jumper, J. *et al.* Highly accurate protein structure prediction with AlphaFold. *Nature* **596,** 583–589 (2021).
67. Anand, N. & Achim, T. Protein Structure and Sequence Generation with Equivariant Denoising Diffusion Probabilistic Models. *arXiv preprint arXiv:2205.15019* (2022).
68. Kabsch, W. A solution for the best rotation to relate two sets of vectors. *Acta Crystallographica Section A: Crystal Physics, Diffraction, Theoretical and General Crystallography* **32,** 922–923 (1976).
69. Coutsias, E. A., Seok, C. & Dill, K. A. Using quaternions to calculate RMSD. *Journal of computational chemistry* **25,** 1849–1857 (2004).
70. Anderson, B. D. Reverse-time diffusion equation models. *Stochastic Processes and their Applications* **12,** 313–326 (1982).
71. Maoutsa, D., Reich, S. & Opper, M. Interacting particle solutions of Fokker–Planck equations through gradient–log–density estimation. *Entropy* **22,** 802 (2020).
72. Chen, R. T., Rubanova, Y., Bettencourt, J. & Duvenaud, D. K. Neural ordinary differential equations. *Advances in neural information processing systems* **31** (2018).
73. Grathwohl, W., Chen, R. T., Bettencourt, J., Sutskever, I. & Duvenaud, D. *FFJORD: Free-Form Continuous Dynamics for Scalable Reversible Generative Models* in *International Conference on Learning Representations* (2018).

74. Jing, B., Corso, G., Berlinghieri, R. & Jaakkola, T. *Subspace diffusion generative models* in *European Conference on Computer Vision* (2022), 274–289.
75. Rombach, R., Blattmann, A., Lorenz, D., Esser, P. & Ommer, B. *High-resolution image synthesis with latent diffusion models* in *Proceedings of the IEEE/CVF conference on computer vision and pattern recognition* (2022), 10684–10695.
76. Kingma, D. P. & Dhariwal, P. Glow: Generative flow with invertible 1x1 convolutions. *Advances in neural information processing systems* **31** (2018).
77. Holtzman, A., Buys, J., Du, L., Forbes, M. & Choi, Y. *The Curious Case of Neural Text Degeneration* in *International Conference on Learning Representations* (2020). `https://openreview.net/forum?id=rygGQyrFvH`.
78. Kool, W., Van Hoof, H. & Welling, M. *Stochastic beams and where to find them: The gumbel-top-k trick for sampling sequences without replacement* in *International Conference on Machine Learning* (2019), 3499–3508.
79. MacKay, D. J. *Information theory, inference and learning algorithms* (Cambridge university press, 2003).
80. Dhariwal, P. & Nichol, A. Diffusion models beat gans on image synthesis. *Advances in Neural Information Processing Systems* **34,** 8780–8794 (2021).
81. Ho, J. & Salimans, T. *Classifier-Free Diffusion Guidance* in *NeurIPS 2021 Workshop on Deep Generative Models and Downstream Applications* (2021). `https://openreview.net/forum?id=qw8AKxfYbI`.
82. Saharia, C. *et al.* Photorealistic text-to-image diffusion models with deep language understanding. *Advances in Neural Information Processing Systems* **35,** 36479–36494 (2022).
83. Ramesh, A., Dhariwal, P., Nichol, A., Chu, C. & Chen, M. Hierarchical text-conditional image generation with clip latents. *arXiv preprint arXiv:2204.06125* (2022).
84. Du, Y. *et al. Reduce, reuse, recycle: Compositional generation with energy-based diffusion models and mcmc* in *International Conference on Machine Learning* (2023), 8489–8510.
85. Song, Y. & Ermon, S. Generative modeling by estimating gradients of the data distribution. *Advances in Neural Information Processing Systems* **32** (2019).
86. Marinari, E. & Parisi, G. Simulated tempering: a new Monte Carlo scheme. *EPL (Europhysics Letters)* **19,** 451 (1992).
87. Hansmann, U. H. Parallel tempering algorithm for conformational studies of biological molecules. *Chemical Physics Letters* **281,** 140–150 (1997).
88. Hong, L. & Lei, J. Scaling law for the radius of gyration of proteins and its dependence on hydrophobicity. *Journal of Polymer Science Part B: Polymer Physics* **47,** 207–214. `https://onlinelibrary.wiley.com/doi/abs/10.1002/polb.21634` (2009).
89. Tanner, J. J. Empirical power laws for the radii of gyration of protein oligomers. *Acta Crystallographica Section D: Structural Biology* **72,** 1119–1129 (2016).
90. Perunov, N. & England, J. L. Quantitative theory of hydrophobic effect as a driving force of protein structure. *Protein Science* **23,** 387–399 (2014).
91. Trippe, B. L. *et al. Diffusion Probabilistic Modeling of Protein Backbones in 3D for the motif-scaffolding problem* in *The Eleventh International Conference on Learning Representations* (2022).
92. Gilmer, J., Schoenholz, S. S., Riley, P. F., Vinyals, O. & Dahl, G. E. *Neural message passing for quantum chemistry* in *International conference on machine learning* (2017), 1263–1272.

93. Barnes, J. & Hut, P. A hierarchical $\mathcal{O}(N \log N)$ force-calculation algorithm. *Nature* **324,** 446–449 (1986).
94. Battaglia, P. W. *et al.* Relational inductive biases, deep learning, and graph networks. *CoRR* **abs/1806.01261.** `http://arxiv.org/abs/1806.01261` (2018).
95. Ingraham, J., Garg, V., Barzilay, R. & Jaakkola, T. Generative models for graph-based protein design. *Advances in neural information processing systems* **32** (2019).
96. Vaswani, A. *et al.* Attention is all you need. *Advances in neural information processing systems* **30** (2017).
97. Child, R., Gray, S., Radford, A. & Sutskever, I. Generating long sequences with sparse transformers. *arXiv preprint arXiv:1904.10509* (2019).
98. Zaheer, M. *et al.* Big bird: Transformers for longer sequences. *Advances in Neural Information Processing Systems* **33,** 17283–17297 (2020).
99. Yang, J. *et al.* *Focal Attention for Long-Range Interactions in Vision Transformers* in *Advances in Neural Information Processing Systems* **34** (Curran Associates, Inc., 2021), 30008–30022. `https://proceedings.neurips.cc/paper/2021/file/fc1a36821b02abbd2503fd949bfc91` `Paper.pdf`.
100. Van den Oord, A. *et al.* *WaveNet: A Generative Model for Raw Audio* in *9th ISCA Speech Synthesis Workshop* (2016), 125–125.
101. AlQuraishi, M. End-to-end differentiable learning of protein structure. *Cell systems* **8,** 292–301 (2019).
102. Wu, K. E. *et al.* Protein structure generation via folding diffusion. *arXiv preprint arXiv:2209.15611* (2022).
103. Anand, N. & Huang, P. Generative modeling for protein structures. *Advances in neural information processing systems* **31** (2018).
104. Senior, A. W. *et al.* Improved protein structure prediction using potentials from deep learning. *Nature* **577,** 706–710 (2020).
105. Marks, D. S., Hopf, T. A. & Sander, C. Protein structure prediction from sequence variation. *Nature biotechnology* **30,** 1072–1080 (2012).
106. Ingraham, J., Riesselman, A., Sander, C. & Marks, D. *Learning protein structure with a differentiable simulator* in *International Conference on Learning Representations* (2018).
107. Belanger, D. & McCallum, A. *Structured prediction energy networks* in *International Conference on Machine Learning* (2016), 983–992.
108. Schoenholz, S. & Cubuk, E. D. Jax md: a framework for differentiable physics. *Advances in Neural Information Processing Systems* **33,** 11428–11441 (2020).
109. Wang, W., Axelrod, S. & Gómez-Bombarelli, R. *Differentiable Molecular Simulations for Control and Learning* in *ICLR 2020 Workshop on Integration of Deep Neural Models and Differential Equations* (2020).
110. Lin, Z. *et al.* Language models of protein sequences at the scale of evolution enable accurate structure prediction. *bioRxiv.* eprint: `https://www.biorxiv.org/content/early/2022/07/21/2022.07.20.500902.full.pdf`. `https://www.biorxiv.org/content/early/2022/07/21/2022.07.20.500902` (2022).
111. Wu, R. *et al.* High-resolution de novo structure prediction from primary sequence. *bioRxiv.* eprint: `https://www.biorxiv.org/content/early/2022/07/22/2022.07.21.500999.full.pdf`. `https://www.biorxiv.org/content/early/2022/07/22/2022.07.21.500999` (2022).

112. Murphy, K. P. Conjugate Bayesian analysis of the Gaussian distribution. *def* **1,** 16 (2007).
113. Anand, N. *et al.* Protein sequence design with a learned potential. *Nature communications* **13,** 1–11 (2022).
114. Li, A. J., Sundar, V., Grigoryan, G. & Keating, A. E. TERMinator: A Neural Framework for Structure-Based Protein Design using Tertiary Repeating Motifs. *arXiv preprint arXiv:2204.13048* (2022).
115. Hsu, C. *et al. Learning inverse folding from millions of predicted structures* in *International Conference on Machine Learning* (2022), 8946–8970.
116. Dauparas, J. *et al.* Robust deep learning–based protein sequence design using Protein-MPNN. *Science* **378,** 49–56 (2022).
117. Lin, Y. & AlQuraishi, M. Generating novel, designable, and diverse protein structures by equivariantly diffusing oriented residue clouds. *arXiv preprint arXiv:2301.12485* (2023).
118. Yim, J. *et al.* SE (3) diffusion model with application to protein backbone generation. *arXiv preprint arXiv:2302.02277* (2023).
119. Watson, J. L. *et al.* De novo design of protein structure and function with RFdiffusion. *Nature,* 1–3 (2023).
120. Chu, A. E., Cheng, L., El Nesr, G., Xu, M. & Huang, P.-S. An all-atom protein generative model. *bioRxiv,* 2023–05 (2023).
121. Lisanza, S. L. *et al.* Joint generation of protein sequence and structure with RoseTTAFold sequence space diffusion. *bioRxiv,* 2023–05 (2023).
122. Verkuil, R. *et al.* Language models generalize beyond natural proteins. *bioRxiv,* 2022–12 (2022).
123. Wells, B. A. & Chaffee, A. L. Ewald summation for molecular simulations. *Journal of chemical theory and computation* **11,** 3684–3695 (2015).
124. Solomonoff, R. J. A formal theory of inductive inference. Part I. *Information and control* **7,** 1–22 (1964).
125. Grathwohl, W., Swersky, K., Hashemi, M., Duvenaud, D. & Maddison, C. *Oops i took a gradient: Scalable sampling for discrete distributions* in *International Conference on Machine Learning* (2021), 3831–3841.
126. Berman, H. M. The Protein Data Bank. *Nucleic Acids Research* **28,** 235–242. `https://doi.org/10.1093/nar/28.1.235` (Jan. 2000).
127. Edgar, R. C. Search and clustering orders of magnitude faster than BLAST. *Bioinformatics* **26,** 2460–2461. ISSN: 1367-4803. eprint: `https://academic.oup.com/bioinformatics/article-pdf/26/19/2460/16896486/btq461.pdf`. `https://doi.org/10.1093/bioinformatics/btq461` (Aug. 2010).
128. Chaudhury, S., Lyskov, S. & Gray, J. J. PyRosetta: a script-based interface for implementing molecular modeling algorithms using Rosetta. *Bioinformatics* **26,** 689–691 (2010).
129. Mistry, J. *et al.* Pfam: The protein families database in 2021. *Nucleic acids research* **49,** D412–D419 (2021).
130. Bateman, A. *et al.* UniProt: the universal protein knowledgebase in 2021. *Nucleic Acids Research* **49,** D480–D489. `https://doi.org/10.1093/nar/gkaa1100` (Nov. 2020).
131. Steinegger, M. & Söding, J. MMseqs2 enables sensitive protein sequence searching for the analysis of massive data sets. *Nature biotechnology* **35,** 1026–1028 (2017).
132. Kingma, D. P. & Ba, J. Adam: A method for stochastic optimization. *arXiv preprint arXiv:1412.6980* (2014).

133. Song, Y. & Ermon, S. Improved techniques for training score-based generative models. *Advances in neural information processing systems* **33,** 12438–12448 (2020).
134. Zhang, H. *et al.* Predicting protein inter-residue contacts using composite likelihood maximization and deep learning. *BMC bioinformatics* **20,** 1–11 (2019).
135. Wootton, J. C. & Federhen, S. Statistics of local complexity in amino acid sequences and sequence databases. *Computers & Chemistry* **17,** 149–163. `https://doi.org/10.1016/0097-8485(93)85006-x` (June 1993).
136. Plaxco, K. W., Simons, K. T. & Baker, D. Contact order, transition state placement and the refolding rates of single domain proteins 1 1Edited by P. E. Wright. *Journal of Molecular Biology* **277,** 985–994. `https://doi.org/10.1006/jmbi.1998.1645` (Apr. 1998).
137. McInnes, L., Healy, J., Saul, N. & Großberger, L. UMAP: Uniform Manifold Approximation and Projection. *Journal of Open Source Software* **3,** 861. `https://doi.org/10.21105/joss.00861` (2018).
138. Røgen, P. & Fain, B. Automatic classification of protein structure by using Gauss integrals. *Proceedings of the National Academy of Sciences* **100,** 119–124 (2003).
139. Harder, T., Borg, M., Boomsma, W., Røgen, P. & Hamelryck, T. Fast large-scale clustering of protein structures using Gauss integrals. *Bioinformatics* **28,** 510–515 (2012).
140. Frishman, D. & Argos, P. Knowledge-based protein secondary structure assignment. *Proteins* **23,** 566–579 (1995).
141. Ivankov, D. *et al.* Contact order revisited: influence of protein size on the folding rate. *Protein Sci* **12,** 2057–2062 (2003).
142. Mackenzie, C. O., Zhou, J. & Grigoryan, G. Tertiary alphabet for the observable protein structural universe. *Proceedings of the National Academy of Sciences* **113** (Nov. 2016).
143. Zheng, F., Zhang, J. & Grigoryan, G. Tertiary Structural Propensities Reveal Fundamental Sequence/Structure Relationships. *Structure* **23,** 961–971. `https://doi.org/10.1016/j.str.2015.03.015` (May 2015).
144. Zhou, J., Panaitiu, A. E. & Grigoryan, G. A general-purpose protein design framework based on mining sequence–structure relationships in known protein structures. *Proceedings of the National Academy of Sciences* **117,** 1059–1068 (2019).
145. Sillitoe, I. *et al.* CATH: increased structural coverage of functional space. *Nucleic acids research* **49,** D266–D273 (2021).
146. Van Kempen, M. *et al.* Fast and accurate protein structure search with Foldseek. *Nature Biotechnology,* 1–4 (2023).
147. Borg, M. *et al.* A probabilistic approach to protein structure prediction: PHAISTOS in CASP9. *LASR2009-Statistical tools for challenges in bioinformatics,* 65–70 (2009).
148. Zhang, Y. & Skolnick, J. TM-align: a protein structure alignment algorithm based on the TM-score. *Nucleic acids research* **33,** 2302–2309 (2005).
149. Evans, R. *et al.* Protein complex prediction with AlphaFold-Multimer. *BioRxiv,* 2021–10 (2021).
150. Lin, Z. *et al.* Evolutionary-scale prediction of atomic-level protein structure with a language model. *Science* **379,** 1123–1130 (2023).
151. Simons, K. T., Kooperberg, C., Huang, E. & Baker, D. Assembly of protein tertiary structures from fragments with similar local sequences using simulated annealing and Bayesian scoring functions. *J. Mol. Biol.* **268,** 209–225 (Apr. 1997).

152. Rhodes, B. & Gutmann, M. U. Enhanced gradient-based MCMC in discrete spaces. *Transactions on Machine Learning Research* (2022).
153. Salimans, T. & Ho, J. *Should EBMs model the energy or the score?* in *Energy Based Models Workshop-ICLR 2021* (2021).
154. Bennett, C. H. Mass tensor molecular dynamics. *Journal of Computational Physics* **19,** 267–279. ISSN: 0021-9991. `https://www.sciencedirect.com/science/article/pii/0021999175900777` (1975).
155. Li, C., Chen, C., Carlson, D. & Carin, L. *Preconditioned stochastic gradient Langevin dynamics for deep neural networks* in *Proceedings of the AAAI conference on artificial intelligence* **30** (2016).
156. Team, S. D. *et al.* Stan modeling language users guide and reference manual. *Technical report* (2016).
157. Gemici, M. C., Rezende, D. & Mohamed, S. *Normalizing Flows on Riemannian Manifolds* 2016. arXiv: `1611.02304 [stat.ML]`.
158. Hie, B. *et al.* A high-level programming language for generative protein design. *bioRxiv,* 2022–12 (2022).
159. Poole, B., Jain, A., Barron, J. T. & Mildenhall, B. Dreamfusion: Text-to-3d using 2d diffusion. *arXiv preprint arXiv:2209.14988* (2022).
160. Hsieh, Y.-P., Kavis, A., Rolland, P. & Cevher, V. Mirrored langevin dynamics. *Advances in Neural Information Processing Systems* **31** (2018).
161. Liu, G.-H., Chen, T., Theodorou, E. A. & Tao, M. Mirror Diffusion Models for Constrained and Watermarked Generation. *stat* **1050,** 2 (2023).
162. Ho, J. *et al. Video Diffusion Models* 2022. arXiv: `2204.03458 [cs.CV]`.
163. Zhou, J. & Grigoryan, G. Rapid search for tertiary fragments reveals protein sequence-structure relationships. *Protein Sci.* **24,** 508–524 (Apr. 2015).
164. Zhou, J. & Grigoryan, G. A C++ library for protein sub-structure search. *bioRxiv preprint 2020.04.26.062612* (2020).
165. Goodsell, D. S. & Olson, A. J. Structural symmetry and protein function. *Annual review of biophysics and biomolecular structure* **29,** 105 (2000).
166. Hsia, Y. *et al.* Design of a hyperstable 60-subunit protein icosahedron. *Nature* **535,** 136–139 (2016).
167. Cohen, T. & Welling, M. *Group equivariant convolutional networks* in *International conference on machine learning* (2016), 2990–2999.
168. Cox, S. & White, A. D. Symmetric Molecular Dynamics. *arXiv preprint arXiv:2204.01114* (2022).
169. Zabrodsky, H., Peleg, S. & Avnir, D. Continuous symmetry measures. *Journal of the American Chemical Society* **114,** 7843–7851 (1992).
170. McWeeny, R. *Symmetry: An introduction to group theory and its applications* (Courier Corporation, 2002).
171. Vincent, A. *Molecular symmetry and group theory: a programmed introduction to chemical applications* (John Wiley & Sons, 2013).
172. Zee, A. *Group theory in a nutshell for physicists* (Princeton University Press, 2016).
173. Harvey, S. C., Tan, R. K.-Z. & Cheatham III, T. E. The flying ice cube: velocity rescaling in molecular dynamics leads to violation of energy equipartition. *Journal of computational chemistry* **19,** 726–740 (1998).

174. Peyré, G., Cuturi, M., *et al.* Computational optimal transport: With applications to data science. *Foundations and Trends® in Machine Learning* **11,** 355–607 (2019).
175. Solomon, J., Peyré, G., Kim, V. G. & Sra, S. Entropic metric alignment for correspondence problems. *ACM Transactions on Graphics (ToG)* **35,** 1–13 (2016).
176. Alvarez-Melis, D. & Jaakkola, T. S. *Gromov-Wasserstein Alignment of Word Embedding Spaces* in *EMNLP* (2018).
177. Tancik, M. *et al.* Fourier features let networks learn high frequency functions in low dimensional domains. *Advances in Neural Information Processing Systems* **33,** 7537–7547 (2020).
178. Consortium, T. U. UniProt: the universal protein knowledgebase in 2021. *Nucleic Acids Research* **49,** D480–D489. `https://doi.org/10.1093/nar/gkaa1100` (Nov. 2020).
179. Black, S., Gao, L., Wang, P., Leahy, C. & Biderman, S. *GPT-Neo: Large Scale Autoregressive Language Modeling with Mesh-Tensorflow* version 1.0. Mar. 2021. `https://doi.org/10.5281/zenodo.5297715`.
180. Gao, L. *et al.* The Pile: An 800GB Dataset of Diverse Text for Language Modeling. *CoRR* **abs/2101.00027.** arXiv: `2101.00027`. `https://arxiv.org/abs/2101.00027` (2021).
181. Lester, B., Al-Rfou, R. & Constant, N. *The Power of Scale for Parameter-Efficient Prompt Tuning* in *Proceedings of the 2021 Conference on Empirical Methods in Natural Language Processing* (2021), 3045–3059.
182. Sawyer, N. *et al.* Designed phosphoprotein recognition in Escherichia coli. *ACS chemical biology* **9,** 2502–2507 (2014).
183. Shen, W., Le, S., Li, Y. & Hu, F. SeqKit: a cross-platform and ultrafast toolkit for FASTA/Q file manipulation. *PloS one* **11,** e0163962 (2016).
184. Li, H. Minimap2: pairwise alignment for nucleotide sequences. *Bioinformatics* **34,** 3094–3100 (2018).
185. Micsonai, A. *et al.* BeStSel: webserver for secondary structure and fold prediction for protein CD spectroscopy. *Nucleic Acids Research* **50,** W90–W98. ISSN: 0305-1048. eprint: `https://academic.oup.com/nar/article-pdf/50/W1/W90/44378197/gkac345.pdf`. `https://doi.org/10.1093/nar/gkac345` (May 2022).
186. Cianci, M. *et al.* P13, the EMBL macromolecular crystallography beamline at the low-emittance PETRA III ring for high-and low-energy phasing with variable beam focusing. *Journal of synchrotron radiation* **24,** 323–332 (2017).
187. Kabsch, W. xds. *Acta Crystallographica Section D: Biological Crystallography* **66,** 125–132 (2010).
188. Evans, P. R. & Murshudov, G. N. How good are my data and what is the resolution? *Acta Crystallographica Section D: Biological Crystallography* **69,** 1204–1214 (2013).
189. McCoy, A. J. *et al.* Phaser crystallographic software. *Journal of applied crystallography* **40,** 658–674 (2007).
190. Adams, P. D. *et al.* PHENIX: a comprehensive Python-based system for macromolecular structure solution. *Acta Crystallographica Section D: Biological Crystallography* **66,** 213–221 (2010).
191. Vallat, R. Pingouin: statistics in Python. *Journal of Open Source Software* **3,** 1026. `https://doi.org/10.21105/joss.01026` (2018).
