# Supplementary material for: Illuminating protein space with a programmable generative model
Source: Nature. 2023 Nov 15;623(7989):1070–8. doi: 10.1038/s41586-023-06728-8 (PMC10686827; doi:10.1038/s41586-023-06728-8)
Supplement: Supplementary file 2 — Reporting Summary [file 41586_2023_6728_MOESM2_ESM.pdf]

nature portfolio

Corresponding author(s): Gevorg Grigoryan

Last updated by author(s): 08/24/2023

Reporting Summary

Nature Portfolio wishes to improve the reproducibility of the work that we publish. This form provides structure for consistency and transparency in reporting. For further information on Nature Portfolio policies, see our Editorial Policies and the Editorial Policy Checklist.

Statistics

For all statistical analyses, confirm that the following items are present in the figure legend, table legend, main text, or Methods section.

n/a	Confirmed	
☐	☒	The exact sample size (*n*) for each experimental group/condition, given as a discrete number and unit of measurement
☐	☒	A statement on whether measurements were taken from distinct samples or whether the same sample was measured repeatedly
☒	☐	The statistical test(s) used AND whether they are one- or two-sided<br>*Only common tests should be described solely by name; describe more complex techniques in the Methods section.*
☐	☒	A description of all covariates tested
☒	☐	A description of any assumptions or corrections, such as tests of normality and adjustment for multiple comparisons
☐	☒	A full description of the statistical parameters including central tendency (e.g. means) or other basic estimates (e.g. regression coefficient) AND variation (e.g. standard deviation) or associated estimates of uncertainty (e.g. confidence intervals)
☒	☐	For null hypothesis testing, the test statistic (e.g. *F*, *t*, *r*) with confidence intervals, effect sizes, degrees of freedom and *P* value noted<br>*Give P values as exact values whenever suitable.*
☒	☐	For Bayesian analysis, information on the choice of priors and Markov chain Monte Carlo settings
☒	☐	For hierarchical and complex designs, identification of the appropriate level for tests and full reporting of outcomes
☐	☒	Estimates of effect sizes (e.g. Cohen's *d*, Pearson's *r*), indicating how they were calculated

Our web collection on statistics for biologists contains articles on many of the points above.

Software and code

Policy information about availability of computer code

Data collection	Our machine learning models are built in PyTorch 1.11.0 (https://pytorch.org). We make structural predictions of designed sequences with AlphaFold v2.3.1 using localcolabfold v1.5.1 (https://github.com/YoshitakaMo/localcolabfold), ESMFold 2.0.0 (https://github.com/facebookresearch/esm) and OmegaFold v1.1.0 (https://github.com/HeliXonProtein/OmegaFold). Our natural language conditioner makes use of the 125 million parameter GPT-Neo model as available on Hugging Face (https://huggingface.co/EleutherAI/gpt-neo-125m). We construct training datasets based on the PDB (https://www.rcsb.org/), as queried on 2022/03/20, UniProt 2022_01 (https://www.uniprot.org) and PFAM 35 (http://pfam.xfam.org/) . We perform preprocessing with USEARCH (11.0.667) (https://drive5.com/usearch/), mmseq2 13.45111 and pyRosetta 2022.49 (https://www.pyrosetta.org). Our examples of shape conditioning use the Liberation Sans font (https://github.com/liberationfonts/liberation-fonts).

Data analysis

For data analysis, we use Python 3.9.7 (https://www.python.org), NumPy 1.24.3 (https://numpy.org), Pandas 2.0.2 (https://pandas.pydata.org), matplotlib 3.7.1 (https://matplotlib.org) and seaborn 0.12.2 (https://seaborn.pydata.org). We visualize structures with PyMOL 2.5.0 (https://pymol.org/2). For experimental designs, our nanopore sequencing uses Bonito Basecaller 0.6.1 (https://github.com/nanoporetech/bonito), SeqKit v2.3.1 (https://bioinf.shenwei.me/seqkit), Minimap2 v2.23 (https://github.com/lh3/minimap2), samtools v1.16.1 (https://github.com/samtools/samtools) and pysam v0.20.0 (https://github.com/pysam-developers/pysam). We also use the public BeStSel server (https://bestsel.elte.hu) to analyze circular dichroism data. We calculate TM-scores using the 2019/08/22 version of TM-align (https://zhanggroup.org/TM-align/). Our CATH coverage analysis is based on the CATH S40 4.3 and PDB100 clusters on 2023/08/04 (https://cdn.rcsb.org/resources/sequence/clusters/clusters-by-entity-100.txt) , using Foldseek 5-53465f0 (https://github.com/steineggerlab/foldseek). Our novelty analysis using Gauss integral representations employs the Phaistos suite 1.0 (https://sourceforge.net/projects/phaistos/) and umap-learn 0.5.3 (https://github.com/lmcinnes/umap). We use Stride (https://webclu.bio.wzw.tum.de/stride/) for secondary structure contents.

For manuscripts utilizing custom algorithms or software that are central to the research but not yet described in published literature, software must be made available to editors and reviewers. We strongly encourage code deposition in a community repository (e.g. GitHub). See the Nature Portfolio guidelines for submitting code & software for further information.

Data

Policy information about availability of data

All manuscripts must include a data availability statement. This statement should provide the following information, where applicable:

- Accession codes, unique identifiers, or web links for publicly available datasets
- A description of any restrictions on data availability
- For clinical datasets or third party data, please ensure that the statement adheres to our policy

We will not place any restrictions on sharing data from this study. All experimental data, including protein structures that will be deposited in the PDB, will be made available upon publication. All computational results are provided in figures or tables in the main text or supplement.

Research involving human participants, their data, or biological material

Policy information about studies with human participants or human data. See also policy information about sex, gender (identity/presentation), and sexual orientation and race, ethnicity and racism.

Reporting on sex and gender: N/A

Reporting on race, ethnicity, or other socially relevant groupings: N/A

Population characteristics: N/A

Recruitment: N/A

Ethics oversight: N/A

Note that full information on the approval of the study protocol must also be provided in the manuscript.

Field-specific reporting

Please select the one below that is the best fit for your research. If you are not sure, read the appropriate sections before making your selection.

☒ Life sciences ☐ Behavioural & social sciences ☐ Ecological, evolutionary & environmental sciences

For a reference copy of the document with all sections, see nature.com/documents/nr-reporting-summary-flat.pdf

Life sciences study design

All studies must disclose on these points even when the disclosure is negative.

Sample size

In characterizing Chroma-generated proteins computationally, sample sizes were chosen to be sufficient for estimating distributional properties (e.g., 10,000 or 50,000 generated proteins), such as distributions of secondary structure, contact order, and contact densities. The number of designed proteins for experimental characterization was chosen based on a purposefully pessimistic assumption that well-behaved proteins would occur at a frequency of 1% or higher in unfiltered Chroma distribution.

Data exclusions

No data were excluded from analysis in this study.

Replication

Split-GFP screens (FACS and Nanopore sequencing) were performed in biological triplicate for unconditional proteins UNC_001 through UNC_172, and in duplicate for unconditional proteins UNC_173 through UNC_268 and proteins conditioned on secondary structure content.

Randomization

We did not use randomization because it was not applicable to the study design or analysis.

Blinding	We did not use blinding because it was not applicable to the study design or analysis.

Reporting for specific materials, systems and methods

We require information from authors about some types of materials, experimental systems and methods used in many studies. Here, indicate whether each material, system or method listed is relevant to your study. If you are not sure if a list item applies to your research, read the appropriate section before selecting a response.

Materials & experimental systems

n/a	Involved in the study
☐	☒ Antibodies
☒	☐ Eukaryotic cell lines
☒	☐ Palaeontology and archaeology
☒	☐ Animals and other organisms
☒	☐ Clinical data
☒	☐ Dual use research of concern
☒	☐ Plants

Methods

n/a	Involved in the study
☒	☐ ChIP-seq
☒	☐ Flow cytometry
☒	☐ MRI-based neuroimaging

Antibodies

Antibodies used	anti-Strep-tag-HRP (StrepMAB-Classic HRP conjugate, IBA-Lifesciences 2-1509-001)
Validation	We observed bands by western blot at the anticipated protein molecular weights using the anti-Strep-tag-HRP antibody with no other background bands observed, and corroborated its specificity using an orthogonal reagent, Streptactin-HRP (IBA-Lifesciences 2-1502-001). Results using these two reagents are compared in Supplementary Fig. 39.
